# Ion‐Pair Hydrogen Atom Transfer Catalysis Enables Cr‐Catalyzed Allylation of Ketones Using Hydrocarbon Alkenes

**DOI:** 10.1002/anie.202503249

**Published:** 2025-03-16

**Authors:** Suguru Arii, Zonghan Yu, Hongyu Chen, Harunobu Mitsunuma, Motomu Kanai

**Affiliations:** ^1^ Graduate School of Pharmaceutical Sciences The University of Tokyo 7‐3‐1 Hongo Bunkyo‐ku Tokyo 113‐0033 Japan

**Keywords:** Allylation, Chromium, Hydrocarbon alkene, Ion pair, Ketone

## Abstract

We report a catalytic allylation of ketones using simple hydrocarbon alkenes, enabled by a synergistic system comprising chromocene and photo‐activatable ion‐pair hydrogen atom transfer (IP‐HAT) catalysts derived from a thiophosphoryl imide and *N*‐heteroaromatics. This catalyst system generates highly nucleophilic, electron‐rich allylchromium(III) species directly from unactivated alkenes, demonstrating broad applicability to various ketones, including aromatic, aliphatic, and multifunctional ketones. The reaction proceeds under mild conditions with high functional group tolerance. The modular design of the IP‐HAT catalysts allows precise tuning of redox potential and HAT activity, with the enhanced reduction potential of the reduced *N*‐heteroaromatics catalyst playing a pivotal role in efficiently regenerating the electron‐rich chromocene(II) catalyst.

## Graphical abstract

We developed the allylation of ketones using simple alkenes promoted by a chromocene catalyst and an ion‐pair hydrogen atom transfer (IP‐HAT) catalyst. This catalytic system can be applied to a wide range of ketones, including aliphatic ketones. The reaction proceeds under mild conditions with high functional group tolerance.

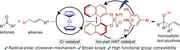

Tertiary homoallylic alcohols are versatile synthetic intermediates and building blocks in organic synthesis.^[^ ^1^ ^]^ The catalytic allylation of ketones using alkenes through sp^3^ C─H bond functionalization is an ideal yet challenging approach to accessing these compounds. Compared to aldehydes, ketones exhibit attenuated electrophilicity and increased steric hindrance, making catalytic allylation of ketones highly demanding, especially using unactivated alkenes as pronucleophiles.

Traditional catalytic intermolecular ketone allylations often require activated allyl sources as pronucleophiles, such as allylic organometallic reagents,^[^ ^2^, ^3^, ^4^, ^5^, ^6^, ^7^, ^8^, ^9^ ^]^ allylic halides,^[^ ^10^, ^11^, ^12^, ^13^ ^]^ or allylic acetates^[^ ^14^, ^15^, ^16^ ^]^ (Scheme 1a). In contrast, Krische reported a coupling reaction of hydrocarbon dienes with secondary alcohols^[^ ^17^, ^18^ ^]^ or ketones^[^ ^19^ ^]^ via a hydride transfer mechanism, yielding the ketone allylation products (Scheme 1b). However, this reaction was only applicable to alcohol substrates, which produce activated ketone intermediates such as pyridyl ketones and isatins. Sato and Mita developed a low‐valent cobalt complex‐catalyzed branch‐selective allylation of ketones, generating allylcobalt species directly from alkenes without pre‐activation (Scheme 1c).^[^ ^20^, ^21^ ^]^ Wang and co‐workers achieved the allylation of isatins via allyliron species using a combination of a cationic iron catalyst and a stoichiometric amount of amine base (Scheme 1d).^[^ ^22^ ^]^ These approaches required stoichiometric amounts of Lewis acids (AlMe_3_ or BF_3_•OEt_2_) for catalyst turnover. Photocatalytic methods have been reported, involving the coupling of allyl and ketyl radicals, generated via allylic sp^3^ C─H bond scission of alkenes by a hydrogen atom transfer (HAT) catalyst and single‐electron ketone reduction by the photoredox catalyst, respectively (Scheme 1e)^[^ ^23^, ^24^, ^25^ ^]^ (we reported a photoredox/HAT/titanium ternary hybrid catalyst system that promoted an intermolecular addition reaction of cyclohexene with ketones through sp^3^ C–H bond activation. See ref. [26]; however, the reaction with linear alkenes provided a mixture of regioisomers. For more details, see the Supporting Information section). While this approach proceeded under mild conditions, the substrate scope was limited due to the difficulty in the single‐electron reduction of ketones. Additionally, the branch/linear selectivity was not satisfactory for unsymmetrical alkenes. All the previous examples through C─H bond functionalization (Scheme 1b,e) were not applicable to the reactions of aliphatic ketones with simple unactivated alkenes.

**Scheme 1:**
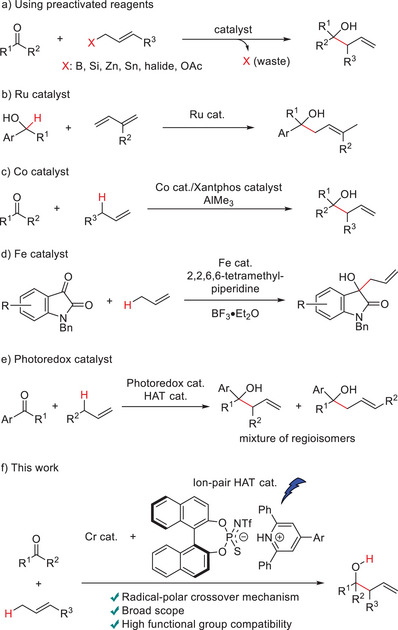
Catalytic Allylations of Ketones.

Herein, we present a catalytic allylation of ketones with unactivated hydrocarbon alkenes through a radical‐polar crossover mechanism (Scheme 1f). The key to this transformation is the catalytic generation of highly nucleophilic allylchromium(III) species enabled by the synergistic combination of ion‐pair HAT (IP‐HAT) and chromocene catalysis. The reaction proceeds with complete branch selectivity, operates under mild conditions, and is applicable to both aromatic and aliphatic ketones. Furthermore, the reaction exhibits excellent functional group tolerance, enabling its application to multifunctional substrates.

We previously reported a catalytic allylation of aldehydes with hydrocarbon alkenes by developing a ternary catalyst system comprised of a photoredox catalyst (PC^+^: e.g., **PC1**), a thiophosphoryl imide (TPI) catalyst, and a CrCl_2_ catalyst.^[^ ^27^, ^28^, ^29^, ^30^, ^31^, ^32^, ^33^, ^34^, ^35^ ^]^ The catalytic cycle involved five main steps (Figure 1a): 1) single‐electron oxidation of TPI by photoexcited photoredox catalyst (PC^+^*) to generate HAT‐active thiyl radical **1** and the reduced photoredox catalyst (PC); 2) allylic sp^3^ C─H bond cleavage of alkenes **2** by thiyl radical **1** to generate allyl radical **3**; 3) intercepting **3** with Cr(II) to generate allylchromium(III) **4**; 4) nucleophilic addition of **4** to aldehydes, followed by protonolysis of the resulting chromium alkoxide to generate the secondary homoallylic alcohol products; and 5) reduction of Cr(III) by PC to regenerate Cr(II) and PC^+^ to close the catalytic cycle).

**Figure 1:**
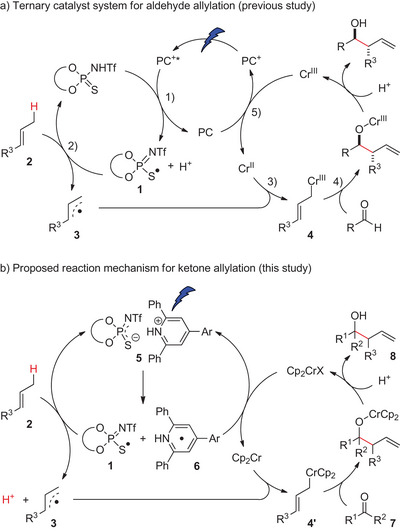
a) Catalytic cycles for aldehyde allylation and b) ketone allylation.

Based on this mechanism, we began the optimization of ketone allylation reaction conditions using ketone **7a** and 2‐butene (**2a**: *E*‐ and *Z*‐mixture) as model substrates (Table 1). Initially, employing previously reported conditions for aldehydes (i.e., **PC1** and **TPI1** as photoredox and HAT catalysts, respectively, under blue light irradiation),^[^ ^27^ ^]^ we observed no desired product formation, likely due to the insufficient nucleophilicity of the allylchromium species formed under those conditions (entry 1). Consequently, we explored various chromium catalysts.^[^ ^36^, ^37^, ^38^ ^]^ While CrCl_3_•3THF (tetrahydrofuran) and Cr(O*t*Bu)_3_ failed to promote the reaction (entries 2 and 3), CpCrCl_2_, which contains an electron‐rich cyclopentadienyl (Cp) ligand, yielded product **8a** in 13% yield (entry 4). Use of Cp_2_Cr (for the use of Cp_2_Cr in Nozaki–Hiyama–Kishi–Takai allylation of aldehydes with allyl halides, see ref. [39]) further improved the yield to 26% (entry 5). The reaction exhibited complete branch selectivity (**8a**/**9a** = >20/1).

**Table 1:** Optimization of reaction conditions.^a)^

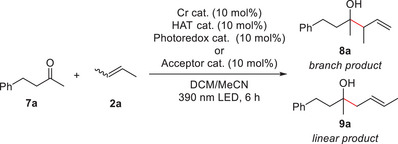					
Entry	Cr cat.	HAT cat.	Photoredox cat. or Acceptor cat.	Yield (%)	b/l (**8a**/**9a**)
1^b)^	CrCl_2_	**TPI1**	**PC1**	0	ND
2^b)^	CrCl_3_•3THF	**TPI1**	**PC1**	0	ND
3^b)^	Cr(O*t*Bu)_3_	**TPI1**	**PC1**	0	ND
4^b)^	CpCrCl_2_	**TPI1**	**PC1**	13	>20/1
5^b)^	Cp_2_Cr	**TPI1**	**PC1**	26	>20/1
6	Cp_2_Cr	**TPI1**	9‐phenylacridine	0	ND
7	Cp_2_Cr	**TPI1**	isoquinoline	38	>20/1
8	Cp_2_Cr	**TPI1**	**TPP1**	65	>20/1
9	Cp_2_Cr	**TPI1**	**TPP2**	91	>20/1
10	Cp_2_Cr	**TPI1**	**TPP3**	0	ND
11	Cp_2_Cr	**TPI1**	**TPP4**	38	>20/1
12	Cp_2_Cr	**TPI1**	**TPP5**	72	>20/1
13	Cp_2_Cr	**TPI1**	**TPP6**	54	>20/1
14	Cp_2_Cr	**TPI1**	**TPP7**	58	>20/1
15	Cp_2_Cr	**TPI2**	**TPP2**	66	>20/1
16	Cp_2_Cr	**TPI3**	**TPP2**	8	>20/1
17	Cp_2_Cr	**TPI4**	**TPP2**	84	>20/1
18	Cp_2_Cr	**TPI5**	**TPP2**	50	>20/1
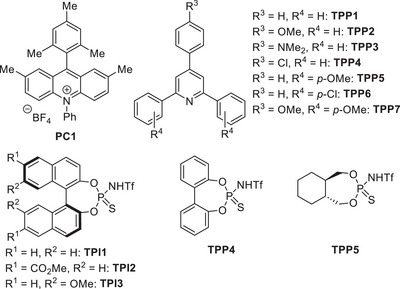					

^a)^ Standard conditions: **7a** (1 equiv.), 2‐butene (*E*‐ and *Z*‐mixture, 0.25 mL as a liquid), Cr catalyst (10 mol%), HAT catalyst (10 mol%), and photoredox catalyst or acceptor catalyst (10 mol%) were reacted in DCM/MeCN (9/1, 1.25 mL) at room temperature under LED light irradiation for 6 h. Yield and b/l were determined by ^1^H NMR analysis of the crude mixture using 1,1,2,2‐tetrachloroethane as an internal standard. b/l = branch (**8a**)/linear (**9a**) ratio. ND = not determined.

^b)^ K_2_HPO_4_ (10 mol%) was added. Reactions were conducted under blue Light irradiation.

**Table 2:** Substrate scope of ketones.^a)^

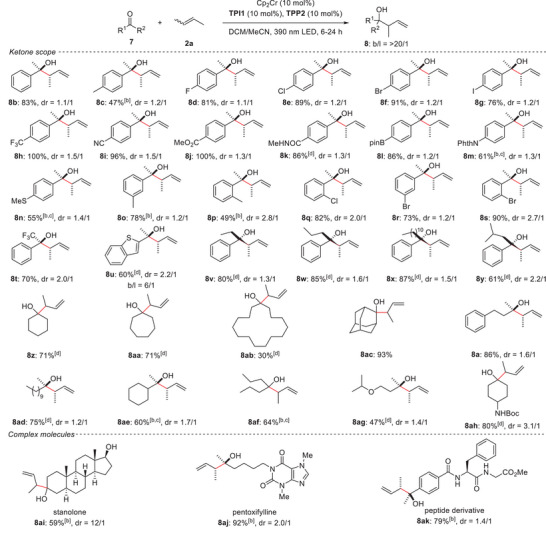

^a)^ General conditions: **7** (0.125 mmol), 2‐butene (*E*‐ and *Z*‐mixture, 0.25 mL as a liquid), Cp_2_Cr (0.0125 mol), **TPI1** (0.0125 mol), and **TPP2** (0.0125 mol) were reacted in DCM/MeCN (9/1, 1.25 mL) at room temperature under 390 nm LED light irradiation for 6 h. Yield was the isolated yield.

^b)^ Reaction time was 24 h.

^c)^ 20 mol% of Cp_2_Cr (0.025 mol) was used.

^d)^ Reaction time was 12 h.

**Table 3:** Substrate scope of alkenes.^a)^

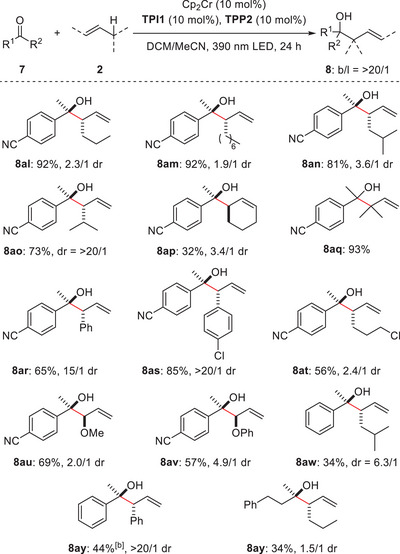

^a)^ General conditions: **7** (0.125 mmol), **2** (0.625 mmol), Cp_2_Cr (0.0125 mol), **TPI1** (0.0125 mol), and **TPP2** (0.0125 mol) were reacted in DCM/MeCN (9/1, 1.25 mL) at room temperature under 390 nm LED light irradiation for 24 h. Yield was the isolated yield.

^b)^ 20 mol% of Cp_2_Cr (0.025 mol) was used.

The moderate yields were attributed to the inefficient reducing ability of the reduced acridinium photoredox catalyst (PC: *E*_1/2_(PC/PC^+^) = −0.58 V vs. SCE)^[^ ^40^ ^]^ for the Cr(III) state (step 5 described above, Figure 1a) due to the increased electron density of the Cr catalyst by the presence of Cp ligands (*E*_red_(Cp_2_CrCl) = −0.91 V vs. SCE, see Figure 2c). We therefore screened some photoredox catalysts with varying redox potential, yet all resulted in no reaction (see Supporting Information for details).

**Figure 2:**
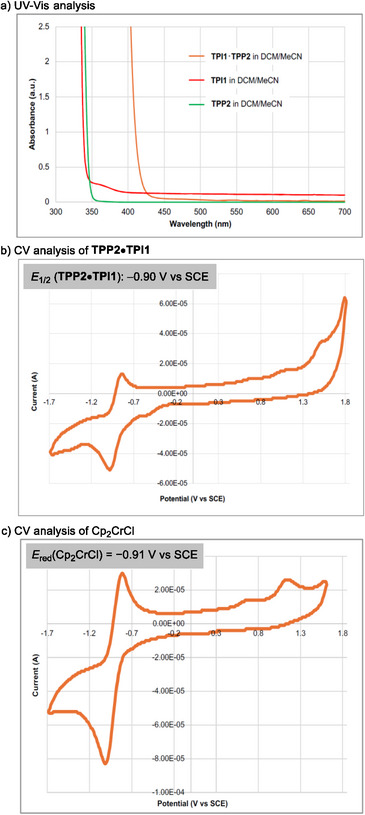
UV–vis spectroscopy and CV analysis.

Subsequently, we explored photoactivatable ion‐pair complexes comprised of *N*‐heteroaromatic electron‐acceptors and **TPI1** as HAT catalyst precursors (such as **5** in Figure 1b). We hypothesized that photoirradiation to an ion‐pair complex catalyst promotes single electron transfer from the **TPI1** anion to the protonated *N*‐heteroaromatics, generating HAT‐active TPI radical **1** and a reduced, protonated *N*‐heteroaromatics (such as **6** in Figure 1b).^[^ ^41^ ^]^ The reduced *N*‐heteroaromatics could exhibit sufficient potential to reduce Cp_2_Cr(III) for regenerating Cp_2_Cr(II).

While 9‐phenylacridine showed no reactivity,^[^ ^42^, ^43^ ^]^ a combination of isoquinoline^[^ ^41^ ^]^ with **TPI1** afforded **8a** in 38% yield (entries 6,7). Through further optimization, we identified triphenylpyridine **TPP1** as an effective catalyst,^[^ ^44^ ^]^ delivering **8a** in 65% yield (entry 8). Modifying the TPP structure revealed that **TPP2**, featuring a methoxy group at the R^3^ position, exhibited the highest catalytic performance, affording **8a** in 91% yield (entry 9). In contrast, introducing a dimethylamino group (**TPP3**) or an electron‐withdrawing chlorine atom at the R^3^ position (**TPP4**) resulted in diminished yield (entries 10,11). Furthermore, various electron‐donating and electron‐withdrawing substituents were examined at the R^4^ position; however, none led to an improvement in yield (entries 12‒14). Subsequent structural optimizations of TPI catalysts were investigated. However, introducing electron‐donating or electron‐withdrawing groups into the binaphthyl backbone, as in **TPI2** and **TPI3**, showed no enhancement in yield (entries 15,16). The reaction also proceeded with backbones such as biphenyl (**TPI4,** entry 17) or aliphatic thiophosphoryl imide (**TPI5**, entry 18). Nevertheless, **TPI1** consistently afforded the highest yield, underscoring its superior catalytic efficiency.

Under the optimized conditions, the substrate scope was examined. First, the generality of ketones was evaluated (Table 2). The reaction proceeded smoothly with acetophenone, affording the desired product **8b**. Acetophenone derivatives bearing various substituents at the *para*‐position, including halogens, electron‐withdrawing groups, and electron‐donating groups, also underwent the reaction with high to moderate yield (**8c**‒**8n**). Substrates with substituents at the *meta*‐ and *ortho*‐positions similarly exhibited successful reactivity (**8o**‒**8s**). Notably, 2,2,2‐trifluoroacetophenone reacted efficiently with **2a**, producing the desired product **8t** in 70% yield. A heteroaryl ketone was also tolerated under the reaction conditions (**8u**). The method was successfully extended to aromatic ketones with longer alkyl substituents than the methyl group (**8v**‒**8y**). Aliphatic ketones, including cyclic and linear ketones, were competent (**8a**, **8z**‒**8ah**). Sterically hindered, less reactive ketones underwent the reaction upon increasing the catalyst loading (**8ae**, **8af**). Functional groups such as ethers and amides were tolerated, highlighting the robustness of the catalyst system (**8ag**, **8ah**). Crotylation reactions proceeded efficiently with biologically active molecules, such as stanolone (**8ai**), pharmaceuticals like pentoxifylline (**8aj**), and a peptide derivative (**8ak**), demonstrating the potential for functionalization of complex molecules. It is noteworthy that this reaction exhibits exceptionally high branch selectivity in all the cases. However, diastereoselectivity of crotylation using 2‐butene was generally low, which must be improved in future studies.

Next, the substrate scope of alkenes was explored (Table 3). 1‐Hexene and 1‐decene were successfully employed (**8al**, **8am**). Alkenes with bulky isopropyl groups reacted smoothly (**8an**, **8ao**). Notably, when 4‐methyl‐1‐pentene was employed, the reaction proceeded with perfect diastereoselectivity (dr = >20/1, **8ao**). Cyclic alkenes were also proved compatible (**8ap**). Furthermore, the reaction with 3‐methyl‐1‐butene proceeded smoothly, constructing sterically congested contiguous tetrasubstituted‐quaternary carbon centers with high efficiency (**8aq**). Despite significant steric hindrance, no linear products were observed. The use of allylbenzene derivatives resulted in the diastereoselective formation of target products (**8ar**, **8as**). Alkenes containing functional groups, such as chlorides and ethers, participated in the reaction without complications (**8at**‒**8av**). The scope was successfully extended to acetophenone and an aliphatic ketone (**8aw**‒**8ay**), thereby demonstrating its broad applicability.

To gain insights into the reaction mechanism, we conducted the following experiments. First, the absorption property of the IP‐HAT catalyst was analyzed using UV–vis spectroscopy (Figure 2a). The results revealed that while **TPI1** and **TPP2** individually showed no absorption at 390 nm, their coexistence caused a significant bathochromic shift, resulting in absorption at this wavelength. This observation suggests that the acid–base ion pair formation between **TPI1** and **TPP2** contributes to the absorption at 390 nm. Furthermore, cyclic voltammetry (CV) measurements indicated that the ion pair exhibits *E*_1/2_ = −0.90 V versus SCE (Figure 2b), supporting the feasibility of reducing Cp_2_Cr(III) to Cp_2_Cr(II) (*E*_red_(Cp_2_CrCl) = −0.91 V versus SCE, Figure 2c). Thus, the IP‐HAT catalyst generated a stronger reductant derived from **TPP2** (**6** in Figure 1b) by photo‐induced single electron transfer within the ion pair, surpassing the reductive strength of the previous ternary catalyst system,^[^ ^27^ ^]^ which includes a photoredox catalyst (PC in Figure 1a: *E*_1/2_(PC/PC^+^) = −0.58 V vs. SCE^[^ ^40^ ^]^). This enhanced reductive capability is the key to the efficient regeneration of the electron‐rich Cp_2_Cr catalyst. Furthermore, the initial kinetics using the **TPI1**/**TPP2** IP‐HAT catalyst was greater than that using the previous **TPI1**/**PC1** catalyst system for the allylation of an aldehyde, where HAT is the rate‐determining step^[^ ^27^ ^]^ (Figure S2). This result suggests that the IP‐HAT catalysis enhances the HAT process, in addition to facilitating the regeneration of the Cp_2_Cr catalyst.

Based on these observations, the proposed reaction mechanism is depicted in Figure 1b. Initially, **TPI1** and **TPP2** form ion pair **5**, which undergoes excitation at 390 nm, enabling efficient single‐electron transfer. This process generates the HAT‐active thiyl radical **1** and pyridine radical **6**. Thiyl radical **1** abstracts allylic C─H bonds from alkene substrates **2**, producing allyl radical **3**. Radical **3** is subsequently captured by Cp_2_Cr at the sterically less congested terminal carbon atom, forming the highly nucleophilic allylchromium(III) species **4′**. The addition of **4′** to ketones **7** occurs in a branch‐selective manner, likely through a linear transition state.^[^ ^45^ ^]^ The resulting chromium alkoxides are protonated to yield products **8** and Cp_2_CrX (where X may be the **TPI1** anion). Finally, pyridine radical **6** has a sufficient reduction potential to regenerate Cp_2_Cr from Cp_2_CrX, completing the catalytic cycle.

In summary, we have successfully developed a catalyst system combining IP‐HAT catalysis and chromocene to enable the allylation of ketones with unactivated, readily available hydrocarbon alkenes. The reaction exhibits high branch selectivity and broad applicability across a variety of ketones and alkenes. Notably, this is the first catalytic allylation of aliphatic ketones through C─H functionalization of simple alkenes. Operating under mild conditions with high functional group tolerance, the system is also compatible with complex ketone substrates. The modular design of the IP‐HAT catalysts enables precise tuning of redox potential and HAT activity, playing a dual role: efficiently generating the HAT‐active radical required for allylic sp^3^ C─H activation and facilitating the reduction of the electron‐rich Cp_2_Cr(III) species. This dual functionality supports the generation and turnover of highly nucleophilic allylchromium intermediates. Building on these findings, we are currently exploring an asymmetric variant of this transformation.

## Conflict of Interests

The authors declare no conflict of interest.

## Supporting information



Supporting Information

## Data Availability

Data sharing is not applicable to this article as no new data were created or analyzed in this study.
