# Supplementary material for: Ion‐Pair Hydrogen Atom Transfer Catalysis Enables Cr‐Catalyzed Allylation of Ketones Using Hydrocarbon Alkenes
Source: Angew Chem Int Ed Engl. 2025 Mar 16;64(21):e202503249. doi: 10.1002/anie.202503249 (PMC12087839; doi:10.1002/anie.202503249)
Supplement: Supplementary file 1 — Supporting Information [file ANIE-64-e202503249-s001.pdf]

Supporting Information

Ion-Pair Hydrogen Atom Transfer Catalysis Enables Cr-Catalyzed Allylation of Ketones Using Hydrocarbon Alkenes

Suguru Arii,[a] Zonghan Yu, [a] Hongyu Chen, [a] Harunobu Mitsunuma,*[a] and Motomu Kanai*[a]

[a]*Graduate School of Pharmaceutical Sciences, The University of Tokyo, 7-3-1 Hongo, Bunkyo-ku, Tokyo 113-0033 (Japan)*

*e-mail: h-mitsunuma@mol.f.u-tokyo.ac.jp, kanai@mol.f.u-tokyo.ac.jp

Contents

1. General Method
2. Optimization of Reaction Conditions
3. Catalytic Allylation of Acetophenone with a Photoredox/HAT/Titanium Ternary Hybrid Catalyst System
4. Preparation of Substrates and Catalysts
5. General Procedure for Catalytic Allylation of Ketones and Characterization Data
6. UV-Vis analysis
7. Acceleration of HAT Process by Ion-pair Catalyst
8. Reaction with Ketoesters
9. References
10. NMR Charts

1. General Method

^{1}H NMR, ^{13}C NMR and ^{19}F NMR spectra were recorded on JEOL ECX500 (500.16 MHz for ^{1}H NMR and 125.77 MHz for ^{13}C NMR), and JEOL ECS400 (391.78 MHz for ^{1}H NMR, 98.52 MHz for ^{13}C NMR and 368.64 MHz for ^{19}F NMR) spectrometer. For ^{1}H NMR and ^{13}C NMR, chemical shifts were reported in the scale relative to $CDCl_3$ (δ = 7.26 for 1H NMR and δ = 77.0 for ^{13}C NMR) used as an internal reference. Electrospray ionization (ESI)-mass spectra were measured on a JEOL JMS-T100LC AccuTOF spectrometer for HRMS. Infrared (IR) spectra were recorded on a JASCO FT/IR 410 Fourier transform infrared spectrophotometer. Column chromatography was performed with silica gel Merck 60 (230-400 mesh ASTM), Biotage Isolera One and Biotage SNAP Ultra, or Yamazen Smart Flash and Universal Column Premium. All non-commercially available compounds were prepared and characterized as described in Section 3 of this SI. Other reagents were purchased from Aldrich, Tokyo Chemical Industry Co., Ltd. (TCI), Kanto Chemical Co., Inc., Wako Pure Chemical Industries, Ltd., and Strem Chemicals, Inc. and were used as received. A Valore VBP-L24-C2 with 38W LED lamp (VBL-SE150-BBB (430)) was used as the 430 nm light source. A Kessil PR160 LED Photo Reaction Lighting PR160-390 nm was used as the 390 nm light source.

2. Optimization of Reaction Conditions

Table S1. Screening of photoredox catalysts for catalytic allylation of ketones

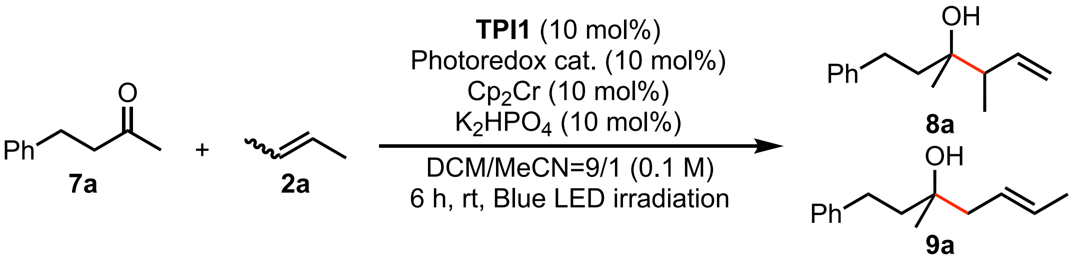

entry	Photoredox cat.	E_{red} (V *vs.* SCE)	yield (%)	b/l (**8a**/**9a**)
1	**PC1**	-0.58	0	ND
2	**PC2**	-0.82	0	ND
3	4CzIPN	-1.24	0	ND
4	$Ru(phen)_3Cl_2$	-1.36	0	ND
5	$[Ir(dF\text{-}CF_3\text{-}ppy)(dtbppy)_2]PF_6$	-1.37	0	ND
6	perylene	-1.67	0	ND

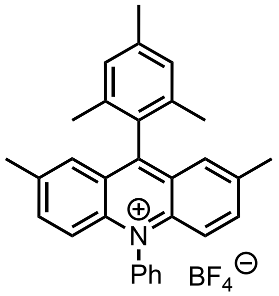

PC1
E_{ox} = +2.09 V
E_{red} = -0.58 V

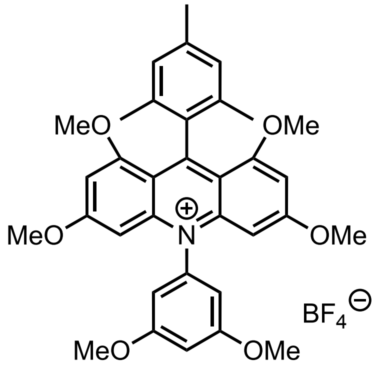

PC2
E_{ox} = +1.65 V
E_{red} = -0.82 V

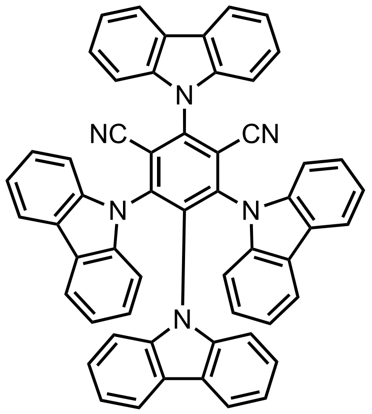

4CzIPN
E_{ox} = +1.49 V
E_{red} = -1.24 V

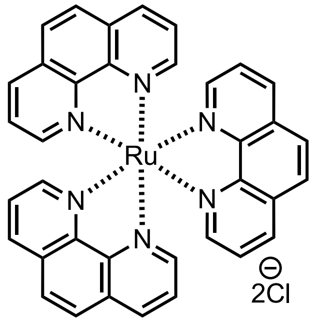

$Ru(phen)_3Cl_2$
E_{ox} = +1.30 V
E_{red} = -1.36 V

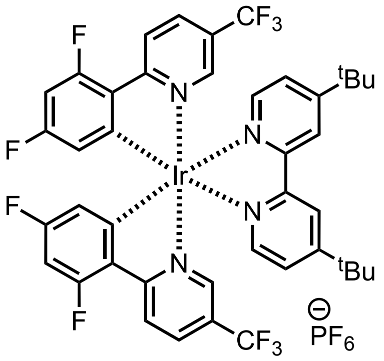

$[Ir(dF\text{-}CF_3\text{-}ppy)(dtbppy)_2]PF_6$
E_{ox} = +1.21 V
E_{red} = -1.37 V

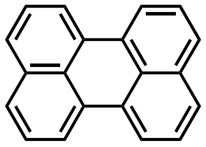

perylene
E_{ox} = +0.85 V
E_{red} = -1.67 V

The moderate yield in allylation reaction with Cp_2Cr (Table 1, entry 5) was attributed to the inefficient reducing ability of the reduced **PC1** ($E_{1/2}$(**PC1**/**PC1**$^+$) = −0.58 V vs. SCE)[1)] for the Cr(III) state due to the increased electron density of the Cr catalyst by the presence of Cp ligands (E_{red}(Cp_2CrCl) = −0.91 V vs. SCE, see Figure 2c). We therefore screened photoredox catalysts with varying redox potential[2)], yet all resulted in low yields (Table S1).

3. Catalytic allylation of ketone with a photoredox/HAT/titanium ternary hybrid catalyst system

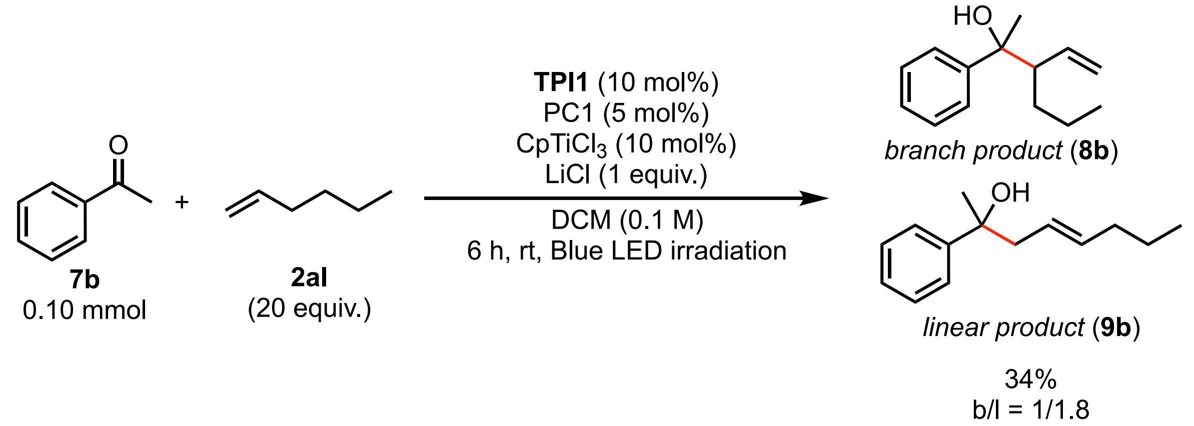

We reported a photoredox/HAT/titanium ternary hybrid catalyst system that promoted an intermolecular addition reaction of cyclohexene with ketones through sp^3 C–H bond activation.[3)] However, a mixture of regioisomers was obtained using linear alkenes.

4. Preparation of Substrates and Catalysts

4-1. Synthesis of substrates

4-Acetyl-*N*-methylbenzamide (**7k**), 2-(4-acetylphenyl)isoindoline-1,3-dione (**7m**) and 1-allyl-4-chlorobenzene (**2as**) were prepared according to the reported method.[4),5),6)] Methyl *L*-phenylalanylglycinate 2,2,2-trifluoroacetate (starting material for **7ak**) was prepared according to the reported method.[7)]

4-1-1. Synthesis of methyl (4-acetylbenzoyl)-*L*-phenylalanylglycinate (**7ak**)

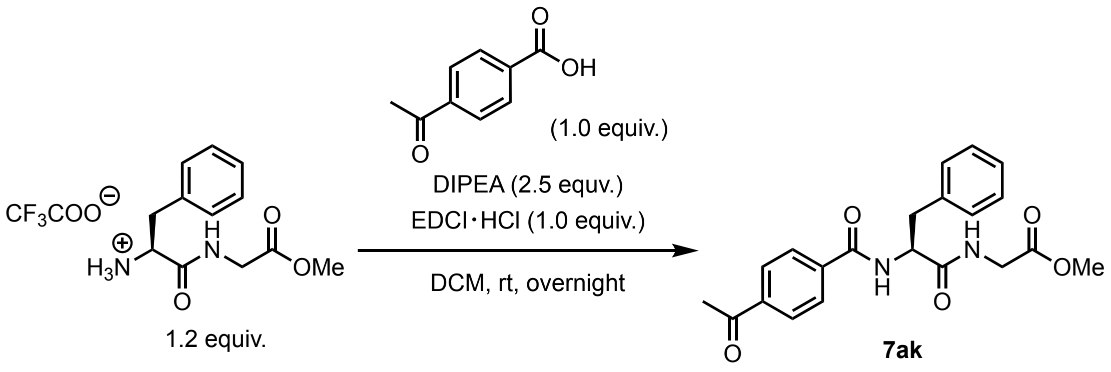

To a solution of methyl *L*-phenylalanylglycinate 2,2,2-trifluoroacetate (453 mg, 1.29 mmol), 4-acetylbenzoic acid (177 mg, 1.08 mmol) and EDCI•HCl (207 mg, 1.08 mmol) in CH_2Cl_2 (5 mL), diisopropylethylamine (0.725 mL, 4.16 mmol) was added at 0 °C. After overnight stirring at room temperature, water was added and organic materials were extracted with CH_2Cl_2 three times. The combined organic layers were washed with brine, dried over Na_2SO_4, filtered, and concentrated under reduced pressure. The crude material was purified by silica gel flash column chromatography (MeOH/Et_2O = 5%, v/v) to afford **7ak** (47.0 mg, 0.123 mmol) in 11% yield as a white solid.

Methyl (4-acetylbenzoyl)-*L*-phenylalanylglycinate (7ak)

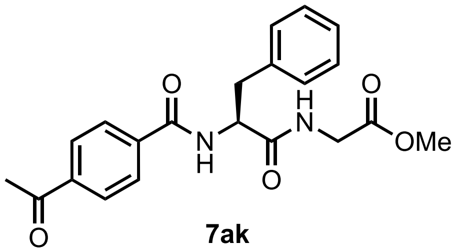

7ak

^{1}H NMR ($CDCl_3$, 500 MHz): δ 7.98 (2H, d, J = 8.6 Hz), 7.79 (2H, d, J = 8.6 Hz), 7.33-7.23 (5H, m), 6.94 (1H, d, J = 7.4 Hz), 6.38 (1H, br s), 4.92-4.91 (1H, m), 4.03-3.95 (2H, m), 3.73 (3H, s), 3.26-3.18 (2H, m), 2.62 (3H, s); ^{13}C NMR ($CDCl_3$, 99 MHz): δ 197.5, 171.5, 169.9, 166.6, 139.4, 137.6, 136.5, 129.4, 128.7, 128.5, 127.6, 127.2, 54.9, 52.5, 41.3, 38.3, 26.9 ; m/z calcd for $C_{21}H_{22}N_2O_5Na$ $[M+Na]^+$ 405.1426. Found 405.1426.; IR (neat): 3322, 3281, 1746, 1689, 1642, 1534, 1430, 1367, 1273, 1208, 1187, 1022, 983, 962, 845, 751, 690, 607 cm^{-1}

4-2. Synthesis of catalysts

TPI1 was prepared according to the reported method.[8),] **TPI2-TPI5** were prepared according to a related procedure.[9)] **TPP2-TPP7** were synthesized according to the following general procedure for TPPs. $CpCrCl_2$ was prepared according to the reported method.[10)]

General synthetic procedure for TPPs

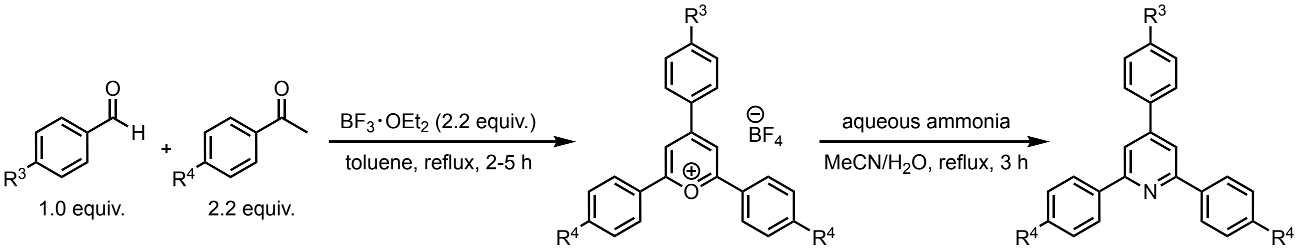

An aldehyde (1.0 equiv.) and a ketone (2.2 equiv.) were added to a flame-dried round-bottom flask with toluene. Then, boron trifluoride diethyl ether complex (2.2 equiv.) was added to the mixtures dropwise at room temperature. The reaction mixtures were stirred under reflux for 2-5 hours. The reaction mixtures were cooled to room temperature and concentrated under reduced pressure. The crude mixture was dissolved in acetone. This solution was poured into Et_2O. The precipitated solid was filtered out and washed with Et_2O to afford a triphenylpyrylium derivative. To a solution of triphenylpyrylium derivative was added aqueous ammonia/ MeCN (1/1). After one hour, half the volume of aqueous ammonia as before was added. The reaction mixture was cooled to room temperature. Water was added and organic materials were extracted with CH_2Cl_2 three times. Combined organic layers were washed with brine, dried over Na_2SO_4, filtered, and concentrated under reduced pressure. The crude material was purified by silica gel flash column chromatography. Fractions including the product were collected and concentrated under reduced pressure. The resulting solid was washed with Et_2O to obtain TPP as a white solid.

5. General Procedure for Catalytic Allylation of Ketones and Characterization Data

5-1. Procedure for preparation of 8a-ak and 8aq

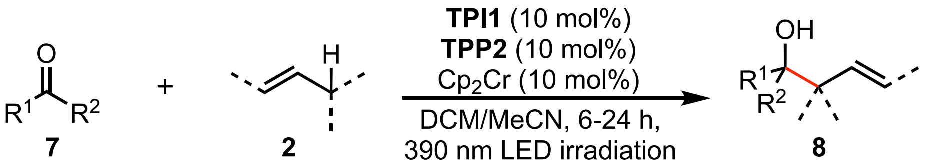

In an argon-filled glove box, Cp_2Cr (2.3 mg, 0.0125 mmol, 10 mol%), **TPI1** (6.2 mg, 0.0125 mmol, 10 mol%) and **TPP2** (4.2 mg, 0.0125 mmol, 10 mol%) were dissolved in degassed CH_2Cl_2 (1.125 mL) and MeCN (0.125 mL) in a screw-capped test tube. Then, ketone **7** (0.125 mmol, 1.0 equiv.) was added to the reaction mixture. The reaction tube was removed from the glove box and the reaction mixture was cooled to –78 ºC. Liquid alkene **2** (ca. 0.250 μL) cooled at –78 ºC was added to the reaction mixture via syringe. The reaction mixture was warmed to room temperature and subjected to 390 nm LED irradiation by a Kessil PR160 LED Photo Reaction Lighting PR160-390 nm for 6-24 hours cooling with a fan. Then, to the reaction mixture, 1 N HCl aqueous solution was added. Organic materials were extracted with CH_2Cl_2 three times. Combined organic layers were dried over Na_2SO_4 and filtered. After evaporation, the diastereomeric ratio was determined by ^{1}H NMR analysis. The residue was purified by silica gel flash column chromatography to afford the target tertiary homoallylic alcohols **8**.

3,4-Dimethyl-1-phenylhex-5-en-3-ol (8a)

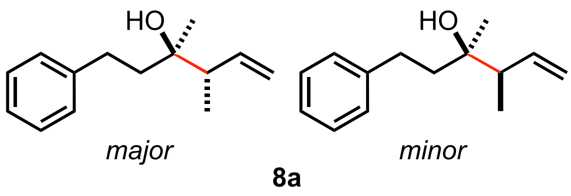

The reaction was conducted using 4-phenylbutan-2-one (**7a**) (18.7 μL, 0.125 mmol, 1.0 equiv.) and liquid 2-butene (**2a**) (ca. 250 μL) for 6 hours. The residue was purified by silica gel flash column chromatography (Et_2O/hexane = 20%, v/v) to afford **8a** as a colorless oil (21.9 mg, 86%, dr = 1.6/1).

^{1}H NMR ($CDCl_3$, 500 MHz): diastereomeric mixture δ 7.30-7.28 (2H, m), 7.22-7.18 (3H, m), 5.91-5.77 (1H, m), 5.15-5.11 (2H, m), 2.81-2.64 (2H, m), 2.37-2.31 (1H, m), 1.82-1.69 (2H, m), 1.22 (3H, s, *minor*), 1.18 (3H, s, *major*), 1.07 (3H, d, *J* = 6.9 Hz, *minor*), 1.06 (3H, d, *J* = 6.9 Hz, *major*); ^{13}C NMR ($CDCl_3$, 126 MHz) : diastereomeric mixture δ 142.9, 140.4, 140.2, 128.5, 128.5, 125.9, 116.9, 116.5, 73.9, 73.7, 48.4, 47.6, 42.2, 41.7, 30.0, 24.0, 23.6, 15.1, 14.8; m/z calcd for $C_{14}H_{20}O_5Na^+$ [M+Na]$^+$ 227.1412. Found 227.1412; IR (neat): 3566, 3420, 2953, 1645, 1609, 1495, 1455, 1381, 917, 743, 703 cm^{-1}

All the spectroscopic data were matched with the previously reported data[11)].

3-Methyl-2-phenylpent-4-en-2-ol (8b)

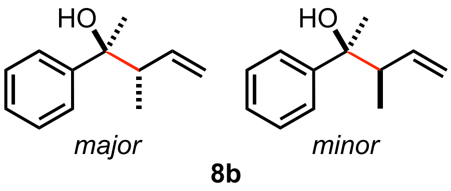

The reaction was conducted using acetophenone (**7b**) (14.6 μL, 0.125 mmol, 1.0 equiv.) and liquid 2-butene (**2a**) (ca. 250 μL) for 6 hours. The residue was purified by silica gel flash column chromatography (Et_2O/hexane = 10%, v/v) to afford **8b** as a colorless oil (18.2 mg, 83%, dr = 1.1/1).

^{1}H NMR ($CDCl_3$, 500 MHz): diastereomeric mixture δ 7.43-7.41 (2H, m), 7.35-7.33 (2H, m), 7.25-7.22 (1H,

m), 5.82 (1H, ddd, J = 17.8, 9.7, 7.4 Hz, *minor*), 5.71 (1H, ddd, J = 17.6, 10.2, 7.0 Hz, *major*), 5.13-5.09 (2H, m), 2.60-2.56 (1H, m), 1.96 (1H, s, *major*), 1.86 (1H, s, *minor*), 1.53 (3H, s), 0.97 (3H, d, J = 6.9 Hz, *major*), 0.87 (3H, d, J = 6.9 Hz, *minor*); ^{13}C NMR ($CDCl_3$, 126 MHz): diastereomeric mixture δ 147.2, 147.1, 140.1, 140.0, 128.0, 126.8, 126.6, 125.6, 125.3, 116.8, 116.5, 75.9, 75.8, 49.1, 48.9, 28.7, 26.0, 14.9, 14.2; m/z calcd for $C_{12}H_{16}ONa$ $[M+Na]^+$ 199.1099. Found 199.1092; IR (neat): 3593, 2980, 1642, 1599, 1492, 1448, 1371, 1274, 1071, 1024, 927, 733, 700 cm^{-1}

All the spectroscopic data were matched with the previously reported data[12)].

3-Methyl-2-(*p*-tolyl)pent-4-en-2-ol (8c)

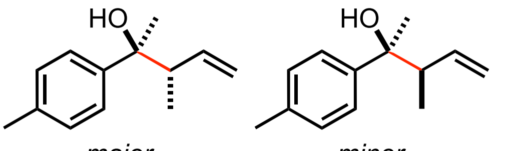

major *minor*

8c

The reaction was conducted using 1-(*p*-tolyl)ethan-1-one (**7c**) (16.7 μL, 0.125 mmol, 1.0 equiv.) and liquid 2-butene (**2a**) (ca. 250 μL) for 24 hours. The residue was purified by silica gel flash column chromatography (Et_2O/hexane = 20%, v/v) to afford **8c** as a colorless oil (11.3 mg, 47%, dr = 1.2/1).

^{1}H NMR ($CDCl_3$, 392 MHz): diastereomeric mixture δ 7.33-7.27 (2H, m), 7.15 (2H, d, J = 8.5 Hz), 5.85-5.67 (1H, m), 5.13-5.07 (2H, m), 2.59-2.53 (1H, m), 2.34 (3H, s), 1.92 (1H, s, *major*), 1.83 (1H, s, *minor*), 1.51 (3H, s), 0.96 (3H, d, J = 7.2 Hz, *major*), 0.87 (3H, d, J = 7.2 Hz, *minor*); ^{13}C NMR ($CDCl_3$, 99 MHz): diastereomeric mixture δ 144.2, 140.2, 140.1, 136.3, 136.1, 128.7, 125.5, 125.3, 116.7, 116.4, 75.8, 75.7, 49.1, 48.9, 28.7, 26.0, 21.1, 15.0, 14.3; m/z calcd for $C_{13}H_{18}ONa$ $[M+Na]^+$ 213.1255. Found 213.1253; IR (neat): 3066, 2966, 2266, 1518, 1378, 1268, 914, 823, 733, 706 cm^{-1}

All the spectroscopic data were matched with the previously reported data[13)].

2-(4-Fluorophenyl)-3-methylpent-4-en-2-ol (8d)

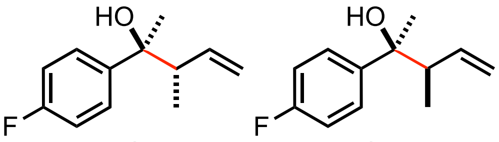

major *minor*

8d

The reaction was conducted using 1-(4-fluorophenyl)ethan-1-one (**7d**) (15.2 μL, 0.125 mmol, 1.0 equiv.) and liquid 2-butene (**2a**) (ca. 250 μL) for 6 hours. The residue was purified by silica gel flash column chromatography (Et_2O/hexane = 15%, v/v) to afford **8d** as a colorless oil (19.8 mg, 81%, dr = 1.1/1).

^{1}H NMR ($CDCl_3$, 500 MHz): diastereomeric mixture δ 7.41-7.34 (2H, m), 7.02-7.00 (2H, m), 5.79 (1H, ddd, J = 18.0, 9.5, 7.7 Hz, *major*), 5.69 (1H, ddd, J = 10.0, 4.6, 2.3 Hz, *minor*), 5.12-5.09 (2H, m), 2.54-2.51 (1H, m), 1.96 (1H, s, *major*), 1.84 (1H, s, *minor*), 1.52 (3H, s), 0.95 (3H, d, J = 6.9 Hz, *major*), 0.86 (3H, d, J = 6.9 Hz, *minor*); ^{13}C NMR ($CDCl_3$, 126 MHz): diastereomeric mixture δ 161.8 (d, J = 246.4 Hz), 161.7 (d, J = 245.2 Hz), 142.8 (d, J = 3.6 Hz), 142.8 (d, J = 3.6 Hz), 139.9, 139.7, 127.2 (d, J = 38.5 Hz), 127.2 (d, J = 39.7 Hz), 117.1, 116.8, 114.8, 114.6, 75.7, 75.6, 49.2, 49.1, 28.7, 26.0, 14.8, 14.4; ^{19}F NMR ($CDCl_3$, 369 MHz): diastereomeric mixture δ -116.3, -116.6 ; m/z calcd for $C_{12}H_{15}FO_5Na$ $[M+Na]^+$ 217.1005. Found 217.1022; IR (neat): 3493, 3080, 2973, 2253, 1642, 1595, 1508, 1452, 1411, 1375, 1271, 1221, 1164, 1081, 1014, 917, 833, 733 cm^{-1}

2-(4-Chlorophenyl)-3-methylpent-4-en-2-ol (8e)

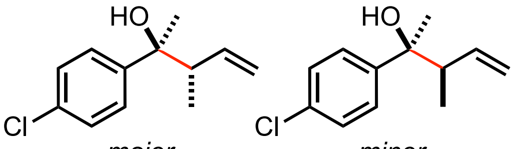

major *minor*

8e

The reaction was conducted using 1-(4-chlorophenyl)ethan-1-one (**7e**) (16.2 μL, 0.125 mmol, 1.0 equiv.) and liquid 2-butene (**2a**) (ca. 250 μL) for 6 hours. The residue was purified by silica gel flash column chromatography (Et_2O/hexane = 15%, v/v) to afford **8e** as a colorless oil (23.4 mg, 89%, dr = 1.2/1).

^{1}H NMR ($CDCl_3$, 500 MHz): diastereomeric mixture δ 7.34-7.23 (4H, m), 5.77 (1H, ddd, *J* = 18.0, 9.7, 7.4 Hz, *minor*), 5.68-5.61 (1H, m, *major*), 5.11-5.07 (2H, m), 2.53-2.47 (1H, m), 1.94 (1H, s, *major*), 1.83 (1H, s, *minor*), 1.48 (3H, s), 0.93 (3H, d, *J* = 6.9 Hz, *major*), 0.83 (3H, d, *J* = 6.9 Hz, *minor*); ^{13}C NMR ($CDCl_3$, 126 MHz): diastereomeric mixture δ 145.7, 139.7, 139.6, 132.6, 128.1, 127.2, 126.9, 117.2, 116.9, 75.7, 75.6, 49.0, 49.0, 28.7, 26.1, 14.8, 14.2; m/z calcd for $C_{12}H_{15}ClO_5Na$ [M+Na]$^{+\cdot}$ 233.0709. Found 233.0733; IR (neat): 3600, 2986, 2260, 1639, 1488, 1458, 1395, 1375, 1271, 1094, 1017, 917, 843, 733, 700 cm^{-1}

All the spectroscopic data were matched with the previously reported data[14)].

2-(4-Bromophenyl)-3-methylpent-4-en-2-ol (8f)

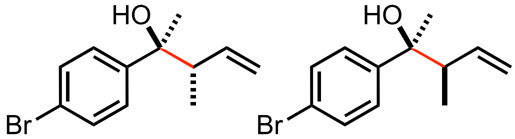

major *minor*

8f

The reaction was conducted using 1-(4-bromophenyl)ethan-1-one (**7f**) (15.7 μL, 0.125 mmol, 1.0 equiv.) and liquid 2-butene (**2a**) (ca. 250 μL) for 6 hours. The residue was purified by silica gel flash column chromatography (Et_2O/hexane = 15%, v/v) to afford **8f** as a colorless oil (28.9 mg, 91%, dr = 1.2/1).

^{1}H NMR ($CDCl_3$, 500 MHz): diastereomeric mixture δ 7.42 (2H, d, *J* = 8.6 Hz), 7.28-7.23 (2H, m), 5.76 (1H, ddd, *J* = 18.0, 9.5, 7.7 Hz, *minor*), 5.68-5.61 (1H, m, *major*), 5.12-5.06 (2H, m), 2.52-2.47 (1H, m), 1.93 (1H, s, *major*), 1.81 (1H, s, *minor*), 1.48 (3H, s) , 0.93 (3H, d, *J* = 6.9 Hz, *major*), 0.82 (3H, d, *J* = 6.9 Hz, *minor*) ; ^{13}C NMR ($CDCl_3$, 126 MHz): diastereomeric mixture δ 146.2, 139.7, 139.5, 131.1, 127.6, 127.3, 120.8, 120.5, 117.2, 116.9, 75.7, 75.6, 48.9, 48.9, 28.7, 26.1, 14.8, 14.2; m/z calcd for $C_{12}H_{15}BrONa$ [M+Na]$^+$ 277.0204. Found 277.0208; IR (neat): 3600, 2973, 2920, 1639, 1599, 1485, 1452, 1395, 1375, 1084, 1004, 927, 837, 740 cm^{-1}

All the spectroscopic data were matched with the previously reported data[15)].

2-(4-Iodophenyl)-3-methylpent-4-en-2-ol (8g)

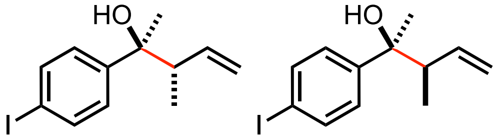

major *minor*

8g

The reaction was conducted using 1-(4-iodophenyl)ethan-1-one (**7g**) (30.8 mg, 0.125 mmol, 1.0 equiv.) and liquid 2-butene (**2a**) (ca. 250 μL) for 6 hours. The residue was purified by silica gel flash column chromatography (Et_2O/hexane = 15%, v/v) to afford **8g** as a colorless oil (28.6 mg, 76%, dr = 1.2/1).

^{1}H NMR ($CDCl_3$, 500 MHz): diastereomeric mixture 7.65 (2H, d, *J* = 8.0 Hz), 7.17-7.15 (2H, m), 5.79 (1H, ddd, *J* = 17.8, 9.7, 7.4 Hz, *minor*), 5.67-5.65 (1H, m, *major*), 5.14-5.08 (2H, m), 2.55-2.49 (1H, m), 1.94 (1H, s, *major*), 1.82 (1H, s, *minor*), 1.49 (3H, s), 0.96 (3H, d, *J* = 6.9 Hz, *major*), 0.85 (3H, d, *J* = 6.9 Hz, *minor*); ^{13}C

NMR ($CDCl_3$, 126 MHz): diastereomeric mixture δ 139.6, 139.5, 137.1, 127.9, 127.6, 117.2, 116.9, 92.4, 76.9, 75.8, 75.7, 48.9, 28.6, 26.0, 14.8, 14.2; m/z calcd for $C_{12}H_{15}IOlNa$ $[M+Na]^+$ 325.0065. Found 325.0058; IR (neat): 3600, 2973, 2940, 1485, 1391, 1371, 1077, 1004, 927, 827, 740 cm^{-1}

3-Methyl-2-(4-(trifluoromethyl)phenyl)pent-4-en-2-ol (8h)

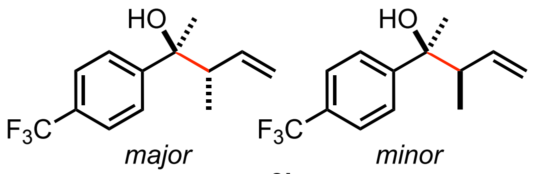

8h

The reaction was conducted using 1-(4-(trifluoromethyl)phenyl)ethan-1-one (**7h**) (23.5 mg, 0.125 mmol, 1.0 equiv.) and liquid 2-butene (**2a**) (ca. 250 μL) for 6 hours. The residue was purified by silica gel flash column chromatography (Et_2O/hexane = 20% to 25%, v/v) to afford **8h** as a colorless oil (30.5 mg, 100%, dr = 1.5/1).

major: ^{1}H NMR ($CDCl_3$, 500 MHz): δ 7.59 (2H, d, *J* = 8.0 Hz), 7.55 (2H, d, *J* = 8.6 Hz), 5.69-5.62 (1H, m), 5.12-5.10 (2H, m), 2.62-2.56 (1H, m), 2.00 (1H, s), 1.54 (3H, s), 0.99 (3H, d, *J* = 6.9 Hz); ^{13}C NMR ($CDCl_3$, 126 MHz): δ 151.2, 139.4, 129.0 (q, *J* = 125.2 Hz), 126.1, 125.0 (q, *J* = 10.8 Hz), 123.3, 117.5, 75.8, 48.9, 26.2, 14.1; ^{19}F NMR ($CDCl_3$, 369 MHz): δ -61.9; m/z calcd for $C_{13}H_{15}F_3ONa$ $[M+Na]^+$ 267.0973. Found 267.0974; IR (neat): 4460, 4200, 3053, 2980, 2300, 1615, 1415, 1328, 1264, 1168, 1121, 1067, 1017, 927, 894, 847, 733, 710 cm^{-1}

minor: ^{1}H NMR ($CDCl_3$, 500 MHz): 7.59 (2H, d, *J* = 8.0 Hz), 7.53 (2H, d, *J* = 8.0 Hz), 5.82 (1H, ddd, *J* = 18.0, 9.7, 7.4 Hz), 5.16-5.13 (2H, m), 2.58-2.52 (1H, m), 1.87 (1H, s), 1.54 (3H, s), 0.84 (3H, d, *J* = 6.9 Hz); ^{13}C NMR ($CDCl_3$, 126 MHz): δ 151.1, 139.3, 128.9 (q, *J* = 98.6 Hz), 125.8, 125.0 (q, *J* = 12.0 Hz), 123.3, 117.2, 75.9, 48.9, 28.9, 14.8; ^{19}F NMR ($CDCl_3$, 369 MHz): δ -61.8; m/z calcd for $C_{13}H_{15}F_3ONa$ $[M+Na]^+$ 267.0973. Found 267.0980; IR (neat): 4467, 3606, 3053, 2980, 1619, 1415, 1375, 1328, 1261, 1164, 1121, 1067, 1014, 920, 894, 850, 733, 706 cm^{-1}

All the spectroscopic data were matched with the previously reported data[16)].

4-(2-Hydroxy-3-methylpent-4-en-2-yl)benzonitrile (8i)

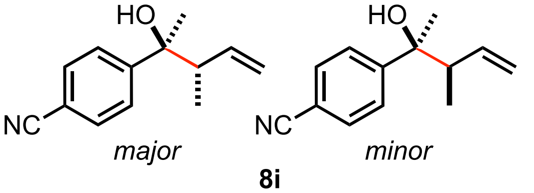

8i

The reaction was conducted using 4-acetylbenzonitrile (**7i**) (18.1 mg, 0.125 mmol, 1.0 equiv.) and liquid 2-butene (**2a**) (ca. 250 μL) for 6 hours. The residue was purified by silica gel flash column chromatography (Et_2O/hexane = 15% to 30%, v/v) to afford **8i** as a yellow oil (24.1 mg, 96%, dr = 1.5/1).

^{1}H NMR ($CDCl_3$, 392 MHz): diastereomeric mixture δ 7.61 (2H, d, *J* = 8.5 Hz), 7.55-7.51 (2H, m), 5.80 (1H, ddd, *J* = 18.0, 9.6, 7.6 Hz, *minor*), 5.61 (1H, ddd, *J* = 17.7, 10.1, 7.0 Hz, *major*), 5.17-5.05 (2H, m), 2.55-2.50 (1H, m), 2.08 (1H, br s, *major*), 1.96 (1H, br s, *minor*), 1.53 (3H, s, *major*), 1.52 (3H, s, *minor*), 0.98 (3H, d, *J* = 6.7 Hz, *major*), 0.82 (3H, d, *J* = 6.7 Hz, *minor*); ^{13}C NMR ($CDCl_3$, 99 MHz): diastereomeric mixture δ 152.6, 139.1, 139.0, 131.9, 131.9, 126.5, 126.3, 119.1, 117.6, 117.4, 110.6, 75.9, 75.8, 48.9, 48.7, 28.7, 26.2, 14.7, 14.1; m/z calcd for $C_{13}H_{15}NONa$ $[M+Na]^+$ 224.1051. Found 224.1051; IR (neat): 3620, 3073, 2980, 2226, 1635, 1605, 1505, 1452, 1405, 1371, 1341, 1268, 1144, 1077, 1017, 924, 850, 733, 703 cm^{-1}

All the spectroscopic data were matched with the previously reported data[17)].

Methyl 4-(2-hydroxy-3-methylpent-4-en-2-yl)benzoate (8j)

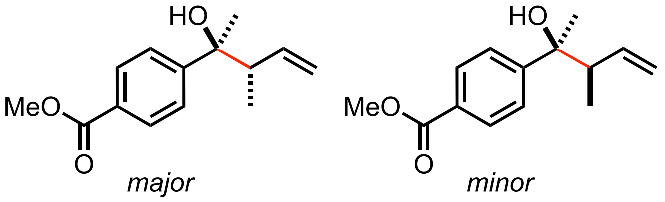

8j

The reaction was conducted using methyl 4-acetylbenzoate (**7j**) (22.3 mg, 0.125 mmol, 1.0 equiv.) and liquid 2-butene (**2a**) (ca. 250 μL) for 6 hours. The residue was purified by silica gel flash column chromatography (Et_2O/hexane = 20% to 30%, v/v) to afford **8j** as a yellow oil (29.3 mg, 100%, dr = 1.3/1).

^{1}H NMR ($CDCl_3$, 392 MHz): diastereomeric mixture δ 7.99 (2H, d, *J* = 8.5 Hz), 7.51-7.46 (2H, m), 5.81 (1H, ddd, *J* = 18.1, 9.5, 7.5 Hz, *minor*), 5.69-5.60 (1H, m, *major*), 5.15-5.06 (2H, m), 3.90 (3H, s), 2.62-2.51 (1H, m), 2.06 (1H, s, *major*), 1.96 (1H, s, *minor*), 1.54 (3H, s, *minor*), 1.53 (3H, s, *major*), 0.98 (3H, d, *J* = 6.7 Hz, *major*), 0.83 (3H, d, *J* = 6.7 Hz, *minor*) ; ^{13}C NMR ($CDCl_3$, 99 MHz): diastereomeric mixture δ 167.2, 152.5, 152.4, 139.5, 139.4, 129.4, 129.4, 128.6, 125.7, 125.4, 117.2, 117.0, 76.0, 75.9, 52.2, 48.9, 48.8, 28.7, 28.5, 26.2, 14.8, 14.1 ; m/z calcd for $C_{14}H_{18}O_3Na$ $[M+Na]^+$ 257.1154. Found 257.1154.; IR (neat): 4453, 4207, 3066, 2993, 2306, 1716, 1609, 1432, 1415, 1268, 1184, 1114, 1017, 894, 860, 730, 703 cm^{-1}

4-(2-Hydroxy-3-methylpent-4-en-2-yl)-*N*-methylbenzamide (8k)

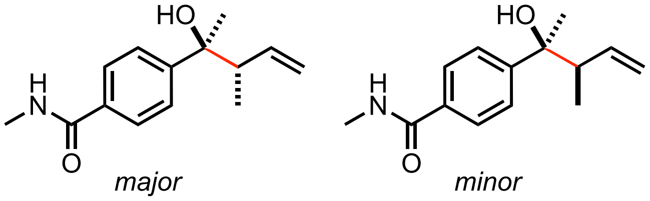

8k

The reaction was conducted using 4-acetyl-*N*-methylbenzamide (**7k**) (22.2 mg, 0.125 mmol, 1.0 equiv.) and liquid 2-butene (**2a**) (ca. 250 μL) for 12 hours. The residue was purified by silica gel flash column chromatography (MeOH/DCM = 2%, v/v) to afford **8k** as a white solid (25.2 mg, 86%, dr = 1.3/1).

^{1}H NMR ($CDCl_3$, 500 MHz): diastereomeric mixture δ 7.71 (2H, d, *J* = 8.0 Hz), 7.46-7.44 (2H, m), 6.28 (1H, br s), 5.80 (1H, ddd, *J* = 17.8, 9.7, 7.4 Hz, *minor*), 5.66-5.64 (1H, m, *major*), 5.13-5.06 (2H, m), 2.99 (3H, d, *J* = 4.6 Hz), 2.57-2.52 (1H, m), 2.14 (1H, br s, *major*), 2.05 (1H, br s, *minor*), 1.52 (3H, s), 0.95 (3H, d, *J* = 6.9 Hz, *major*), 0.83 (3H, d, *J* = 6.9 Hz, *minor*); ^{13}C NMR ($CDCl_3$, 126 MHz): diastereomeric mixture δ 168.3, 150.7, 139.6, 139.5, 133.0, 126.6, 126.6, 125.9, 125.6, 117.2, 116.9, 75.9, 75.8, 48.9, 28.6, 26.9, 26.1, 14.8, 14.1; m/z calcd for $C_{14}H_{19}NO_2Na$ $[M+Na]^+$ 256.1313. Found 256.1318; IR (neat): 3466, 3053, 2980, 2300, 1659, 1535, 1495, 1418, 1375, 1264, 1158, 1081, 1007, 924, 897, 854, 733, 696 cm^{-1}

3-Methyl-2-(4-(4,4,5,5-tetramethyl-1,3,2-dioxaborolan-2-yl)phenyl)pent-4-en-2-ol (8l)

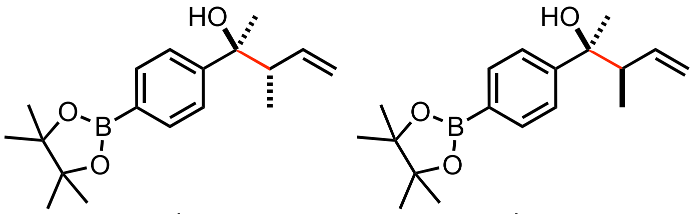

major *minor*

8l

The reaction was conducted using 1-(4-(4,4,5,5-tetramethyl-1,3,2-dioxaborolan-2-yl)phenyl)ethan-1-one (**7l**) (30.8 mg, 0.125 mmol, 1.0 equiv.) and liquid 2-butene (**2a**) (ca. 250 μL) for 6 hours. The residue was purified by silica gel flash column chromatography (Et_2O/hexane = 20% to 25%, v/v) to afford **8l** as a white solid (32.5 mg, 86%, dr = 1.2/1).

^1H NMR ($CDCl_3$, 500 MHz): diastereomeric mixture δ 7.79 (2H, d, *J* = 8.0 Hz), 7.43-7.41 (2H, m), 5.82 (1H, ddd, *J* = 17.8, 9.7, 7.4 Hz, *minor*), 5.71-5.64 (1H, m, *major*), 5.12-5.08 (2H, m), 2.60-2.54 (1H, m), 1.97 (1H, s, *major*), 1.88 (1H, s, *major*), 1.52 (3H, s), 1.35 (12H, s), 0.98 (3H, d, *J* = 6.9 Hz, *major*), 0.84 (3H, d, *J* = 6.9 Hz, *minor*); ^{13}C NMR ($CDCl_3$, 125 MHz): diastereomeric mixture δ 150.5, 150.4, 139.9, 139.9, 134.6, 124.9, 124.7, 116.8, 116.6, 83.9, 76.1, 75.9, 48.9, 48.6, 28.7, 26.2, 25.0, 25.0, 14.9, 14.0; m/z calcd for $C_{18}H_{27}BO_3Na$ $[M+Na]^+$ 325.1951. Found 325.1951; IR (neat): 3593, 2993, 1612, 1515, 1458, 1401, 1361, 1321, 1264, 1211, 1144, 1094, 1021, 960, 924, 860, 827, 737, 706, 656 cm^{-1}

2-(4-(2-Hydroxy-3-methylpent-4-en-2-yl)phenyl)isoindoline-1,3-dione (8m)

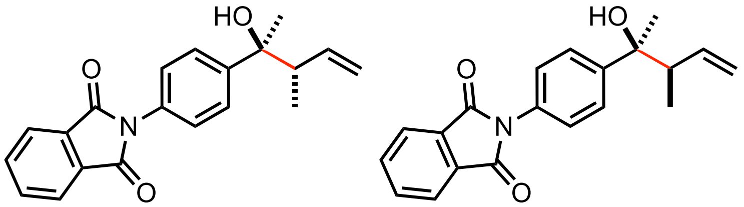

major *minor*

8m

The reaction was conducted using 2-(4-acetylphenyl)isoindoline-1,3-dione (**7m**) (33.2 mg, 0.125 mmol, 1.0 equiv.) and liquid 2-butene (**2a**) (ca. 250 μL) for 24 hours. 20 mol% of Cp_2Cr (0.025 mol) was used. The residue was purified by silica gel flash column chromatography (EtOAc/hexane = 33%, v/v) to afford **8m** as a white solid (24.5 mg, 61%, dr = 1.3/1).

^1H NMR ($CDCl_3$, 392 MHz): diastereomeric mixture δ 7.96 (2H, dd, *J* = 5.4, 3.1 Hz), 7.79 (2H, dd, *J* = 5.4, 3.1 Hz), 7.57-7.55 (2H, m), 7.42 (2H, d, *J* = 8.5 Hz), 5.85 (1H, ddd, *J* = 18.0, 9.6, 7.0 Hz, *minor*), 5.74 (1H, ddd, *J* = 17.5, 10.1, 7.0 Hz, *major*), 5.16-5.12 (2H, m), 2.65-2.57 (1H, m), 2.02 (1H, s, *major*), 1.91 (1H, s, *minor*), 1.55 (3H, s), 1.01 (3H, d, *J* = 6.7 Hz, *major*), 0.91 (3H, d, *J* = 7.2 Hz, *minor*); ^{13}C NMR ($CDCl_3$, 99 MHz): diastereomeric mixture δ 167.5, 147.2, 147.1, 139.8, 139.8, 134.5, 131.9, 130.3, 130.1, 126.5, 126.2, 126.0, 123.9, 117.1, 116.8, 75.9, 75.8, 48.9, 48.7, 28.9, 26.2, 15.0, 14.2; m/z calcd for $C_{20}H_{19}NO_3Na$ $[M+Na]^+$ 344.1263. Found 344.1262; IR (neat): 3533, 3080, 2980, 2926, 2880, 1789, 1742, 1709, 1665, 1612, 1508, 1462, 1378, 1218, 1171, 1121, 1087, 1017, 920, 887, 840, 797 cm^{-1}

3-Methyl-2-(4-(methylthio)phenyl)pent-4-en-2-ol (8n)

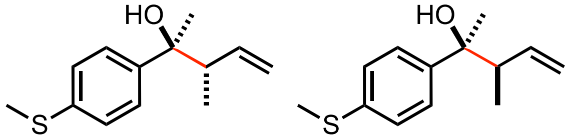

major *minor*

8n

The reaction was conducted using 1-(4-(methylthio)phenyl)ethan-1-one (**7n**) (20.8 mg, 0.125 mmol, 1.0 equiv.) and liquid 2-butene (**2a**) (ca. 250 μL) for 24 hours. 20 mol% of Cp_2Cr (0.025 mol) was used. The residue was purified by silica gel flash column chromatography

(Et_2O/hexane = 20%, v/v) to afford **8n** as a colorless oil (15.3 mg, 55%, dr = 1.4/1).

^{1}H NMR ($CDCl_3$, 500 MHz): diastereomeric mixture δ 7.39-7.33 (2H, m), 7.29-7.24 (2H, m), 5.82 (1H, ddd, *J* = 17.8, 9.7, 7.4 Hz, *major*), 5.76-5.69 (1H, m, *minor*), 5.14-5.12 (2H, m), 2.59-2.54 (1+3H, m), 1.95 (1H, s, *minor*), 1.85 (1H, s, *major*), 1.53 (3H, s), 0.98 (3H, d, *J* = 6.9 Hz, *minor*), 0.89 (3H, d, *J* = 6.9 Hz, *major*); ^{13}C NMR ($CDCl_3$, 125 MHz): diastereomeric mixture δ 144.1, 140.0, 139.9, 136,6 136.4, 126.4, 126.3, 126.3, 126.0, 116.9, 116.6, 75.7, 75.6, 49.0, 49.0, 28.6, 26.0, 16.1, 16.1, 14.9, 14.3; m/z calcd for $C_{13}H_{18}SONa$ [M+Na]$^+$ 245.0976. Found 245.0978; IR (neat): 3640, 3053, 2980, 2313, 1639, 1599, 1498, 1432, 1368, 1264, 1097, 1011, 927, 820, 733, 700 cm^{-1}

3-Methyl-2-(*m*-tolyl)pent-4-en-2-ol (8o)

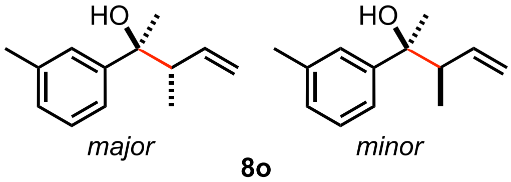

major **8o** *minor*

The reaction was conducted using 1-(*m*-tolyl)ethan-1-one (**7o**) (16.7 μL, 0.125 mmol, 1.0 equiv.) and liquid 2-butene (**2a**) (ca. 250 μL) for 24 hours. The residue was purified by silica gel flash column chromatography (Et_2O/hexane = 10%, v/v) to afford **8o** as a colorless oil (18.5 mg, 78%, dr = 1.2/1).

^{1}H NMR ($CDCl_3$, 500 MHz): diastereomeric mixture δ 7.29-7.21 (3H, m), 7.10 (1H, s, *major*), 7.08 (1H, s, *minor*), 5.86 (1H, ddd, *J* = 18.0, 9.6, 6.7 Hz, *minor*), 5.75 (1H, ddd, *J* = 17.5, 10.1, 7.0 Hz, *major*), 5.18-5.12 (2H, m), 2.68-2.54 (1H, m), 2.40 (3H, s), 1.98 (1H, s, *major*), 1.86 (1H, s, *minor*), 1.55 (3H, s), 1.00 (3H, d, *J* = 6.7 Hz, *major*), 0.90 (3H, d, *J* = 7.2 Hz, *minor*); ^{13}C NMR ($CDCl_3$, 99 MHz): diastereomeric mixture δ 147.2, 140.2, 140.1, 137.6, 127.9, 127.5, 127.3, 126.3, 126.0, 122.7, 116.8, 116.4, 75.9, 49.0, 48.8, 28.8, 26.0, 21.8, 14.9, 14.2; m/z calcd for $C_{13}H_{18}ONa$ [M+Na]$^+$ 213.1255. Found 213.1255; IR (neat): 4460, 4207, 3060, 2993, 2306, 1425, 1261, 897, 737, 703 cm^{-1}

3-Methyl-2-(*o*-tolyl)pent-4-en-2-ol (8p)

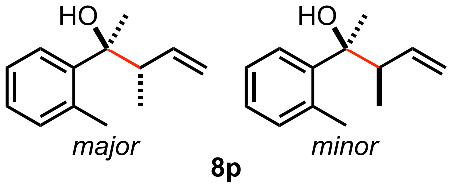

major **8p** *minor*

The reaction was conducted using 1-(*o*-tolyl)ethan-1-one (**7p**) (16.4 μL, 0.125 mmol, 1.0 equiv.) and liquid 2-butene (**2a**) (ca. 250 μL) for 24 hours. The residue was purified by silica gel flash column chromatography (DCM/hexane = 66%, v/v) to afford **8p** as a colorless oil (11.7 mg, 49%, dr = 2.8/1).

^{1}H NMR ($CDCl_3$, 500 MHz): diastereomeric mixture δ 7.48-7.34 (1H, m), 7.16-7.13 (3H, m), 5.92-5.79 (1H, m), 5.18-5.14 (2H, m, *major*), 5.09-5.07 (2H, m, *minor*), 2.95-2.91 (1H, m), 2.59 (3H, s, *major*), 2.55 (3H, s, *minor*), 1.98 (1H, s, *major*), 1.72 (1H, s, *minor*), 1.58 (3H, s), 0.94 (3H, d, *J* = 6.9 Hz, *major*), 0.92 (3H, d, *J* = 6.9 Hz, *minor*); ^{13}C NMR ($CDCl_3$, 126 MHz): diastereomeric mixture δ 144.4, 140.2, 140.1, 135.8, 132.9, 132.7, 127.2, 127.0, 126.8, 125.6, 125.4, 117.1, 116.1, 77.5, 77.3, 45.9, 45.6, 29.1, 27.7, 25.2, 22.9, 14.3; m/z calcd for $C_{13}H_{18}ONa$ [M+Na]$^+$ 213.1255. Found 213.1255; IR (neat): 4453, 3593, 3053, 2980, 2306, 1421, 1378, 1268, 894, 733, 703 cm^{-1}

2-(2-Chlorophenyl)-3-methylpent-4-en-2-ol (8q)

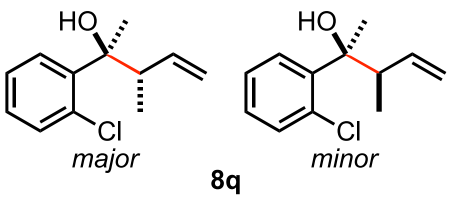

8q

The reaction was conducted using 1-(2-chlorophenyl)ethan-1-one (**7q**) (15.3 µL, 0.125 mmol, 1.0 equiv.) and liquid 2-butene (**2a**) (ca. 250 µL) for 6 hours. The residue was purified by silica gel flash column chromatography (Et_2O/hexane = 10%, v/v) to afford **8q** as a colorless oil (21.5 mg, 82%, dr = 2.0/1).

^{1}H NMR ($CDCl_3$, 500 MHz): diastereomeric mixture δ 7.65 (1H, d, *J* = 7.6 Hz, *minor*), 7.54 (1H, d, *J* = 7.6 Hz, *major*), 7.28-7.26 (1H, m), 7.17-7.11 (2H, m), 5.86 (1H, ddd, *J* = 17.6, 9.8, 6.8 Hz, *minor*), 5.63-5.54 (1H, m, *major*), 5.08-4.94 (2H, m), 3.43-3.36 (1H, m, *major*), 3.34-3.26 (1H, m, *minor*), 2.31 (1H, s, *major*), 2.09 (1H, s, *minor*), 1.60 (3H, s, *minor*), 1.59 (3H, s, *major*), 1.00 (3H, d, *J* = 6.7 Hz, *major*), 0.74 (3H, d, *J* = 6.7 Hz, *minor*); ^{13}C NMR ($CDCl_3$, 125 MHz): diastereomeric mixture δ 144.5, 139.7, 131.6, 128.6, 128.4, 128.3, 128.2, 126.8, 116.8, 116.3, 76.7, 44.0, 43.1, 26.3, 24.3, 14.4, 13.3; m/z calcd for $C_{12}H_{15}ClONa$ $[M+Na]^+$ 233.0709. Found 233.7014; IR (neat): 3060, 2980, 2306, 1428, 1264, 1151, 1081, 1047, 924, 894, 733, 706 cm^{-1}

2-(3-Bromophenyl)-3-methylpent-4-en-2-ol (8r)

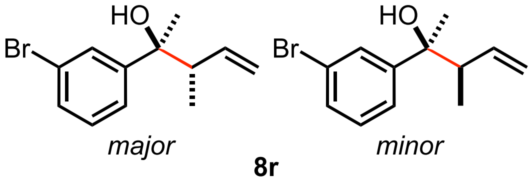

8r

The reaction was conducted using 1-(3-bromophenyl)ethan-1-one (**7r**) (16.6 µL, 0.125 mmol, 1.0 equiv.) and liquid 2-butene (**2a**) (ca. 250 µL) for 6 hours. The residue was purified by silica gel flash column chromatography (Et_2O/hexane = 10%, v/v) to afford **8r** as a colorless oil (23.3 mg, 73%, dr = 1.2/1).

^{1}H NMR ($CDCl_3$, 500 MHz): diastereomeric mixture δ 7.61-7.60 (1H, m , *major*), 7.58 (1H, m, *minor*), 7.36-7.32 (2H, m), 7.21-7.19 (1H, m), 5.81 (1H, ddd, *J* = 17.9, 9.6, 7.6 Hz, *minor*), 5.71-5.64 (1H, m, *major*), 5.16-5.09 (2H, m), 2.59-2.48 (1H, m), 1.98 (1H, s, *major*), 1.85 (1H, s, *minor*), 1.50 (3H, s), 0.98 (3H, d, *J* = 6.9 Hz, *major*), 0.85 (3H, d, *J* = 6.9 Hz, *minor*); ^{13}C NMR ($CDCl_3$, 126 MHz): diastereomeric mixture δ 149.6, 139.5, 139.5, 129.9, 129.7, 129.6, 129.6, 128.9, 128.7, 124.4, 124.1, 122.5, 117.3, 117.0, 75.7, 75.6, 48.9, 48.8, 28.8, 26.1, 14.8, 14.1; m/z calcd for $C_{12}H_{15}BrONa$ $[M+Na]^+$ 277.0204. Found 277.0204; IR (neat): 3606, 3053, 2986, 2306, 1428, 1268, 1004, 900, 733, 706 cm^{-1}

2-(2-Bromophenyl)-3-methylpent-4-en-2-ol (8s)

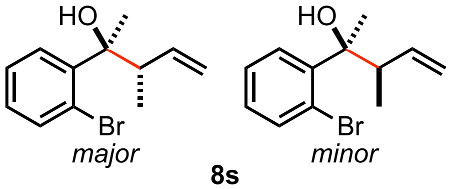

8s

The reaction was conducted using 1-(2-bromophenyl)ethan-1-one (**7s**) (16.8 µL, 0.125 mmol, 1.0 equiv.) and liquid 2-butene (**2a**) (ca. 250 µL) for 6 hours. The residue was purified by silica gel flash column chromatography (Et_2O/hexane = 10%, v/v) to afford **8s** as a colorless oil (28.7 mg, 90%, dr = 2.7/1).

^{1}H NMR ($CDCl_3$, 500 MHz): diastereomeric mixture δ 7.74-7.72 (1H, m, *minor*), 7.63-7.61 (1H, m, *major*), 7.59-7.57 (1H, m), 7.31-7.26 (1H, m), 7.09 (1H, m), 5.92 (1H, ddd, *J* = 17.6, 10.2, 7.0 Hz, *minor*), 5.72-5.65 (1H, m, *major*), 5.14-5.04 (2H, m), 3.61-3.56 (1H, m, *major*), 3.52-3.47 (1H, m, *minor*), 2.47 (1H, s, *major*), 2.23 (1H, s, *minor*), 1.70 (3H, s, *minor*), 1.68 (3H, s, *major*), 1.06 (3H, d, *J* = 6.9 Hz, *major*), 0.83 (3H, d, *J* = 6.9 Hz, *minor*); ^{13}C NMR ($CDCl_3$, 126 MHz): diastereomeric mixture δ 145.7, 145.2, 139.7, 139.6, 135.4, 135.2,

128.9, 128.7, 128.6, 128.5, 127.3, 120.1, 116.8, 116.5, 77.1, 43.7, 43.0, 26.1, 24.1, 14.2, 13.4; m/z calcd for $C_{12}H_{15}BrONa$ $[M+Na]^+$ 277.0204. Found 227.0203; IR (neat): 3493, 2980, 1462, 1425, 1151, 1017, 920, 884, 767, 727 cm^{-1}

1,1,1-Trifluoro-3-methyl-2-phenylpent-4-en-2-ol (8t)

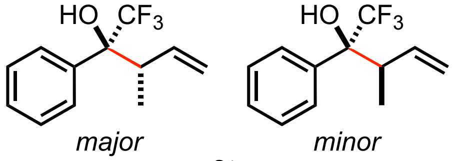

8t

The reaction was conducted using 2,2,2-trifluoro-1-phenylethan-1-one (**7t**) (17.3 μL, 0.125 mmol, 1.0 equiv.) and liquid 2-butene (**2a**) (ca. 250 μL) for 6 hours. The residue was purified by silica gel flash column chromatography (Et_2O/hexane = 15%, v/v) to afford **8t** as a colorless oil (20.0 mg, 70%, dr = 2.0/1).

^1H NMR ($CDCl_3$, 500 MHz): diastereomeric mixture δ 7.56-7.55 (2H, m), 7.42-7.33 (3H, m), 6.08 (1H, ddd, *J* = 16.3, 4.8, 2.4 Hz, *major*), 5.56 (1H, ddd, *J* = 18.9, 8.6, 4.3 Hz, *minor*), 5.34-5.27 (2H, m, *major*), 5.07-5.04 (2H, m, *minor*), 3.15-3.04 (1H, m), 2.58 (1H, s, *major*), 2.53 (1H, s, *minor*), 1.20 (3H, d, *J* = 6.9 Hz, *minor*), 0.80 (3H, d, *J* = 6.9 Hz, *major*); ^{13}C NMR ($CDCl_3$, 99 MHz): diastereomeric mixture δ 137.3 (q, *J* = 51.0 Hz), 128.4, 128.4, 126.1, 125.8, 118.3, 117.9, 78.8 (q, *J* = 78.4 Hz), 44.1, 42.2, 14.0, 13.7; ^{19}F NMR ($CDCl_3$, 369 MHz): diastereomeric mixture δ -72.6, -73.3; m/z calcd for $C_{12}H_{13}F_3ONa$ $[M+Na]^+$ 253.0816. Found 253.0829; IR (neat): 3433, 1726, 1452, 1268, 1154, 1074, 1031, 1001, 970, 927, 910, 767, 713 cm^{-1}

All the spectroscopic data were matched with the previously reported data[18)].

2-(Benzo[*b*]thiophen-2-yl)-3-methylpent-4-en-2-ol (8u)

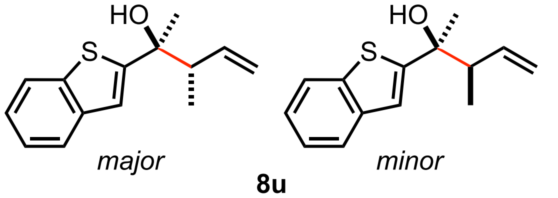

8u

The reaction was conducted using 1-(benzo[*b*]thiophen-2-yl)ethan-1-one (**7u**) (22.0 mg, 0.125 mmol, 1.0 equiv.) and liquid 2-butene (**2a**) (ca. 250 μL) for 12 hours. The residue was purified by silica gel flash column chromatography (EtOAc/hexane = 30%, v/v) to afford **8u** as a colorless oil (18.7 mg, 60%, dr = 2.2/1).

^1H NMR ($CDCl_3$, 392 MHz): diastereomeric mixture δ 7.81-7.79 (1H, m), 7.72-7.70 (1H, m), 7.33-7.29 (2H, m), 7.14 (1H, s, *major*), 7.13 (1H, s, *minor*), 5.93-5.78 (1H, m), 5.24-5.13 (2H, m), 2.69-2.63 (1H, m), 2.34 (1H, s, *major*), 2.26 (1H, s, *minor*), 1.65 (3H, s, *minor*), 1.64 (3H, s, *major*), 1.08 (3H, d, *J* = 7.2 Hz, *minor*), 1.05 (3H, d, *J* = 6.7 Hz, *major*); ^{13}C NMR ($CDCl_3$, 126 MHz): diastereomeric mixture δ 153.2, 139.5, 139.3, 124.3, 124.0, 123.9, 123.4, 122.4, 122.3, 119.9, 119.5, 117.8, 117.7, 75.6, 49.8, 49.4, 28.9, 26.3, 15.1, 14.6; m/z calcd for $C_{14}H_{16}OSNa$ $[M+Na]^+$ 255.0820. Found 255.0820; IR (neat): 3480, 2986, 2926, 2880, 2360, 1639, 1462, 1435, 1368, 1311, 1251, 1181, 1134, 1027, 1004, 927, 864, 837, 750, 730 cm^{-1}

4-Methyl-3-phenylhex-5-en-3-ol (8v)

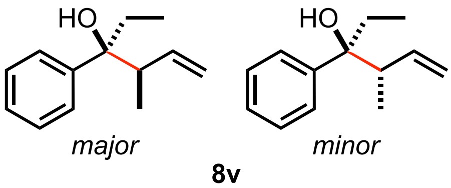

8v

The reaction was conducted using propiophenone (**7v**) (16.6 μL, 0.125 mmol, 1.0 equiv.) and liquid 2-butene (**2a**) (ca. 250 μL) for 12 hours. The residue was purified by silica gel flash column chromatography (EtOAc/hexane = 8%, v/v) to afford **8v** as a colorless oil (19.0 mg, 80%, dr = 1.3/1).

^{1}H NMR ($CDCl_3$, 500 MHz): diastereomeric mixture δ 7.38-7.32 (4H, m), 7.24-7.22 (1H, m), 5.88-5.81 (1H, m, *major*), 5.66-5.59 (1H, m, *minor*), 5.13-5.05 (2H, m), 2.68-2.62 (1H, m, *minor*), 2.60-2.55 (1H, m, *major*), 2.01-1.82 (2H, m), 1.80 (1H, s), 1.03 (3H, d, *J* = 6.9 Hz, *minor*), 0.82 (3H, d, *J* = 6.9 Hz, *major*), 0.73 (3H, t, *J* = 7.4 Hz, *minor*), 0.68 (3H, t, *J* = 7.2 Hz, *major*); ^{13}C NMR ($CDCl_3$, 126 MHz): diastereomeric mixture δ 144.8, 144.5, 140.2, 128.0, 127.9, 126.5, 126.4, 126.3, 126.0, 116.5, 116.4, 78.5, 78.3, 48.5, 47.8, 33.5, 31.5, 15.1, 13.5, 7.9, 7.9; m/z calcd for $C_{13}H_{18}ONa$ $[M+Na]^+$ 213.1255. Found 213.1255; IR (neat): 3520, 2986, 2926, 2886, 1639, 1602, 1495, 1448, 1254, 1164, 1054, 967, 914, 763, 706, 680 cm^{-1}
All the spectroscopic data were matched with the previously reported data[11)].

3-Methyl-4-phenylhept-1-en-4-ol (8w)

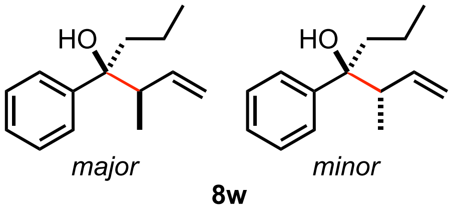

8w

The reaction was conducted using 1-phenylbutan-1-one (**7w**) (18.3 μL, 0.125 mmol, 1.0 equiv.) and liquid 2-butene (**2a**) (ca. 250 μL) for 12 hours. The residue was purified by silica gel flash column chromatography (EtOAc/hexane = 5%, v/v) to afford **8w** as a colorless oil (21.7 mg, 85%, dr = 1.6/1).

^{1}H NMR ($CDCl_3$, 500 MHz): diastereomeric mixture δ 7.38-7.31 (4H, m), 7.23-7.22 (1H, m), 5.86-5.84 (1H, m, *major*), 5.66-5.59 (1H, m, *minor*), 5.17-5.12 (2H, m, *major*), 5.07-5.02 (2H, m, *minor*), 2.67-2.62 (1H, m, *minor*), 2.58-2.55 (1H, m, *major*), 1.93-1.74 (2+1H, m), 1.34-1.19 (1H, m), 1.04-0.80 (7H, m); ^{13}C NMR ($CDCl_3$, 126 MHz): diastereomeric mixture δ 145.3, 145.0, 140.2, 140.1, 128.0, 127.9, 126.4, 126.3, 126.1, 125.8, 116.5, 116.5, 78.3, 78.1, 48.7, 48.0, 43.4, 41.4, 16.9, 16.9, 15.0, 14.6, 13.5; m/z calcd for $C_{14}H_{20}ONa$ $[M+Na]^+$ 227.1412. Found 227.1412; IR (neat): 3513, 2960, 1632, 1445, 1365, 1168, 1007, 910, 773, 703cm^{-1}
All the spectroscopic data were matched with the previously reported data[17)].

3-Methyl-4-phenylpentadec-1-en-4-ol (8x)

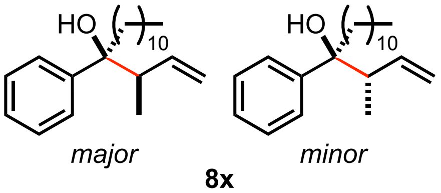

8x

The reaction was conducted using 1-phenyldodecan-1-one (**7x**) (36 μL, 0.125 mmol, 1.0 equiv.) and liquid 2-butene (**2a**) (ca. 250 μL) for 12 hours. The residue was purified by silica gel flash column chromatography (DCM/hexane = 30%, v/v) to afford **8x** as a yellow oil (34.2 mg, 87%, dr = 1.5/1).

^{1}H NMR ($CDCl_3$, 392 MHz): diastereomeric mixture δ 7.39-7.31 (4H, m), 7.24-7.22 (1H, m), 5.86-5.83 (1H, m, *major*), 5.64-5.61 (1H, m, *minor*), 5.17-5.12 (2H, m, *major*), 5.07-5.02 (2H, m, *minor*), 2.65 (1H, m, *minor*), 2.60-2.52 (1H, m, *major*), 1.97-1.74 (2+1H, m), 1.29-1.18 (18H, m), 1.03 (3H, d, *J* = 6.7 Hz, *minor*), 0.88 (3H,

t, J = 7.0 Hz), 0.81 (3H, d, J = 7.2 Hz, *major*); ^{13}C NMR ($CDCl_3$, 99 MHz): diastereomeric mixture δ 145.2, 144.9, 140.2, 140.1, 127.9, 127.9, 126.4, 126.3, 126.1, 125.9, 116.5, 116.4, 78.3, 78.1, 48.8, 48.0, 41.0, 39.0, 32.0, 30.5, 30.3, 30.2, 29.7, 29.7, 29.5, 23.6, 23.5, 22.8, 15.1, 14.3, 13.5; m/z calcd for $C_{22}H_{36}ONa$ $[M+Na]^+$ 339.2664. Found 339.2654; IR (neat): 3606, 3060, 2926, 2853, 1635, 1602, 1495, 1445, 1378, 1261, 1007, 920, 737, 706 cm^{-1}

3,6-Dimethyl-4-phenylhept-1-en-4-ol (8y)

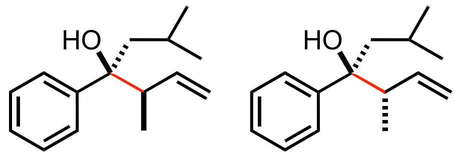

major *minor*

8y

The reaction was conducted using 3-methyl-1-phenylbutan-1-one (**7y**) (20.9 μL, 0.125 mmol, 1.0 equiv.) and liquid 2-butene (**2a**) (ca. 250 μL) for 12 hours. The residue was purified by silica gel flash column chromatography (Et_2O/hexane = 8%, v/v) to afford **8y** as a colorless oil (16.7 mg, 61%, dr = 2.2/1).

^{1}H NMR ($CDCl_3$, 500 MHz): diastereomeric mixture δ 7.38-7.30 (4H, m), 7.24-7.20 (1H, m), 5.86 (1H, ddd, J = 18.0, 9.4, 7.4 Hz, *major*), 5.60-5.51 (1H, m, *minor*), 5.17-5.11 (2H, m, *major*), 5.04-5.00 (2H, m, *minor*), 2.58-2.51 (1H, m), 1.89-1.81 (2H, m), 1.71-1.61 (1H, m), 1.44-1.41 (1H, m), 1.05 (3H, d, J = 6.7 Hz, *minor*), 0.89-0.88 (3H, m), 0.75 (3H, d, J = 7.2 Hz, *major*), 0.70 (3H, d, J = 6.7 Hz, *minor*), 0.64 (3H, d, J = 6.7 Hz, *major*); ^{13}C NMR ($CDCl_3$, 99 MHz): diastereomeric mixture δ 145.6, 145.1, 140.2, 140.1, 127.9, 126.3, 126.2, 126.0, 125.9, 116.7, 116.5, 93.2, 78.8, 78.6, 49.9, 49.8, 49.1, 47.7, 25.0, 24.8, 24.5, 24.2, 24.1, 14.9, 13.2; m/z calcd for $C_{15}H_{22}ONa$ $[M+Na]^+$ 241.1586. Found 241.1586; IR (neat): 3620, 3060, 2980, 2886, 2320, 1642, 1452, 1418, 1365, 1158, 997, 920, 887, 700 cm^{-1}

1-(But-3-en-2-yl)cyclohexan-1-ol (8z)

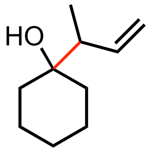

8z

The reaction was conducted using cyclohexanone (**7z**) (13.0 μL, 0.125 mmol, 1.0 equiv.) and liquid 2-butene (**2a**) (ca. 250 μL) for 12 hours. The residue was purified by silica gel flash column chromatography (Et_2O/pentane = 10% to 15%, v/v) to afford **8z** as a colorless oil (13.6 mg, 71%).

^{1}H NMR ($CDCl_3$, 500 MHz): δ 5.83 (1H, ddd, J = 18.2, 9.3, 7.3 Hz), 5.08-5.07 (1H, m), 5.06-5.04 (1H, m), 2.20-2.14 (1H, m), 1.63-1.38 (10H, m), 1.27 (1H, s), 1.02 (3H, d, J = 6.9 Hz); ^{13}C NMR ($CDCl_3$, 126 MHz): δ 140.6, 116.0, 72.5, 48.5, 35.1, 34.5, 26.0, 22.0, 14.3; m/z calcd for $C_{10}H_{18}ONa$ $[M+Na]^+$ 177.1255. Found 177.1256; IR (neat): 3468, 2940, 2860, 1462, 1171, 960, 947 cm^{-1}

All the spectroscopic data were matched with the previously reported data[19)].

1-(But-3-en-2-yl)cycloheptan-1-ol (8aa)

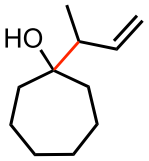

8aa

The reaction was conducted using cycloheptanone (**7aa**) (14.7 μL, 0.125 mmol, 1.0 equiv.) and liquid 2-butene (**2a**) (ca. 250 μL) for 12 hours. The residue was purified by silica gel flash column chromatography (Et_2O/hexane = 12%, v/v) to afford **8aa** as a colorless oil (14.9 mg, 71%).

^{1}H NMR ($CDCl_3$, 500 MHz): δ 5.84-5.82 (1H, m), 5.08 (1H, m), 5.06-5.05 (1H, m), 2.24-2.18

(1H, m), 1.76-1.39 (12H, m), 1.32 (1H, s), 1.03 (3H, d, *J* = 7.4 Hz); ^{13}C NMR ($CDCl_3$, 126 MHz): δ 140.9, 116.0, 49.7, 39.3, 38.4, 29.8, 29.8, 22.8, 22.6, 14.8; m/z calcd for $C_{11}H_{20}ONa$ $[M+Na]^+$ 191.1412. Found 191.1412; IR (neat): 3453, 3080, 2926, 2853, 1642, 1515, 1455, 1378, 1281, 1184, 1121, 1001, 960, 910, 850, 790, 740 cm^{-1}

1-(But-3-en-2-yl)cyclopentadecan-1-ol (8ab)

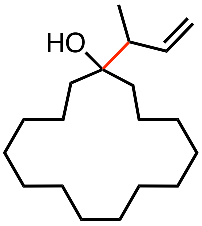

8ab

The reaction was conducted using ketone (**7ab**) (31.3 μL, 0.125 mmol, 1.0 equiv.) and liquid 2-butene (**2a**) (ca. 250 μL) for 12 hours. The residue was purified by silica gel flash column chromatography (EtOAc/hexane = 5%, v/v) to afford **8ab** as a colorless oil (10.8 mg, 30%).

^{1}H NMR ($CDCl_3$, 392 MHz): δ 5.89 (1H, ddd, *J* = 18.1, 9.5, 7.7 Hz), 5.06-5.03 (2H, m), 2.25-2.17 (1H, m), 1.53-1.26 (28H, m), 1.13 (1H, s), 1.01 (3H, d, *J* = 6.7 Hz); ^{13}C NMR ($CDCl_3$, 99 MHz): δ 140.8, 115.5, 75.6, 45.5, 37.6, 36.0, 28.0, 28.0, 27.0, 26.9, 26.8, 26.7, 26.1, 26.1, 22.0, 21.8, 14.2; m/z calcd for $C_{19}H_{36}ONa$ $[M+Na]^+$ 303.2664. Found 303.2662; IR (neat): 3620, 3053, 2940, 2853, 1635, 1462, 1351, 1158, 1001, 920 cm^{-1}

(1*r*,3*r*,5*r*,7*r*)-2-(But-3-en-2-yl)adamantan-2-ol (8ac)

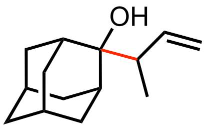

8ac

The reaction was conducted using (1*r*,3*r*,5*r*,7*r*)-adamantan-2-one (**7ac**) (18.8 mg, 0.125 mmol, 1.0 equiv.) and liquid 2-butene (**2a**) (ca. 250 μL) for 6 hours. The residue was purified by silica gel flash column chromatography (EtOAc/hexane = 4%, v/v) to afford **8ac** as a colorless oil (23.9 mg, 93%).

^{1}H NMR ($CDCl_3$, 500 MHz): δ 5.90 (1H, ddd, *J* = 18.0, 9.7, 7.4 Hz), 5.10-5.05 (2H, m), 2.92-2.86 (1H, m), 2.14-2.13 (2H, m), 1.94-1.51 (12H, m), 1.33 (1H, s), 0.97 (3H, d, *J* = 6.9 Hz); ^{13}C NMR ($CDCl_3$, 126 MHz): δ 140.3, 115.5, 75.5, 40.4, 38.5, 35.7, 34.0, 33.9, 33.8, 33.3, 27.2, 27.1, 12.7; m/z calcd for $C_{14}H_{22}ONa$ $[M+Na]^+$ 229.1568. Found 229.1568; IR (neat): 3633, 3046, 2986, 2926, 2866, 2313, 1629, 1462, 1418, 1134, 1101, 1057, 987, 930, 894 cm^{-1}

3,4-Dimethyldodec-1-en-4-ol (8ad)

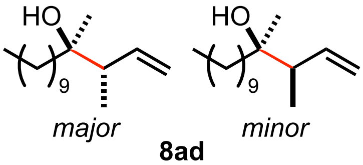

8ad

The reaction was conducted using decan-2-one (**7ad**) (28.0 μL, 0.125 mmol, 1.0 equiv.) and liquid 2-butene (**2a**) (ca. 250 μL) for 12 hours. The residue was purified by silica gel flash column chromatography (Et_2O/hexane = 15%, v/v) to afford **8ad** as a colorless oil (22.4 mg, 75%, dr = 1.2/1).

^{1}H NMR ($CDCl_3$, 500 MHz): diastereomeric mixture δ 5.88-5.76 (1H, m), 5.09-5.07 (2H, m), 2.27-2.24 (1H, m), 1.47-1.26 (18+1H, m), 1.11 (3H, s, *major*), 1.09 (3H, s, *minor*), 1.03 (3H, d, *J* = 6.9 Hz, *major*), 1.00 (3H, d, *J* = 6.9 Hz, *minor*), 0.88 (3H, t, *J* = 6.9 Hz); ^{13}C NMR ($CDCl_3$, 126 MHz): diastereomeric mixture δ 140.7, 140.6, 116.4, 116.0, 74.0, 73.9, 47.9, 47.3, 40.2, 39.7, 32.1, 30.5, 30.4, 29.8, 29.8, 29.5, 29.5, 24.3, 23.8, 23.5, 23.4, 22.8, 15.1, 14.7, 14.3; m/z calcd for $C_{16}H_{32}ONa$ $[M+Na]^+$ 263.2351. Found 263.2350; IR (neat): 3640, 3046, 2973, 2976, 2846, 1472, 1418, 1385, 930, 887 cm^{-1}

2-Cyclohexyl-3-methylpent-4-en-2-ol (8ae)

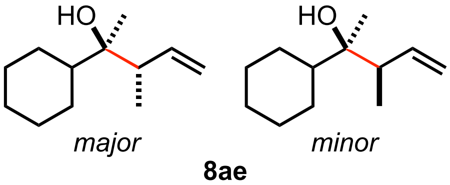

8ae

The reaction was conducted using 1-cyclohexylethan-1-one (**7ae**) (17.2 μL, 0.125 mmol, 1.0 equiv.) and liquid 2-butene (**2a**) (ca. 250 μL) for 24 hours. 20 mol% of Cp_2Cr (0.025 mol) was used. The residue was purified by silica gel flash column chromatography (DCM/hexane = 66% to 75%, v/v) to afford **8ae** as a colorless oil (13.7 mg, 60%, dr = 1.7/1).

^{1}H NMR ($CDCl_3$, 500 MHz): diastereomeric mixture δ 5.88 (1H, m), 5.11-5.01 (2H, m), 2.41-2.37 (1H, m), 1.83-1.68 (6H, m), 1.48-1.38 (1H, m), 1.24-0.99 (11H, m); ^{13}C NMR ($CDCl_3$, 126 MHz): diastereomeric mixture δ 141.2, 140.8, 116.0, 115.4, 75.4, 75.3, 45.1, 44.8, 44.6, 44.3, 27.7, 27.4, 27.0, 26.9, 26.8, 26.7, 26.6, 26.4, 21.0, 20.3, 14.8, 14.0; m/z calcd for $C_{12}H_{22}ONa$ $[M+Na]^+$ 205.1568. Found 205.1568; IR (neat): 3486, 3080, 2940, 2853, 2660, 1632, 1508, 1448, 1348, 1298, 1201, 1121, 1067, 990, 914, 847, 813 cm^{-1}

All the spectroscopic data were matched with the previously reported data[11)].

3-Methyl-4-propylhept-1-en-4-ol (8af)

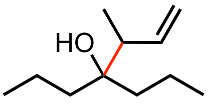

8af

The reaction was conducted using heptan-4-one (**7af**) (17.5 μL, 0.125 mmol, 1.0 equiv.) and liquid 2-butene (**2a**) (ca. 250 μL) for 24 hours. 20 mol% of Cp2Cr (0.025 mol) was used. The residue was purified by silica gel flash column chromatography (Et_2O/pentane = 12%, v/v) to afford **8af** as a colorless oil (13.7 mg, 64%).

^{1}H NMR ($CDCl_3$, 392 MHz): δ 5.91-5.82 (1H, m), 5.08-5.03 (2H, m), 2.34-2.27 (1H, m), 1.45-1.24 (9H, m), 1.00 (3H, d, *J* = 7.6 Hz), 0.91-0.90 (6H, m); ^{13}C NMR ($CDCl_3$, 99 MHz): δ 140.7, 115.9, 75.4, 45.3, 39.3, 38.8, 16.7, 16.6, 14.9, 14.9, 14.5; m/z calcd for $C_{11}H_{22}ONa$ $[M+Na]^+$ 193.1568. Found 193.1568; IR (neat): 3606, 2966, 2873, 1642, 1462, 1421, 1385, 1127, 957, 914 cm^{-1}

1-Isopropoxy-3,4-dimethylhex-5-en-3-ol (8ag)

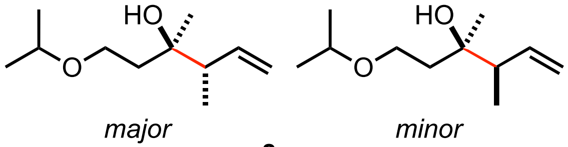

8ag

The reaction was conducted using 4-isopropoxybutan-2-one (**7ag**) (18.2 μL, 0.125 mmol, 1.0 equiv.) and liquid 2-butene (**2a**) (ca. 250 μL) for 12 hours. The residue was purified by silica gel flash column chromatography (Et_2O/hexane = 20%, v/v) to afford **8ag** as a colorless oil (10.9 mg, 47%, dr = 1.4/1).

^{1}H NMR ($CDCl_3$, 392 MHz): diastereomeric mixture δ 5.98-5.88 (1H, m, *major*), 5.79 (1H, ddd, *J* = 18.0, 9.6, 7.6 Hz, *minor*), 5.07-5.00 (2H, m), 3.73-3.55 (1+2+1H, m), 2.33-2.22 (1H, m), 1.92-1.76 (1H, m), 1.70-1.58 (1H, m), 1.17-1.14 (9H, m), 1.06 (3H, d, *J* = 6.7 Hz, *minor*), 1.02 (3H, d, *J* = 6.7 Hz, *major*); ^{13}C NMR ($CDCl_3$, 99 MHz): diastereomeric mixture δ 141.2, 140.8, 115.3, 73.9, 73.9, 72.4, 72.3, 65.3, 65.2, 48.4, 48.1, 38.2, 37.6, 23.7, 23.0, 22.2, 22.1, 14.8, 14.4; m/z calcd for $C_{11}H_{22}O_2Na$ $[M+Na]^+$ 209.1517. Found 209.1517; IR (neat): 3486, 3053, 2986, 2933, 2873, 2320, 1635, 1462, 1425, 1385, 1335, 1127, 994, 907, 793 cm^{-1}

tert-Butyl (4-(but-3-en-2-yl)-4-hydroxycyclohexyl)carbamate (8ah)

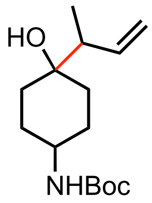

8ah

The reaction was conducted using *tert*-butyl (4-oxocyclohexyl)carbamate (**7ah**) (26.7 mg, 0.125 mmol, 1.0 equiv.) and liquid 2-butene (**2a**) (ca. 250 µL) for 12 hours. The residue was purified by silica gel flash column chromatography (EtOAc/hexane = 33%, v/v) to afford **8ah** as a white solid (26.8 mg, 80%, dr = 3.1/1).

^{1}H NMR ($CDCl_3$, 500 MHz): δ 5.83-5.77 (1H, m), 5.08-5.06 (2H, m), 4.43 (1H, br s), 3.68 (1H, br s, *minor*), 3.35 (1H, br s, *major*), 2.29-2.28 (1H, m, *minor*), 2.15-2.12 (1H, m, *major*), 1.79-1.26 (18H, m), 1.04-0.97 (3H, m); ^{13}C NMR ($CDCl_3$, 126 MHz): δ 140.1, 116.7, 71.4, 49.5, 49.0, 33.7, 32.9, 28.7, 28.6, 14.5, 14.0; m/z calcd for $C_{15}H_{27}NO_3Na$ $[M+Na]^+$ 292.1889. Found 292.1889; IR (neat): 3446, 3060, 2973, 2946, 1712, 1498, 1458, 1391, 1365, 1271, 1174, 1044, 1021, 957, 917, 880, 830, 737 cm^{-1}

(5*S*,8*R*,9*S*,10*S*,13*S*,14*S*,17*S*)-3-(But-3-en-2-yl)-10,13-dimethylhexadecahydro-1*H*-cyclopenta[*a*]phenanthrene-3,17-diol (8ai)

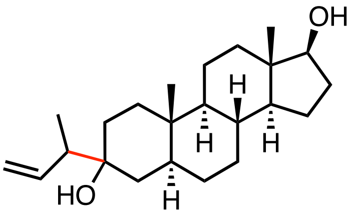

8ai

The reaction was conducted using stanolone (**7ai**) (36.3 mg, 0.125 mmol, 1.0 equiv.) and liquid 2-butene (**2a**) (ca. 250 µL) for 24 hours. The residue was purified by silica gel flash column chromatography (MeOH/DCM = 2%, v/v) to afford **8ai** as a white solid (25.6 mg, 59%, dr = 12/1).

^{1}H NMR ($CDCl_3$, 500 MHz): diastereomeric mixture δ 5.85-5.77 (1H, m), 5.07-5.03 (2H, m), 3.62 (1H, t, J = 8.6 Hz), 2.09-2.03 (2H, m), 1.80-0.72 (32H, m); ^{13}C NMR ($CDCl_3$, 126 MHz): diastereomeric mixture δ 140.5, 116.2, 82.1, 72.9, 54.4, 51.2, 49.6, 49.5, 43.1, 41.0, 40.9, 37.7, 37.4, 36.9, 35.9, 35.7, 34.0, 31.7, 30.9, 30.7, 30.5, 28.7, 23.5, 20.7, 14.3, 11.3; m/z calcd for $C_{23}H_{38}O_2Na$ $[M+Na]^+$ 369.2769. Found 369.2758; IR (neat): 3606, 3053, 2926, 2840, 1639, 1455, 1381, 1191, 1004, 897, 740 cm^{-1}

Note: In the ^{13}C NMR spectra additional signals were observed. These correspond to the pseudoenantiomer forms of the product. In the ^{1}H NMR spectra the signals could not be distinguished.

1-(5-Hydroxy-5,6-dimethyloct-7-en-1-yl)-3,7-dimethyl-3,7-dihydro-1H-purine-2,6-dione (8aj)

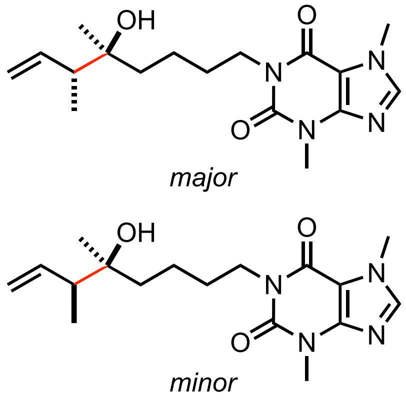

8aj

The reaction was conducted using pentoxifylline (**7aj**) (34.8 mg, 0.125 mmol, 1.0 equiv.) and liquid 2-butene (**2a**) (ca. 250 µL) for 24 hours. The residue was purified by silica gel flash column chromatography (MeOH/DCM = 5%, v/v) to afford **8aj** as a yellow oil (38.4 mg, 92% dr = 2.0/1).

^{1}H NMR ($CDCl_3$, 500 MHz): diastereomeric mixture δ 7.48 (1H, s), 5.86-5.72 (1H, m), 5.04-5.02 (2H, m), 4.01-3.99 (2H, m), 3.95 (3H, s), 3.53 (3H, s), 2.23-2.20 (1H, m), 1.70-1.34 (7H, m), 1.07 (3H, s, *minor*), 1.05 (3H, s, *major*), 1.00 (3H, d, J = 6.9 Hz, *minor*), 0.97 (3H, d, J = 6.9 Hz, *major*); ^{13}C NMR ($CDCl_3$, 126 MHz): diastereomeric mixture δ 155.5, 151.6, 148.8, 141.6, 141.5, 140.7, 140.5, 116.2, 115.8, 107.7, 73.7, 73.6, 48.1, 47.4, 41.1, 41.1, 39.2, 38.8, 33.7,

29.8, 28.4, 28.4, 23.8, 23.6, 20.4, 20.4, 14.9, 14.6; m/z calcd for $C_{17}H_{36}N_4O_3Na$ $[M+Na]^+$ 357.1903. Found 357.1894; IR (neat): 3620, 3053, 2993, 1702, 1659, 1602, 1545, 1492, 1455, 1435, 1415, 1351, 1325, 1261, 1228, 1191, 1051, 1004, 894 cm^{-1}

Methyl (4-(2-hydroxy-3-methylpent-4-en-2-yl)benzoyl)-*L*-phenylalanylglycinate (8ak)

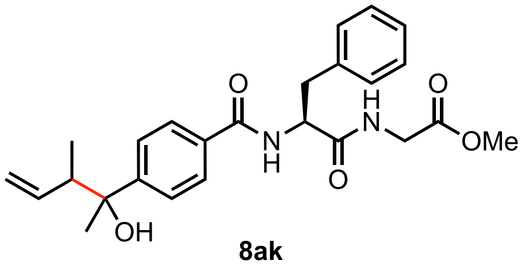

8ak

The reaction was conducted using methyl (4-acetylbenzoyl)-*L*-phenylalanylglycinate (**7ak**) (38.2 mg, 0.100 mmol, 1.0 equiv.) and liquid 2-butene (**2a**) (ca. 250 µL) for 24 hours. The residue was purified by silica gel flash column chromatography (MeOH/DCM = 3%, v/v) to afford **8ak** as a yellow solid (34.8 mg, 79% dr = 1.4/1).

^{1}H NMR ($CDCl_3$, 392 MHz): diastereomeric mixture δ 7.64 (2H, d, *J* = 8.1 Hz), 7.41-7.39 (2H, m), 7.25-7.22 (5H, m), 7.03 (1H, br s), 6.85 (1H, br s), 5.82-5.73 (1H, m, *minor*), 5.67-5.58 (1H, m, *major*), 5.10-5.05 (2H, m), 4.96-4.95 (1H, m), 4.01-3.88 (2H, m), 3.68 (3H, s), 3.21 (2H, d, *J* = 7.2 Hz), 2.56-2.48 (1H, m), 2.22 (1H, br s), 1.49 (3H, s, *major*), 1.48 (3H, s, *minor*), 0.93 (3H, d, *J* = 7.0 Hz, *major*), 0.80 (3H, d, *J* = 7.0 Hz, *minor*); ^{13}C NMR ($CDCl_3$, 99 MHz): diastereomeric mixture δ 171.6, 169.9, 167.4, 151.3, 139.5, 139.5, 136.7, 132.0, 131.8, 129.5, 128.8, 127.2, 126.9, 126.9, 126.0, 125.7, 117.1, 116.9, 75.9, 75.8, 54.7, 52.5, 48.9, 48.8, 41.3, 38.3, 28.6, 26.1, 26.1, 14.8, 14.1 ; m/z calcd for $C_{25}H_{30}N_2O_5Na$ $[M+Na]^+$ 461.2052. Found 461.2047; IR (neat): 3433, 3300, 3066, 2993, 1746, 1642, 1518, 1495, 1442, 1375, 1211, 1014, 920, 894, 850 cm^{-1}

Note: In the ^{13}C NMR spectra additional signals were observed. These correspond to the pseudoenantiomer forms of the product. In the ^{1}H NMR spectra the signals could not be distinguished.

4-(2-Hydroxy-3,3-dimethylpent-4-en-2-yl)benzonitrile (8aq)

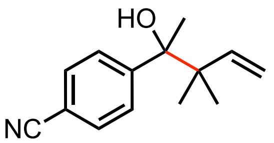

8aq

The reaction was conducted using 4-acetylbenzonitrile (**7i**) (18.1 mg, 0.125 mmol, 1.0 equiv.) and liquid 3-methylbut-1-ene (**2aq**) (ca. 250 µL) for 24 hours. The residue was purified by silica gel flash column chromatography (EtOAc/hexane = 12%, v/v) to afford **8aq** as a colorless oil (24.9 mg, 93%).

^{1}H NMR ($CDCl_3$, 392 MHz): δ 7.58-7.54 (4H, m), 5.90 (1H, dd, *J* = 17.5, 10.8 Hz), 5.14 (1H, dd, *J* = 10.8, 1.3 Hz), 5.04 (1H, *d*, J = 17.5 Hz), 2.02 (1H, s), 1.57 (3H, s), 0.99 (3H, s), 0.96 (3H, s); ^{13}C NMR ($CDCl_3$, 99 MHz): δ 150.9, 144.2, 131.0, 128.1, 119.1, 114.9, 110.5, 77.6, 44.5, 25.3, 22.8, 22.3; m/z calcd for $C_{14}H_{17}NONa$ $[M+Na]^+$ 238.1208. Found 238.1208; IR (neat): 3500, 3073, 2993, 2873, 2220, 1605, 1505, 1465, 1401, 1371, 1264, 1168, 1131, 1097, 1024, 917, 840, 733 cm^{-1}

5-2. Procedure for preparation of 8al-ap and 8ar-ay

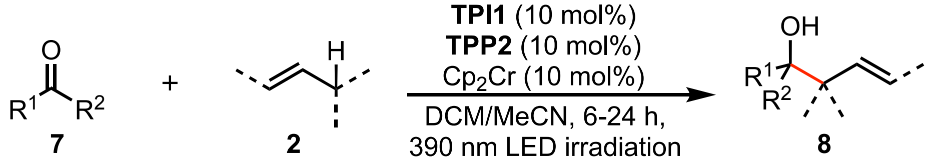

In an argon-filled glove box, Cp_2Cr (2.3 mg, 0.0125 mmol, 10 mol%), **TPI1** (6.2 mg, 0.0125 mmol, 10 mol%) and **TPP2** (4.2 mg, 0.0125 mmol, 10 mol%) were dissolved in degassed CH_2Cl_2 (1.125 mL) and MeCN (0.125 mL) in a screw-capped vial. Then, ketone **7** (0.125 mmol, 1.0 equiv.) and alkene **2** (0.625 mmol, 5.0 equiv.) were added to the reaction mixture. The reaction tube was removed from the glove box and subjected to 390 nm LED irradiation by a Kessil PR160 LED Photo Reaction Lighting PR160-390 nm for 6-24 hours cooling with a fan. Then, to the reaction mixture was added 1 N HCl aqueous solution. Organic materials were extracted with CH_2Cl_2 three times. Combined organic layers were dried over Na_2SO_4, filtered. After evaporation, diastereomeric ratio was determined by ^{1}H NMR analysis. The residue was purified by silica gel flash column chromatography to afford the target tertiary homoallylic alcohols **8**.

4-(2-Hydroxy-3-vinylhexan-2-yl)benzonitrile (8al)

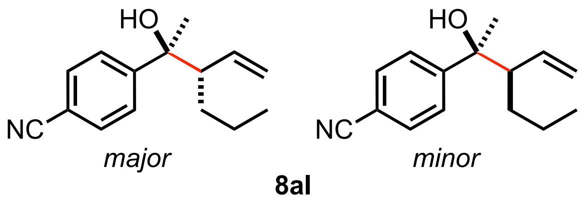

The reaction was conducted using 4-acetylbenzonitrile (**7i**) (18.1 mg, 0.125 mmol, 1.0 equiv.) and 1-hexene (**2al**) (77.8 μL, 0.625 mmol, 5.0 equiv.) for 24 hours. The residue was purified by silica gel flash column chromatography (EtOAc/hexane = 15%, v/v) to afford **8al** as a yellow oil (26.3 mg, 92%, dr = 2.3/1).

^{1}H NMR ($CDCl_3$,392 MHz): diastereomeric mixture δ 7.62-7.61 (2H, m), 7.55-7.49 (2H, m), 5.61-5.45 (1H, m), 5.21-5.19 (1H, m), 5.11-5.06 (1H, m), 2.26-2.22 (1H, m), 2.19 (1H, br s, *major*), 2.03 (1H, br s, *minor*), 1.54 (3H, s, *major*), 1.50 (3H, s, *minor*), 1.37-0.97 (4H, m), 0.79-0.74 (3H, m); ^{13}C NMR ($CDCl_3$, 125 MHz): diastereomeric mixture δ 153.0, 152.2, 138.1, 137.7, 131.9, 131.8, 126.9, 126.3, 119.7, 119.1, 119.1, 110.6, 75.6, 56.4, 55.2, 30.8, 30.7, 28.9, 25.7, 20.9, 20.7, 14.0, 13.9; m/z calcd for $C_{15}H_{19}NONa$ $[M+Na]^+$ 252.1364. Found 252.1364; IR (neat):3586, 3060, 2960, 2873, 1609, 1508, 1428, 1381, 1338, 1198, 1067, 1004, 930, 897, 847 cm^{-1}

4-(2-Hydroxy-3-vinyldecan-2-yl)benzonitrile (8am)

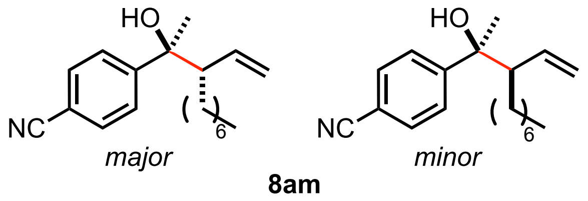

The reaction was conducted using 4-acetylbenzonitrile (**7i**) (18.1 mg, 0.125 mmol, 1.0 equiv.) and 1-decene (**2am**) (118 μL, 0.625 mmol, 5.0 equiv.) for 24 hours. The residue was purified by silica gel flash column chromatography (EtOAc/hexane = 12%, v/v) to afford **8am** as a yellow oil (32.9 mg, 92%, dr = 1.9/1).

^{1}H NMR ($CDCl_3$, 392 MHz): diastereomeric mixture δ 7.63-7.61 (2H, m), 7.56-7.50 (2H, m), 5.61-5.45 (1H, m), 5.23-5.18 (1H, m), 5.11-5.07 (1H, m), 2.27-2.20 (1H, m), 2.17 (1H, brs, *major*) 1.99 (1H, brs, *minor*), 1.52 (3H, s, *major*), 1.50 (3H, s, *minor*), 1.37-0.92 (12H, m), 0.85-0.83 (3H, m); ^{13}C NMR ($CDCl_3$, 99 MHz): diastereomeric mixture δ 153.0, 152.2, 138.1, 137.7, 131.9, 131.8, 126.9, 126.3, 119.8, 119.1, 110.6, 110.4, 76.2, 75.6, 56.6, 55.5, 31.9, 29.5, 29.4, 29.2, 29.0, 28.6, 28.5, 27.8, 27.7, 25.6, 22.7, 14.2; m/z calcd for $C_{19}H_{27}NONa$ $[M+Na]^+$ 308.1990. Found 308.1986; IR (neat): 3513, 3066, 2933, 2840, 2240, 1635, 1605, 1508, 1458, 1408,

1378, 1258, 1191, 1154, 1077, 1001, 907, 837, 790, 747, 723, 686, 663, 593, 553 cm^{-1}

4-(2-Hydroxy-5-methyl-3-vinylhexan-2-yl)benzonitrile (8an)

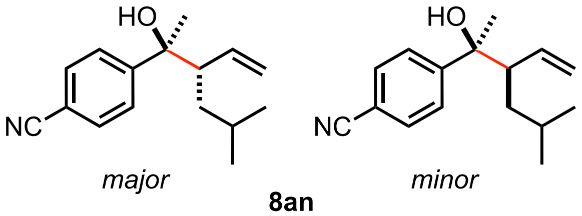

8an

The reaction was conducted using 4-acetylbenzonitrile (**7i**) (18.1 mg, 0.125 mmol, 1.0 equiv.) and 5-methylhex-1-ene (**2an**) (88.4 µL, 0.625 mmol, 5.0 equiv.) for 24 hours. The residue was purified by silica gel flash column chromatography (EtOAc/hexane = 15%, v/v) to afford **8an** as a yellow oil (24.5 mg, 81%, dr = 3.6/1).

^{1}H NMR ($CDCl_3$, 392 MHz): diastereomeric mixture δ 7.63-7.57 (2H, m), 7.53-7.51 (2H, m), 5.55-5.50 (1H, m), 5.21-5.11 (2H, m), 2.37-2.31 (1H, m), 2.17 (1H, s, *major*), 1.98 (1H, s, *minor*), 1.53 (3H, s, *major*), 1.50 (3H, s, *minor*), 1.43-1.40 (1H, m), 1.25-1.09 (1H, m), 1.02-0.96 (1H, m), 0.82-0.77 (3H, m), 0.68-0.66 (3H, m); ^{13}C NMR ($CDCl_3$, 99 MHz): diastereomeric mixture δ 152.9, 152.2, 138.2, 137.7, 131.9, 131.8, 126.9, 126.3, 119.6, 119.1, 119.0, 110.6, 76.2, 75.6, 54.4, 53.2, 37.9, 37.8, 29.2, 25.6, 25.4, 25.2, 24.2, 20.7; m/z calcd for $C_{16}H_{21}NONa$ [M+Na]$^+$ 266.1521. Found 266.1521; IR (neat): 3507, 3075, 2954, 2874, 2362, 2241, 1638, 1604, 1503, 1469, 1408, 1365, 1340, 1202, 1081, 1010, 909, 840, 777, 737, 700, 588 cm^{-1}

4-((2*S*,3*S*)-2-Hydroxy-3-isopropylpent-4-en-2-yl)benzonitrile (8ao)

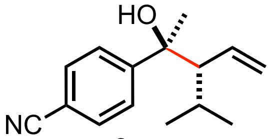

8ao

The reaction was conducted using 4-acetylbenzonitrile (**7i**) (18.1 mg, 0.125 mmol, 1.0 equiv.) and 4-methylpent-1-ene (**2ao**) (79.2 µL, 0.625 mmol, 5.0 equiv.) for 24 hours. The residue was purified by silica gel flash column chromatography (EtOAc/hexane = 12%, v/v) to afford **8ao** as a colorless oil (20.9 mg, 73%, dr = >20/1).

^{1}H NMR ($CDCl_3$, 392 MHz): δ 7.62-7.52 (4H, m), 5.73-5.68 (1H, ddd, J = 17.1, 10.3, 10.1 Hz), 5.15 (1H, dd, J = 10.1, 2.2 Hz), 4.94 (1H, dd, J = 17.1, 2.2 Hz), 2.14 (1H, dd, J = 10.3, 2.2 Hz), 2.01 (1H, s), 1.93-1.91 (1H, m), 1.59 (3H, s), 0.77 (3H, d, J = 7.2 Hz), 0.72 (3H, d, J = 6.7 Hz); ^{13}C NMR ($CDCl_3$, 99 MHz): δ 152.9, 134.2, 131.8, 126.7, 120.4, 119.1, 110.5, 76.3, 62.0, 27.5, 26.8, 23.9, 18.4; m/z calcd for $C_{15}H_{19}NONa$ [M+Na]$^+$ 252.1364. Found 252.1365; IR (neat): 3486, 3080, 2966, 2220, 1632, 1605, 1502, 1455, 1408, 1365, 1171, 1134, 1081, 1007, 924, 840, 750, 593, 543 cm^{-1}

4-(1-(Cyclohex-2-en-1-yl)-1-hydroxyethyl)benzonitrile (8ap)

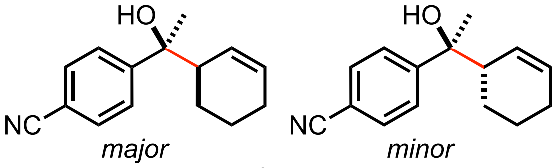

8ap

The reaction was conducted using 4-acetylbenzonitrile (**7i**) (18.1 mg, 0.125 mmol, 1.0 equiv.) and cyclohexene (**2ap**) (63.3 µL, 0.625 mmol, 5.0 equiv.) for 24 hours. The residue was purified by silica gel flash column chromatography (EtOAc/hexane = 20%, v/v) to afford **8ap** as a colorless oil (9.2 mg, 32%, dr = 3.4/1).

^{1}H NMR ($CDCl_3$, 392 MHz): diastereomeric mixture δ 7.64-7.61 (2H, m), 7.57-7.53 (2H, m), 6.01-5.98 (1H, m, *major*), 5.89-5.87 (1H, m, *minor*), 5.80-5.78 (1H, m, *major*), 5.22-5.20 (1H, m, *minor*), 2.65 (1H, d, J = 2.2

Hz, *minor*), 2.55-2.53 (1H, m, *major*), 1.96-1.92 (2H, m), 1.87 (1H, s, *minor*), 1.83 (1H, s, *major*), 1.72-1.69 (1H, m), 1.59 (3H, s, *major*), 1.55-1.14 (4H, m), 1.48 (3H, s, *minor*); ^{13}C NMR ($CDCl_3$, 99 MHz): diastereomeric mixture δ 152.6, 133.2, 132.9, 132.1, 132.0, 126.2, 125.9, 125.3, 119.2, 110.4, 76.4, 76.1, 46.3, 46.2, 28.4, 26.9, 25.2, 24.4, 23.8, 21.9, 21.8; m/z calcd for $C_{15}H_{17}NONa$ $[M+Na]^+$ 250.1208. Found 250.1208; IR (neat): 3493, 3033, 2926, 2880, 2246, 1652, 1609, 1502, 1448, 1398, 1368, 1271, 1201, 1071, 1007, 880, 837, 737, 646, 590, 559 cm^{-1}

All the spectroscopic data were matched with the previously reported data[20)].

4-(2-Hydroxy-3-phenylpent-4-en-2-yl)benzonitrile (8ar)

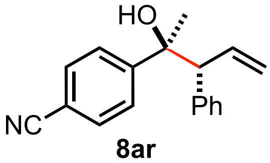

8ar

The reaction was conducted using 4-acetylbenzonitrile (**7i**) (18.1 mg, 0.125 mmol, 1.0 equiv.) and allylbenzene (**2ar**) (82.5 µL, 0.625 mmol, 5.0 equiv.) for 24 hours. The residue was purified by silica gel flash column chromatography (EtOAc/hexane = 15%, v/v) to afford **8ar** as a white solid (21.2 mg, 65%, dr = 15/1).

^{1}H NMR ($CDCl_3$, 500 MHz): δ 7.59 (2H, d, *J* = 8.3 Hz), 7.46 (2H, d, *J* = 8.3 Hz), 7.31-7.26 (3H, m), 7.13 (2H, d, *J* = 6.3 Hz), 6.10 (1H, ddd, *J* = 17.8, 9.6, 8.3 Hz), 5.07 (1H, dd, *J* = 9.6, 1.0 Hz), 4.94 (1H, dd, *J* = 17.8, 1.0 Hz), 3.57 (1H, d, *J* = 8.3 Hz), 2.09 (1H, s), 1.45 (3H, s); ^{13}C NMR ($CDCl_3$, 126 MHz): δ 151.9, 139.4, 136.6, 131.8, 129.5, 128.5, 127.3, 126.6, 119.1, 118.8, 110.6, 76.3, 61.9, 28.3; m/z calcd for $C_{18}H_{17}NONa$ $[M+Na]^+$ 286.1208. Found 286.1206; IR (neat): 3468, 3074, 2994, 2240, 1609, 1502, 1456, 1394, 1368, 1263, 1199, 1159, 1119, 1062, 1001, 923, 840, 720, 698, 663, 599, 567, 540 cm^{-1}

All the spectroscopic data were matched with the previously reported data[21)].

4-(3-(4-Chlorophenyl)-2-hydroxypent-4-en-2-yl)benzonitrile (8as)

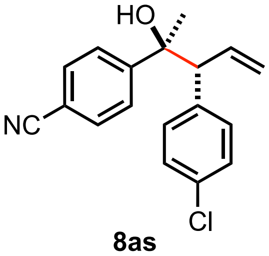

8as

The reaction was conducted using 4-acetylbenzonitrile (**7i**) (18.1 mg, 0.125 mmol, 1.0 equiv.) and 1-allyl-4-chlorobenzene (**2as**) (95.4 mg, 0.625 mmol, 5.0 equiv.) for 24 hours. The residue was purified by silica gel flash column chromatography (EtOAc/hexane = 30%, v/v) to afford **8as** as a white solid (31.8 mg, 85%, dr = >20/1).

^{1}H NMR ($CDCl_3$, 500 MHz): 7.60 (2H, d, *J* = 8.3 Hz), 7.47 (2H, d, *J* = 8.3 Hz), 7.27 (2H, d, *J* = 8.6 Hz), 7.09 (2H, d, *J* = 8.6 Hz), 6.04 (1H, ddd, *J* = 17.6, 9.7, 8.3 Hz), 5.07 (1H, dd, *J* = 9.7, 1.3 Hz), 4.91 (1H, dd, *J* = 17.6, 1.3 Hz), 3.56 (1H, d, *J* = 8.3 Hz), 2.07-2.06 (1H, br m), 1.44 (3H, s); ^{13}C NMR ($CDCl_3$, 99 MHz): δ 151.8, 138.1, 136.2, 133.1, 131.9, 130.9, 128.5, 126.5, 119.1, 119.0, 110.7, 76.3, 61.1, 28.3; m/z calcd for $C_{18}H_{16}ClNONa$ $[M+Na]^+$ 320.0818. Found 320.0811; IR (neat): 3452, 2984, 2273, 1905, 1491, 1406, 1272, 1198, 1171, 1121, 1082, 1055, 1008, 955, 910, 895, 847, 786, 750, 684, 610, 581 cm^{-1}

4-(6-Chloro-2-hydroxy-3-vinylhexan-2-yl)benzonitrile (8at)

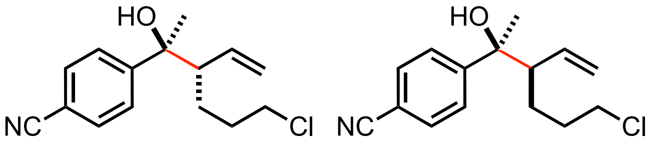

major *minor*

8at

The reaction was conducted using 4-acetylbenzonitrile (**7i**) (18.1 mg, 0.125 mmol, 1.0 equiv.) and 6-chlorohex-1-ene (**2at**) (82.3 µL, 0.625 mmol, 5.0 equiv.) for 24 hours. The residue was purified by silica gel flash column chromatography (EtOAc/hexane = 20%, v/v) to afford **8at** as a colorless oil (18.4 mg, 56%, dr = 2.4/1).

^{1}H NMR ($CDCl_3$, 392 MHz): diastereomeric mixture δ 7.64-7.62 (2H, m), 7.55-7.51 (2H, m), 5.63-5.59 (1H, m, *minor*), 5.51-5.46 (1H, m, *major*), 5.25 (1H, dd, *J* = 10.3, 1.8 Hz, *minor*), 5.21 (1H, dd, *J* = 10.3, 1.8 Hz, *major*), 5.10 (1H, dd, *J* = 17.1, 1.8 Hz), 3.49-3.34 (2H, m), 2.30-2.21 (1H, m), 2.09 (1H, s, *major*), 1.91 (1H, s, *minor*), 1.79-1.14 (4H, m), 1.57 (3H, s, *major*), 1.52 (3H, s, *minor*); ^{13}C NMR ($CDCl_3$, 99 MHz): diastereomeric mixture δ 151.7, 137.4, 137.1, 132.0, 131.9, 126.9, 126.2, 120.2, 119.7, 119.0, 110.8, 76.1, 75.6, 55.9, 54.8, 44.9, 44.8, 30.8, 29.0, 26.3, 25.8; m/z calcd for $C_{15}H_{18}ClNONa$ $[M+Na]^+$ 286.0975. Found 286.0975; IR (neat): 3486, 3080, 2973, 2233, 1642, 1602, 1508, 1448, 1408, 1378, 1295, 1148, 1074, 1007, 920, 840, 760, 727, 700, 643, 590 cm^{-1}

4-(2-Hydroxy-3-methoxypent-4-en-2-yl)benzonitrile (8au)

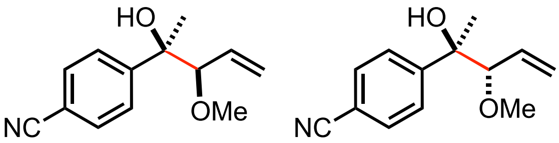

major *minor*

8au

The reaction was conducted using 4-acetylbenzonitrile (**7i**) (18.1 mg, 0.125 mmol, 1.0 equiv.) and 3-methoxyprop-1-ene (**2au**) (58.6 µL, 0.625 mmol, 5.0 equiv.) for 24 hours. The residue was purified by silica gel flash column chromatography (EtOAc/hexane = 20%, v/v) to afford **8au** as a colorless oil (18.8 mg, 69%, dr = 2.0/1).

^{1}H NMR ($CDCl_3$, 500 MHz): diastereomeric mixture δ 7.64-7.49 (4H, m), 5.67 (1H, ddd, *J* = 17.8, 10.0, 7.5 Hz, *major*), 5.46-5.39 (1H, m, *minor*), 5.37 (1H, dd, *J* = 10.0, 0.9 Hz, *major*), 5.23 (1H, dd, *J* = 10.5, 1.1 Hz, *minor*), 5.19-5.15 (1H, m), 3.62-3.60 (1H, m), 3.29 (3H, s, *minor*), 3.23 (3H, s, *major*), 3.08-3.07 (1H, br m), 1.58 (3H, s, *minor*), 1.44 (3H, s, *major*); ^{13}C NMR ($CDCl_3$, 99 MHz): diastereomeric mixture δ 151.2, 150.1, 133.6, 133.3, 131.9, 131.7, 126.8, 126.7, 121.6, 121.1, 119.1, 110.8, 110.7, 89.3, 89.2, 76.0, 75.6, 57.1, 57.0, 26.3, 24.7; m/z calcd for $C_{13}H_{15}NO_2Na$ $[M+Na]^+$ 240.1000. Found 240.0999; IR (neat): 3486, 3066, 2986, 2933, 2820, 2233, 1639, 1605, 1502, 1455, 1398, 1341, 1194, 1084, 994, 944, 837, 747, 696, 596, 543 cm^{-1}

4-(2-Hydroxy-3-phenoxypent-4-en-2-yl)benzonitrile (8av)

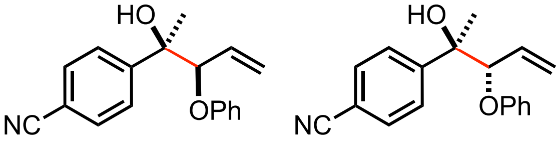

major *minor*

8av

The reaction was conducted using 4-acetylbenzonitrile (**7i**) (18.1 mg, 0.125 mmol, 1.0 equiv.) and (allyloxy)benzene (**2av**) (86 µL, 0.625 mmol, 5.0 equiv.) for 24 hours. The residue was purified by silica gel flash column chromatography (EtOAc/hexane = 20%, v/v) to afford **8av** as a yellow oil (20.0 mg, 57%, dr = 4.5/1).

^{1}H NMR ($CDCl_3$, 392 MHz): diastereomeric mixture δ 7.68-7.63 (4H, m), 7.27-7.24 (2H, m), 6.99-6.82 (3H,

m), 5.83-5.74 (1H, m, *major*), 5.70-5.61 (1H, m, *minor*), 5.38-5.20 (2H, m), 4.68-4.66 (1H, m), 2.98 (1H, s, *major*), 2.81 (1H, s, *minor*), 1.72 (3H, s, *minor*), 1.63 (3H, s, *major*); ^{13}C NMR ($CDCl_3$, 99 MHz): diastereomeric mixture δ 157.7, 150.2, 133.0, 132.8, 132.0, 129.6, 126.9, 126.7, 122.0, 121.9, 121.1, 119.0, 116.5, 116.3, 111.2, 85.9, 85.3, 76.3, 76.0, 26.3, 24.8; m/z calcd for $C_{18}H_{17}NO_2Na$ $[M+Na]^+$ 302.1157. Found 302.1152; IR (neat): 3480, 3046, 2986, 2226, 1595, 1492, 1401, 1368, 1305, 1244, 1168, 1094, 1004, 930, 847, 753, 700, 596, 556 cm^{-1}

5-Methyl-2-phenyl-3-vinylhexan-2-ol (8aw)

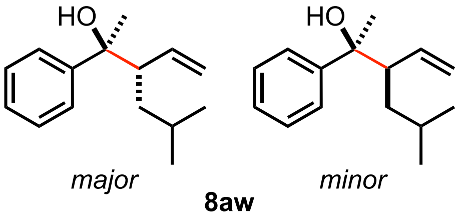

8aw

The reaction was conducted using acetophenone (**7b**) (14.6 μL, 0.125 mmol, 1.0 equiv.) and 5-methylhex-1-ene (**2an**) (88.4 μL, 0.625 mmol, 5.0 equiv.) for 24 hours. The residue was purified by silica gel flash column chromatography (EtOAc/hexane = 10%, v/v) to afford **8aw** as a colorless oil (9.2 mg, 34%, dr = 6.3/1).

^{1}H NMR ($CDCl_3$, 500 MHz): δ 7.42-7.37 (2H, m), 7.34-7.31 (2H, m), 7.25-7.22 (1H, m), 5.61-5.56 (1H, m), 5.20-5.07 (2H, m), 2.42-2.36 (1H, m), 2.08 (1H, s, *major*), 1.92 (1H, s, *minor*), 1.53 (3H, s, *major*), 1.51 (3H, s, *minor*), 1.46-1.38 (1H, m), 1.15-1.10 (1H, m), 1.04-0.99 (1H, m), 0.80 (3H, d, *J* = 6.9 Hz), 0.71 (3H, d, *J* = 6.3 Hz, *minor*), 0.66 (3H, d, *J* = 6.3 Hz, *major*); ^{13}C NMR ($CDCl_3$, 99 MHz): *major* δ 146.6, 139.1, 127.9, 126.8, 126.0, 118.8, 75.6, 54.5, 38.1, 25.5, 25.5, 24.3, 20.8; m/z calcd for $C_{15}H_{22}ONa$ $[M+Na]^+$ 241.1568. Found 241.1568; IR (neat): 3480, 3073, 2993, 1632, 1609, 1498, 1468, 1442, 1418, 1371, 1061, 1031, 001, 910, 860, 760, 700, 666, 600, 559 cm^{-1}

2,3-Diphenylpent-4-en-2-ol (8ax)

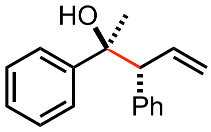

8ax

The reaction was conducted using acetophenone (**7b**) (14.6 μL, 0.125 mmol, 1.0 equiv.) and allylbenzene (**2ar**) (82.5 μL, 0.625 mmol, 5.0 equiv.) for 24 hours. 20 mol% of Cp_2Cr (0.025 mol) was used. The residue was purified by silica gel flash column chromatography (Et_2O/hexane = 10%, v/v) to afford **8ax** as a white solid (13.2 mg, 44%, dr = > 20/1).

^{1}H NMR ($CDCl_3$, 500 MHz): δ 7.36-7.22 (8H, m), 7.16-7.13 (2H, m), 6.14 (1H, ddd, *J* = 17.6, 9.9, 8.2 Hz), 5.07 (1H, dd, *J* = 9.9, 1.1 Hz), 4.95 (1H, dd, *J* = 17.6, 1.1 Hz), 3.65 (1H, d, *J* = 8.2 Hz), 2.02 (1H, s), 1.46 (3H, s); ^{13}C NMR ($CDCl_3$, 99 MHz): δ 146.5, 140.2, 137.5, 129.8, 128.2, 127.9, 126.9, 126.7, 125.6, 118.2, 76.4, 62.0, 28.6; m/z calcd for $C_{17}H_{18}ONa$ $[M+Na]^+$ 261.1255. Found 261.1261; IR (neat): 3560, 3480, 3073, 3026, 2986, 2946, 1949, 1639, 1599, 1498, 1445, 1378, 1064, 1027, 1001, 934, 747, 696, 566 cm^{-1}
All the spectroscopic data match with the previously reported data[11)].

3-Methyl-1-phenyl-4-vinylheptan-3-ol (8ay)

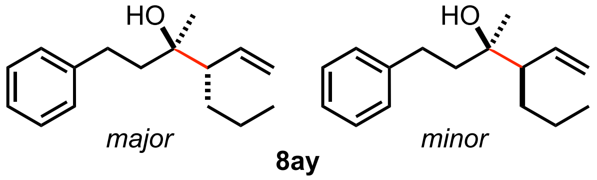

8ay

The reaction was conducted using 4-phenylbutan-2-one (**7a**) (18.7 μL, 0.125 mmol, 1.0 equiv.) and 1-hexene (**2al**) (77.8 μL, 0.625 mmol, 5.0 equiv.) for 24 hours. The residue was purified by silica gel flash column

chromatography (Et_2O/hexane = 12%, v/v) to afford **8ay** as a colorless oil (9.6 mg, 33%, dr = 1.5/1).

^{1}H NMR ($CDCl_3$, 500 MHz): δ 7.30-7.26 (2H, m), 7.23-7.16 (3H, m), 5.65-5.58 (1H, m), 5.24-5.09 (2H, m), 2.81-2.62 (2H, m), 2.13-2.06 (1H, m), 1.85-1.11 (10H, m), 0.90-0.89 (3H, m); ^{13}C NMR ($CDCl_3$, 99 MHz): δ 143.0, 139.1, 139.0, 128.5, 125.8, 119.1, 118.4, 73.7, 73.3, 55.4, 54.4, 42.2, 42.1, 31.4, 31.0, 29.9, 29.8, 24.2, 21.3, 14.2, 14.2; m/z calcd for $C_{16}H_{24}ONa$ $[M+Na]^+$ 255.1725. Found 255.1724; IR (neat): 3573, 3473, 3086, 3026, 2960, 2873, 1635, 1602, 1495, 1458, 1375, 1264, 1198, 1107, 1057, 997, 910, 743, 703 cm^{-1}

6. UV-vis analysis

Each substance was dissolved in CH_2Cl_2 /MeCN = 9/1 (0.01 M) in a screw-capped vial. After stirring for 1 h, 200 µL of the solution was transferred to a rectangular quartz cell (0.5 cm pathlength), and the UV-vis spectrum was measured.

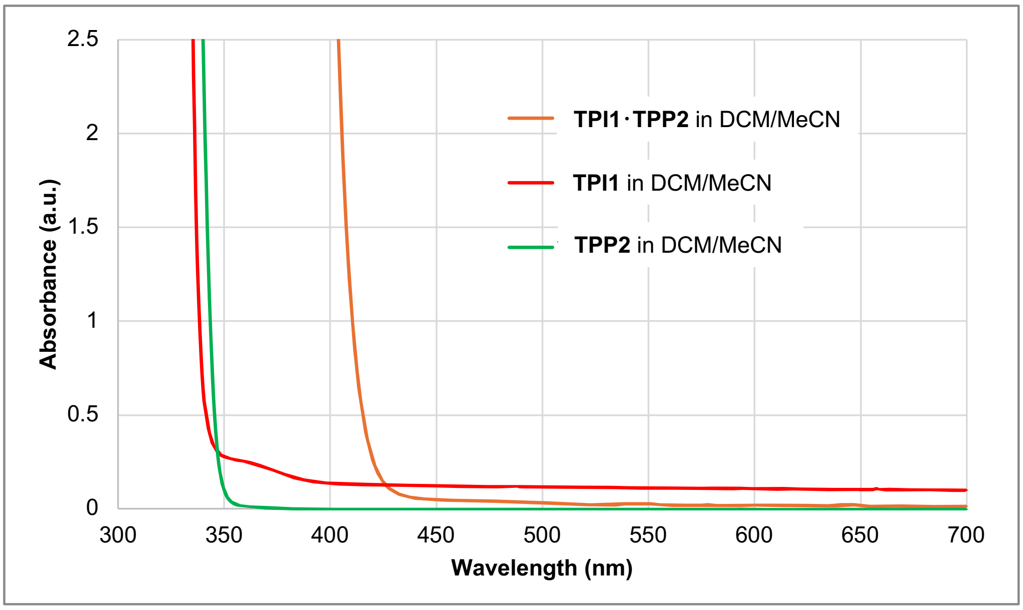

Absorption spectra of each reaction component

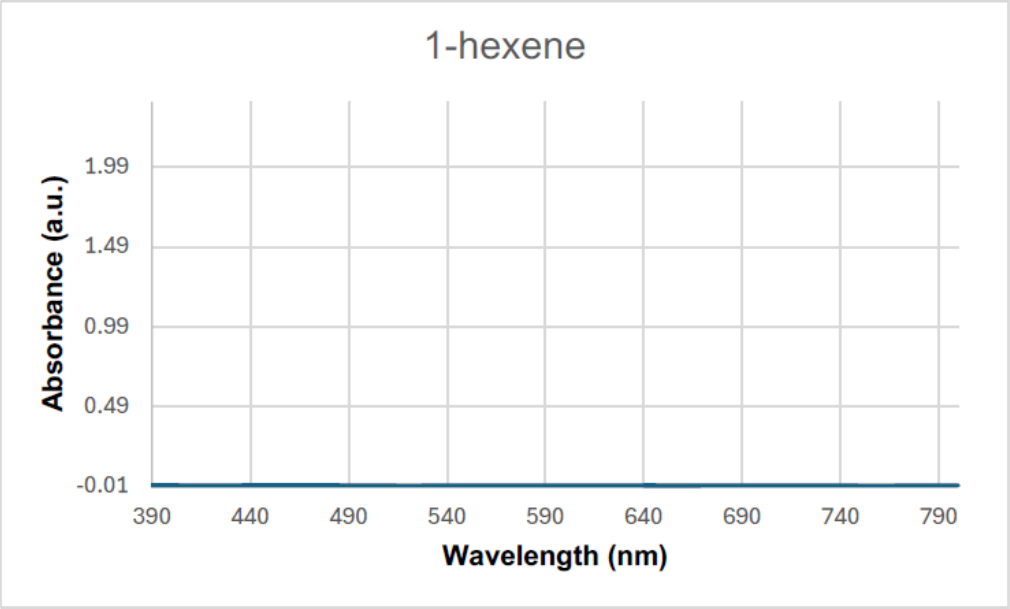

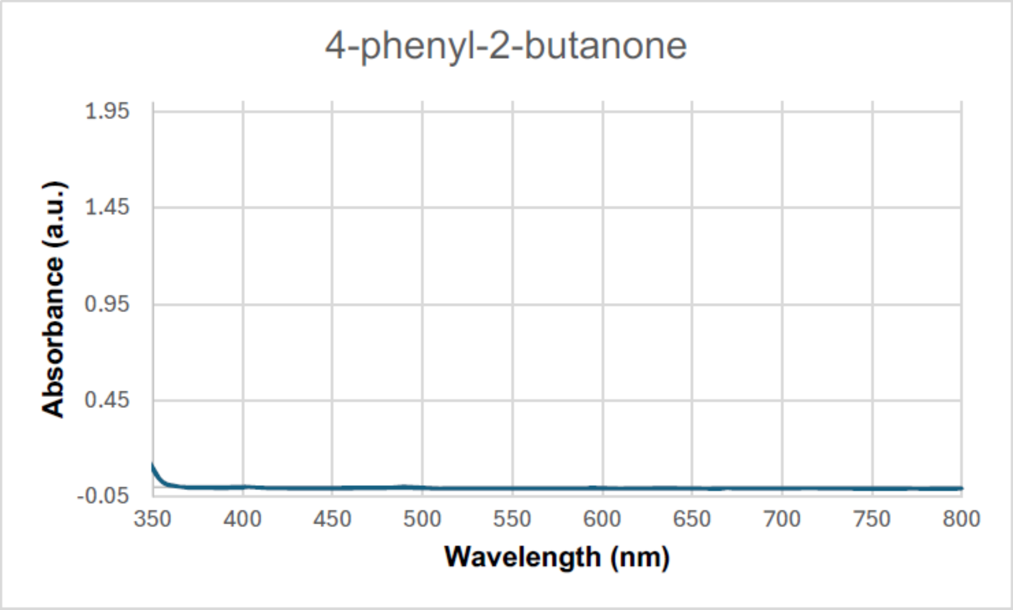
4-phenyl-2-butanone
Absorbance (a.u.)
1.95
1.45
0.95
0.45
-0.05
350
400
450
500
550
600
650
700
750
800
Wavelength (nm)

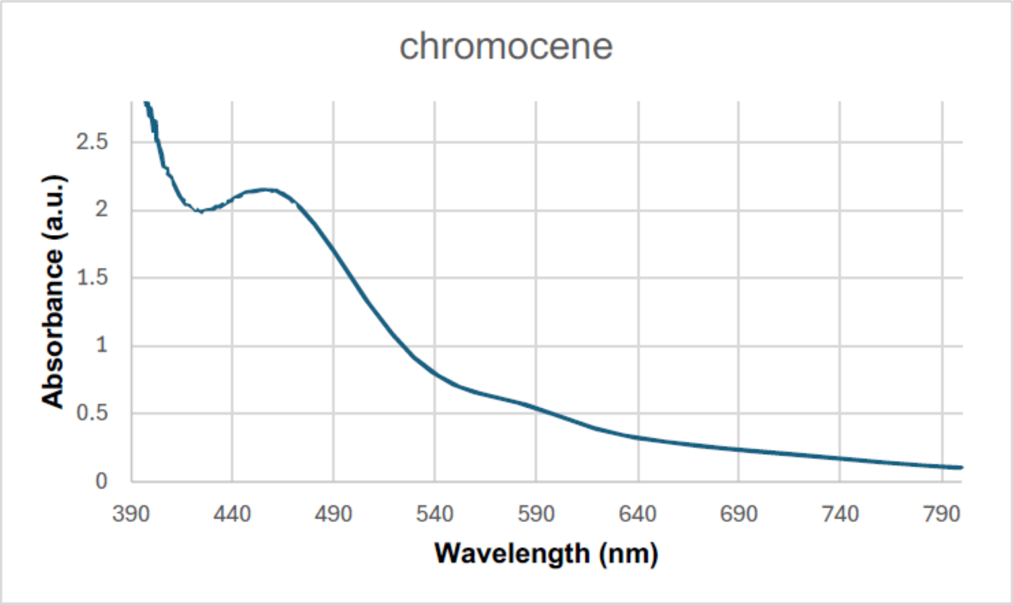
chromocene
Absorbance (a.u.)
2.5
2
1.5
1
0.5
0
390
440
490
540
590
640
690
740
790
Wavelength (nm)

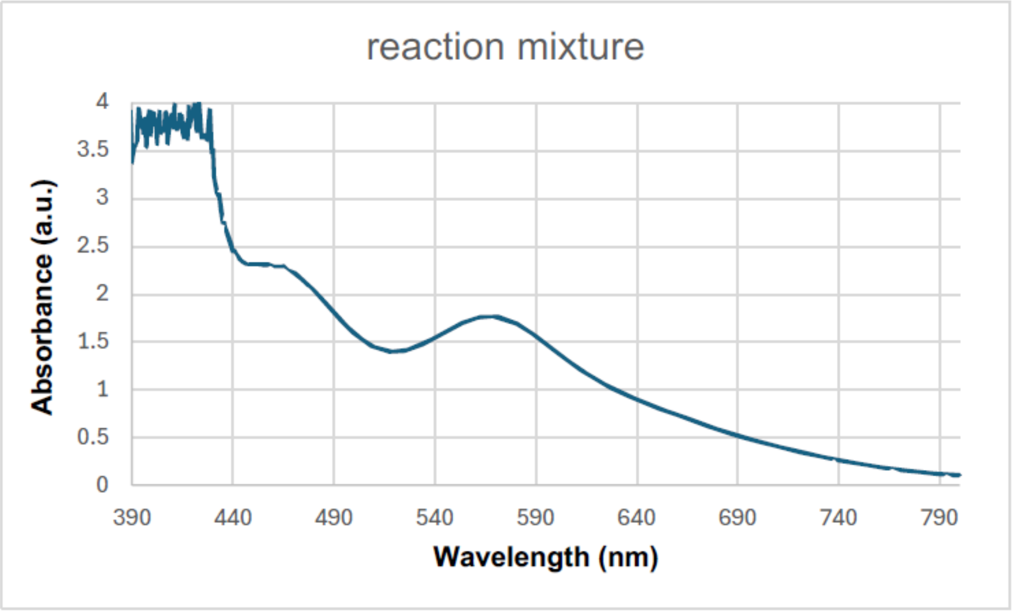

Figure S1. Absorption spectra of 1-hexene (0.5 M), 4-phenyl-2-butanone **7a** (0.1 M), chromocene (0.01 M) and the reaction mixture (1-hexene: 0.5 M, **7a**: 0.1 M, chromocene: 0.01 M, **TPI1**: 0.01 M, **TPP2**: 0.01 M). The results revealed that both ketone **7a** and alkene (1-hexene) exhibit negligible absorption at 390 nm. In contrast, chromocene displays a strong broad absorption extending over >650 nm, which is also observed in the reaction mixture.

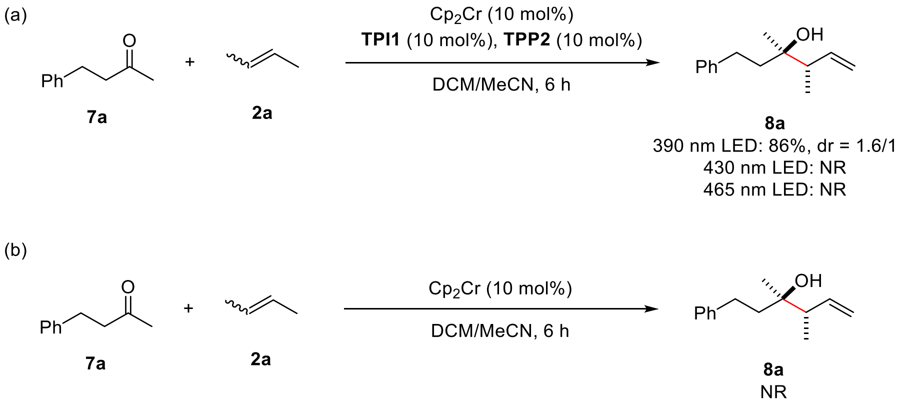

Scheme S1. Control experiments. (a) Reaction at different wavelength irradiation. The reaction was evaluated under varying irradiation wavelengths. In addition to the optimal conditions (390 nm light irradiation), reactions were carried out under irradiation at 430 nm and 465 nm, the wavelengths where the ion-pair catalyst exhibits minimal absorption. The results revealed that the reaction proceeded exclusively under optimal conditions, while no reaction was observed at 430 nm or 465 nm light irradiation. Therefore, the excitation of the ion-pair catalyst is essential for the reaction progress. (b) Reaction without **TPI1**/**TPP2** catalyst. To rule out the possibility that the reaction proceeds solely through the excitation of the chromocene catalyst, the reaction was performed in the absence of the ion-pair catalyst. Under these conditions, no reaction proceeded. Therefore, the chromocene catalyst alone does not promote the reaction. Based on these results, the ion-pair catalyst is indispensable for the reaction progress.

7. Acceleration of HAT Process by Ion-pair Catalyst

An advantage of the ion-pair catalyst lies in its ability to facilitate the generation of thiyl radical **1**, thereby accelerating the HAT process. To experimentally verify the acceleration of the HAT process, we conducted a comparative study using the allylation of aldehydes with simple alkenes, where the C–H abstraction step is rate-determining (*JACS*. **2020**, *142*, 12374. ref 11 in the main text).

Allylation of benzaldehyde using photoredox catalyst **PC1**

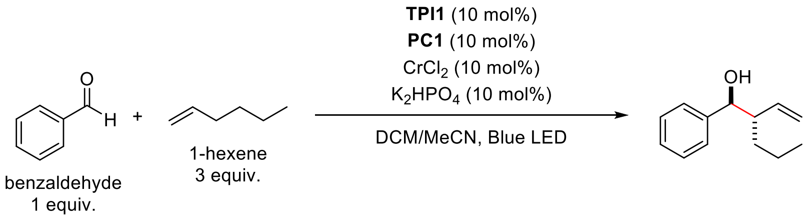

Allylation of benzaldehyde using ion-pair catalyst

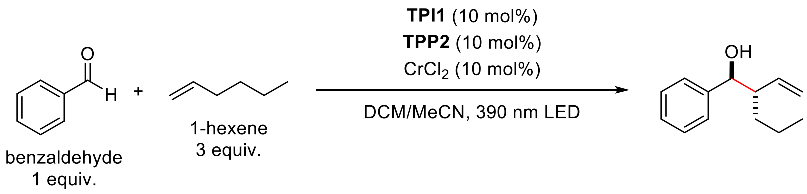

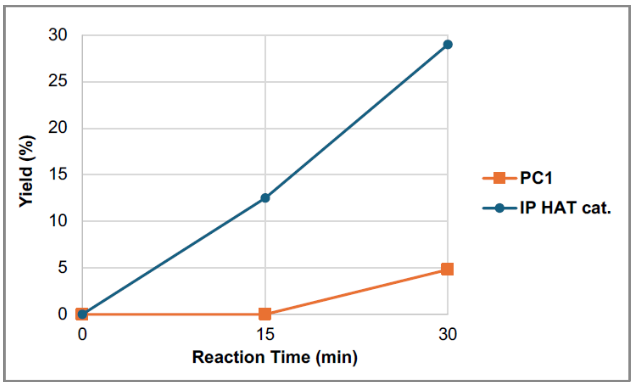

Figure S2. Reactivity differences between photoredox/HAT catalyst conditions (orange line) and ion-pair HAT catalyst conditions (blue line)

In an argon-filled glove box, $CrCl_2$ (1.50 mg, 0.0125 mmol, 10 mol%), **TPI1** (6.2 mg, 0.0125 mmol, 10 mol%) and **TPP2** (4.2 mg, 0.0125 mmol, 10 mol%) or **PC1** (6.2 mg, 0.0125 mmol, 10 mol%) and K_2HPO_4 (2.2 mg, 0.0125 mmol, 10 mol%) were dissolved in degassed CH_2Cl_2 (1.125 mL) and MeCN (0.125 mL) in a screw-capped test tube. Then, benzaldehyde (12.8 μl, 0.125 mmol, 1.0 equiv.) and 1-hexene (46.9 μl, 0.375 mmol, 3.0 equiv.) were added to the reaction mixture. The reaction tube was removed from the glove box and the reaction mixture was subjected to 390 nm LED irradiation by a Kessil PR160 LED Photo Reaction Lighting PR160-390 nm or a Valore VBP-L24-C2 with 38W LED lamp (VBL-SE150-BBB (430)) cooling with a fan. Then, the reaction mixture was diluted by hexane (ca. 2 mL) and passed through a pad of silica gel with CH_2Cl_2 elution. The reaction progress was checked by NMR in 15 and 30 min. The results revealed that, compared to the photoredox catalytic conditions, the ion-pair catalyst significantly enhanced the initial reaction rate. These findings indicate that the ion-pair catalyst efficiently generates HAT active radical **1**, thereby expediting C–H abstraction.

8. Reaction with Ketoesters

The reaction using ketoesters and 2-butene **2a** yielded a mixture of regioisomers (b/l = 1/1–1/2) in moderate yield. The reaction may proceed through an ally radical addition mechanism without the intervention of allylchromium, similar to the previous titanium complex-catalyzed reaction (Ref. 10 in the main text).

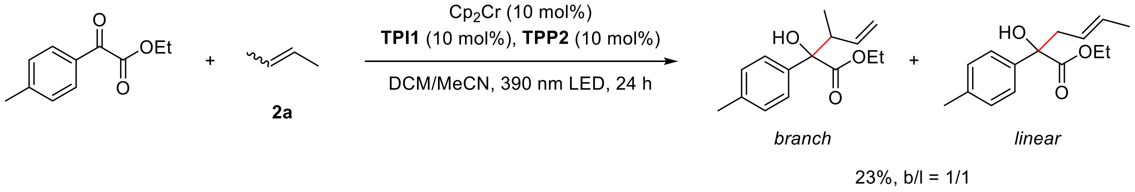

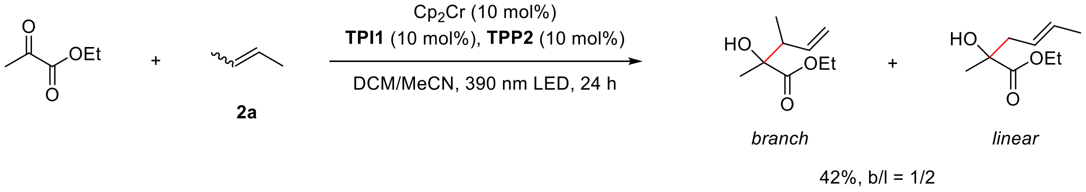

9. References

1) N. A. Romero, K. A. Margrey, N. E. Tay, D. A. Nicewicz, *Science* **2015**, *349*, 1326–1330.
2) [a] for **PC2**; A. Joshi-Pangu, F. Levésque, H. G. Roth, S. F. Oliver, L.-Charles Campeau, D. Nicewicz, D. A. DiRocco, *J. Org. Chem.* **2016**, *81*, 7244-7249. [b] for 4CzIPN; E. Speckmeier, T. G. Fischer, K. Zeitler, *J. Am. Chem. Soc.* **2018**, *140*, 15353. [c] for Ru(phen)$_3$Cl$_2$; M. M. Cookea, E. H. Doevenb, C. F. Hoganb, J. L. Adcocka, G. P. McDermotta, X. A. Conlanc, N. W. Barnetta, F. M. Pfeffera, P. S. Francis, *Analytica Chimica Acta* **2009**, *635*, 94. [d] for [Ir(dF-CF$_3$-ppy)(dtbppy)$_2$]PF$_6$; T. Koike, M. Akita, *Inorg. Chem. Front.* **2014**, *1*, 562. [e] for perylene; J. Merz, A. Steffen, J. Nitsch, J. Fink, C. B. Schürger, A. Friedrich, I. Krummenacher, H. Braunschweig, M. Moos, D. Mims, C. Lambert, T. B. Marder, *Chem. Sci.* **2019**, *32*, 10.
3) X. Peng, Y. Hirao, S. Yabu, H. Sato, M. Higashi, T. Akai, S. Masaoka, H. Mitsunuma, M. Kanai, *J. Org. Chem.* **2023**, *88*, 6333-6346.
4) Synthesis for **7k**; M. A. Hosny1, Y. H. Zaki, W. A. Mokbel, A. O. Abdelhamid, *BMC Chemistry,* **2019**, *13*, 37.
5) Synthesis for **7m**; X. Sun, W. Zhao, B.-Jie. Li, *Chem. Commun.*, **2020**, *56*, 1298.
6) Synthesis for **2as**; D. F. Taber, C. M. Paquette, P. Gu, W. Tian, *J. Org. Chem.* **2013**, *78*, 9772.
7) B. D. Schwartz, A. P. Smyth, P. E. Nashar, M. G. Gardiner, L. R. Malins, *Org. Lett.* **2022**, *24*, 1268.
8) S. Tanabe, H. Mitsunuma, M. Kanai, *J. Am. Chem. Soc.* **2020**, *142*, 12374.
9) S. Kato, Y. Saga, M. Kojima, H. Fuse, S. Matsunaga, A. Fukatsu, M. Kondo, S. Masaoka, M. Kanai, *J. Am. Chem. Soc.* **2017**, *139*, 2204.
10) H. Liu, S. Xu, X. Shi, *Inorg. Chem. Commun.* **2021**, *133*, 108885.
11) Y. Yatsumonji, T. Nishimura, A. Tsubouchi, K. Noguchi, T. Takeda, *Chem.-Eur. J.* **2009**, *15*, 2680.
12) M. K. Reilly, S. C. Rychnovsky, *Org. Lett.* **2010**, *12*, 4892.
13) X. Zhang, W. T. Teo, W. Rao, D.-L. Ma, C.-H. Leung, P. W. H. Chan, *Tetrahedron Letters* **2014**, *55*, 3881.
14) M. Yasuda, K. Hirata, M. Nishino, A. Yamamoto, A. Baba, *J. Am. Chem. Soc.* **2002**, *124*, 13442.
15) F. Nowrouzi, A. N. Thadani, R. A. Batey, *Org. Lett.* **2009**, *11*, 2631.
16) Y. Yatsumonji, T. Sugita, A. Tsubouchi, T. Takeda, *Org. Lett.* **2010**, *12*, 1968.
17) I. Suzuki, K. Yagi, S. Miyamoto, I. Shibata, *RSC Adv.*, **2020**, *10*, 6030.
18) F. W. Mei, C. Qin, R. J. Morrison, A. H. Hoveyda, *J. Am. Chem. Soc.* **2017**, *139*, 9053.
19) P. Dey, M. Koli, D. Goswami, A. Sharma, S. Chattopadhyay, *Eur. J. Org. Chem.* **2018**, *2018*, 1333.
20) H. Ren, G. Dunet, P. Mayer, P. Knochel, *J. Am. Chem. Soc.* **2007**, *129*, 5376.
21) Z. Peng, T. D. Blümke, P. Mayer, P. Knochel, *Angew. Chem. Int. Ed.* **2010**, *49*, 8516.

\\172.20.1.218\share_Instruments_Data_Data_NMR_files\Arii\NMRdata_complete\SA-7ak-proton-1-1.als

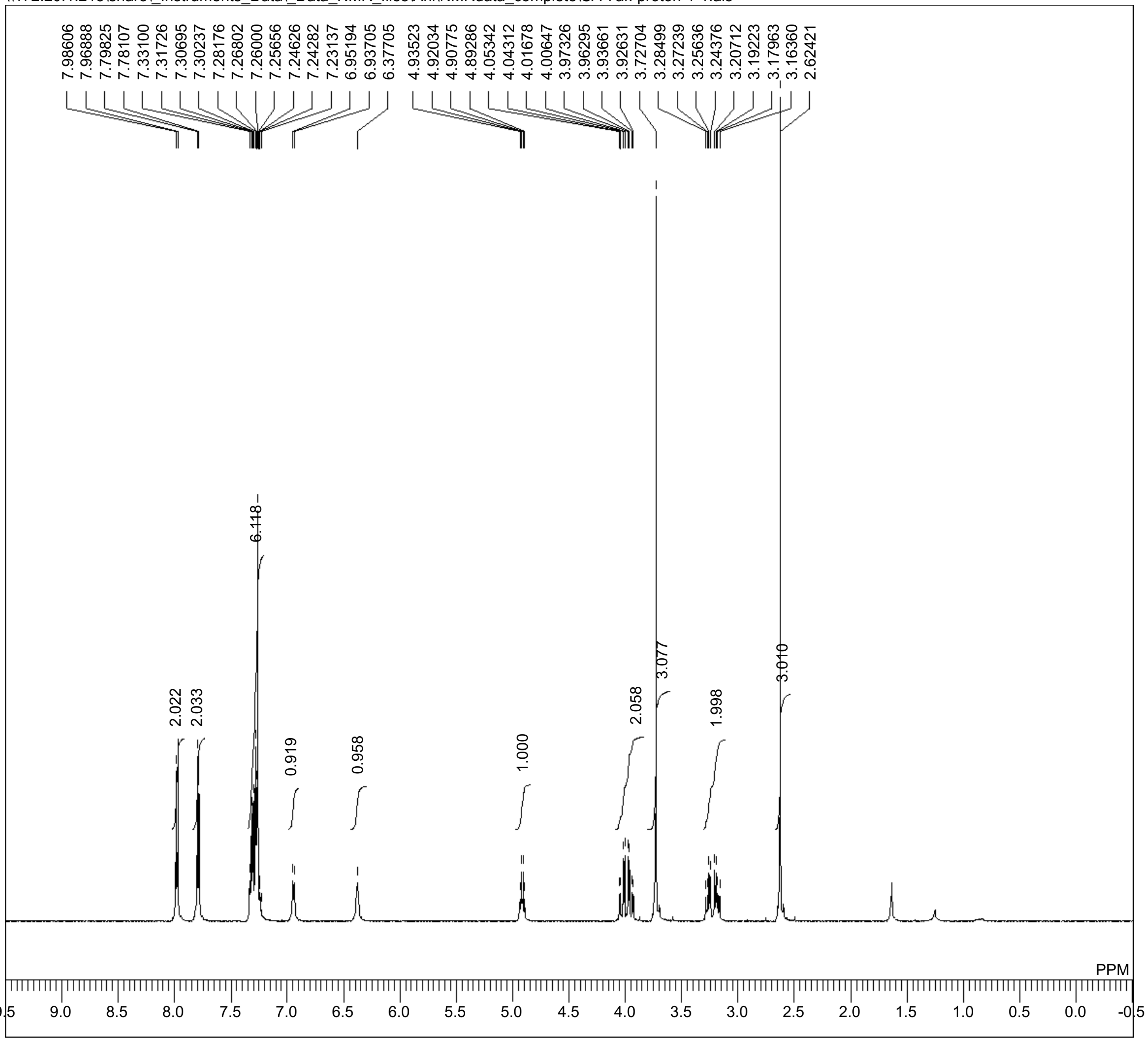

DFILE	SA-7ak-proton-1-1.als
COMNT	
DATIM	2025-02-01 03:46:27
OBNUC	1H
EXMOD	proton.jxp
OBFRQ	500.16 MHz
OBSET	2.41 KHz
OBFIN	6.01 Hz
POINT	13107
FREQU	7507.51 Hz
SCANS	8
ACQTM	1.7459 sec
PD	5.0000 sec
PW1	5.55 usec
IRNUC	1H
CTEMP	21.4 c
SLVNT	CDCL3
EXREF	7.26 ppm
BF	1.02 Hz
RGAIN	30

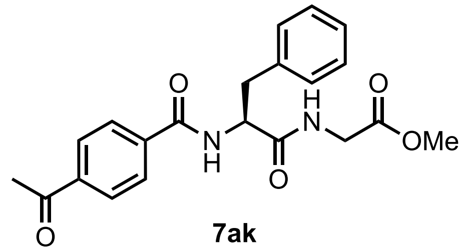

7ak

\\172.20.1.218\share_Instruments_Data_Data_NMR_files\Arii\NMRdata_complete\SA7ak-carbon-1-1.als

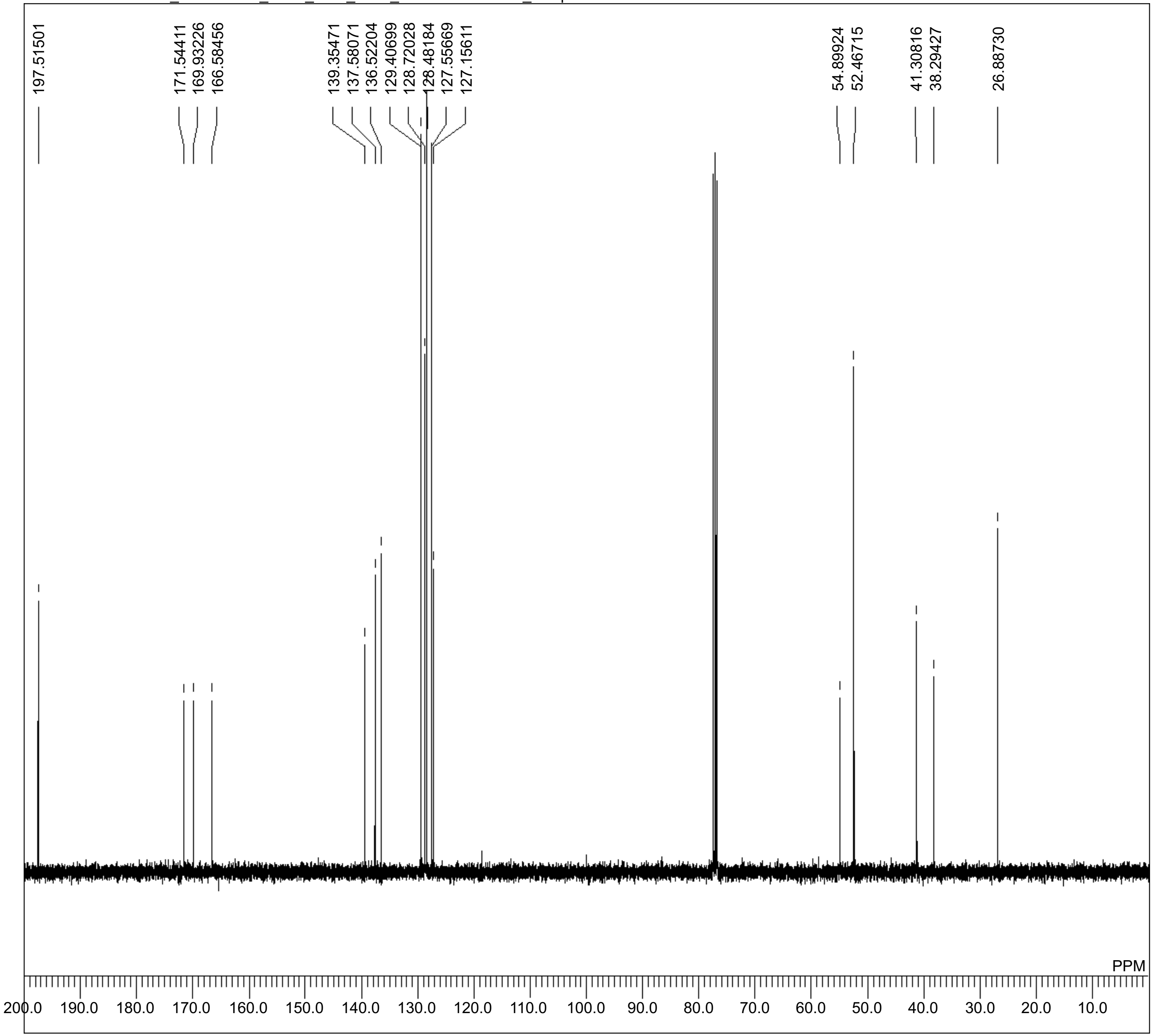

DFILE	SA7ak-carbon-1-1.als
COMNT	
DATIM	2025-01-17 22:06:16
OBNUC	13C
EXMOD	carbon.jxp
OBFRQ	98.52 MHz
OBSET	4.64 KHz
OBFIN	8.74 Hz
POINT	26214
FREQU	24630.54 Hz
SCANS	301
ACQTM	1.0643 sec
PD	2.0000 sec
PW1	2.93 usec
IRNUC	1H
CTEMP	20.5 c
SLVNT	CDCL3
EXREF	77.16 ppm
BF	1.02 Hz
RGAIN	60

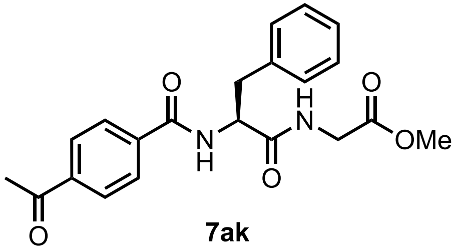

7ak

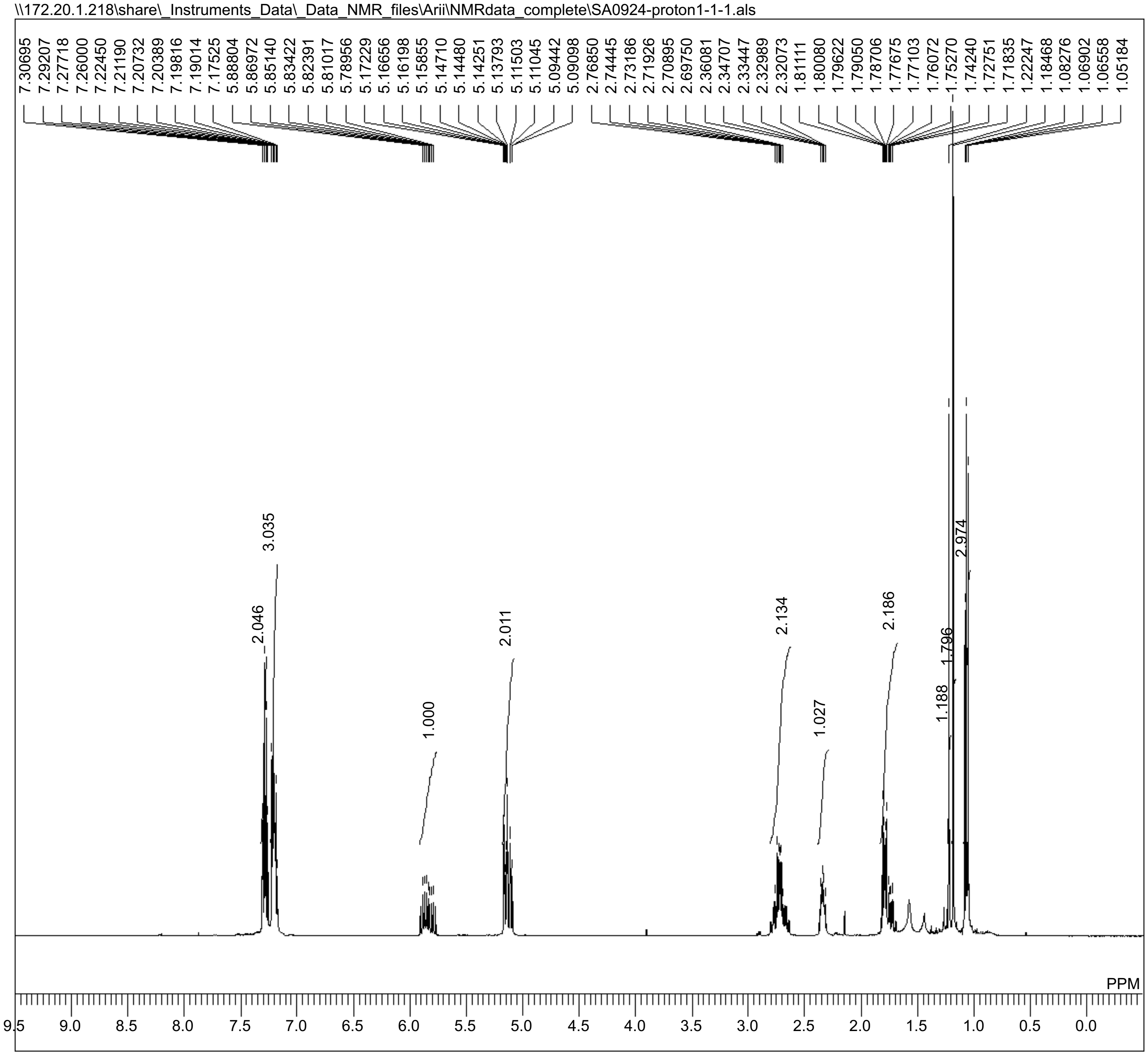

DFILE	SA0924-proton1-1-1.als
COMNT	
DATIM	2024-12-06 12:57:14
OBNUC	1H
EXMOD	proton.jxp
OBFRQ	500.16 MHz
OBSET	2.41 KHz
OBFIN	6.01 Hz
POINT	13107
FREQU	7507.51 Hz
SCANS	8
ACQTM	1.7459 sec
PD	5.0000 sec
PW1	5.55 usec
IRNUC	1H
CTEMP	21.7 c
SLVNT	CDCL3
EXREF	7.26 ppm
BF	1.02 Hz
RGAIN	28

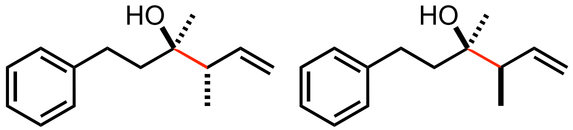

major *minor*

8a

\\172.20.1.218\share_Instruments_Data_Data_NMR_files\Arii\NMRdata_complete\SA0924-carbon-1-1.als

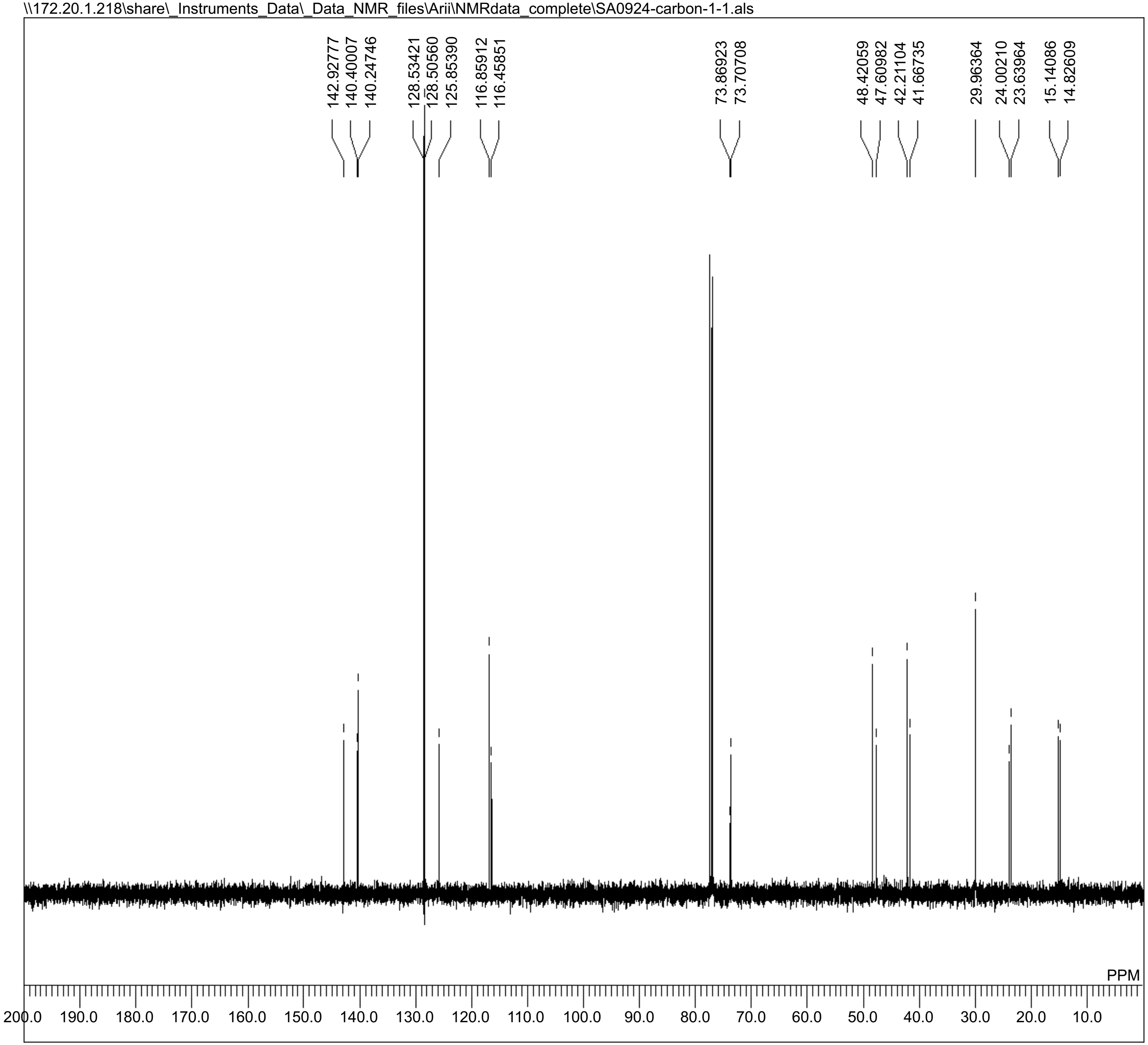

DFILE	SA0924-carbon-1-1.als
COMNT	
DATIM	2024-12-06 12:59:00
OBNUC	13C
EXMOD	carbon.jxp
OBFRQ	125.77 MHz
OBSET	7.87 KHz
OBFIN	4.21 Hz
POINT	26214
FREQU	31446.54 Hz
SCANS	241
ACQTM	0.8336 sec
PD	1.0000 sec
PW1	3.40 usec
IRNUC	1H
CTEMP	22.0 c
SLVNT	CDCL3
EXREF	77.16 ppm
BF	0.12 Hz
RGAIN	60

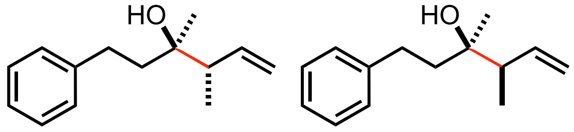

major *minor*

8a

\\172.20.1.218\share_Instruments_Data_Data_NMR_files\Arii\NMRdata_complete\SA1010-proton-1-1.als

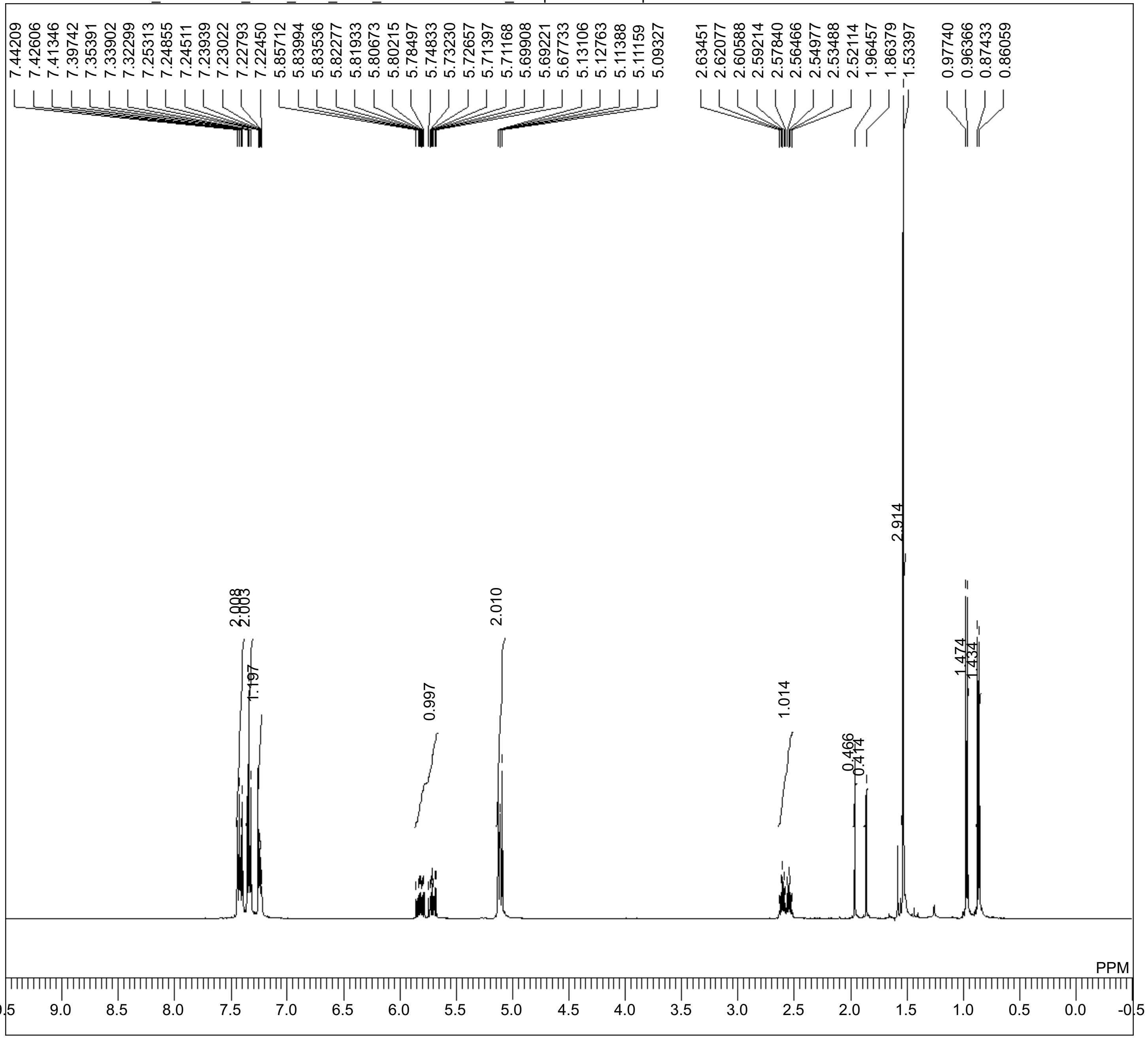

DFILE	SA1010-proton-1-1.als
COMNT	
DATIM	2024-12-19 12:59:43
OBNUC	1H
EXMOD	proton.jxp
OBFRQ	500.16 MHz
OBSET	2.41 KHz
OBFIN	6.01 Hz
POINT	13107
FREQU	7507.51 Hz
SCANS	8
ACQTM	1.7459 sec
PD	5.0000 sec
PW1	5.55 usec
IRNUC	1H
CTEMP	21.4 c
SLVNT	CDCL3
EXREF	7.26 ppm
BF	0.12 Hz
RGAIN	30

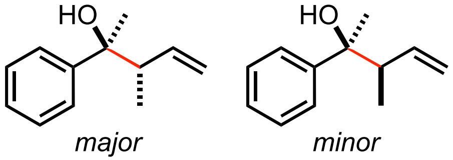

8b

\\172.20.1.218\share_Instruments_Data_Data_NMR_files\Arii\NMRdata_complete\SA1010-carbon-1-1.als

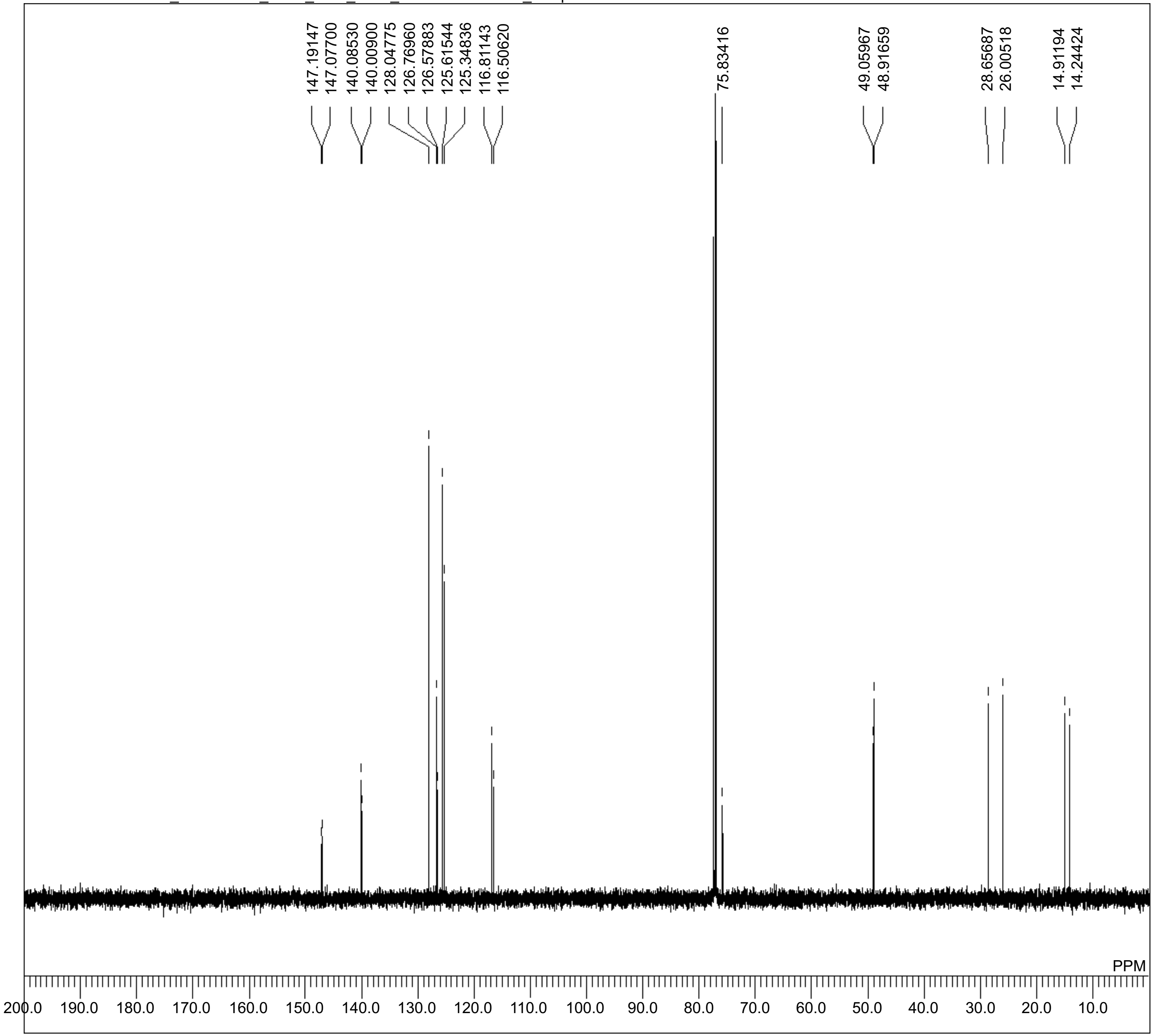

DFILE	SA1010-carbon-1-1.als
COMNT	
DATIM	2024-12-19 13:01:48
OBNUC	13C
EXMOD	carbon.jxp
OBFRQ	125.77 MHz
OBSET	7.87 KHz
OBFIN	4.21 Hz
POINT	26214
FREQU	31446.54 Hz
SCANS	336
ACQTM	0.8336 sec
PD	1.0000 sec
PW1	3.40 usec
IRNUC	1H
CTEMP	21.8 c
SLVNT	CDCL3
EXREF	77.16 ppm
BF	0.42 Hz
RGAIN	60

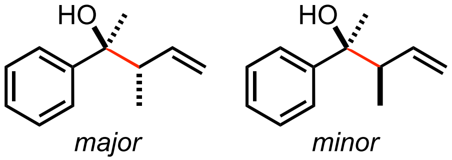

8b

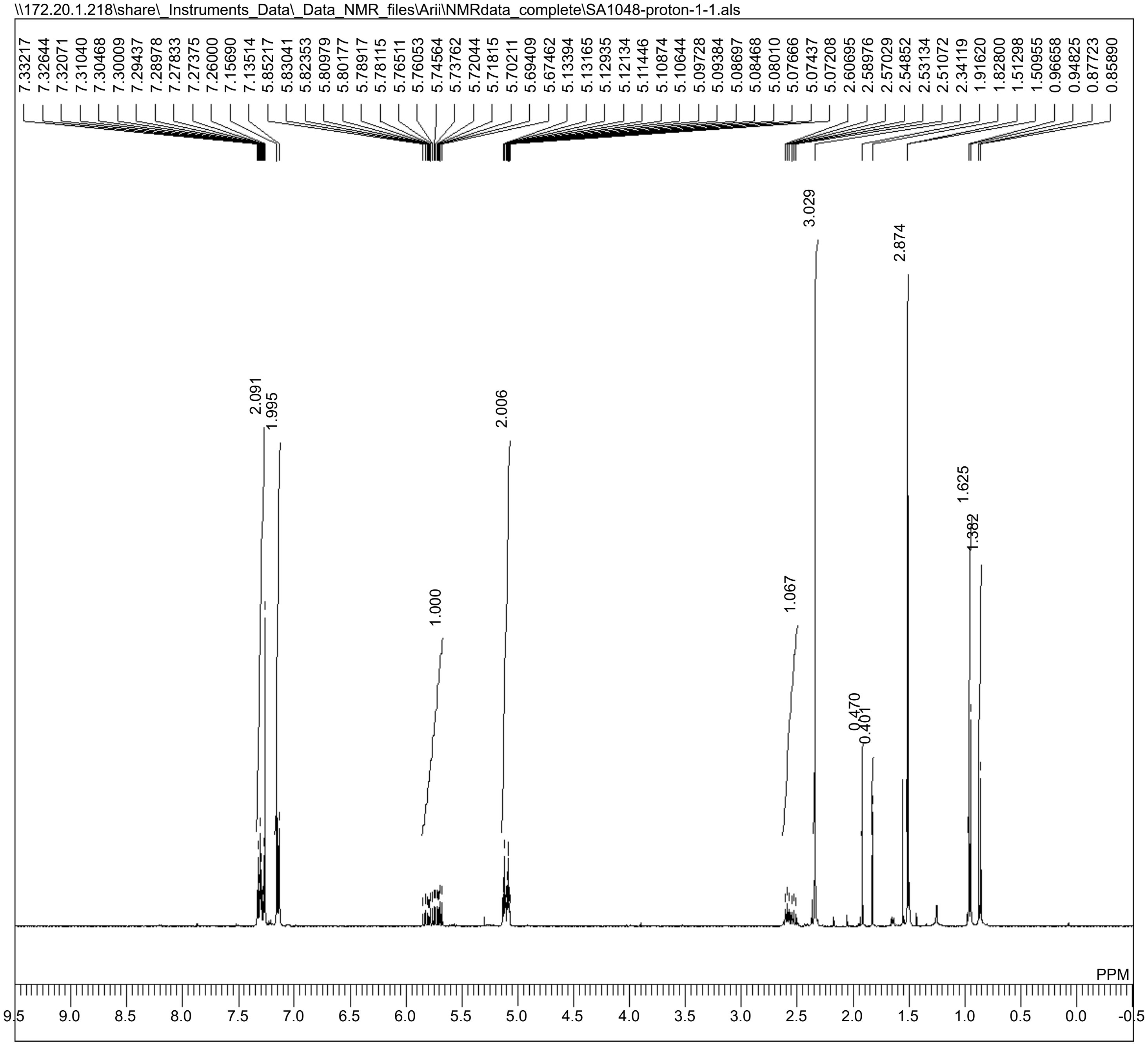

DFILE	SA1048-proton-1-1.als
COMNT	
DATIM	2025-01-19 02:22:42
OBNUC	1H
EXMOD	proton.jxp
OBFRQ	391.78 MHz
OBSET	8.51 KHz
OBFIN	3.34 Hz
POINT	13107
FREQU	5882.35 Hz
SCANS	8
ACQTM	2.2282 sec
PD	4.0000 sec
PW1	6.30 usec
IRNUC	1H
CTEMP	20.2 c
SLVNT	CDCL3
EXREF	7.26 ppm
BF	1.02 Hz
RGAIN	44

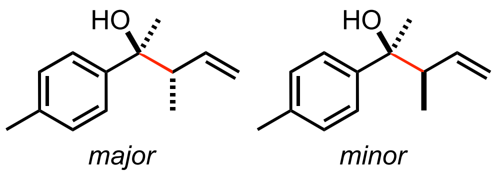

8c

\\172.20.1.218\share_Instruments_Data_Data_NMR_files\Arii\NMRdata_complete\SA1048-carbon-1-1.als

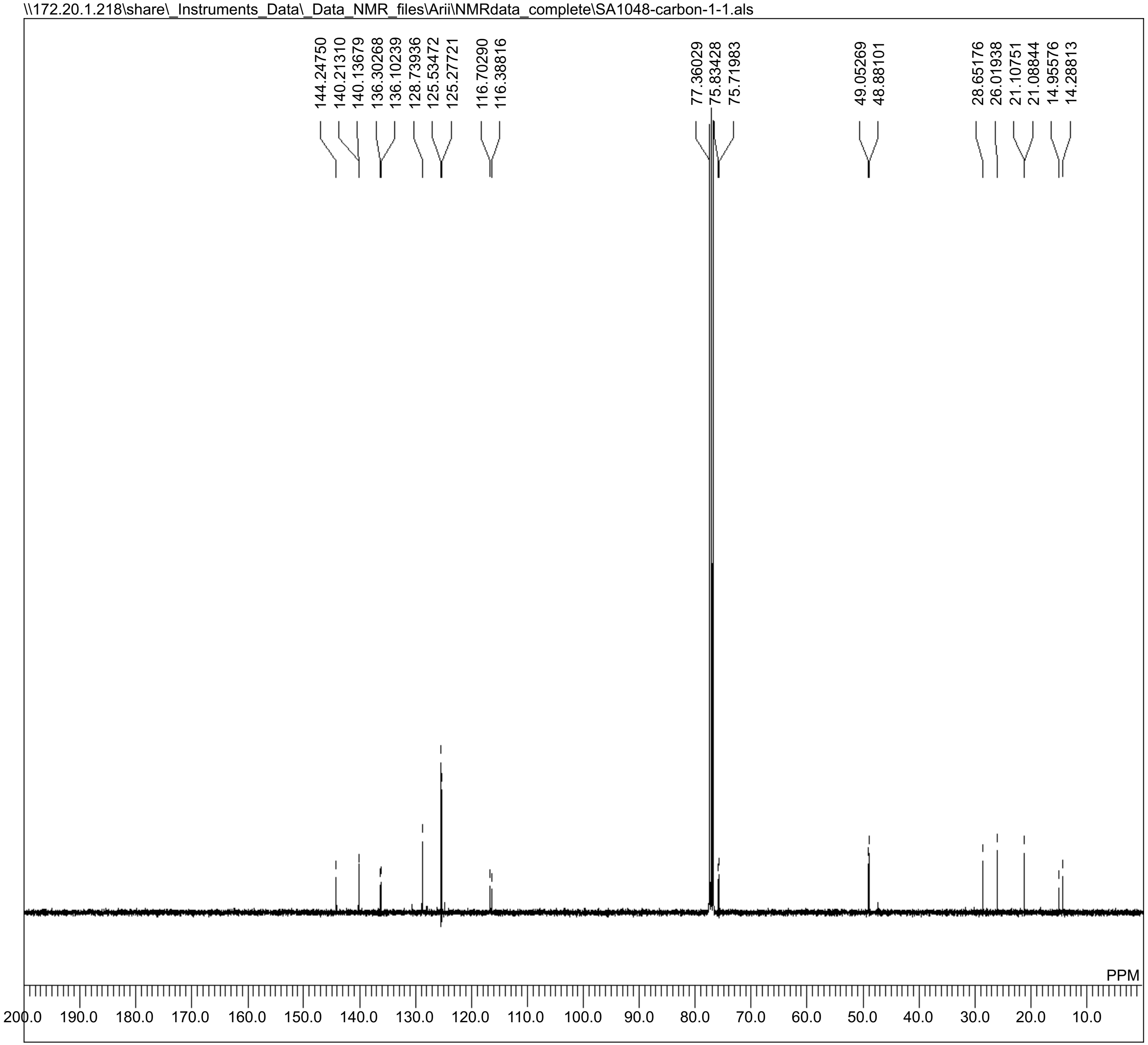

DFILE	SA1048-carbon-1-1.als
COMNT	
DATIM	2025-01-19 02:24:20
OBNUC	13C
EXMOD	carbon.jxp
OBFRQ	98.52 MHz
OBSET	4.64 KHz
OBFIN	8.74 Hz
POINT	26214
FREQU	24630.54 Hz
SCANS	5187
ACQTM	1.0643 sec
PD	2.0000 sec
PW1	2.93 usec
IRNUC	1H
CTEMP	20.4 c
SLVNT	CDCL3
EXREF	77.16 ppm
BF	1.02 Hz
RGAIN	60

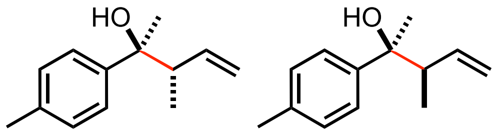

major *minor*

8c

\\172.20.1.218\share_Instruments_Data_Data_NMR_files\Arii\NMRdata_complete\SA0918-proton-1-1.als

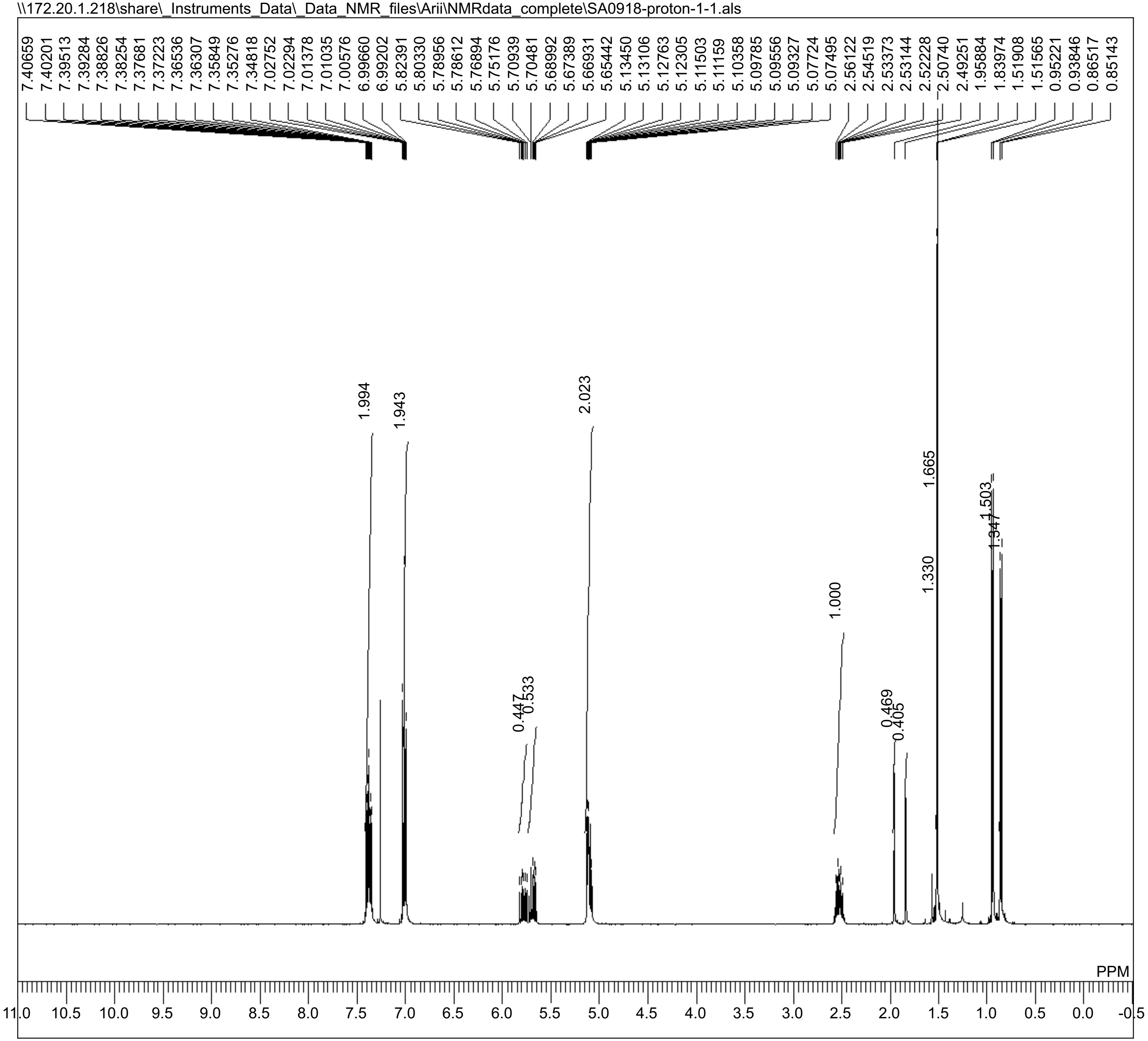

DFILE	SA0918-proton-1-1.als
COMNT	
DATIM	2025-01-16 13:18:40
OBNUC	1H
EXMOD	proton.jxp
OBFRQ	500.16 MHz
OBSET	2.41 KHz
OBFIN	6.01 Hz
POINT	13107
FREQU	7507.51 Hz
SCANS	8
ACQTM	1.7459 sec
PD	5.0000 sec
PW1	5.55 usec
IRNUC	1H
CTEMP	21.2 c
SLVNT	CDCL3
EXREF	7.26 ppm
BF	0.12 Hz
RGAIN	30

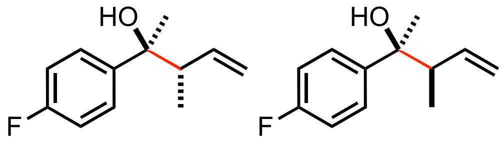

major *minor*

8d

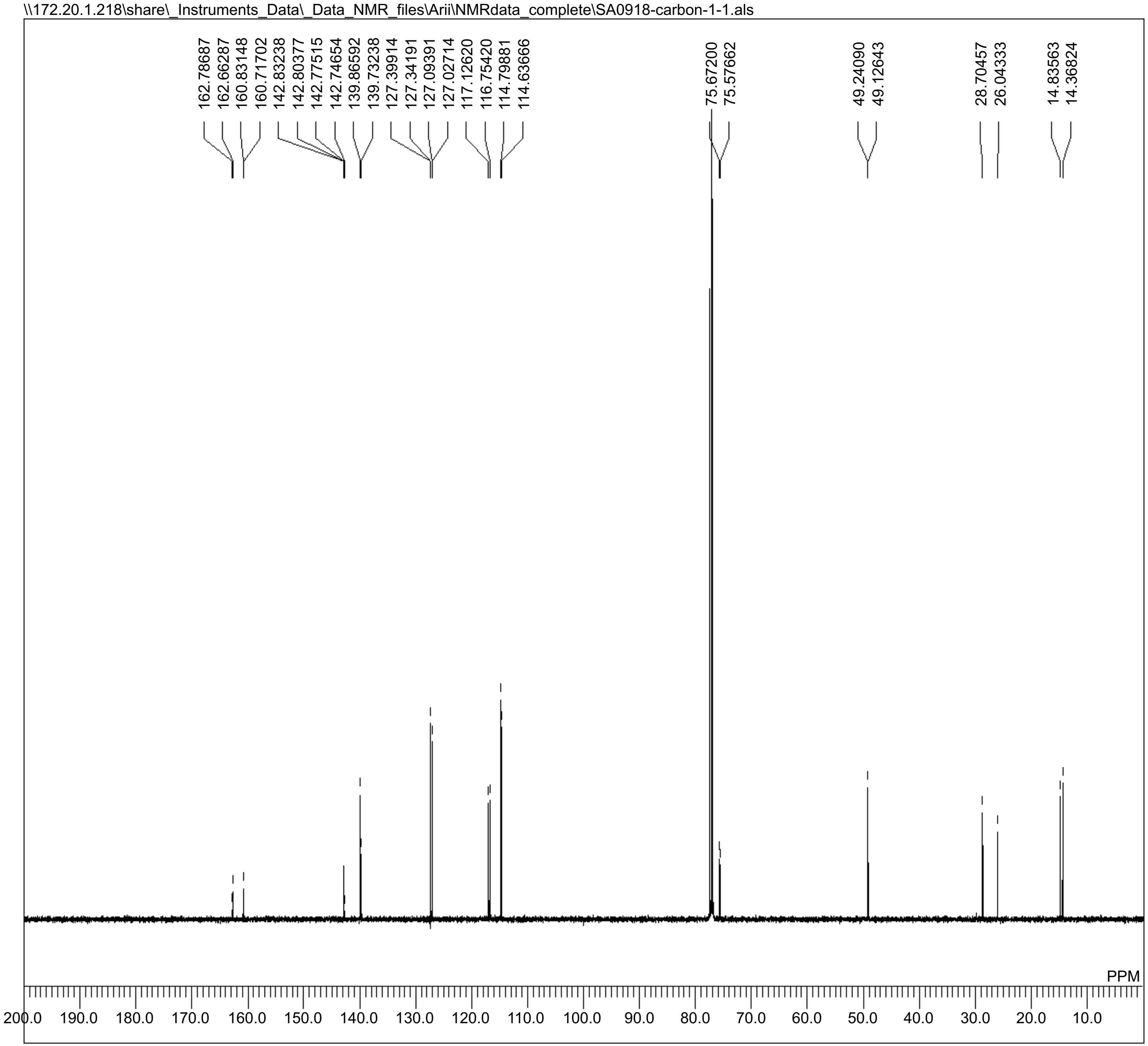

DFILE	SA0918-carbon-1-1.als
COMNT	
DATIM	2025-01-16 17:43:01
OBNUC	13C
EXMOD	carbon.jxp
OBFRQ	125.77 MHz
OBSET	7.87 KHz
OBFIN	4.21 Hz
POINT	26214
FREQU	31446.54 Hz
SCANS	6214
ACQTM	0.8336 sec
PD	1.0000 sec
PW1	3.40 usec
IRNUC	1H
CTEMP	21.9 c
SLVNT	CDCL3
EXREF	77.16 ppm
BF	0.12 Hz
RGAIN	60

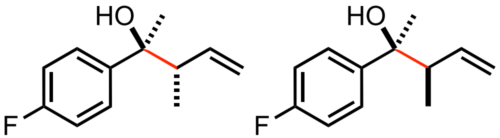

major *minor*

8d

\\172.20.1.218\share_Instruments_Data_Data_NMR_files\Arii\NMRdata_complete\SA0918-fluorine-1-4.als

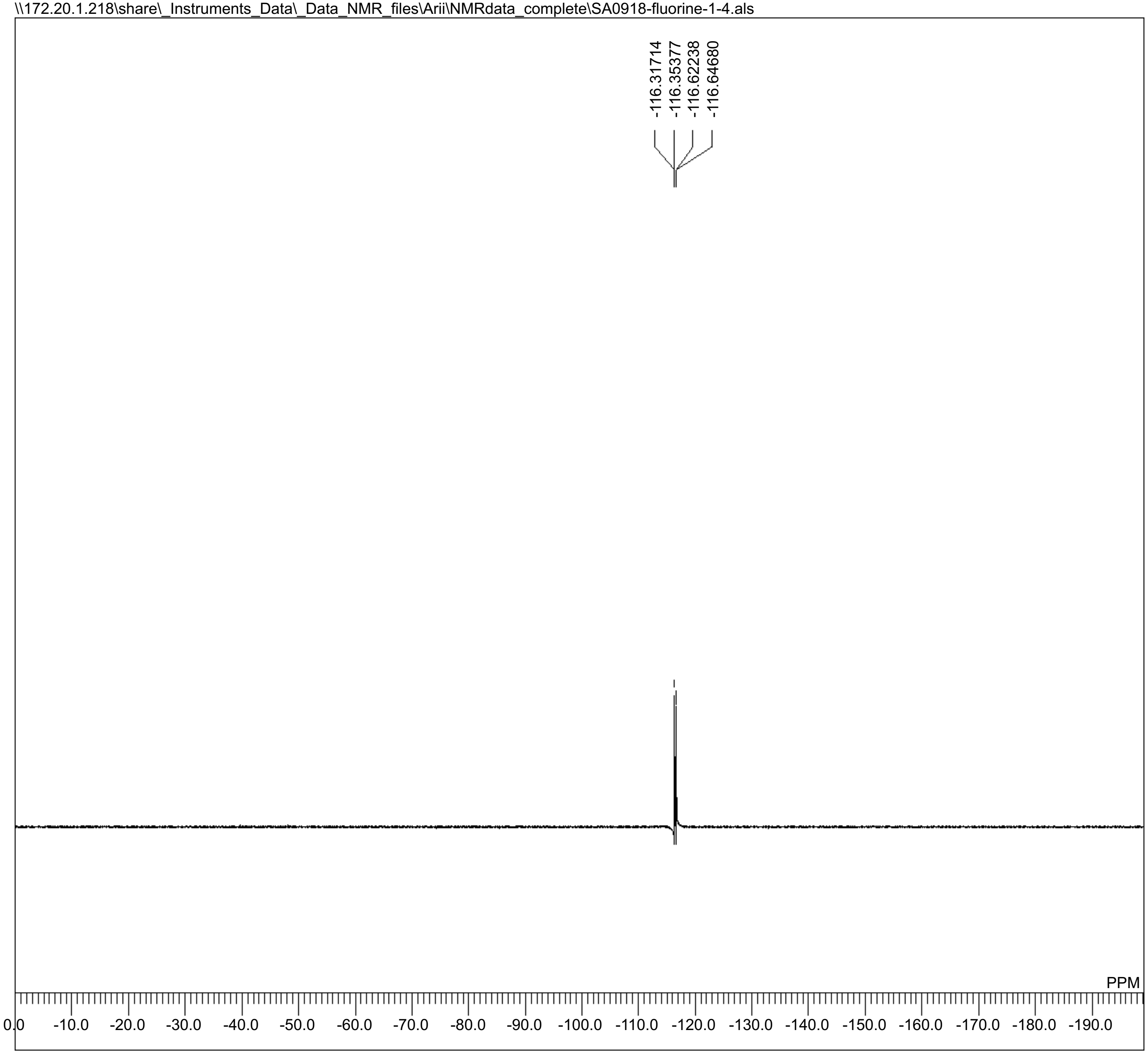

DFILE	SA0918-fluorine-1-4.als
COMNT	
DATIM	2025-01-16 22:19:13
OBNUC	19F
EXMOD	single_pulse.jxp
OBFRQ	368.64 MHz
OBSET	7.63 KHz
OBFIN	2.85 Hz
POINT	32768
FREQU	147492.62 Hz
SCANS	8
ACQTM	0.2222 sec
PD	5.0000 sec
PW1	4.10 usec
IRNUC	19F
CTEMP	20.4 c
SLVNT	CDCL3
EXREF	-164.90 ppm
BF	1.02 Hz
RGAIN	50

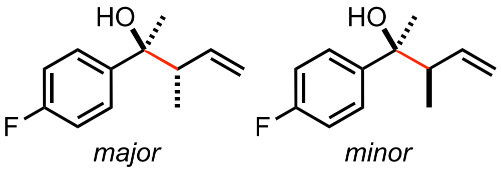

8d

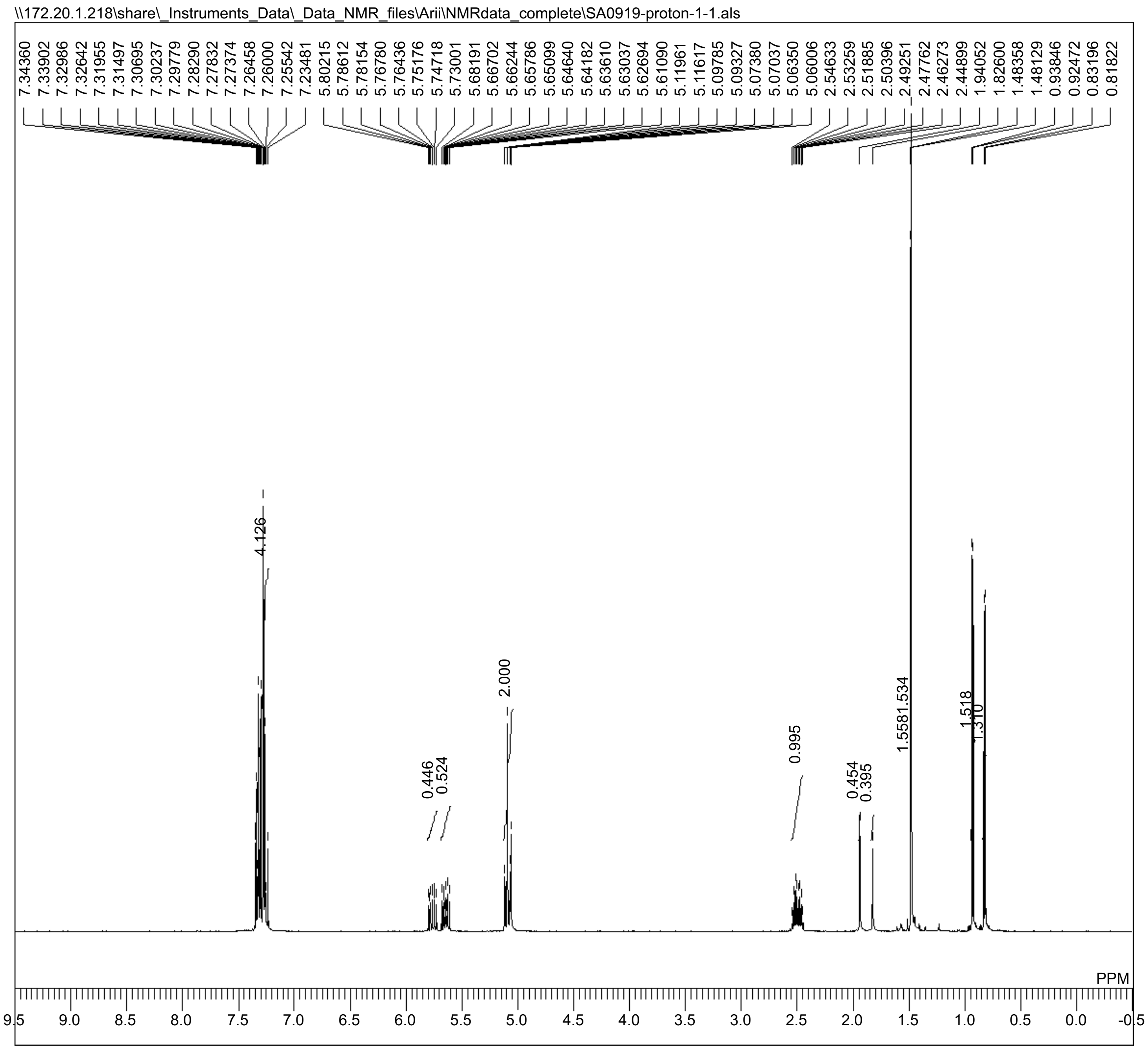

DFILE	SA0919-proton-1-1.als
COMNT	
DATIM	2025-01-16 11:54:26
OBNUC	1H
EXMOD	proton.jxp
OBFRQ	500.16 MHz
OBSET	2.41 KHz
OBFIN	6.01 Hz
POINT	13107
FREQU	7507.51 Hz
SCANS	8
ACQTM	1.7459 sec
PD	5.0000 sec
PW1	5.55 usec
IRNUC	1H
CTEMP	21.1 c
SLVNT	CDCL3
EXREF	7.26 ppm
BF	0.12 Hz
RGAIN	30

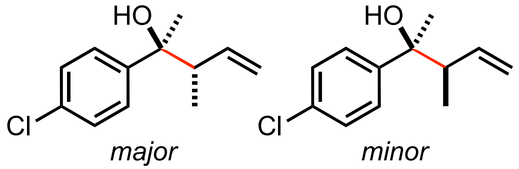

8e

\\172.20.1.218\share_Instruments_Data_Data_NMR_files\Arii\NMRdata_complete\SA0919-carbon-1-1.als

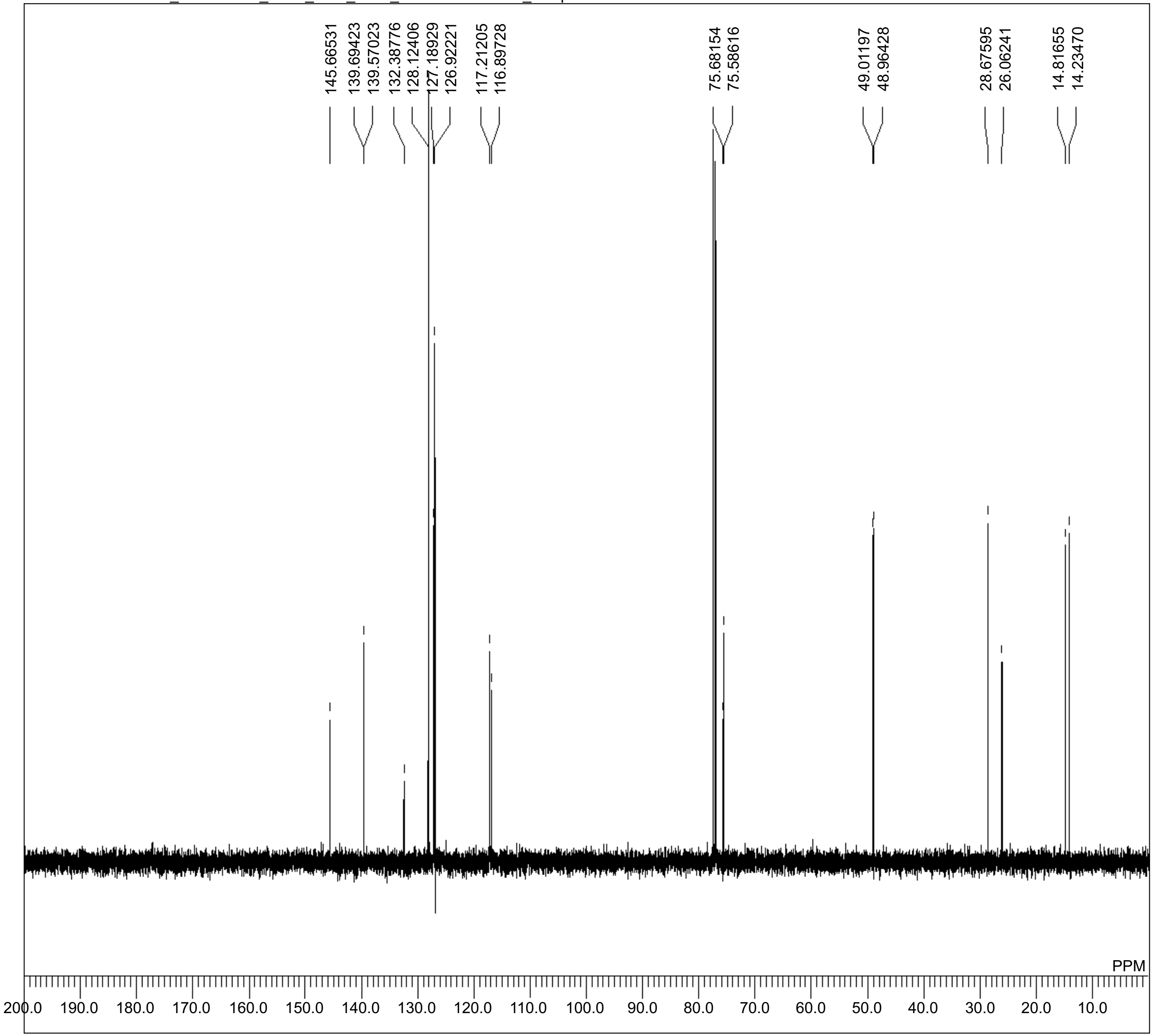

DFILE	SA0919-carbon-1-1.als
COMNT	
DATIM	2025-01-16 11:56:14
OBNUC	13C
EXMOD	carbon.jxp
OBFRQ	125.77 MHz
OBSET	7.87 KHz
OBFIN	4.21 Hz
POINT	26214
FREQU	31446.54 Hz
SCANS	245
ACQTM	0.8336 sec
PD	1.0000 sec
PW1	3.40 usec
IRNUC	1H
CTEMP	21.8 c
SLVNT	CDCL3
EXREF	77.16 ppm
BF	0.12 Hz
RGAIN	60

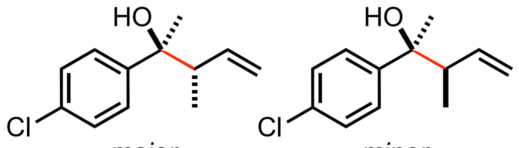

major *minor*

8e

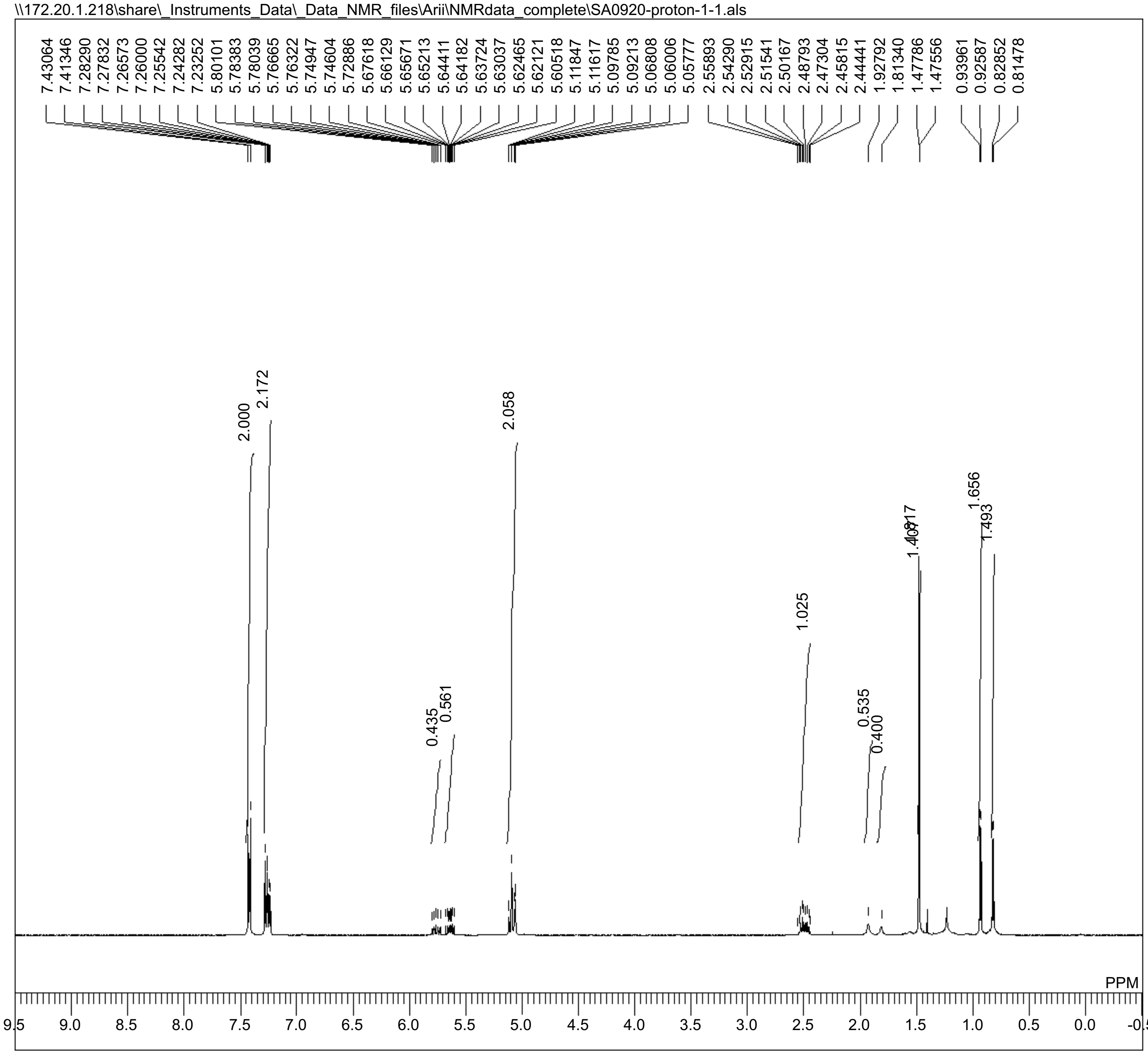

DFILE	SA0920-proton-1-1.als
COMNT	
DATIM	2024-12-13 02:11:32
OBNUC	1H
EXMOD	proton.jxp
OBFRQ	500.16 MHz
OBSET	2.41 KHz
OBFIN	6.01 Hz
POINT	13107
FREQU	7507.51 Hz
SCANS	8
ACQTM	1.7459 sec
PD	5.0000 sec
PW1	5.55 usec
IRNUC	1H
CTEMP	21.5 c
SLVNT	CDCL3
EXREF	7.26 ppm
BF	0.12 Hz
RGAIN	30

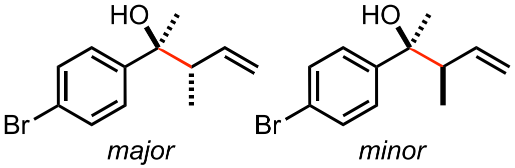

8f

\\172.20.1.218\share_Instruments_Data_Data_NMR_files\Arii\NMRdata_complete\SA0920-carbon-1-1.als

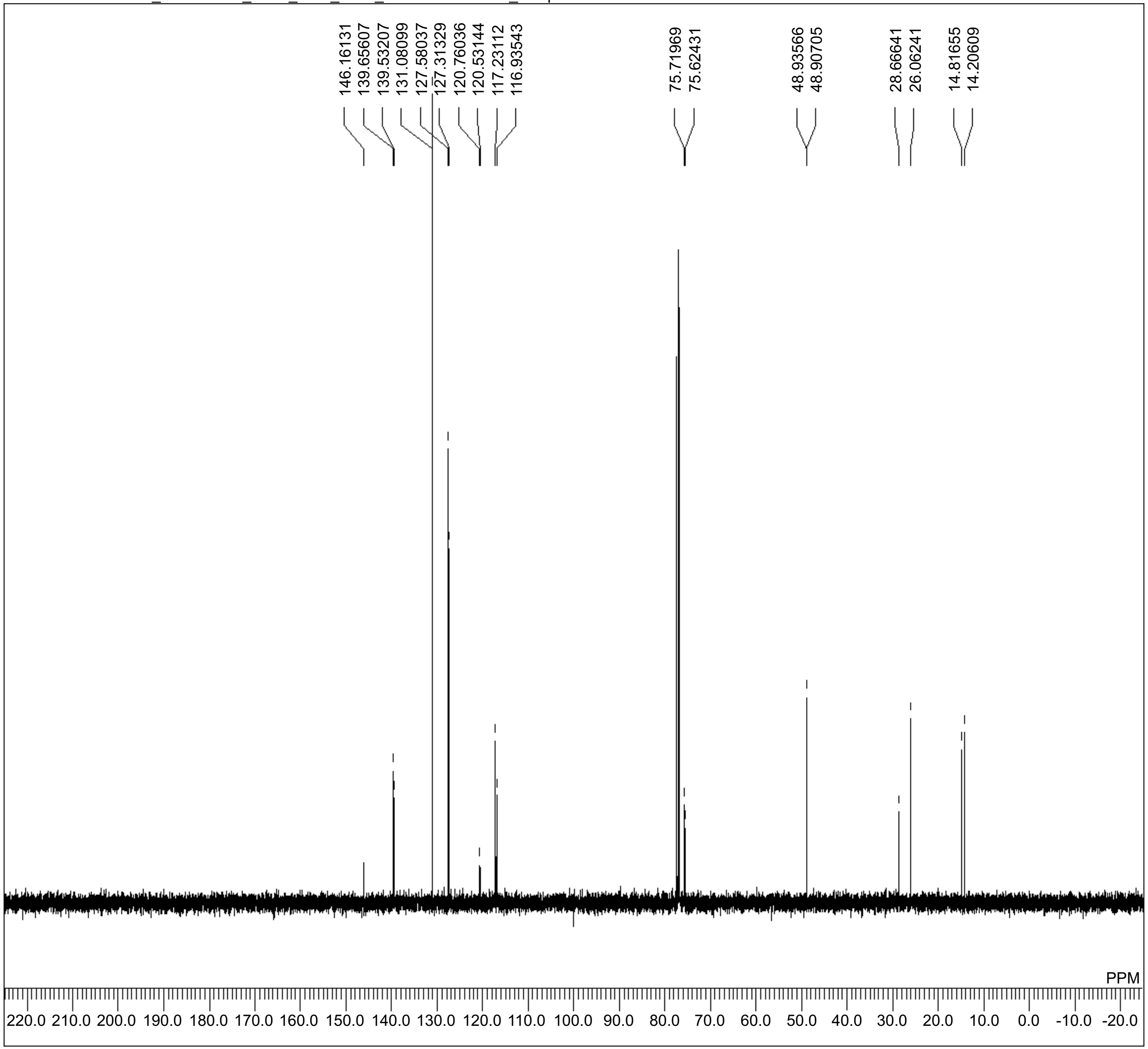

DFILE	SA0920-carbon-1-1.als
COMNT	
DATIM	2024-12-11 19:03:53
OBNUC	13C
EXMOD	carbon.jxp
OBFRQ	125.77 MHz
OBSET	7.87 KHz
OBFIN	4.21 Hz
POINT	26214
FREQU	31446.54 Hz
SCANS	210
ACQTM	0.8336 sec
PD	1.0000 sec
PW1	3.40 usec
IRNUC	1H
CTEMP	22.1 c
SLVNT	CDCL3
EXREF	77.16 ppm
BF	0.42 Hz
RGAIN	42

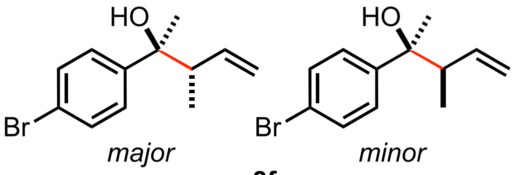

8f

\\172.20.1.218\share_Instruments_Data_Data_NMR_files\Arii\NMRdata_complete\SA0921-proton-1-1.als

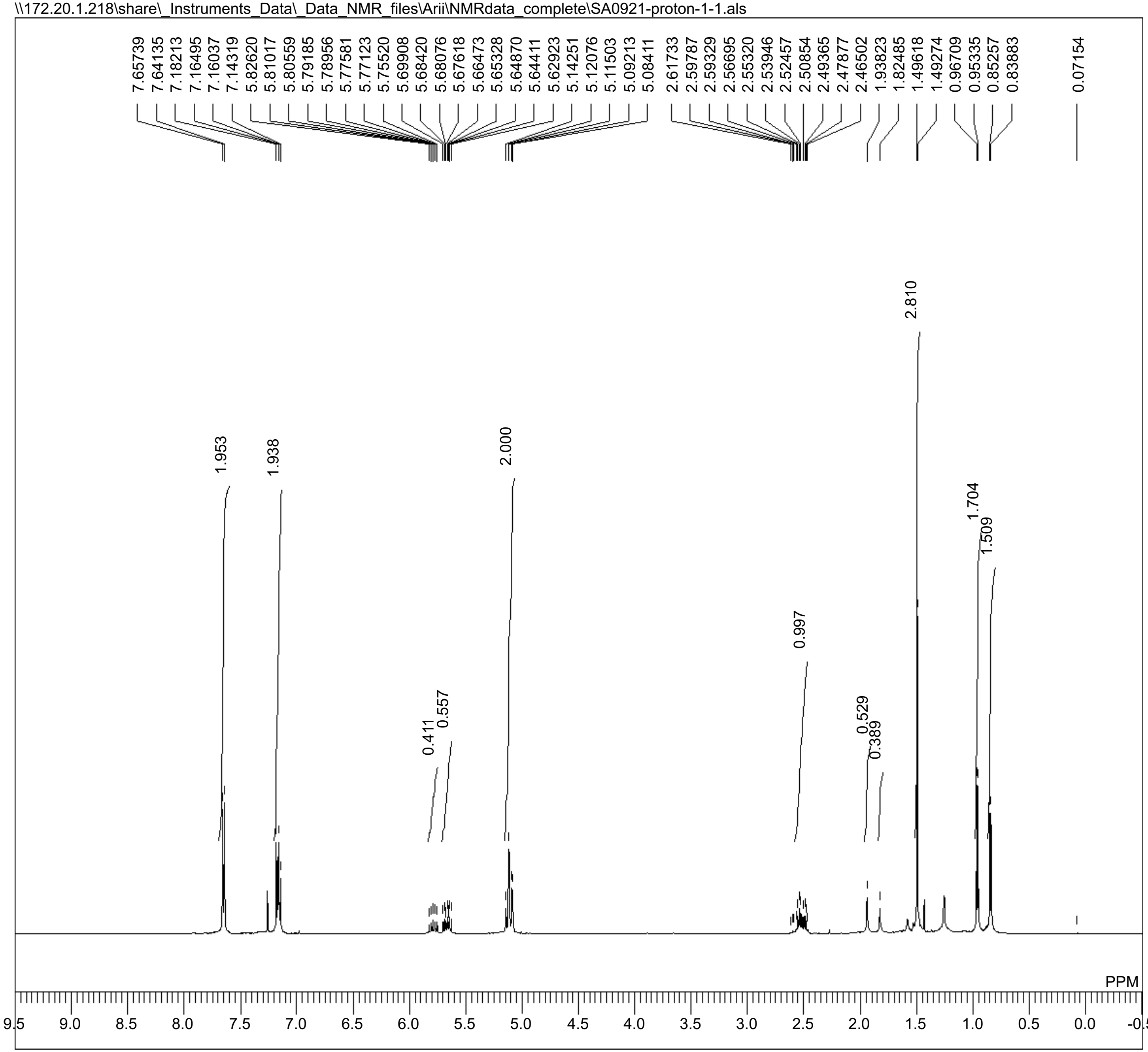

DFILE	SA0921-proton-1-1.als
COMNT	
DATIM	2024-12-16 18:14:59
OBNUC	1H
EXMOD	proton.jxp
OBFRQ	500.16 MHz
OBSET	2.41 KHz
OBFIN	6.01 Hz
POINT	13107
FREQU	7507.51 Hz
SCANS	8
ACQTM	1.7459 sec
PD	5.0000 sec
PW1	5.55 usec
IRNUC	1H
CTEMP	21.8 c
SLVNT	CDCL3
EXREF	7.26 ppm
BF	0.42 Hz
RGAIN	30

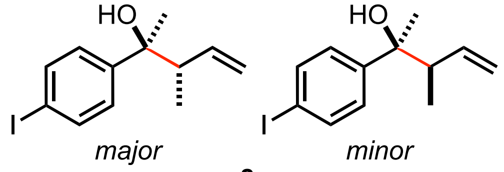

8g

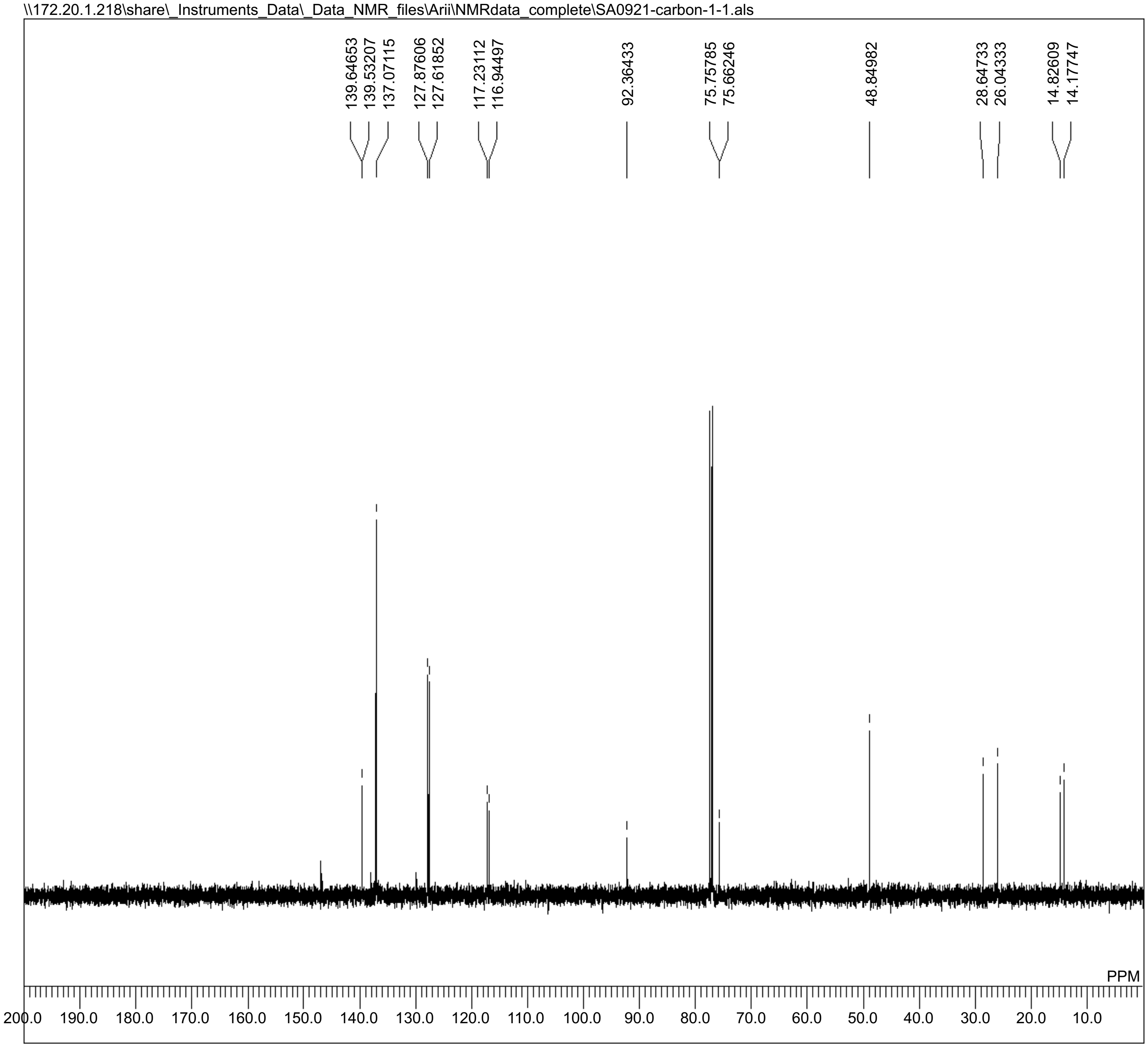

DFILE	SA0921-carbon-1-1.als
COMNT	
DATIM	2024-12-11 19:17:02
OBNUC	13C
EXMOD	carbon.jxp
OBFRQ	125.77 MHz
OBSET	7.87 KHz
OBFIN	4.21 Hz
POINT	26214
FREQU	31446.54 Hz
SCANS	115
ACQTM	0.8336 sec
PD	1.0000 sec
PW1	3.40 usec
IRNUC	1H
CTEMP	21.9 c
SLVNT	CDCL3
EXREF	77.16 ppm
BF	0.42 Hz
RGAIN	60

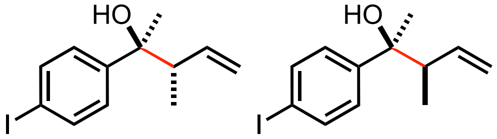

major *minor*

8g

\\172.20.1.218\share_Instruments_Data_Data_NMR_files\Arii\NMRdata_complete\SA1050-high-polar-proton-1-1.als

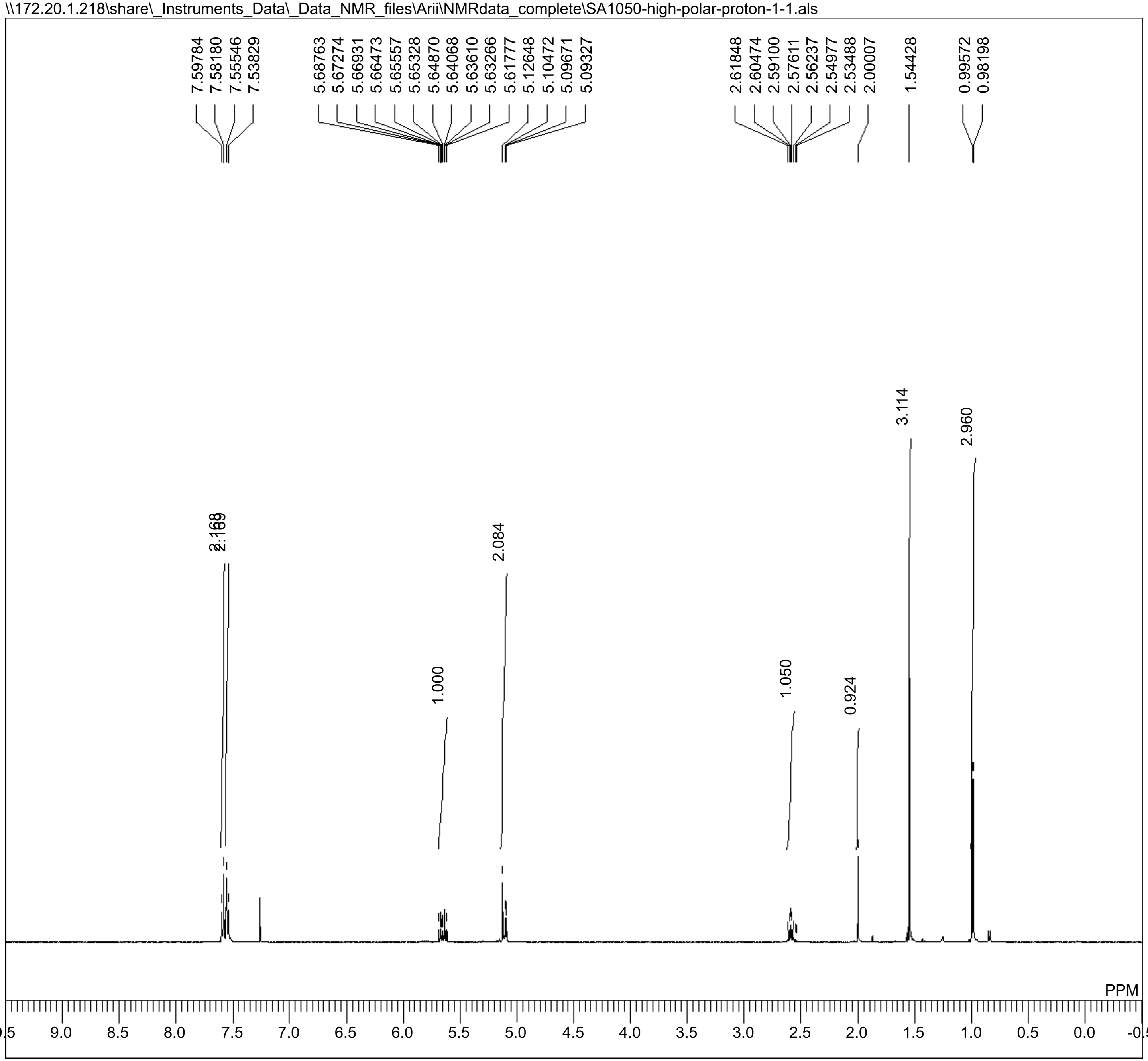

DFILE	SA1050-high-polar-proton-1-1.als
COMNT	
DATIM	2025-01-19 01:18:42
OBNUC	1H
EXMOD	proton.jxp
OBFRQ	500.16 MHz
OBSET	2.41 KHz
OBFIN	6.01 Hz
POINT	13107
FREQU	7507.51 Hz
SCANS	8
ACQTM	1.7459 sec
PD	5.0000 sec
PW1	5.55 usec
IRNUC	1H
CTEMP	21.2 c
SLVNT	CDCL3
EXREF	7.26 ppm
BF	0.42 Hz
RGAIN	30

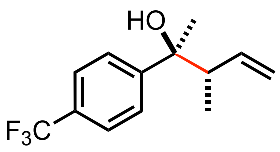

\\172.20.1.218\share_Instruments_Data_Data_NMR_files\Arii\NMRdata_complete\SA1050-high-polar-carbon-1-1.als

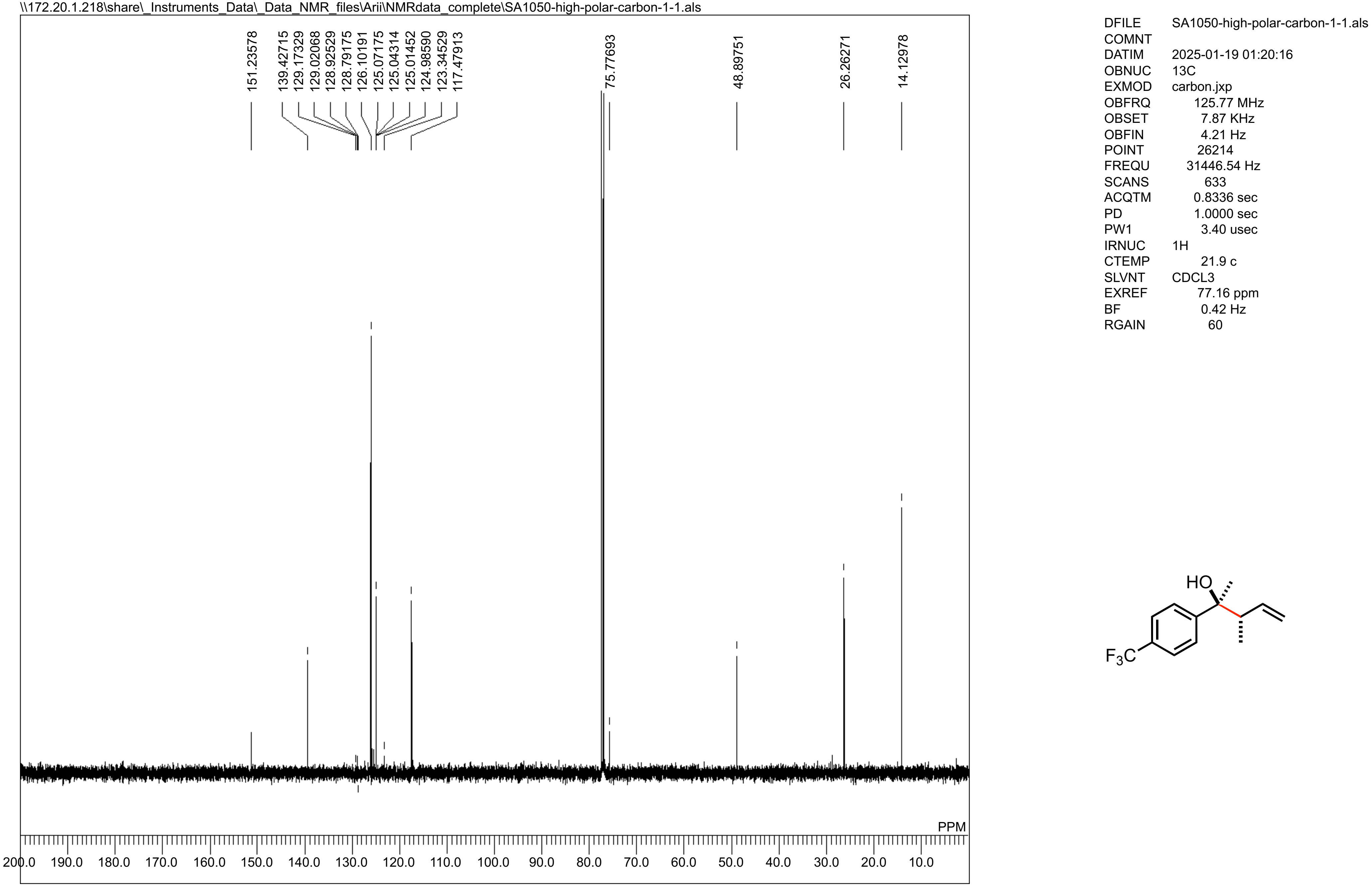

\\172.20.1.218\share_Instruments_Data_Data_NMR_files\Arii\NMRdata_complete\SA1050-high-polar-fluorine-1-1.als

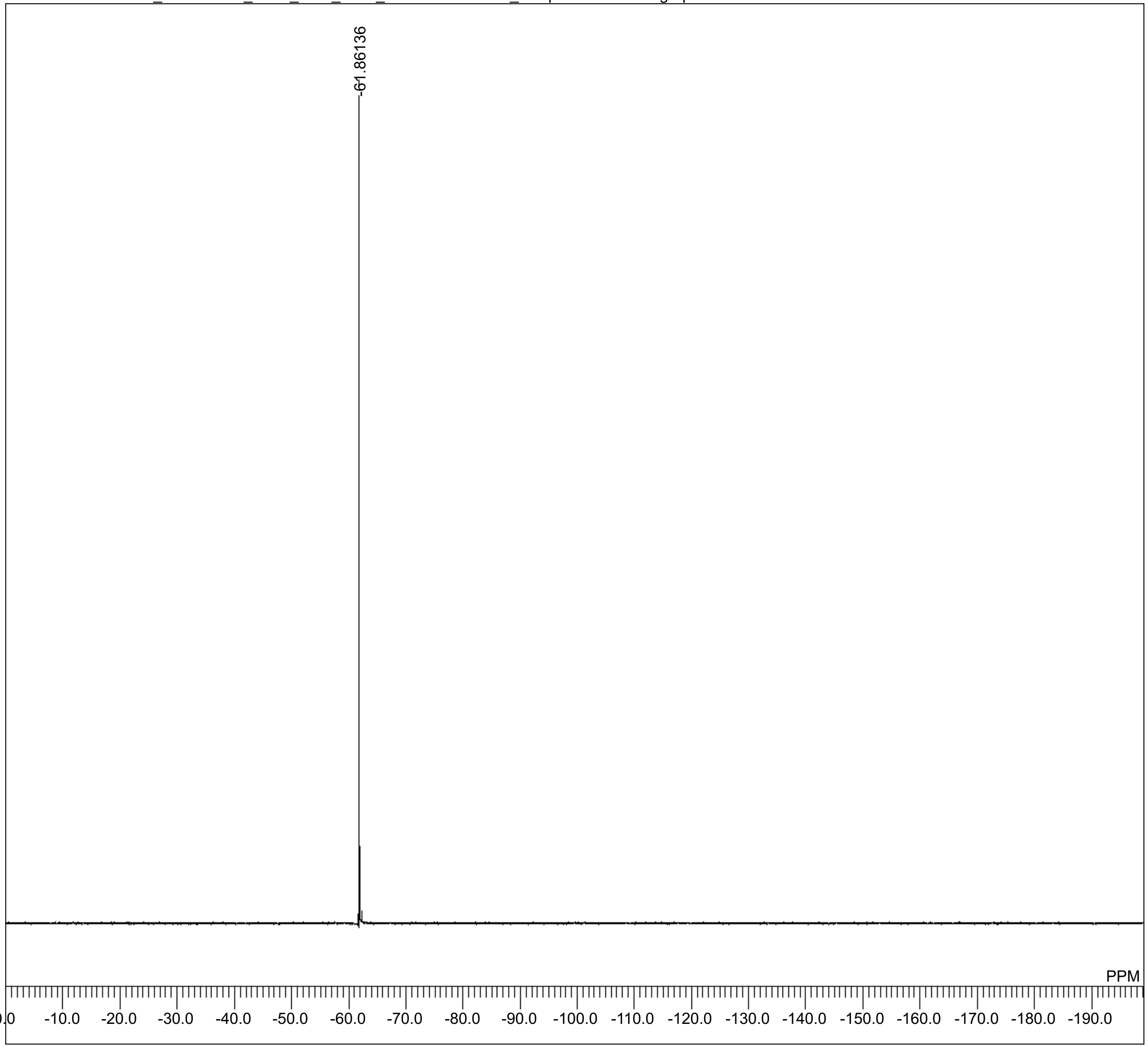

DFILE	SA1050-high-polar-fluorine-1-1.als
COMNT	
DATIM	2025-01-19 06:53:17
OBNUC	19F
EXMOD	single_pulse.jxp
OBFRQ	368.64 MHz
OBSET	7.63 KHz
OBFIN	2.85 Hz
POINT	32768
FREQU	147492.62 Hz
SCANS	8
ACQTM	0.2222 sec
PD	5.0000 sec
PW1	4.10 usec
IRNUC	19F
CTEMP	20.1 c
SLVNT	CDCL3
EXREF	-164.90 ppm
BF	1.02 Hz
RGAIN	52

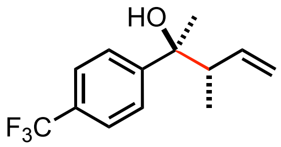

\\172.20.1.218\share_Instruments_Data_Data_NMR_files\Arii\NMRdata_complete\SA1050-low-polar-proton-1-1.als

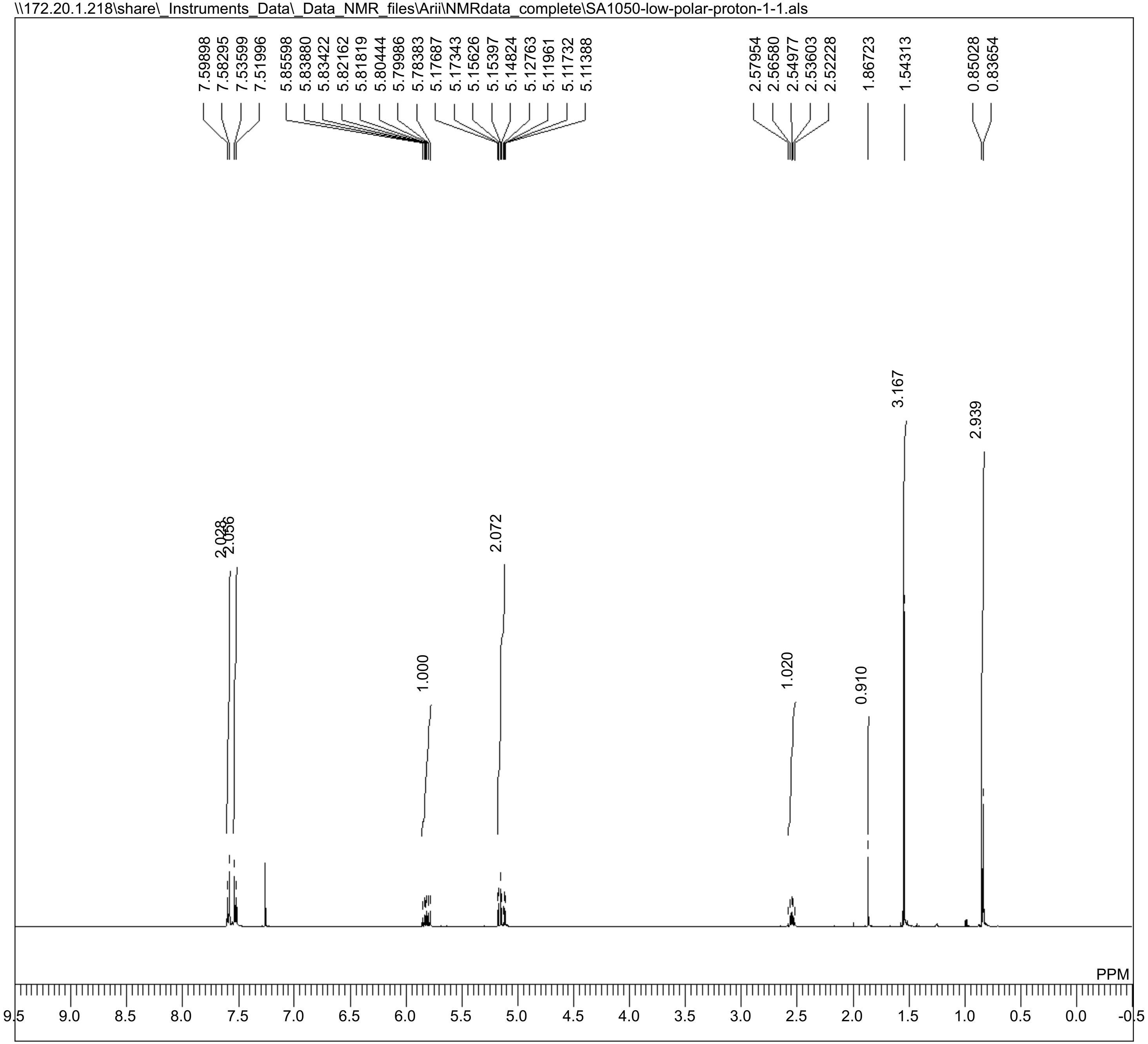

DFILE	SA1050-low-polar-proton-1-1.als
COMNT	
DATIM	2025-01-19 01:46:12
OBNUC	1H
EXMOD	proton.jxp
OBFRQ	500.16 MHz
OBSET	2.41 KHz
OBFIN	6.01 Hz
POINT	13107
FREQU	7507.51 Hz
SCANS	8
ACQTM	1.7459 sec
PD	5.0000 sec
PW1	5.55 usec
IRNUC	1H
CTEMP	20.8 c
SLVNT	CDCL3
EXREF	7.26 ppm
BF	0.42 Hz
RGAIN	30

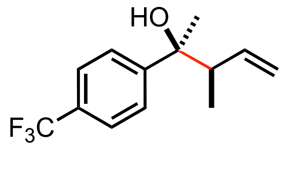

\\172.20.1.218\share_Instruments_Data_Data_NMR_files\Arii\NMRdata_complete\SA1050-low-polar-carbon-1-1.als

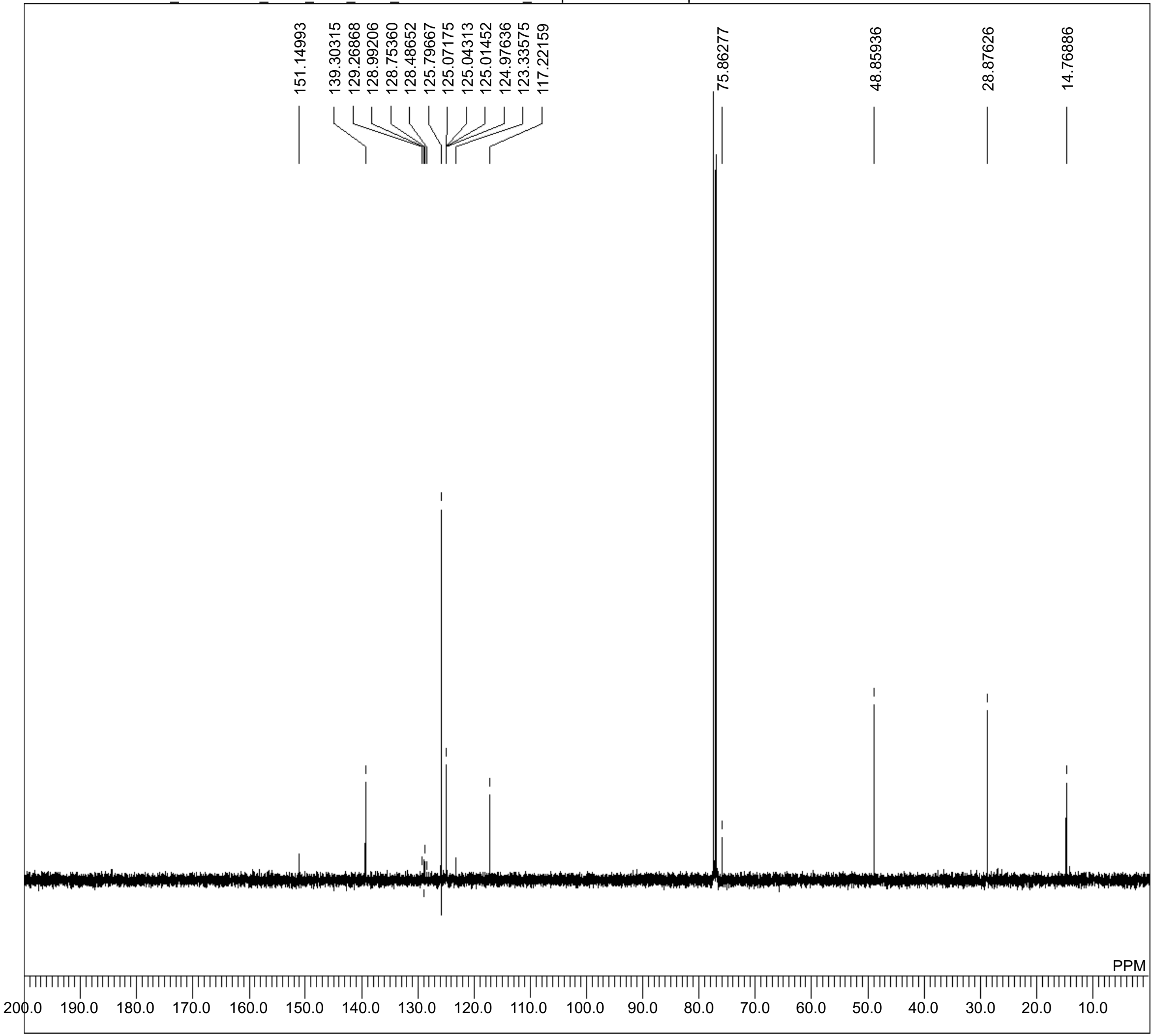

DFILE	SA1050-low-polar-carbon-1-1.als
COMNT	
DATIM	2025-01-19 01:47:44
OBNUC	13C
EXMOD	carbon.jxp
OBFRQ	125.77 MHz
OBSET	7.87 KHz
OBFIN	4.21 Hz
POINT	26214
FREQU	31446.54 Hz
SCANS	1000
ACQTM	0.8336 sec
PD	1.0000 sec
PW1	3.40 usec
IRNUC	1H
CTEMP	21.6 c
SLVNT	CDCL3
EXREF	77.16 ppm
BF	0.42 Hz
RGAIN	60

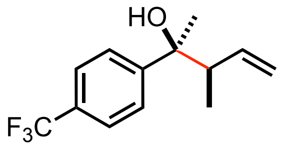

\\172.20.1.218\share_Instruments_Data_Data_NMR_files\Arii\NMRdata_complete\SA1050-low-polar-fluorine-1-1.als

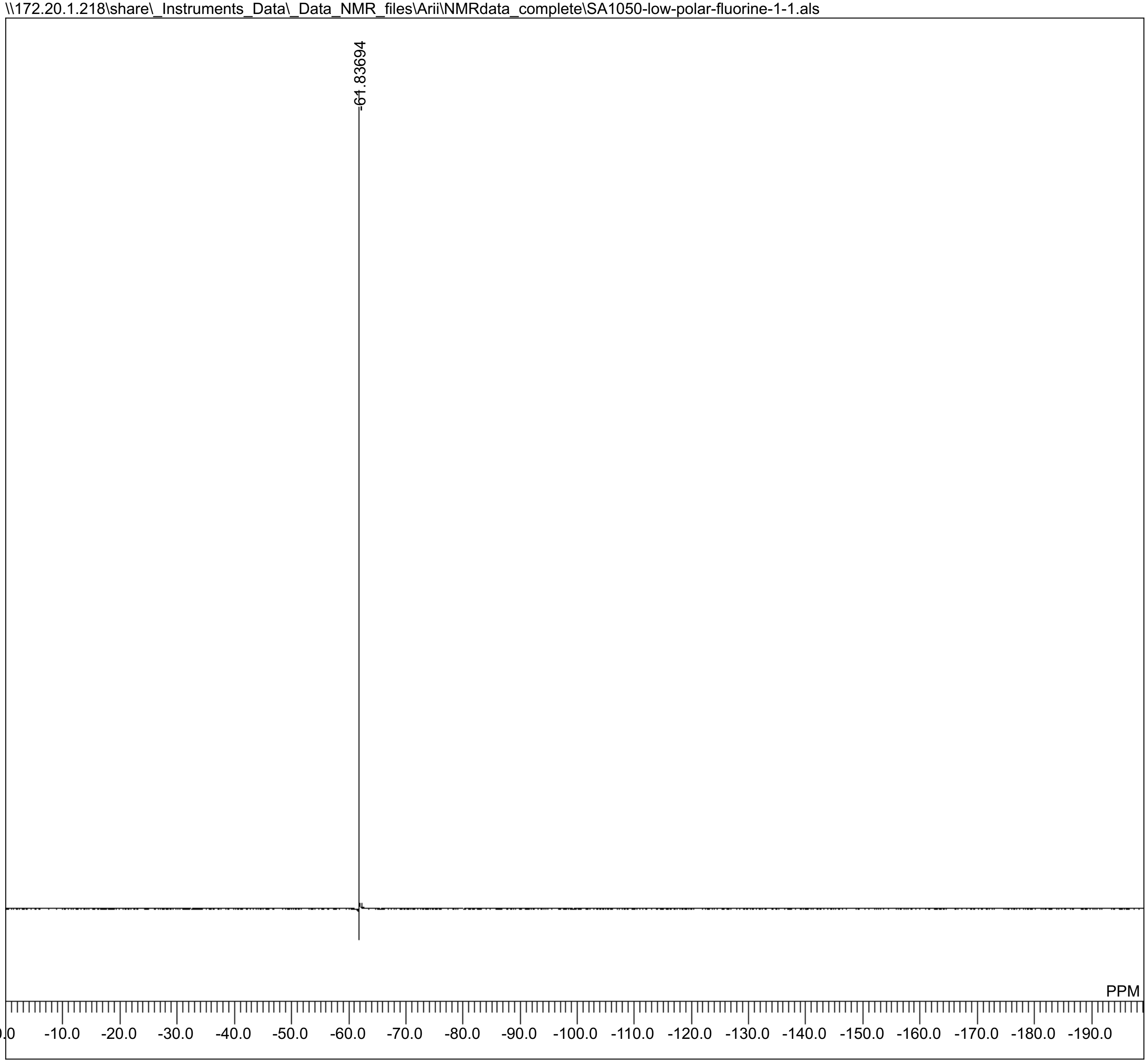

DFILE	SA1050-low-polar-fluorine-1-1.als
COMNT	
DATIM	2025-01-19 07:01:20
OBNUC	19F
EXMOD	single_pulse.jxp
OBFRQ	368.64 MHz
OBSET	7.63 KHz
OBFIN	2.85 Hz
POINT	32768
FREQU	147492.62 Hz
SCANS	8
ACQTM	0.2222 sec
PD	5.0000 sec
PW1	4.10 usec
IRNUC	19F
CTEMP	19.9 c
SLVNT	CDCL3
EXREF	-164.90 ppm
BF	1.02 Hz
RGAIN	56

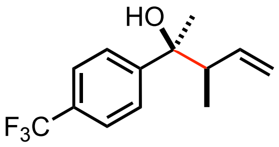

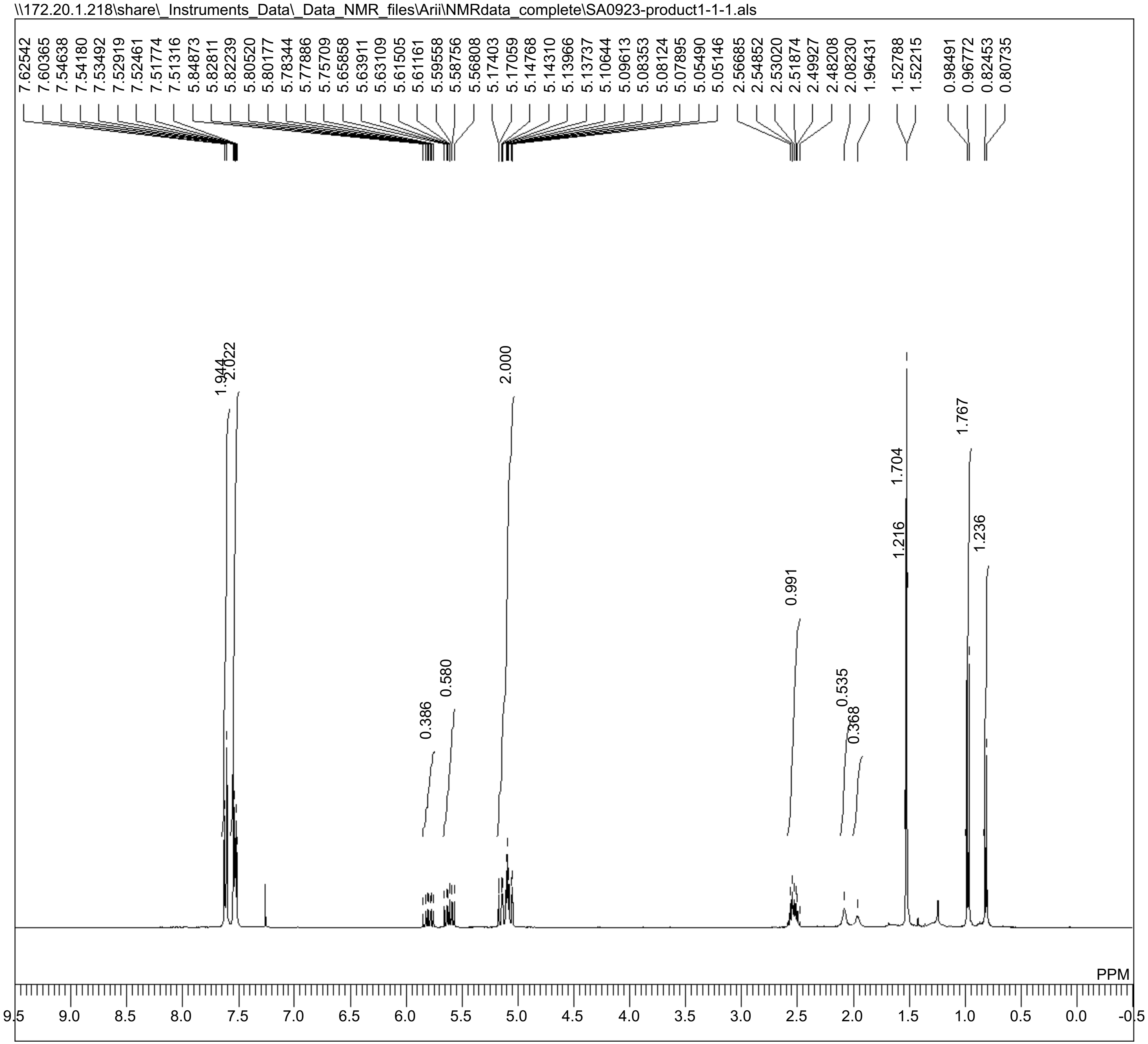

DFILE	SA0923-product1-1-1.als
COMNT	
DATIM	2024-12-06 13:42:40
OBNUC	1H
EXMOD	proton.jxp
OBFRQ	391.78 MHz
OBSET	8.51 KHz
OBFIN	3.34 Hz
POINT	13107
FREQU	5882.35 Hz
SCANS	8
ACQTM	2.2282 sec
PD	4.0000 sec
PW1	6.30 usec
IRNUC	1H
CTEMP	20.9 c
SLVNT	CDCL3
EXREF	7.26 ppm
BF	0.12 Hz
RGAIN	30

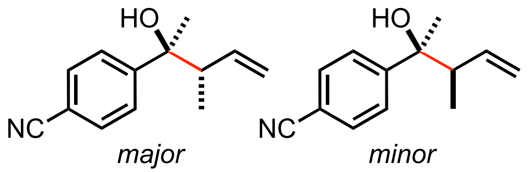

8i

\\172.20.1.218\share_Instruments_Data_Data_NMR_files\Arii\NMRdata_complete\SA0923-carbon-1-1.als

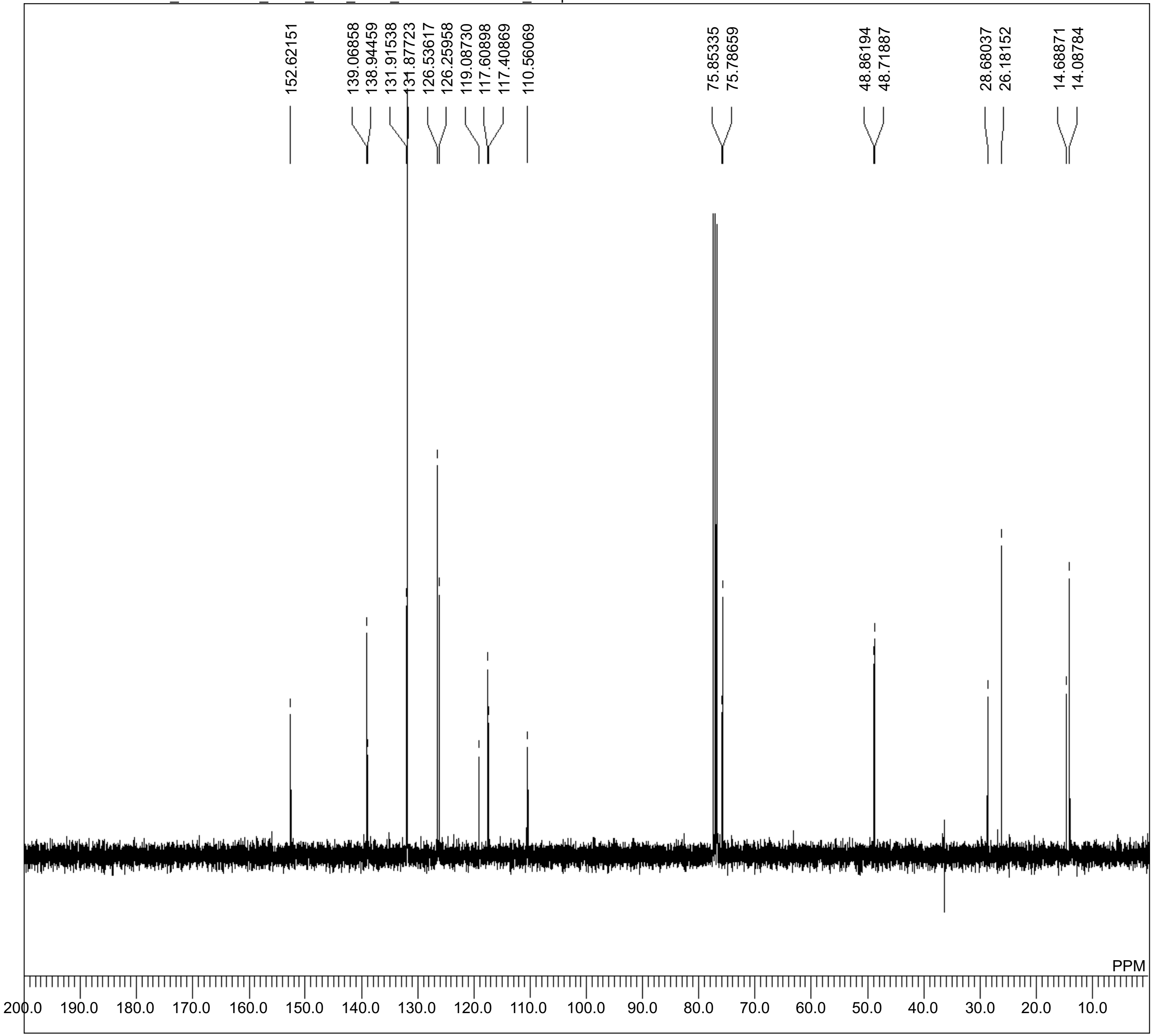

DFILE	SA0923-carbon-1-1.als
COMNT	
DATIM	2024-12-06 13:45:25
OBNUC	13C
EXMOD	carbon.jxp
OBFRQ	98.52 MHz
OBSET	4.64 KHz
OBFIN	8.74 Hz
POINT	26214
FREQU	24630.54 Hz
SCANS	118
ACQTM	1.0643 sec
PD	2.0000 sec
PW1	2.93 usec
IRNUC	1H
CTEMP	21.1 c
SLVNT	CDCL3
EXREF	77.16 ppm
BF	0.12 Hz
RGAIN	60

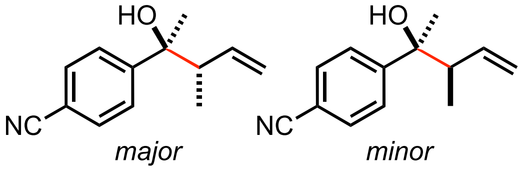

8i

\\172.20.1.218\share_Instruments_Data_Data_NMR_files\Arii\NMRdata_complete\SA0952-proton-1-1.als

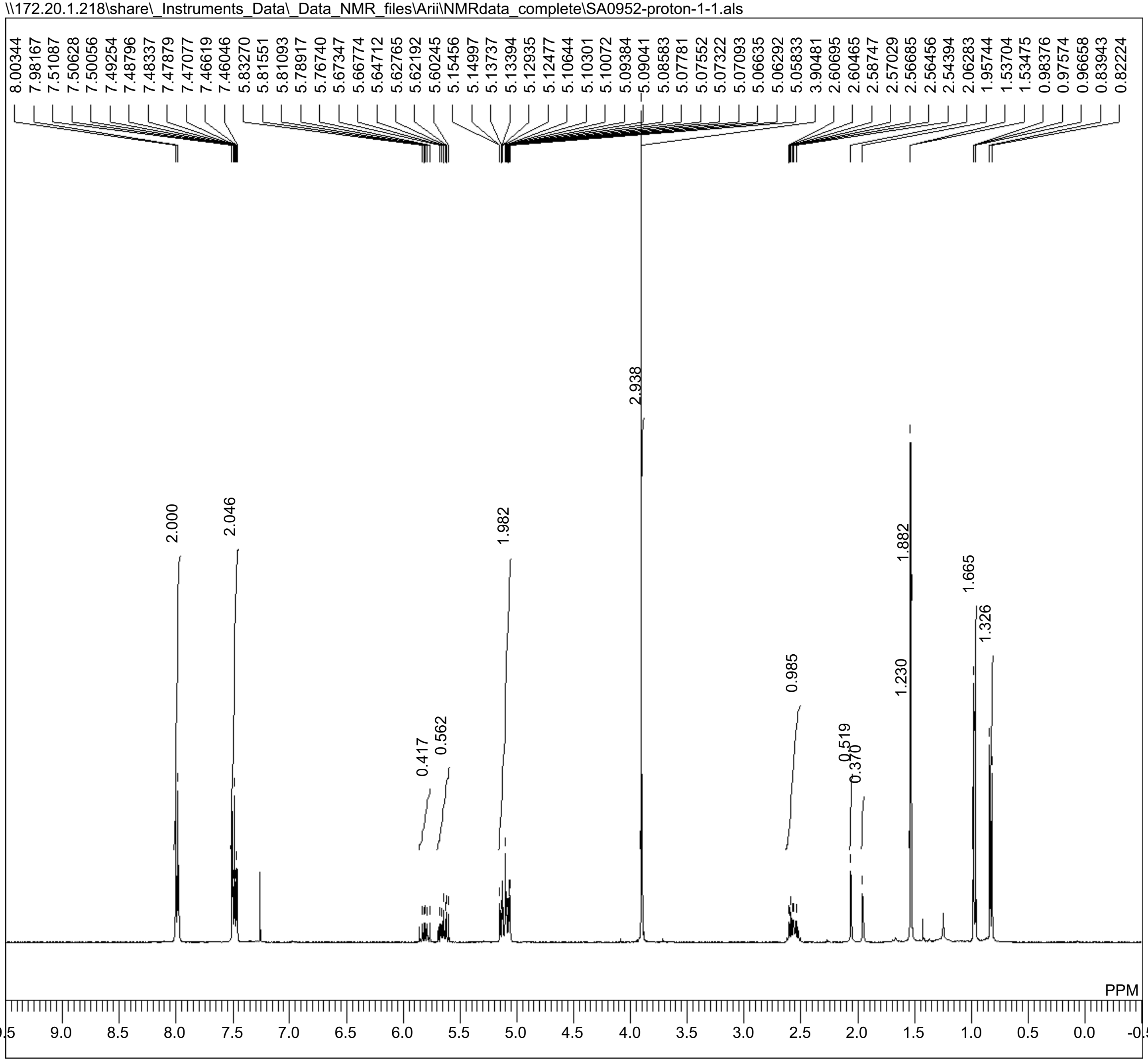

DFILE	SA0952-proton-1-1.als
COMNT	
DATIM	2025-01-10 16:19:43
OBNUC	1H
EXMOD	proton.jxp
OBFRQ	391.78 MHz
OBSET	8.51 KHz
OBFIN	3.34 Hz
POINT	13107
FREQU	5882.35 Hz
SCANS	8
ACQTM	2.2282 sec
PD	4.0000 sec
PW1	6.30 usec
IRNUC	1H
CTEMP	20.7 c
SLVNT	CDCL3
EXREF	7.26 ppm
BF	0.12 Hz
RGAIN	36

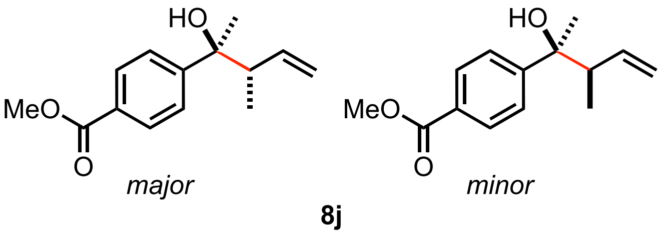

major *minor*

8j

\\172.20.1.218\share_Instruments_Data_Data_NMR_files\Arii\NMRdata_complete\SA0952-carbon-1-1.als

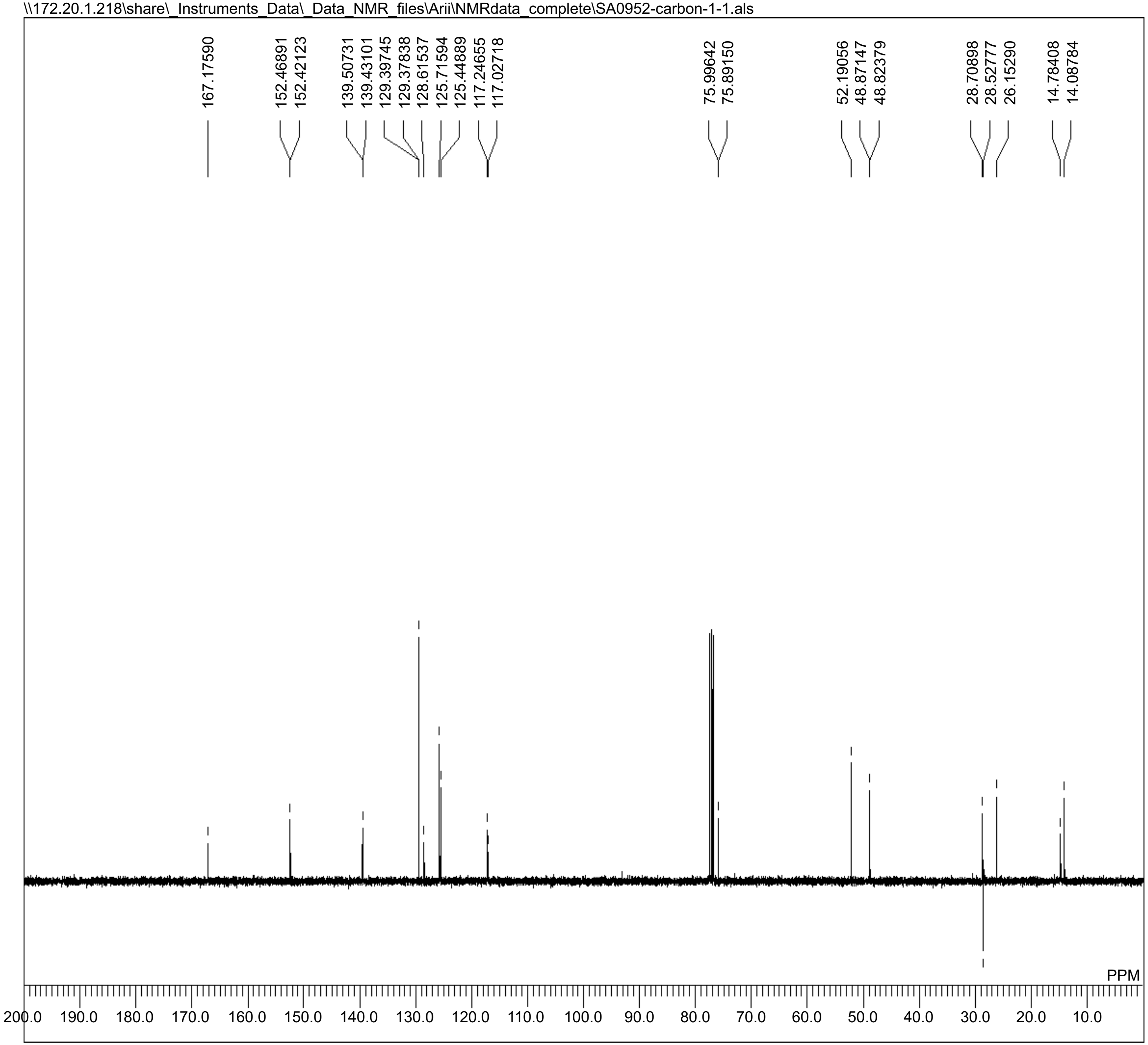

DFILE	SA0952-carbon-1-1.als
COMNT	
DATIM	2025-01-10 16:21:58
OBNUC	13C
EXMOD	carbon.jxp
OBFRQ	98.52 MHz
OBSET	4.64 KHz
OBFIN	8.74 Hz
POINT	26214
FREQU	24630.54 Hz
SCANS	232
ACQTM	1.0643 sec
PD	2.0000 sec
PW1	2.93 usec
IRNUC	1H
CTEMP	21.0 c
SLVNT	CDCL3
EXREF	77.16 ppm
BF	0.12 Hz
RGAIN	60

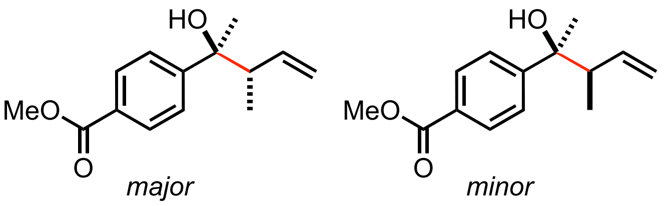

8j

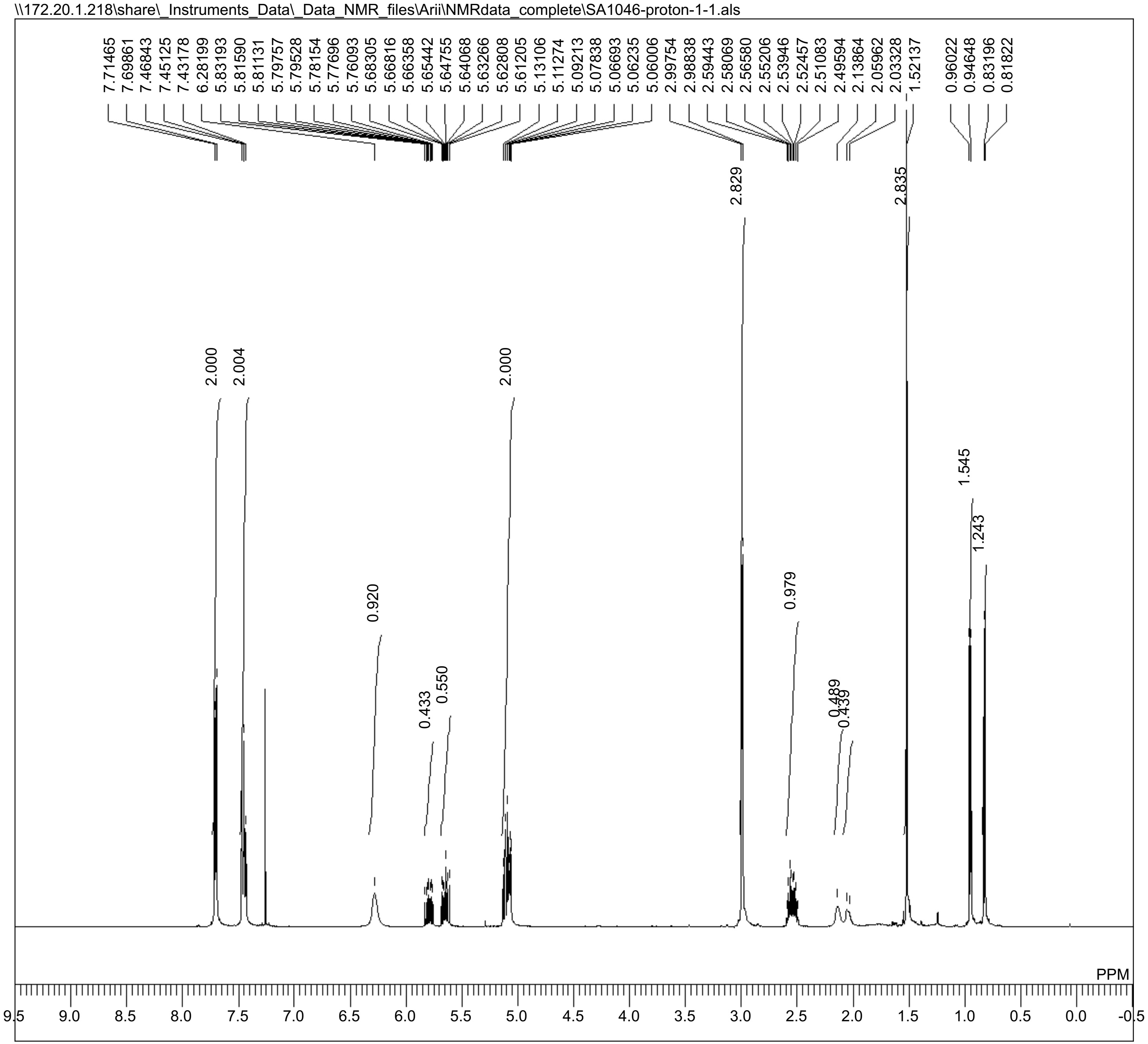

DFILE	SA1046-proton-1-1.als
COMNT	
DATIM	2025-01-16 20:57:33
OBNUC	1H
EXMOD	proton.jxp
OBFRQ	500.16 MHz
OBSET	2.41 KHz
OBFIN	6.01 Hz
POINT	13107
FREQU	7507.51 Hz
SCANS	8
ACQTM	1.7459 sec
PD	5.0000 sec
PW1	5.55 usec
IRNUC	1H
CTEMP	21.6 c
SLVNT	CDCL3
EXREF	7.26 ppm
BF	0.12 Hz
RGAIN	30

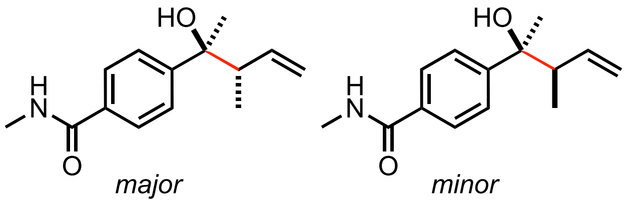

8k

\\172.20.1.218\share_Instruments_Data_Data_NMR_files\Arii\NMRdata_complete\SA1046-carbon-1-1.als

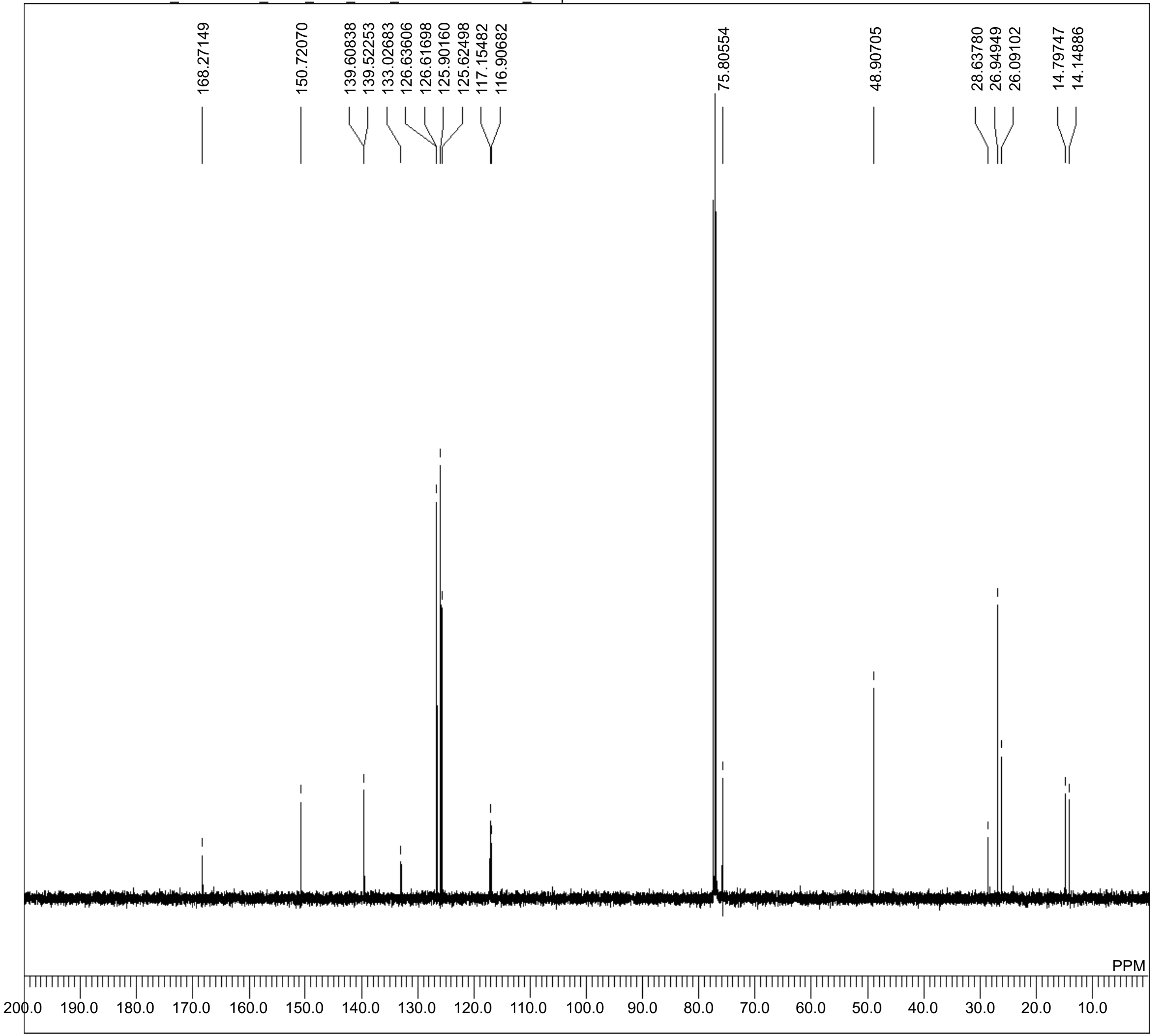

DFILE	SA1046-carbon-1-1.als
COMNT	
DATIM	2025-01-16 20:59:06
OBNUC	13C
EXMOD	carbon.jxp
OBFRQ	125.77 MHz
OBSET	7.87 KHz
OBFIN	4.21 Hz
POINT	26214
FREQU	31446.54 Hz
SCANS	730
ACQTM	0.8336 sec
PD	1.0000 sec
PW1	3.40 usec
IRNUC	1H
CTEMP	22.0 c
SLVNT	CDCL3
EXREF	77.16 ppm
BF	0.12 Hz
RGAIN	60

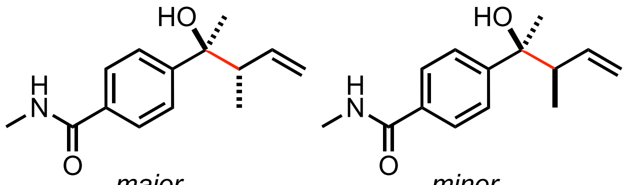

major *minor*

8k

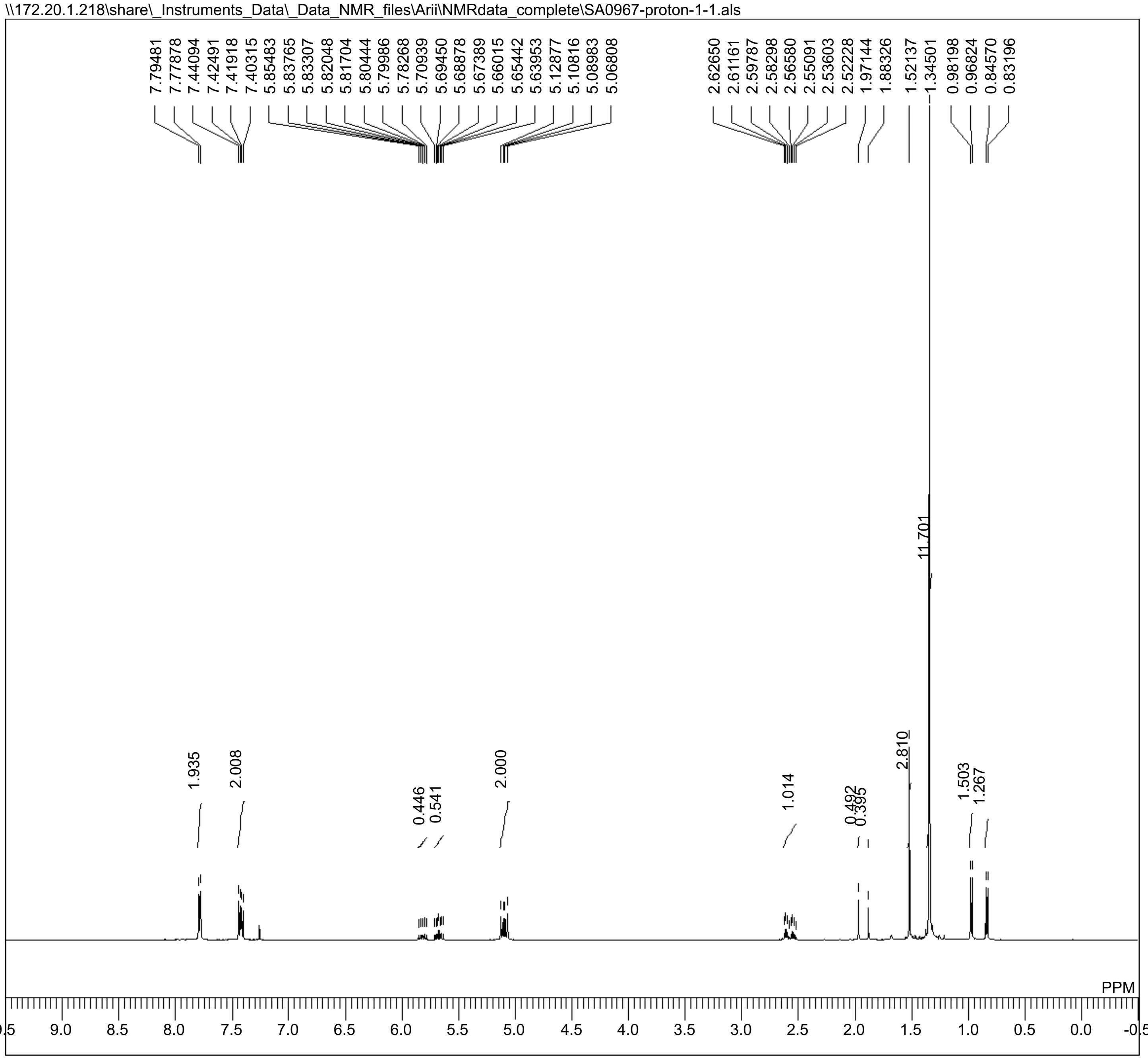

DFILE	SA0967-proton-1-1.als
COMNT	
DATIM	2024-12-06 14:07:06
OBNUC	1H
EXMOD	proton.jxp
OBFRQ	500.16 MHz
OBSET	2.41 KHz
OBFIN	6.01 Hz
POINT	13107
FREQU	7507.51 Hz
SCANS	8
ACQTM	1.7459 sec
PD	5.0000 sec
PW1	5.55 usec
IRNUC	1H
CTEMP	21.7 c
SLVNT	CDCL3
EXREF	7.26 ppm
BF	0.12 Hz
RGAIN	28

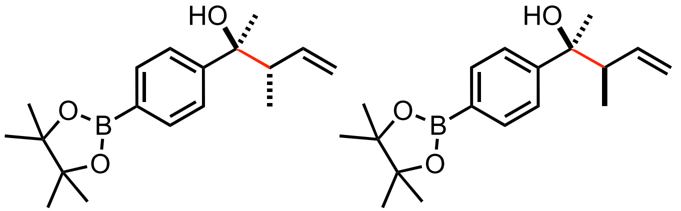

major *minor*

8l

\\172.20.1.218\share_Instruments_Data_Data_NMR_files\Arii\NMRdata_complete\SA0967-carbon-1-1.als

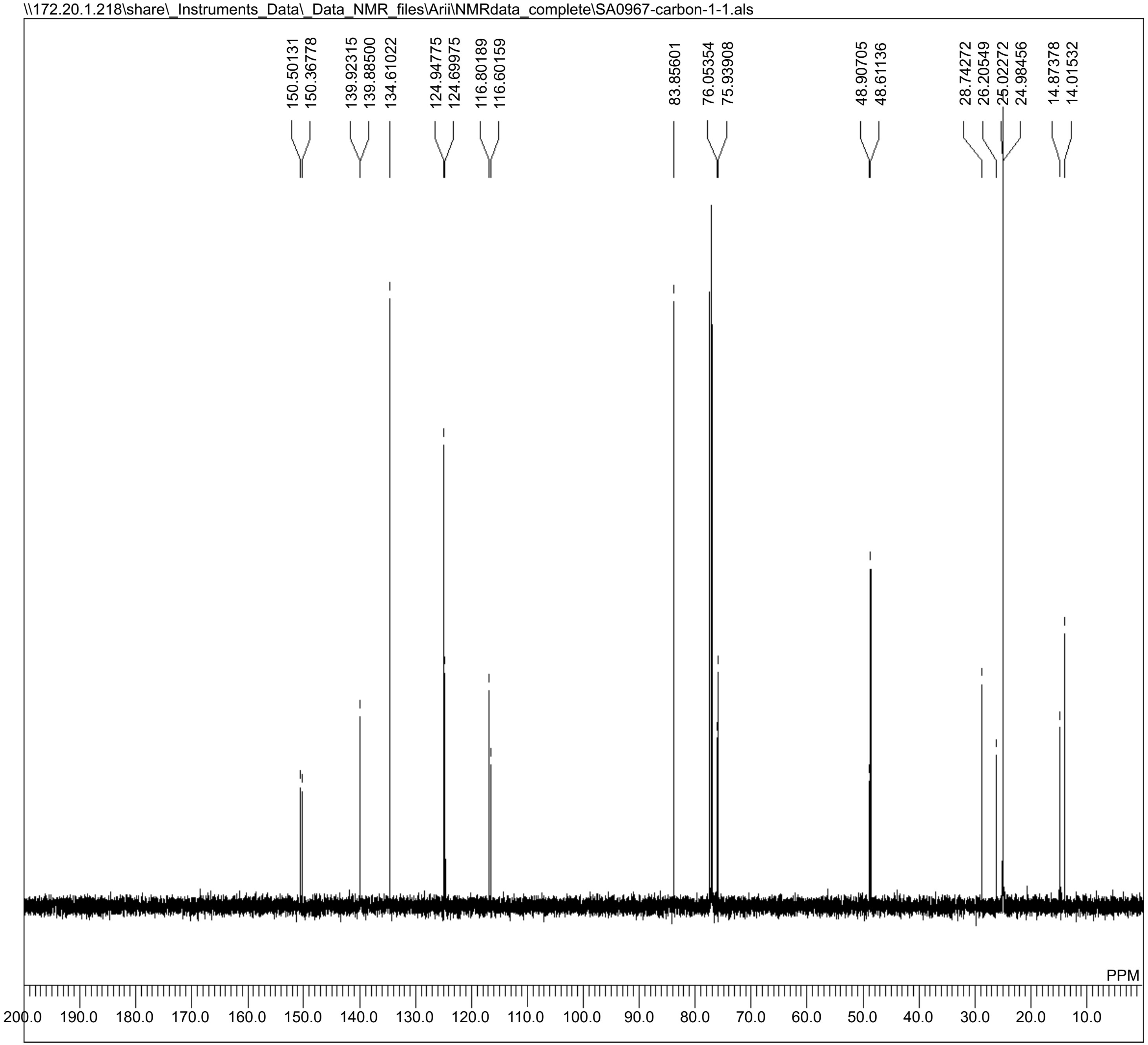

DFILE	SA0967-carbon-1-1.als
COMNT	
DATIM	2024-12-06 14:11:47
OBNUC	13C
EXMOD	carbon.jxp
OBFRQ	125.77 MHz
OBSET	7.87 KHz
OBFIN	4.21 Hz
POINT	26214
FREQU	31446.54 Hz
SCANS	269
ACQTM	0.8336 sec
PD	1.0000 sec
PW1	3.40 usec
IRNUC	1H
CTEMP	22.0 c
SLVNT	CDCL3
EXREF	77.16 ppm
BF	0.12 Hz
RGAIN	60

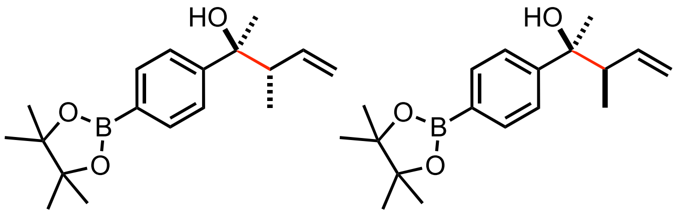

major *minor*

8l

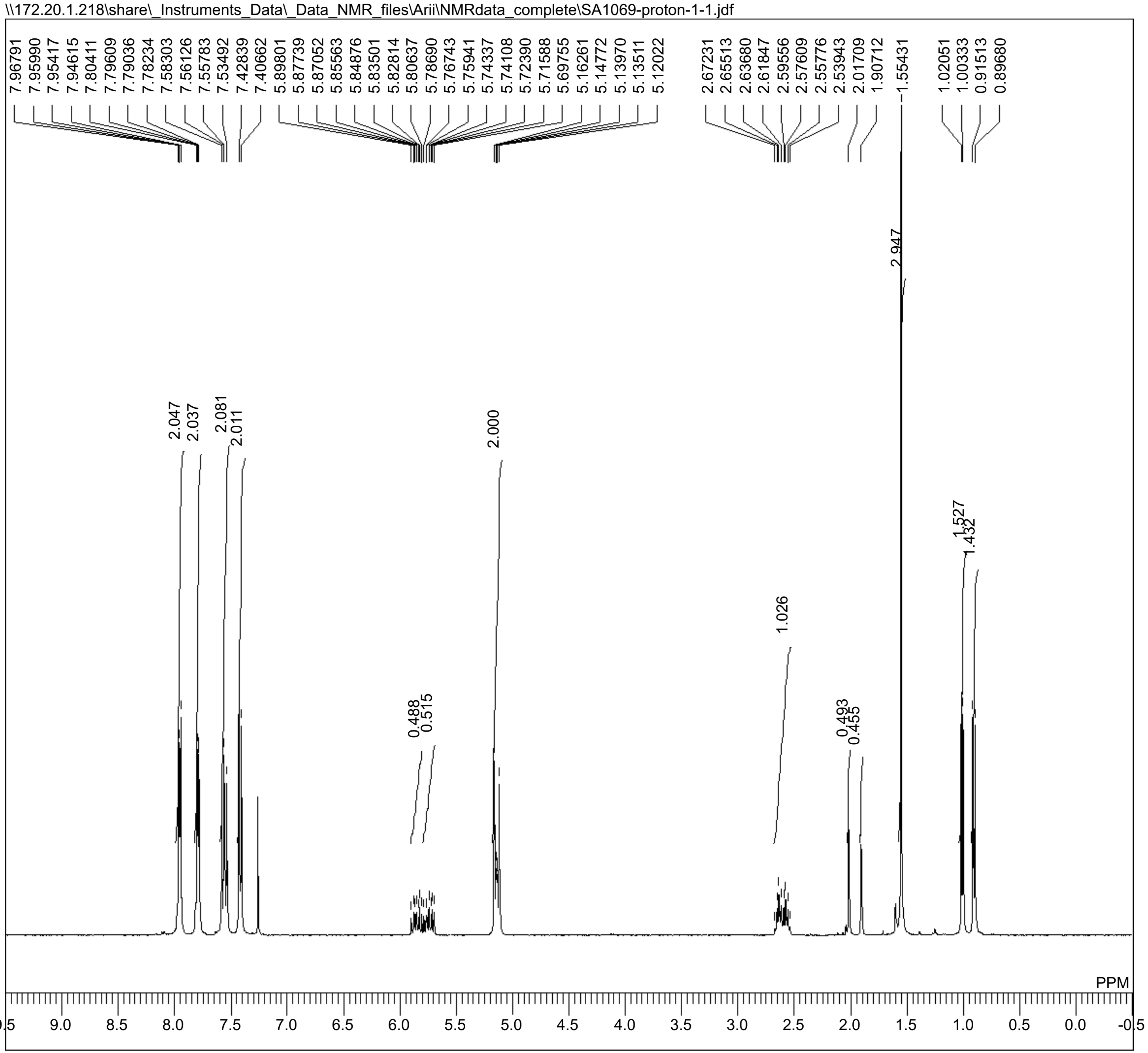

DFILE	SA1069-proton-1-1.jdf
COMNT	
DATIM	2025-01-26 14:50:08
OBNUC	1H
EXMOD	proton.jxp
OBFRQ	391.78 MHz
OBSET	8.51 KHz
OBFIN	3.34 Hz
POINT	16384
FREQU	7352.94 Hz
SCANS	8
ACQTM	2.2282 sec
PD	4.0000 sec
PW1	6.30 usec
IRNUC	1H
CTEMP	20.8 c
SLVNT	CDCL3
EXREF	7.26 ppm
BF	0.72 Hz
RGAIN	44

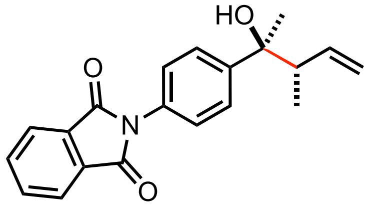

major

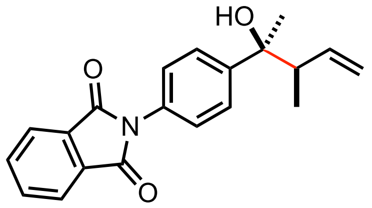

minor

8m

\\172.20.1.218\share_Instruments_Data_Data_NMR_files\Arii\NMRdata_complete\SA1069-carbon-1-1.als

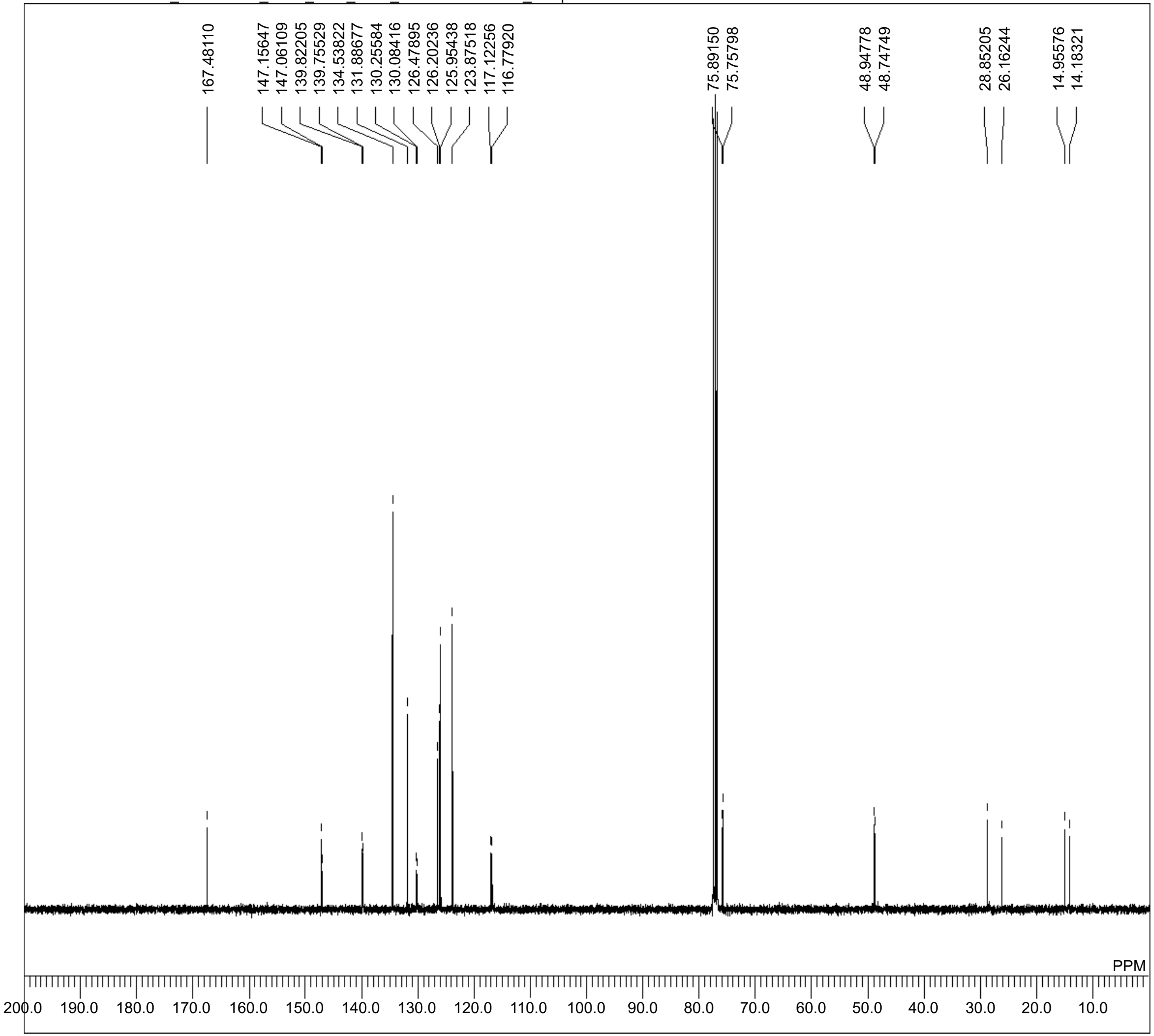

DFILE	SA1069-carbon-1-1.als
COMNT	
DATIM	2025-01-26 14:51:35
OBNUC	13C
EXMOD	carbon.jxp
OBFRQ	98.52 MHz
OBSET	4.64 KHz
OBFIN	8.74 Hz
POINT	26214
FREQU	24630.54 Hz
SCANS	1034
ACQTM	1.0643 sec
PD	2.0000 sec
PW1	2.93 usec
IRNUC	1H
CTEMP	20.7 c
SLVNT	CDCL3
EXREF	77.16 ppm
BF	0.42 Hz
RGAIN	60

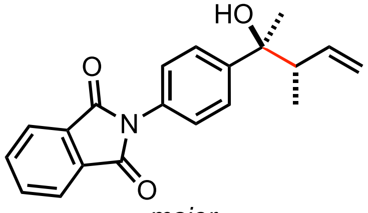

major

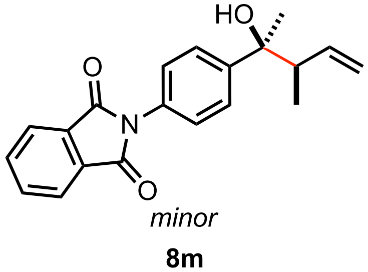

minor

8m

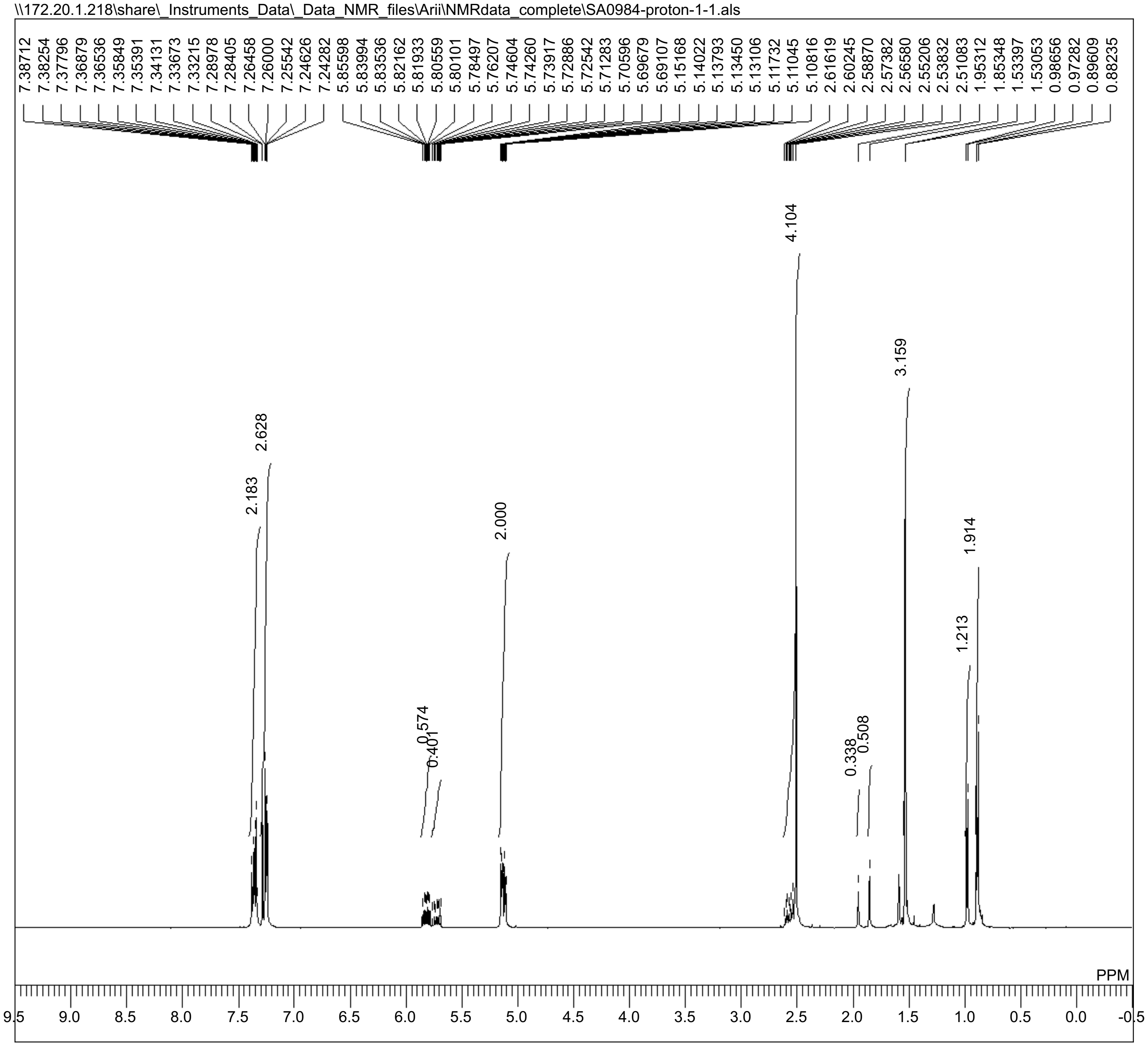

DFILE	SA0984-proton-1-1.als
COMNT	
DATIM	2024-12-06 14:56:23
OBNUC	1H
EXMOD	proton.jxp
OBFRQ	500.16 MHz
OBSET	2.41 KHz
OBFIN	6.01 Hz
POINT	13107
FREQU	7507.51 Hz
SCANS	8
ACQTM	1.7459 sec
PD	5.0000 sec
PW1	5.55 usec
IRNUC	1H
CTEMP	22.1 c
SLVNT	CDCL3
EXREF	7.26 ppm
BF	0.12 Hz
RGAIN	30

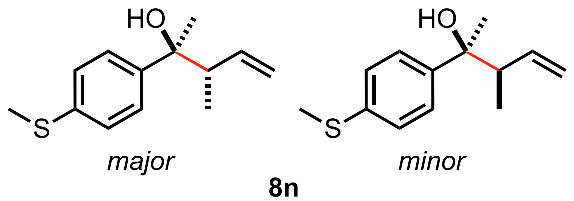

8n

\\172.20.1.218\share_Instruments_Data_Data_NMR_files\Arii\paperNMR\SA0984-carbon-1-1.als

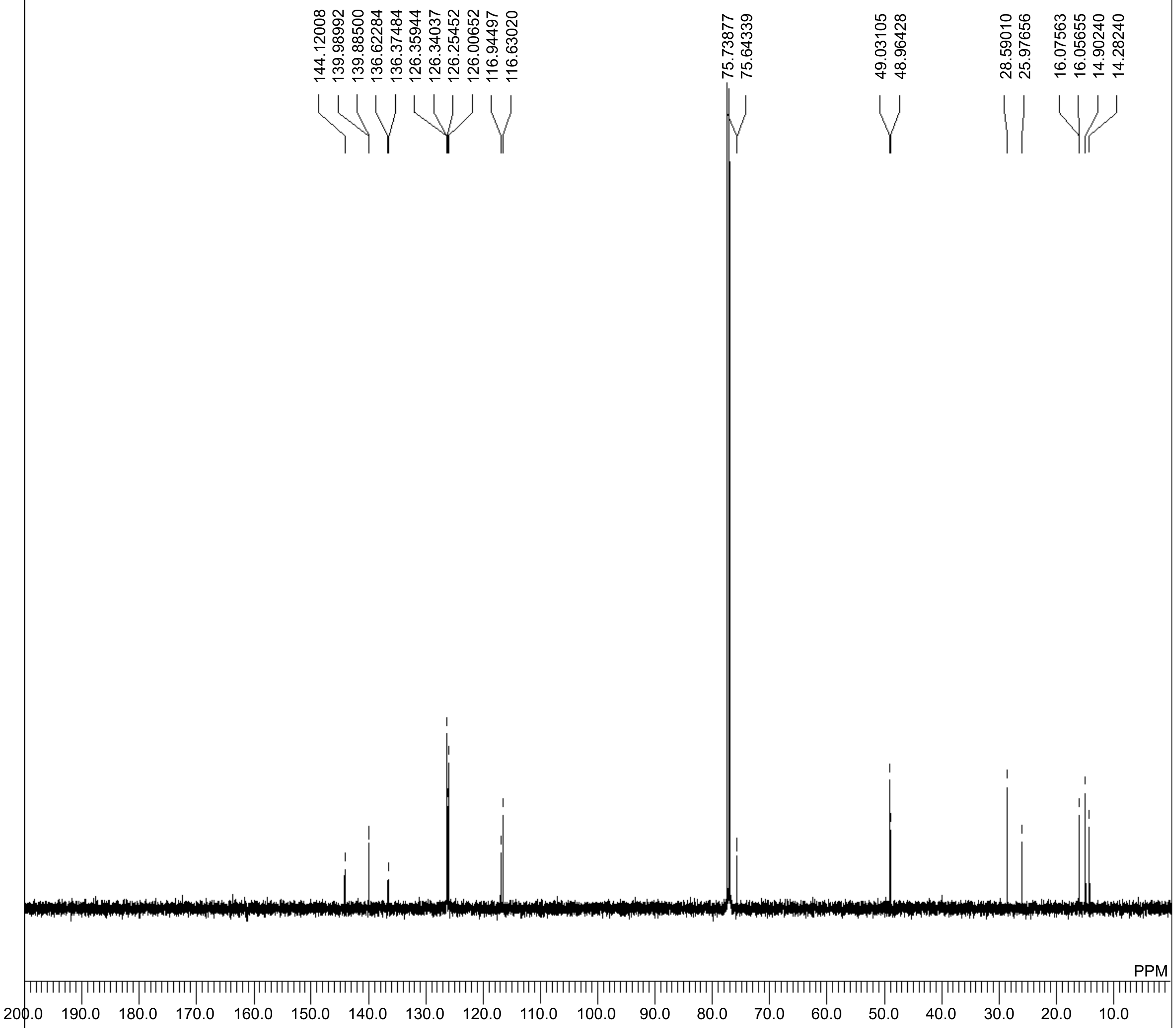

DFILE	SA0984-carbon-1-1.als
COMNT	
DATIM	2024-12-06 14:58:05
OBNUC	13C
EXMOD	carbon.jxp
OBFRQ	125.77 MHz
OBSET	7.87 KHz
OBFIN	4.21 Hz
POINT	26214
FREQU	31446.54 Hz
SCANS	1108
ACQTM	0.8336 sec
PD	1.0000 sec
PW1	3.40 usec
IRNUC	1H
CTEMP	22.1 c
SLVNT	CDCL3
EXREF	77.16 ppm
BF	0.12 Hz
RGAIN	60

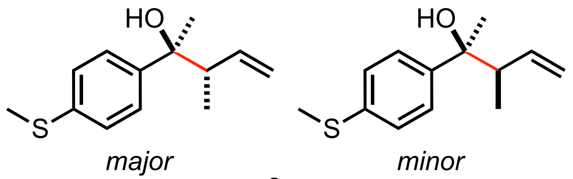

8n

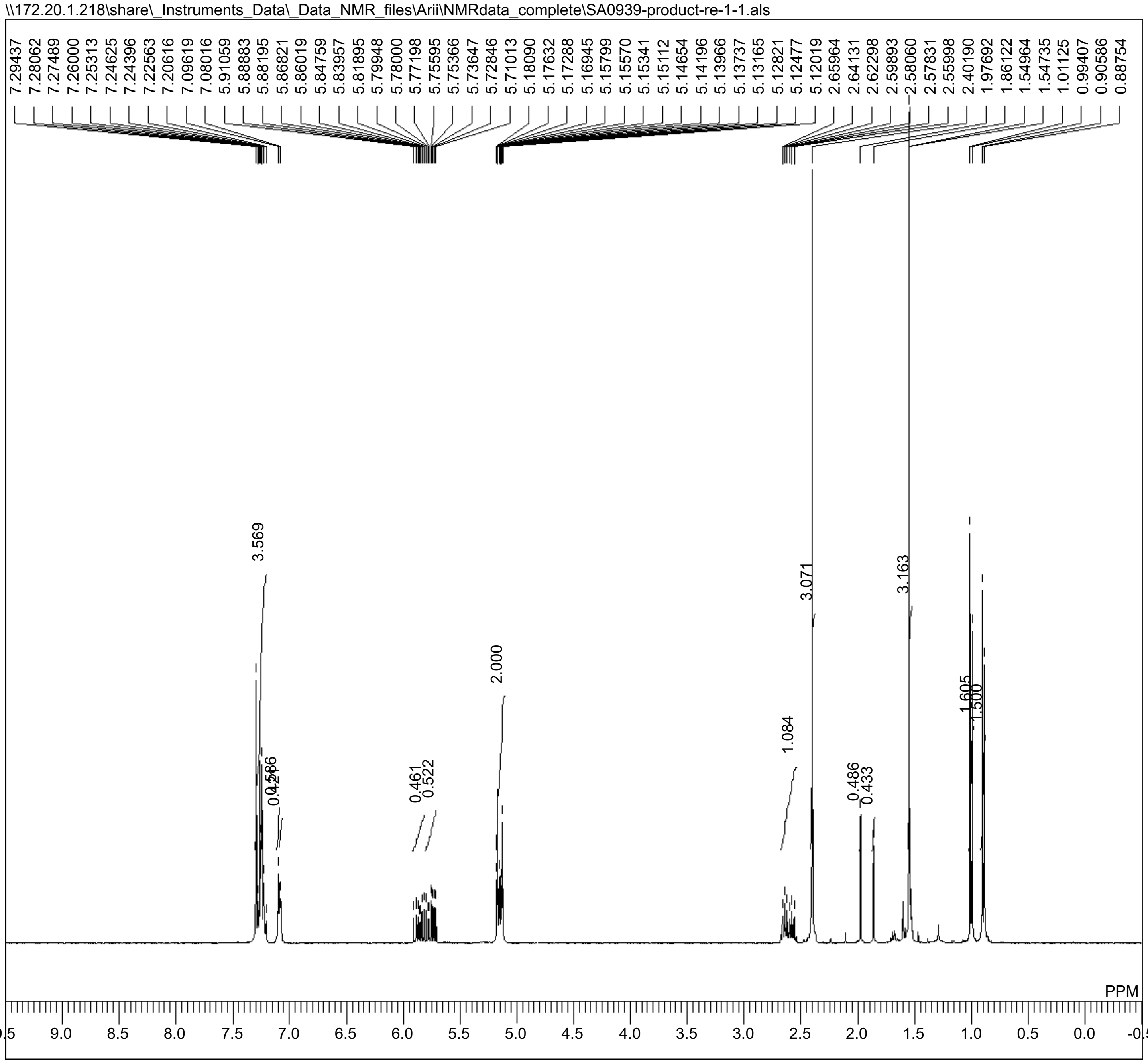

DFILE	SA0939-product-re-1-1.als
COMNT	
DATIM	2024-12-04 15:16:44
OBNUC	1H
EXMOD	proton.jxp
OBFRQ	391.78 MHz
OBSET	8.51 KHz
OBFIN	3.34 Hz
POINT	13107
FREQU	5882.35 Hz
SCANS	8
ACQTM	2.2282 sec
PD	4.0000 sec
PW1	6.30 usec
IRNUC	1H
CTEMP	20.6 c
SLVNT	CDCL3
EXREF	7.26 ppm
BF	0.12 Hz
RGAIN	38

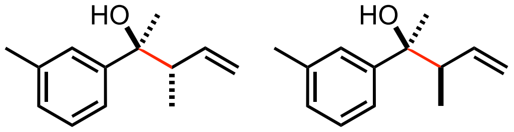

major **8o** *minor*

\\172.20.1.218\share_Instruments_Data_Data_NMR_files\Arii\NMRdata_complete\SA0939-carbon-1-1.als

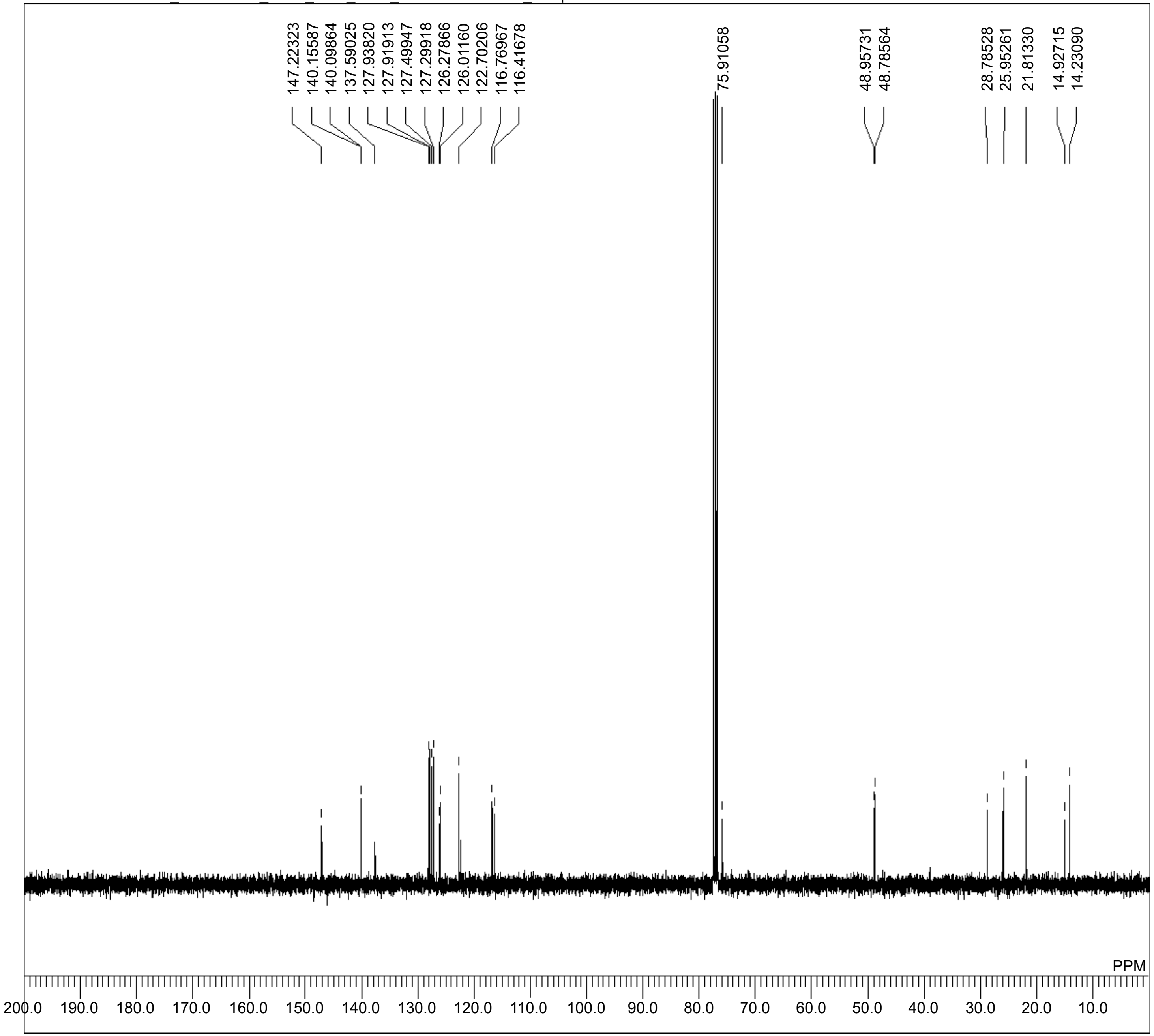

DFILE	SA0939-carbon-1-1.als
COMNT	
DATIM	2024-12-04 15:23:18
OBNUC	13C
EXMOD	carbon.jxp
OBFRQ	98.52 MHz
OBSET	4.64 KHz
OBFIN	8.74 Hz
POINT	26214
FREQU	24630.54 Hz
SCANS	342
ACQTM	1.0643 sec
PD	2.0000 sec
PW1	2.93 usec
IRNUC	1H
CTEMP	21.2 c
SLVNT	CDCL3
EXREF	77.16 ppm
BF	0.12 Hz
RGAIN	60

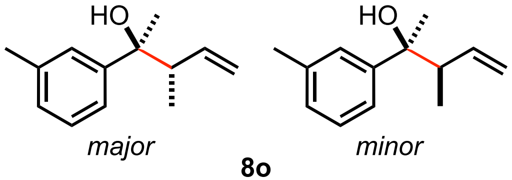

8o

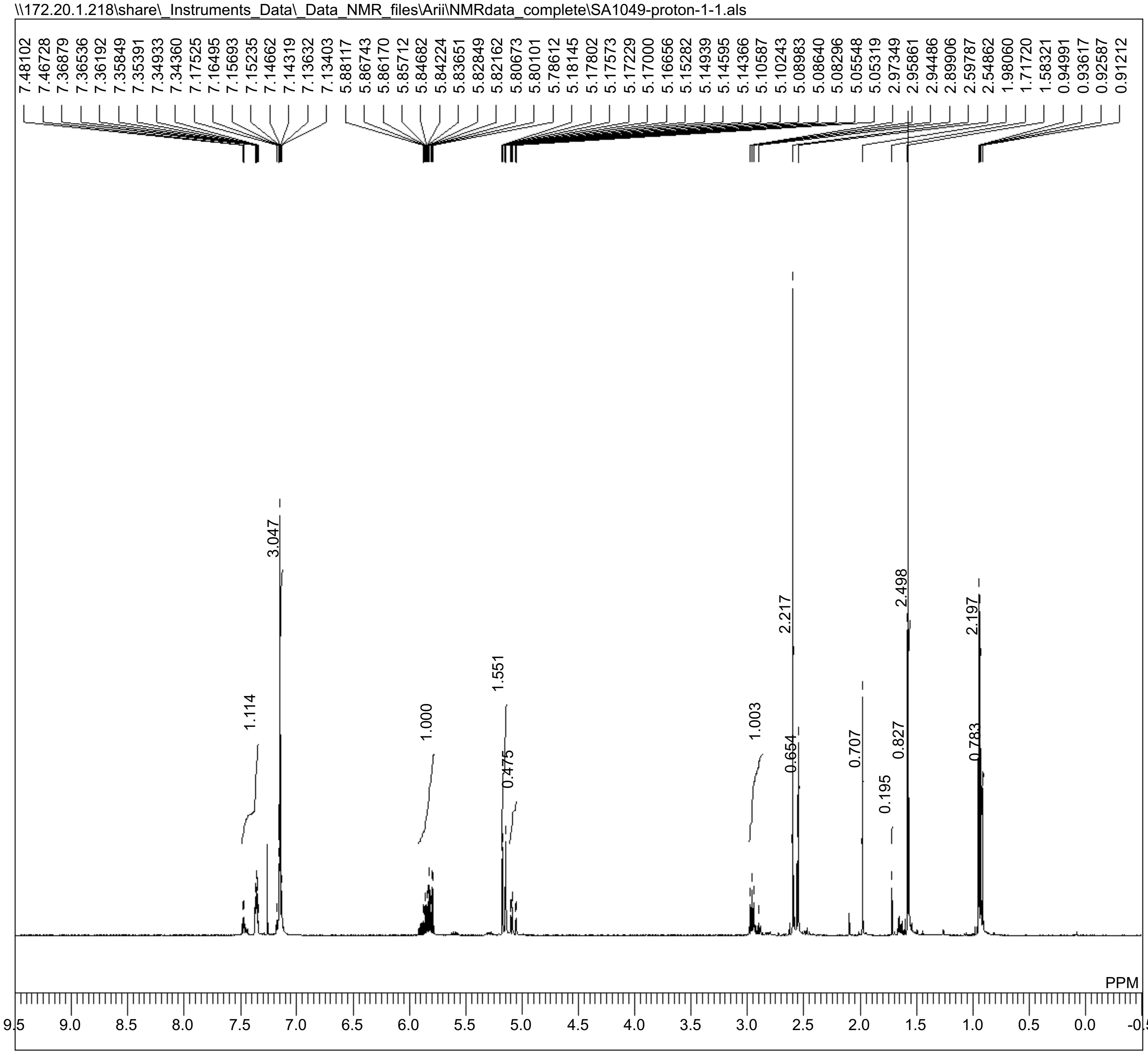

DFILE	SA1049-proton-1-1.als
COMNT	
DATIM	2025-01-18 06:55:27
OBNUC	1H
EXMOD	proton.jxp
OBFRQ	500.16 MHz
OBSET	2.41 KHz
OBFIN	6.01 Hz
POINT	13107
FREQU	7507.51 Hz
SCANS	8
ACQTM	1.7459 sec
PD	5.0000 sec
PW1	5.55 usec
IRNUC	1H
CTEMP	21.7 c
SLVNT	CDCL3
EXREF	7.26 ppm
BF	0.42 Hz
RGAIN	30

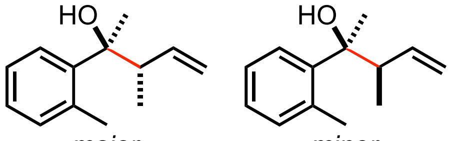

major **8p** *minor*

\\172.20.1.218\share_Instruments_Data_Data_NMR_files\Arii\NMRdata_complete\SA1049-carbon-1-1.als

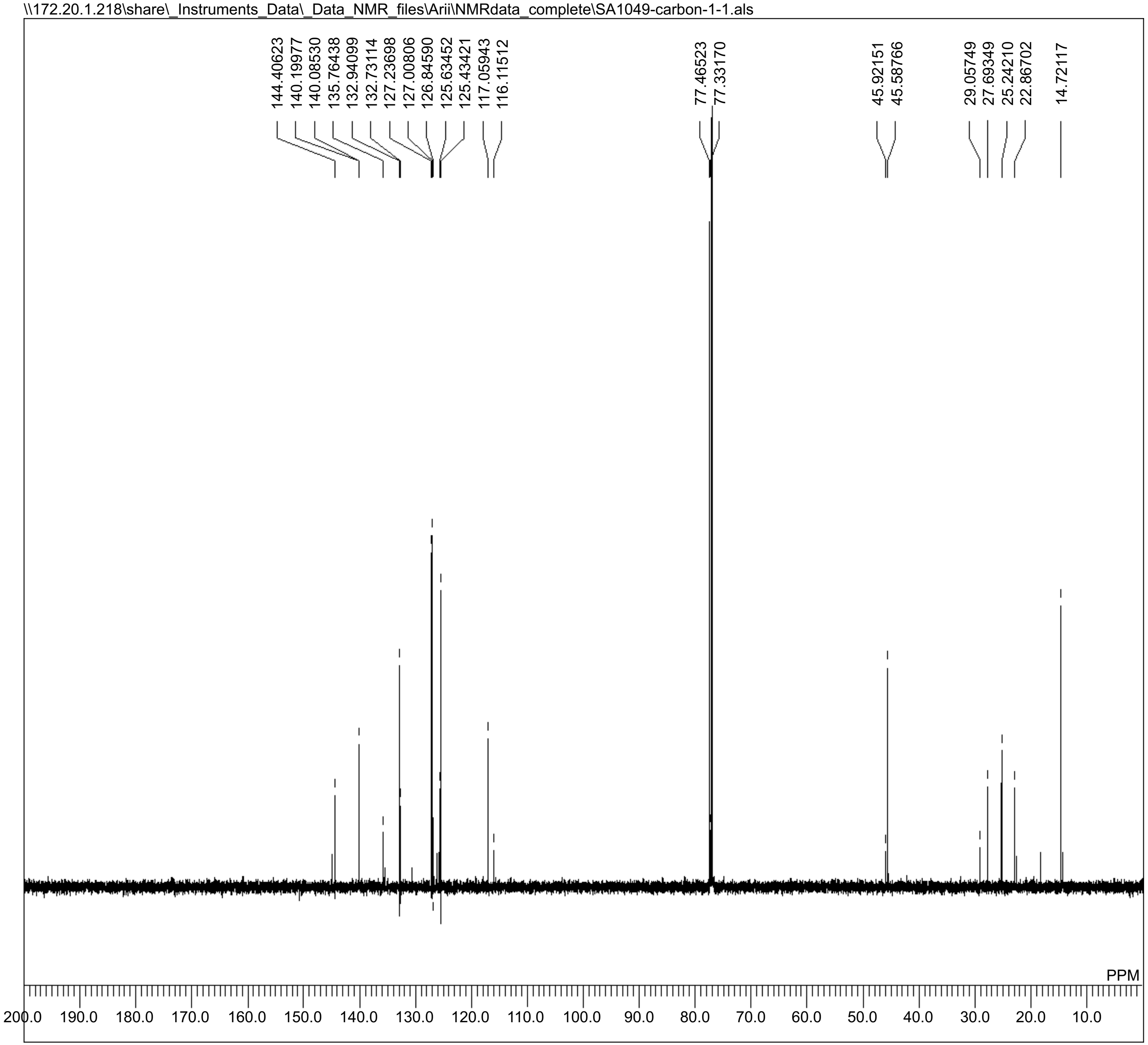

DFILE	SA1049-carbon-1-1.als
COMNT	
DATIM	2025-01-18 07:02:10
OBNUC	13C
EXMOD	carbon.jxp
OBFRQ	125.77 MHz
OBSET	7.87 KHz
OBFIN	4.21 Hz
POINT	26214
FREQU	31446.54 Hz
SCANS	1000
ACQTM	0.8336 sec
PD	1.0000 sec
PW1	3.40 usec
IRNUC	1H
CTEMP	21.6 c
SLVNT	CDCL3
EXREF	77.16 ppm
BF	0.42 Hz
RGAIN	60

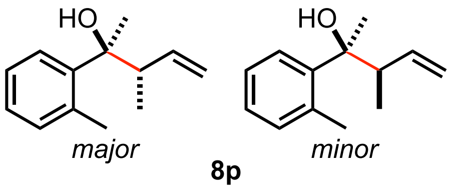

major **8p** *minor*

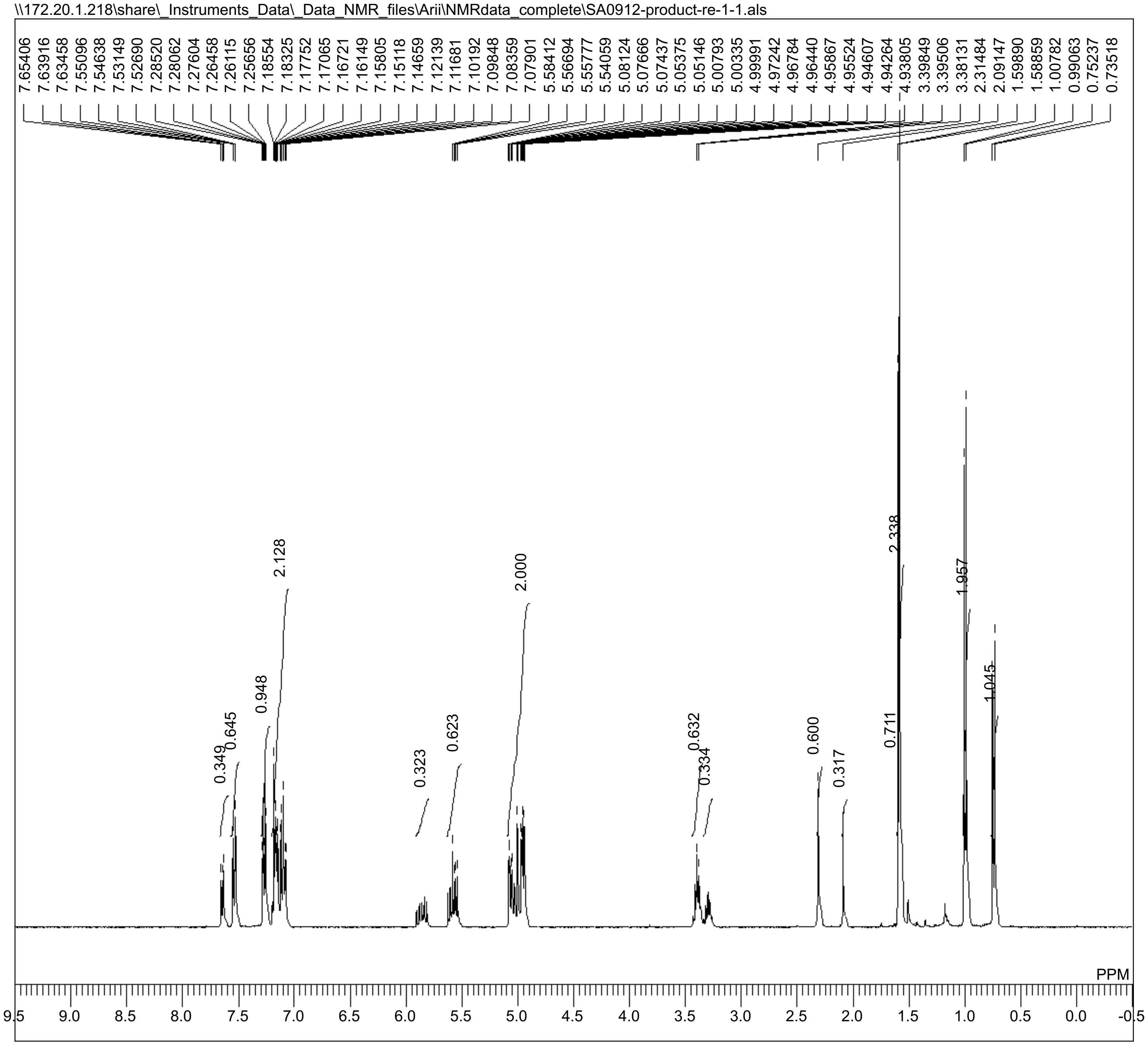

DFILE	SA0912-product-re-1-1.als
COMNT	
DATIM	2024-12-04 14:25:07
OBNUC	1H
EXMOD	proton.jxp
OBFRQ	391.78 MHz
OBSET	8.51 KHz
OBFIN	3.34 Hz
POINT	13107
FREQU	5882.35 Hz
SCANS	8
ACQTM	2.2282 sec
PD	4.0000 sec
PW1	6.30 usec
IRNUC	1H
CTEMP	21.0 c
SLVNT	CDCL3
EXREF	7.26 ppm
BF	0.12 Hz
RGAIN	34

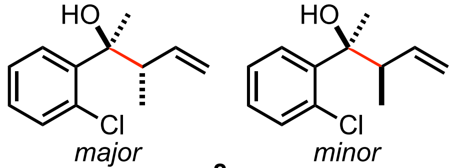

8q

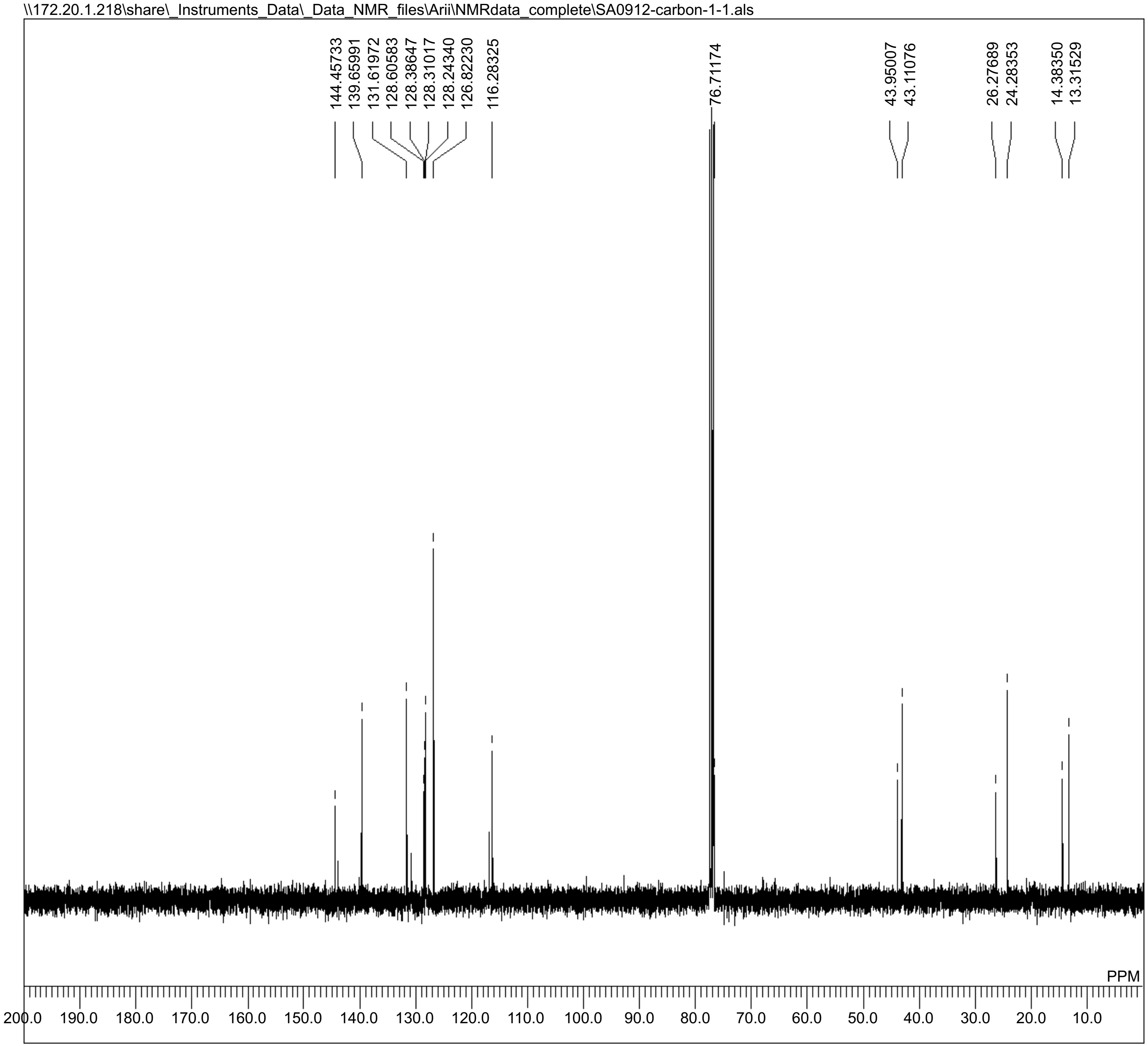

DFILE	SA0912-carbon-1-1.als
COMNT	
DATIM	2024-12-04 14:31:07
OBNUC	13C
EXMOD	carbon.jxp
OBFRQ	98.52 MHz
OBSET	4.64 KHz
OBFIN	8.74 Hz
POINT	26214
FREQU	24630.54 Hz
SCANS	343
ACQTM	1.0643 sec
PD	2.0000 sec
PW1	2.93 usec
IRNUC	1H
CTEMP	21.0 c
SLVNT	CDCL3
EXREF	77.16 ppm
BF	0.12 Hz
RGAIN	60

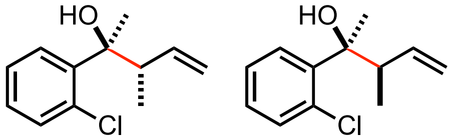

major **8q** *minor*

\\172.20.1.218\share_Instruments_Data_Data_NMR_files\Arii\NMRdata_complete\SA0935-proton-1-1.als

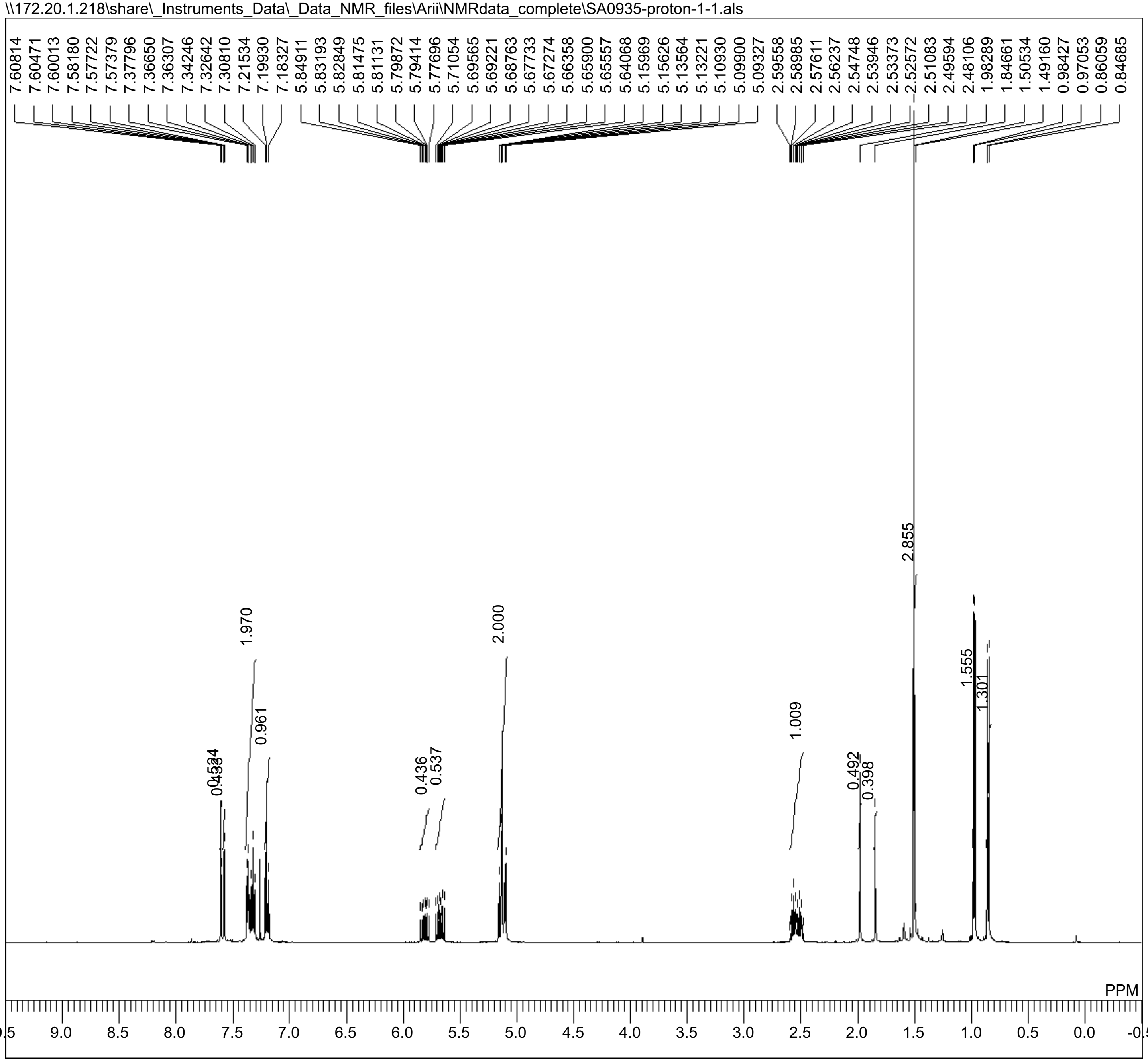

DFILE	SA0935-proton-1-1.als
COMNT	
DATIM	2024-12-06 13:45:31
OBNUC	1H
EXMOD	proton.jxp
OBFRQ	500.16 MHz
OBSET	2.41 KHz
OBFIN	6.01 Hz
POINT	13107
FREQU	7507.51 Hz
SCANS	8
ACQTM	1.7459 sec
PD	5.0000 sec
PW1	5.55 usec
IRNUC	1H
CTEMP	21.9 c
SLVNT	CDCL3
EXREF	7.26 ppm
BF	0.12 Hz
RGAIN	30

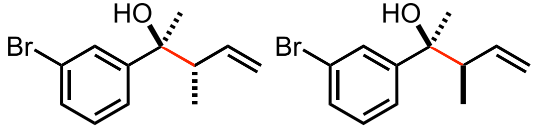

major *minor*

8r

\\172.20.1.218\share_Instruments_Data_Data_NMR_files\Arii\NMRdata_complete\SA0935-carbon-1-1.als

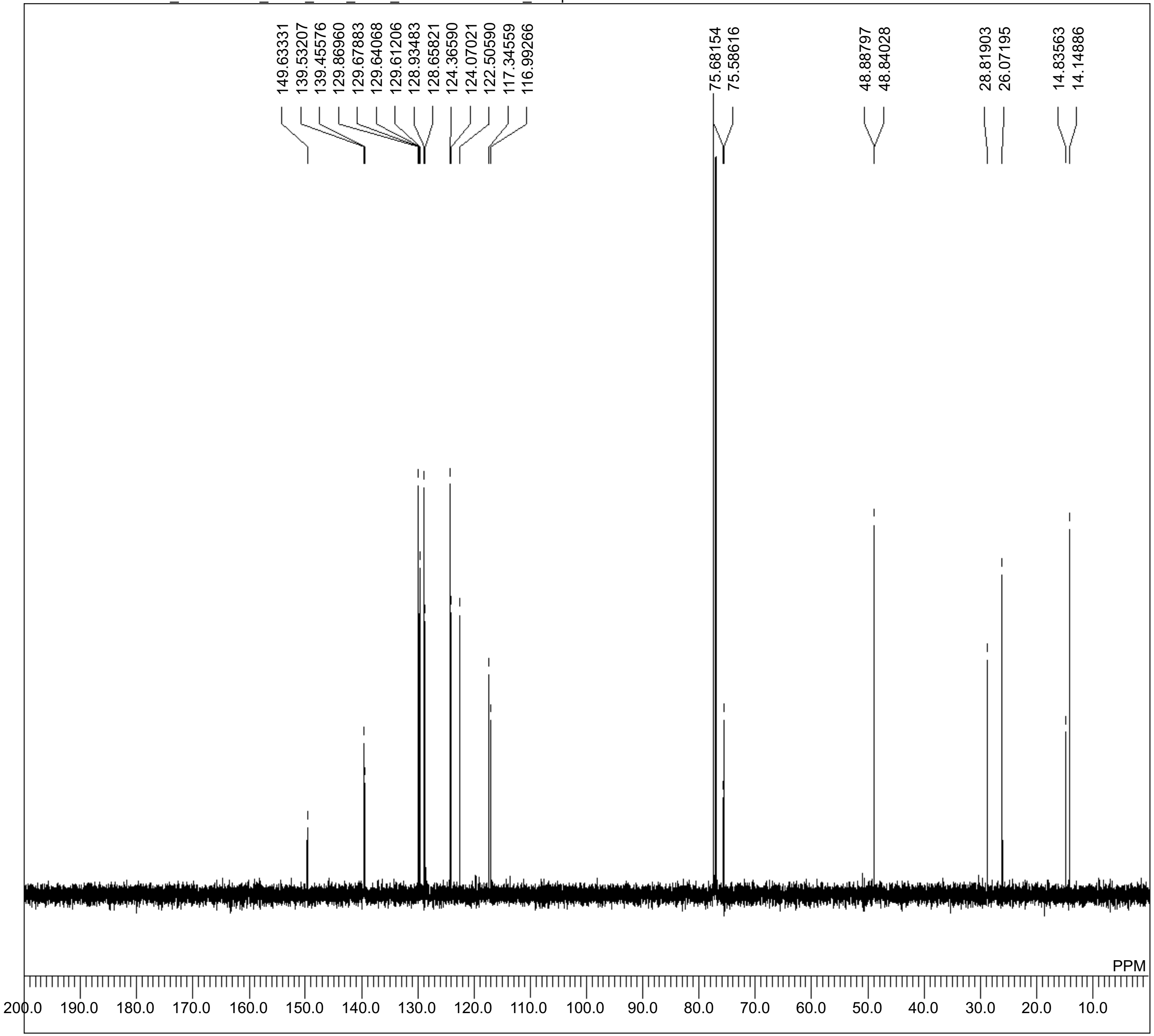

DFILE	SA0935-carbon-1-1.als
COMNT	
DATIM	2024-12-06 13:47:08
OBNUC	13C
EXMOD	carbon.jxp
OBFRQ	125.77 MHz
OBSET	7.87 KHz
OBFIN	4.21 Hz
POINT	26214
FREQU	31446.54 Hz
SCANS	323
ACQTM	0.8336 sec
PD	1.0000 sec
PW1	3.40 usec
IRNUC	1H
CTEMP	21.9 c
SLVNT	CDCL3
EXREF	77.16 ppm
BF	0.12 Hz
RGAIN	60

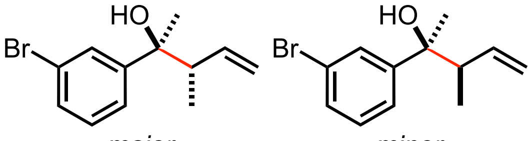

major *minor*

8r

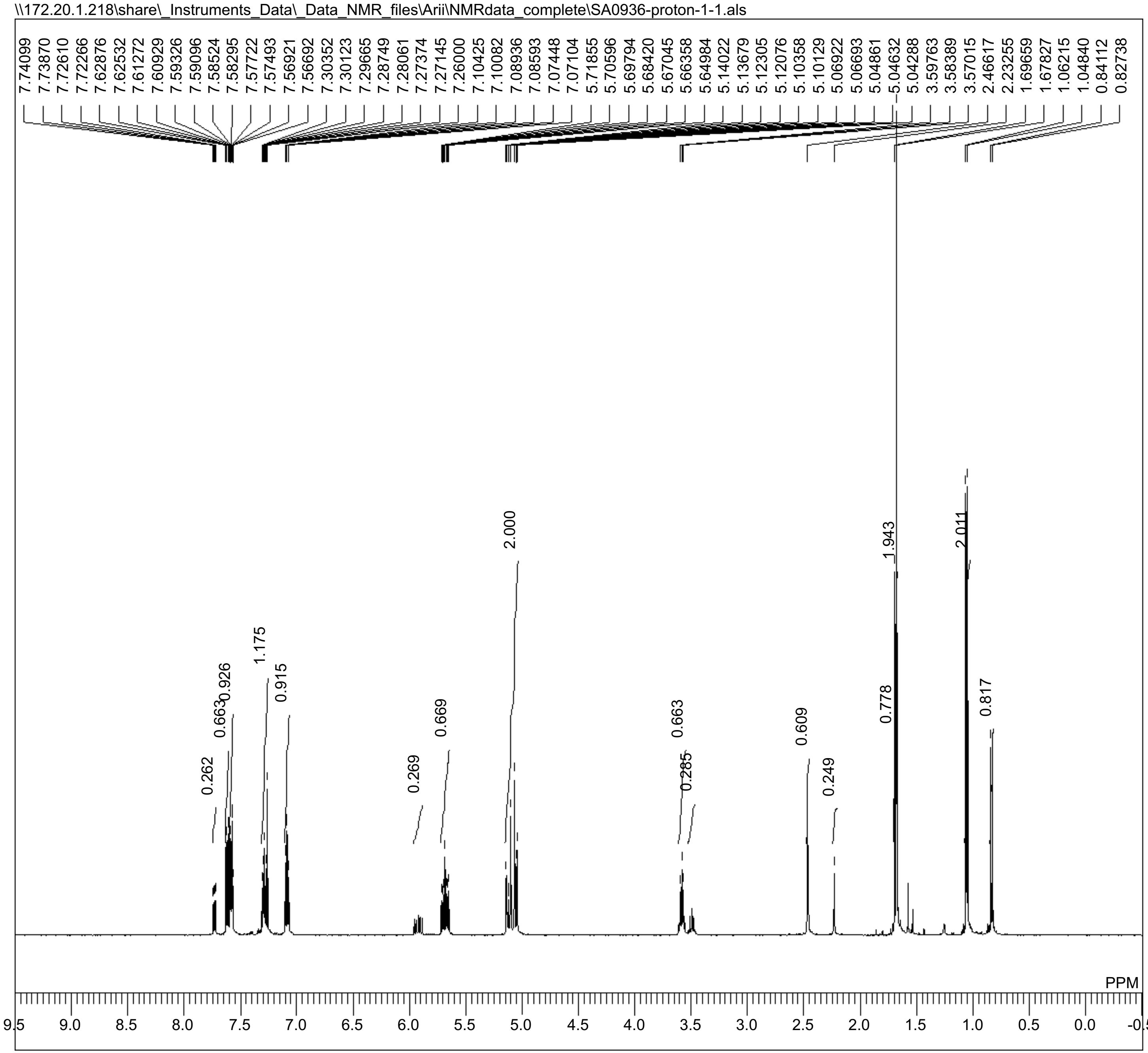

DFILE	SA0936-proton-1-1.als
COMNT	
DATIM	2025-01-16 12:08:53
OBNUC	1H
EXMOD	proton.jxp
OBFRQ	500.16 MHz
OBSET	2.41 KHz
OBFIN	6.01 Hz
POINT	13107
FREQU	7507.51 Hz
SCANS	8
ACQTM	1.7459 sec
PD	5.0000 sec
PW1	5.55 usec
IRNUC	1H
CTEMP	21.3 c
SLVNT	CDCL3
EXREF	7.26 ppm
BF	0.12 Hz
RGAIN	30

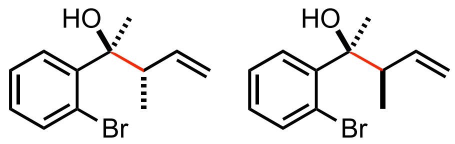

major *minor*

8s

\\172.20.1.218\share_Instruments_Data_Data_NMR_files\Arii\NMRdata_complete\SA0936-carbon-1-1.als

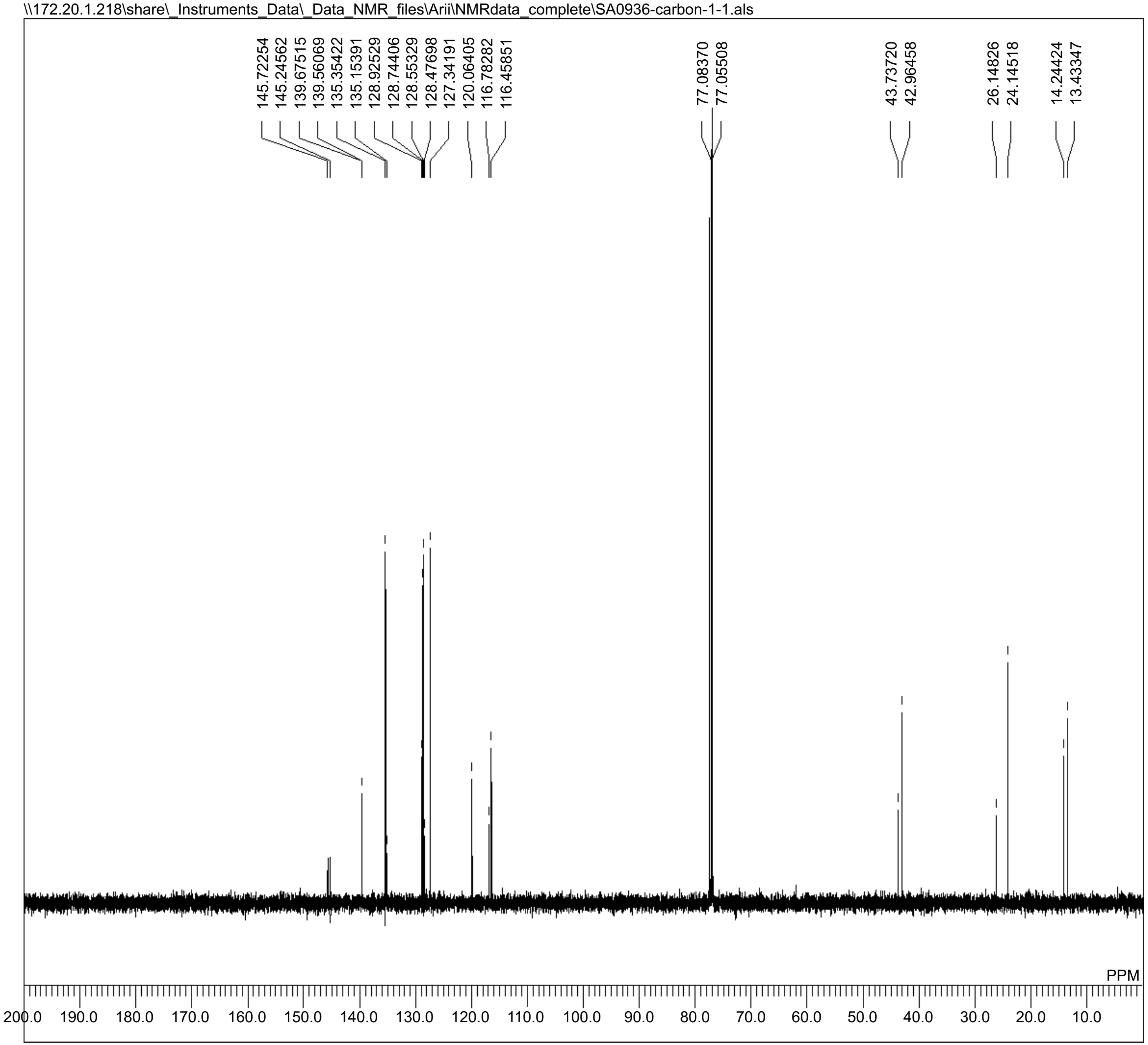

DFILE	SA0936-carbon-1-1.als
COMNT	
DATIM	2025-01-16 12:10:26
OBNUC	13C
EXMOD	carbon.jxp
OBFRQ	125.77 MHz
OBSET	7.87 KHz
OBFIN	4.21 Hz
POINT	26214
FREQU	31446.54 Hz
SCANS	520
ACQTM	0.8336 sec
PD	1.0000 sec
PW1	3.40 usec
IRNUC	1H
CTEMP	21.9 c
SLVNT	CDCL3
EXREF	77.16 ppm
BF	0.12 Hz
RGAIN	60

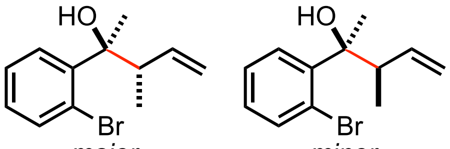

major **8s** *minor*

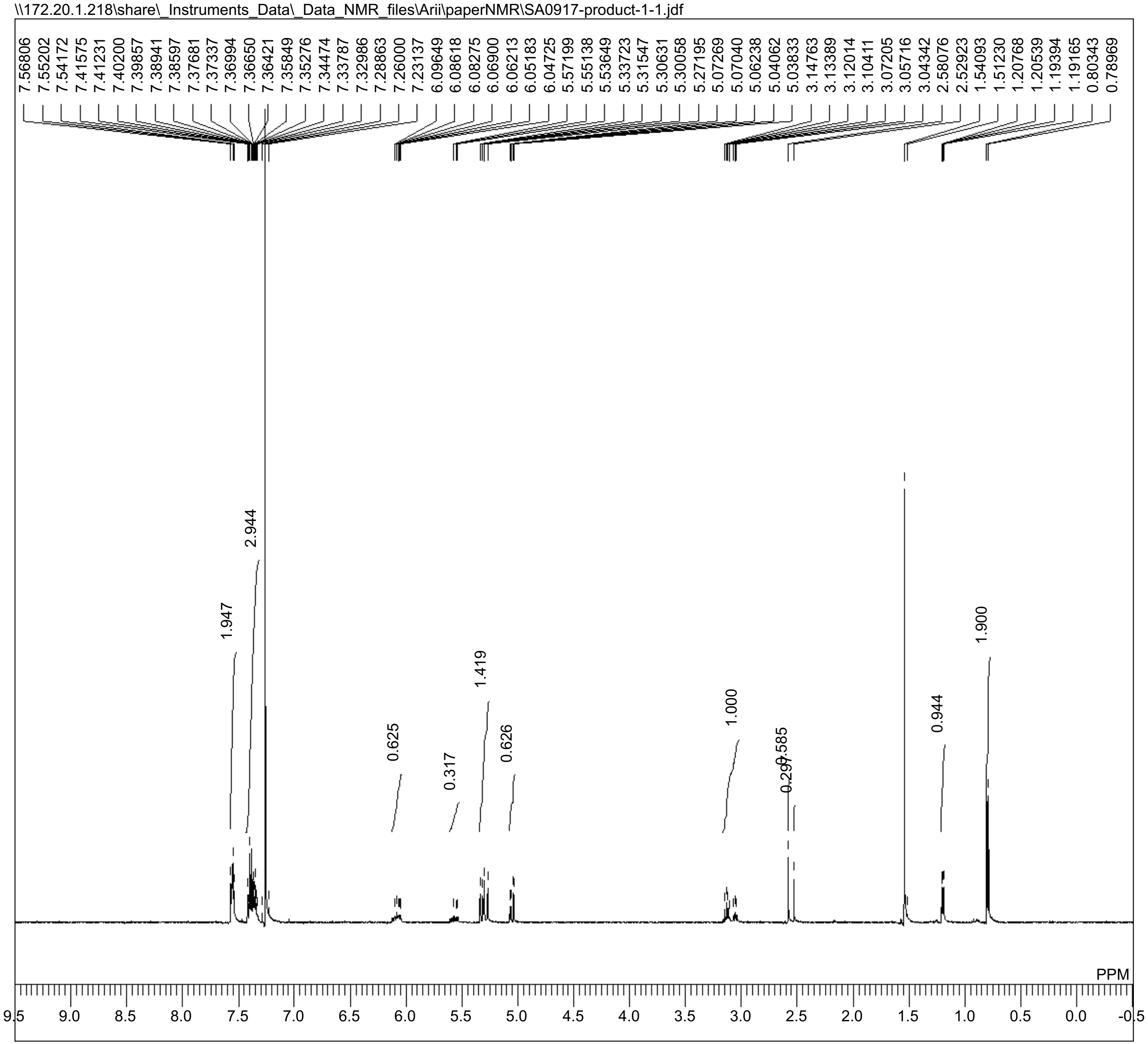

DFILE	SA0917-product-1-1.jdf
COMNT	
DATIM	2024-11-14 19:28:34
OBNUC	1H
EXMOD	proton.jxp
OBFRQ	500.16 MHz
OBSET	2.41 KHz
OBFIN	6.01 Hz
POINT	16384
FREQU	9384.38 Hz
SCANS	8
ACQTM	1.7459 sec
PD	5.0000 sec
PW1	5.55 usec
IRNUC	1H
CTEMP	21.7 c
SLVNT	CDCL3
EXREF	7.26 ppm
BF	0.12 Hz
RGAIN	32

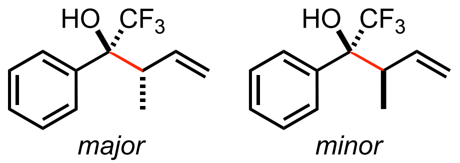

8t

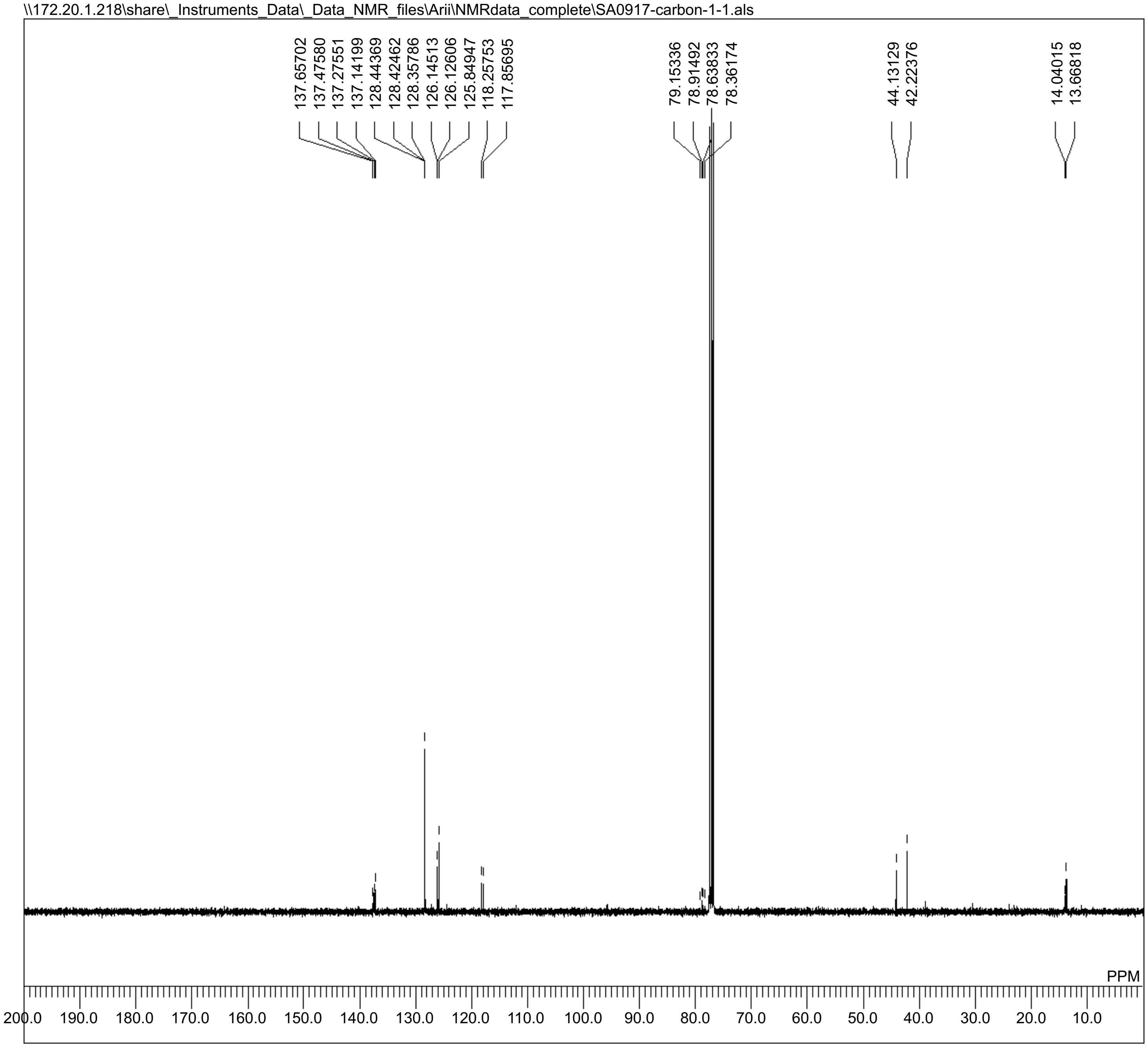

DFILE	SA0917-carbon-1-1.als
COMNT	
DATIM	2025-01-17 13:49:10
OBNUC	13C
EXMOD	carbon.jxp
OBFRQ	98.52 MHz
OBSET	4.64 KHz
OBFIN	8.74 Hz
POINT	26214
FREQU	24630.54 Hz
SCANS	3678
ACQTM	1.0643 sec
PD	2.0000 sec
PW1	2.93 usec
IRNUC	1H
CTEMP	20.7 c
SLVNT	CDCL3
EXREF	77.16 ppm
BF	1.02 Hz
RGAIN	60

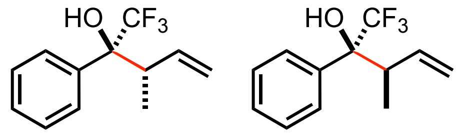

major *minor*

8t

\\172.20.1.218\share_Instruments_Data_Data_NMR_files\Arii\NMRdata_complete\SA0917-fluorine-1-4.jdf

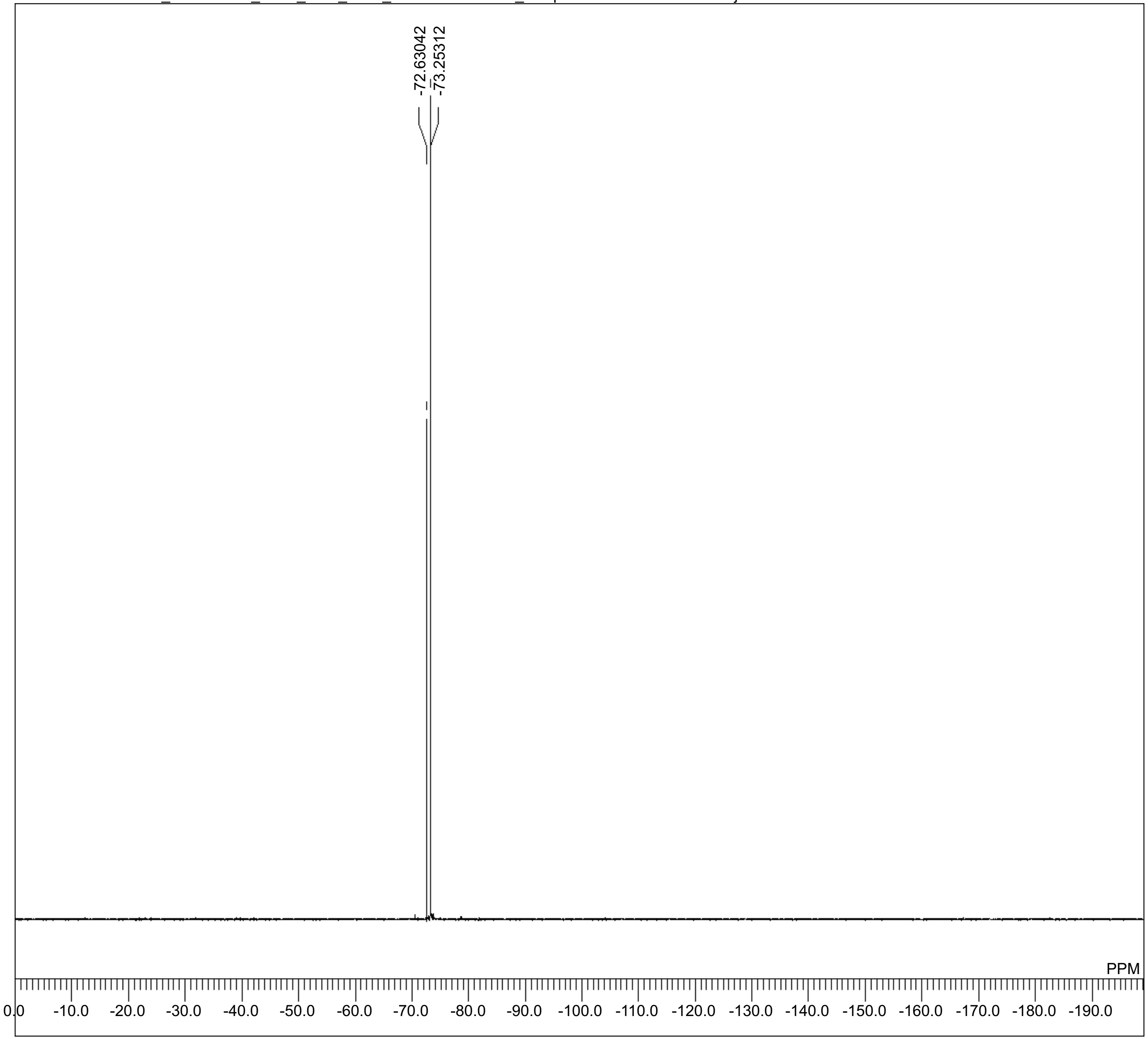

DFILE	SA0917-fluorine-1-4.jdf
COMNT	
DATIM	2025-01-17 01:48:38
OBNUC	19F
EXMOD	single_pulse.jxp
OBFRQ	368.64 MHz
OBSET	7.63 KHz
OBFIN	2.85 Hz
POINT	32768
FREQU	147492.62 Hz
SCANS	8
ACQTM	0.2222 sec
PD	5.0000 sec
PW1	4.10 usec
IRNUC	19F
CTEMP	20.2 c
SLVNT	CDCL3
EXREF	-164.90 ppm
BF	1.02 Hz
RGAIN	50

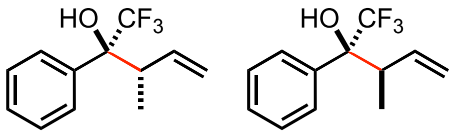

major *minor*

8t

\\172.20.1.218\share_Instruments_Data_Data_NMR_files\Arii\NMRdata_complete\SA0974-proton-1-1.als

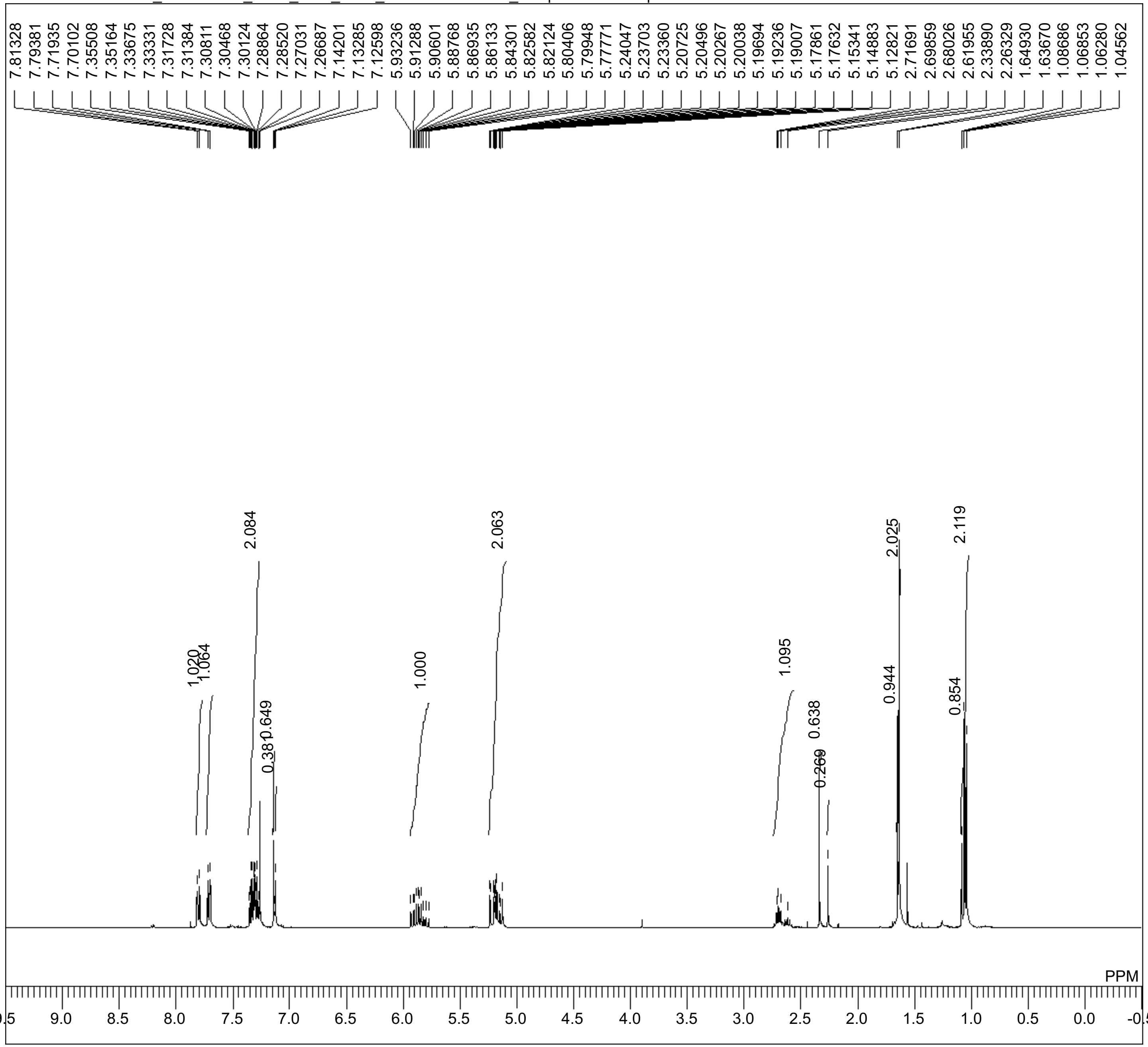

DFILE	SA0974-proton-1-1.als
COMNT	
DATIM	2025-01-25 21:29:04
OBNUC	1H
EXMOD	proton.jxp
OBFRQ	391.78 MHz
OBSET	8.51 KHz
OBFIN	3.34 Hz
POINT	13107
FREQU	5882.35 Hz
SCANS	8
ACQTM	2.2282 sec
PD	4.0000 sec
PW1	6.30 usec
IRNUC	1H
CTEMP	20.5 c
SLVNT	CDCL3
EXREF	7.26 ppm
BF	0.12 Hz
RGAIN	44

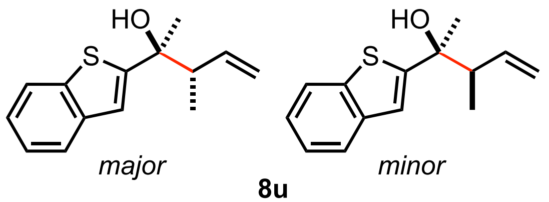

8u

\\172.20.1.218\share_Instruments_Data_Data_NMR_files\Arii\NMRdata_complete\SA0974-carbon-1-1.als

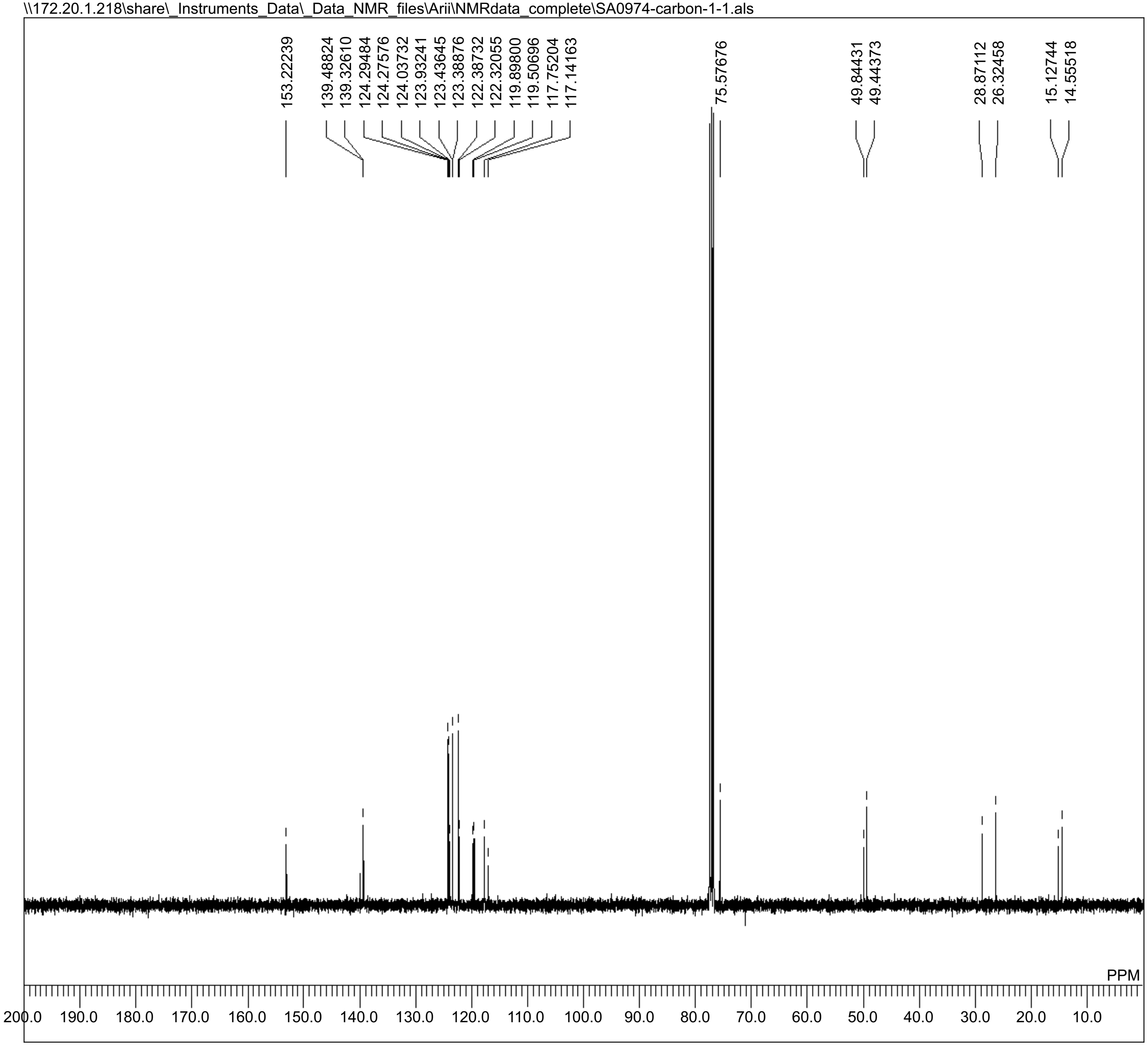

DFILE	SA0974-carbon-1-1.als
COMNT	
DATIM	2025-01-25 21:30:38
OBNUC	13C
EXMOD	carbon.jxp
OBFRQ	98.52 MHz
OBSET	4.64 KHz
OBFIN	8.74 Hz
POINT	26214
FREQU	24630.54 Hz
SCANS	1181
ACQTM	1.0643 sec
PD	2.0000 sec
PW1	2.93 usec
IRNUC	1H
CTEMP	20.6 c
SLVNT	CDCL3
EXREF	77.16 ppm
BF	0.12 Hz
RGAIN	60

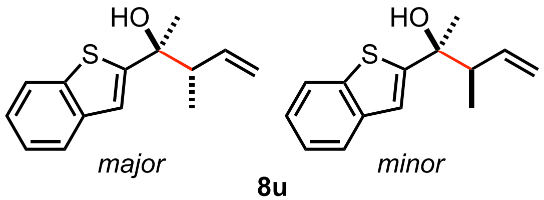

major **8u** *minor*

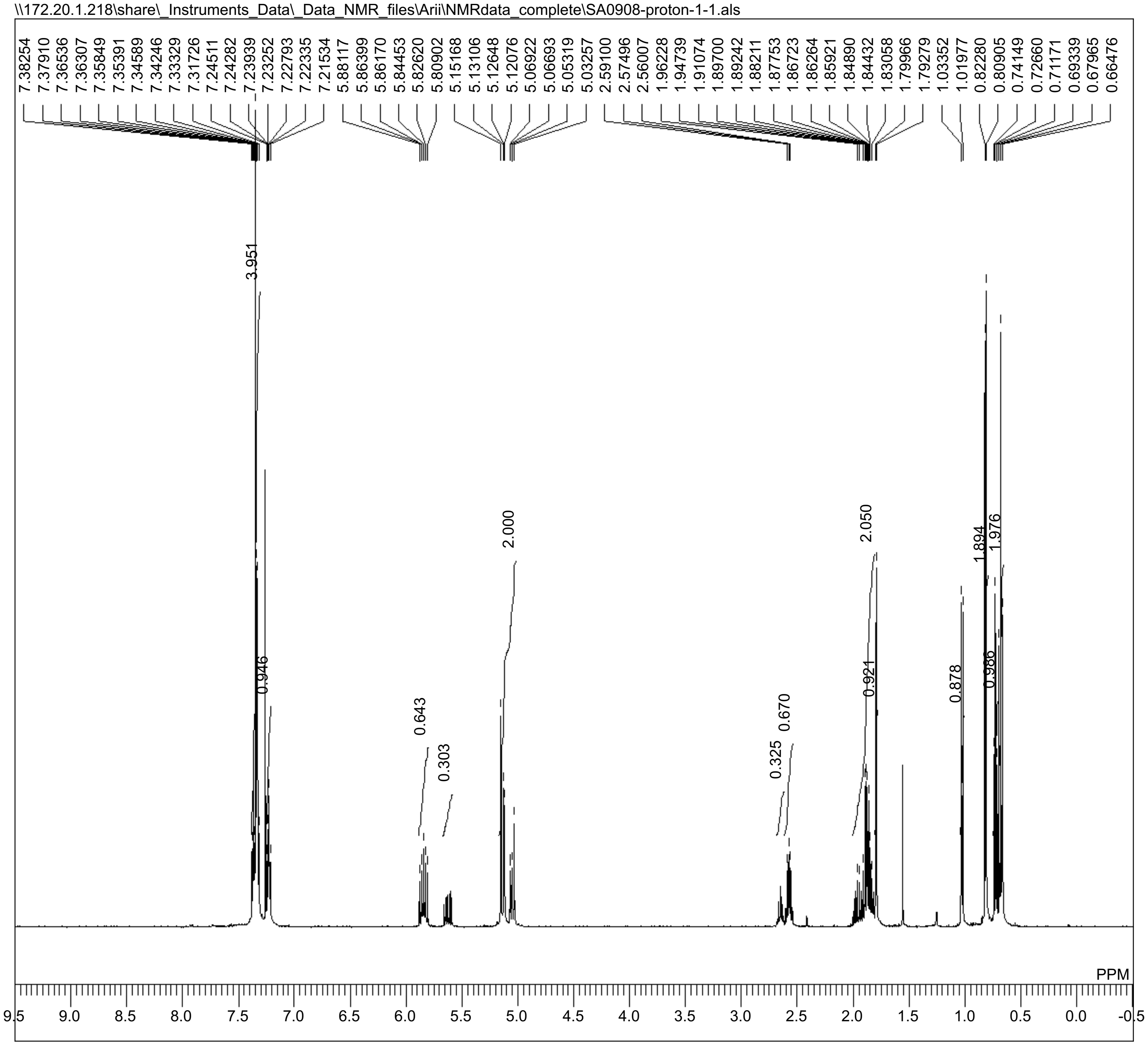

DFILE	SA0908-proton-1-1.als
COMNT	
DATIM	2025-01-16 13:43:59
OBNUC	1H
EXMOD	proton.jxp
OBFRQ	500.16 MHz
OBSET	2.41 KHz
OBFIN	6.01 Hz
POINT	13107
FREQU	7507.51 Hz
SCANS	8
ACQTM	1.7459 sec
PD	5.0000 sec
PW1	5.55 usec
IRNUC	1H
CTEMP	21.7 c
SLVNT	CDCL3
EXREF	7.26 ppm
BF	0.12 Hz
RGAIN	30

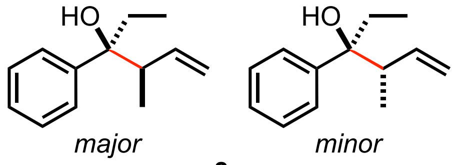

8v

\\172.20.1.218\share_Instruments_Data_Data_NMR_files\Arii\NMRdata_complete\SA0908-carbon-1-1.als

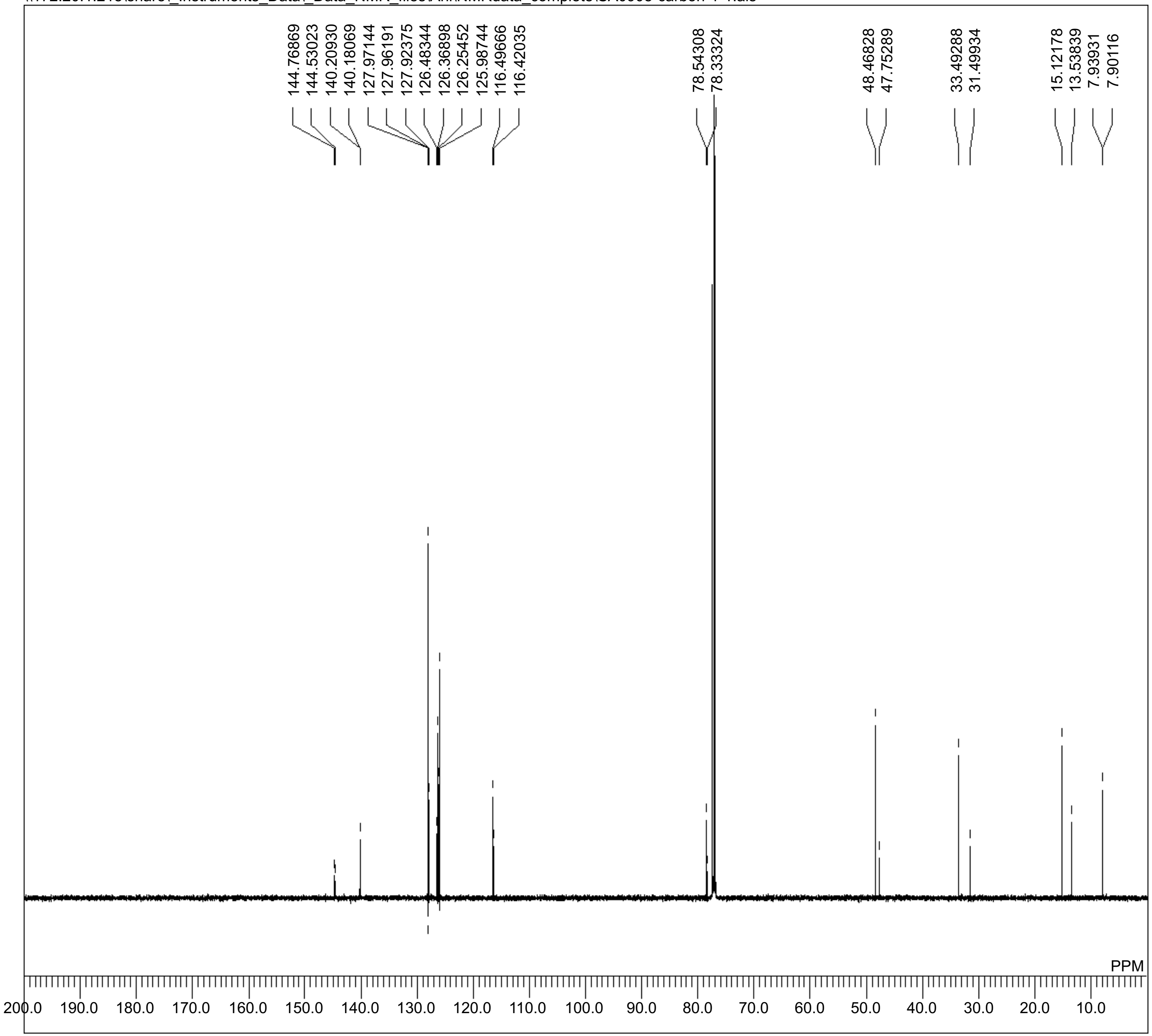

DFILE	SA0908-carbon-1-1.als
COMNT	
DATIM	2025-01-16 13:45:31
OBNUC	13C
EXMOD	carbon.jxp
OBFRQ	125.77 MHz
OBSET	7.87 KHz
OBFIN	4.21 Hz
POINT	26214
FREQU	31446.54 Hz
SCANS	7666
ACQTM	0.8336 sec
PD	1.0000 sec
PW1	3.40 usec
IRNUC	1H
CTEMP	21.7 c
SLVNT	CDCL3
EXREF	77.16 ppm
BF	0.12 Hz
RGAIN	60

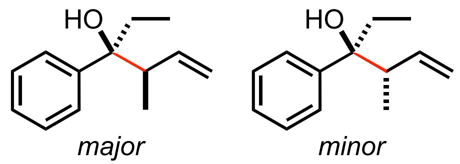

8v

\\172.20.1.218\share_Instruments_Data_Data_NMR_files\Arii\NMRdata_complete\SA0940-proton-1-1.als

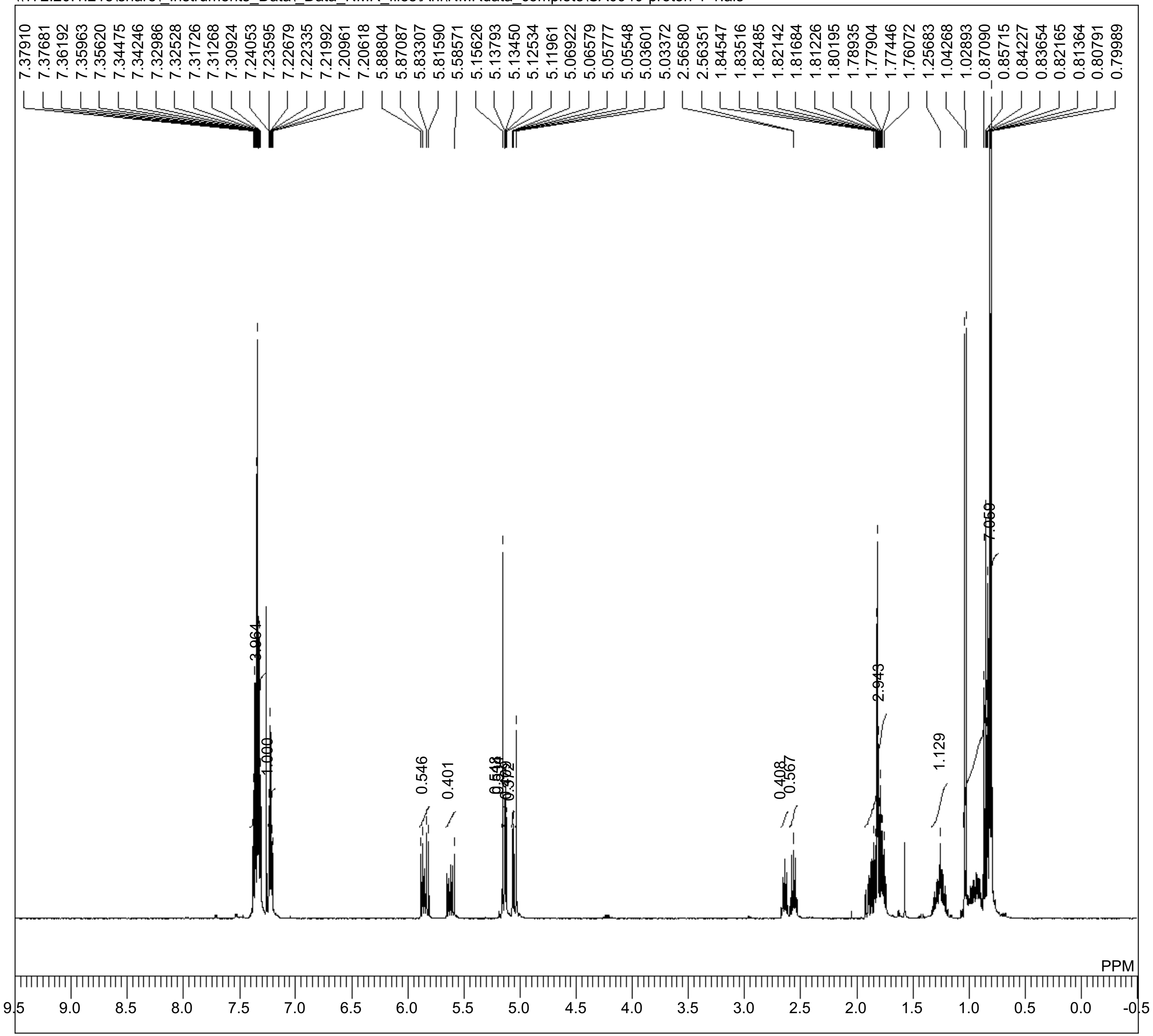

DFILE	SA0940-proton-1-1.als
COMNT	
DATIM	2025-01-16 12:30:29
OBNUC	1H
EXMOD	proton.jxp
OBFRQ	500.16 MHz
OBSET	2.41 KHz
OBFIN	6.01 Hz
POINT	13107
FREQU	7507.51 Hz
SCANS	8
ACQTM	1.7459 sec
PD	5.0000 sec
PW1	5.55 usec
IRNUC	1H
CTEMP	21.6 c
SLVNT	CDCL3
EXREF	7.26 ppm
BF	0.12 Hz
RGAIN	30

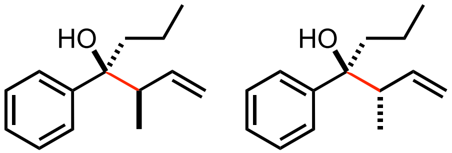

major *minor*

8w

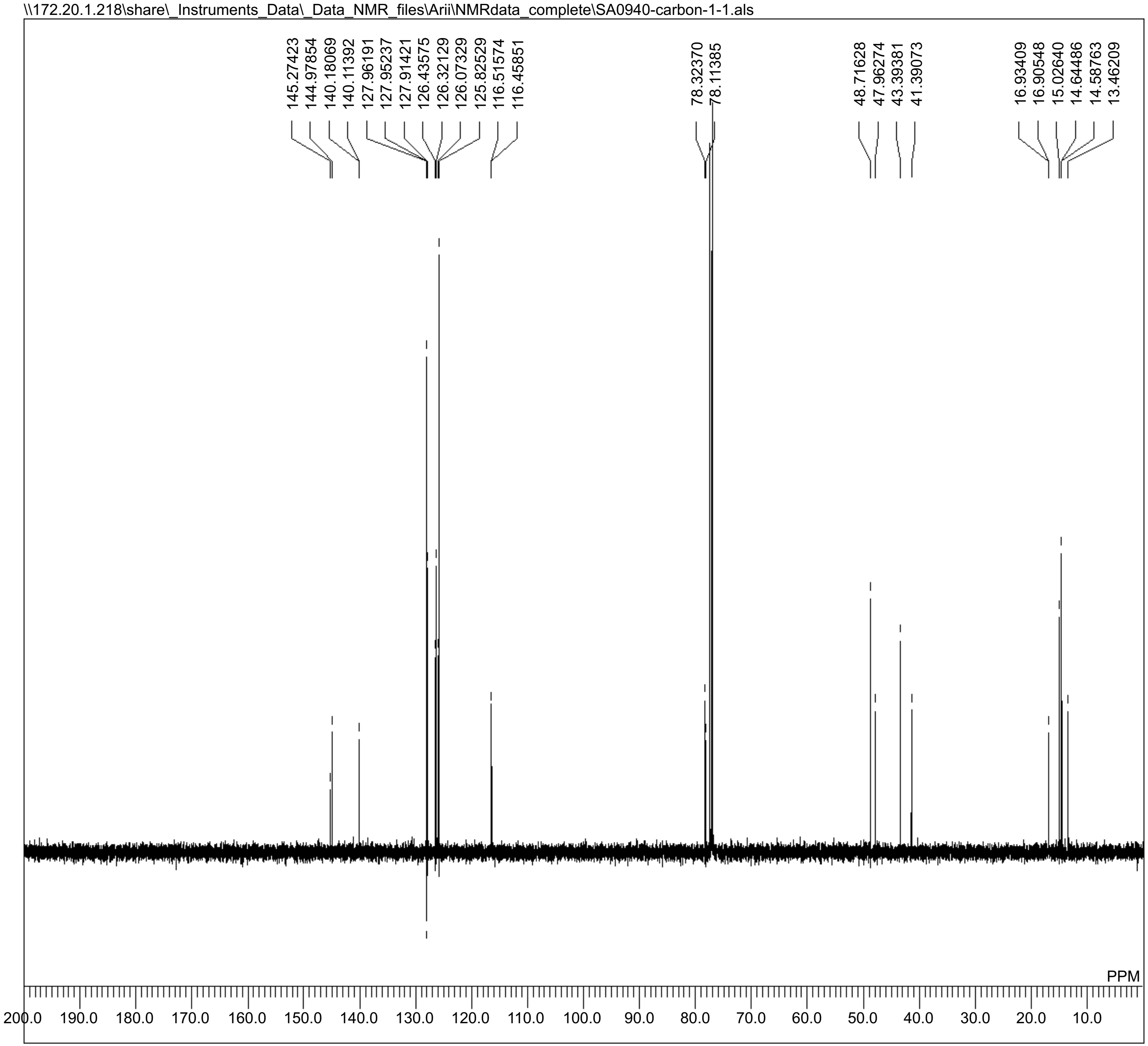

DFILE	SA0940-carbon-1-1.als
COMNT	
DATIM	2025-01-16 12:32:02
OBNUC	13C
EXMOD	carbon.jxp
OBFRQ	125.77 MHz
OBSET	7.87 KHz
OBFIN	4.21 Hz
POINT	26214
FREQU	31446.54 Hz
SCANS	470
ACQTM	0.8336 sec
PD	1.0000 sec
PW1	3.40 usec
IRNUC	1H
CTEMP	22.0 c
SLVNT	CDCL3
EXREF	77.16 ppm
BF	0.12 Hz
RGAIN	60

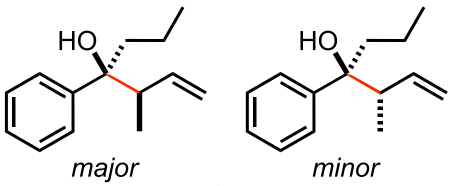

8w

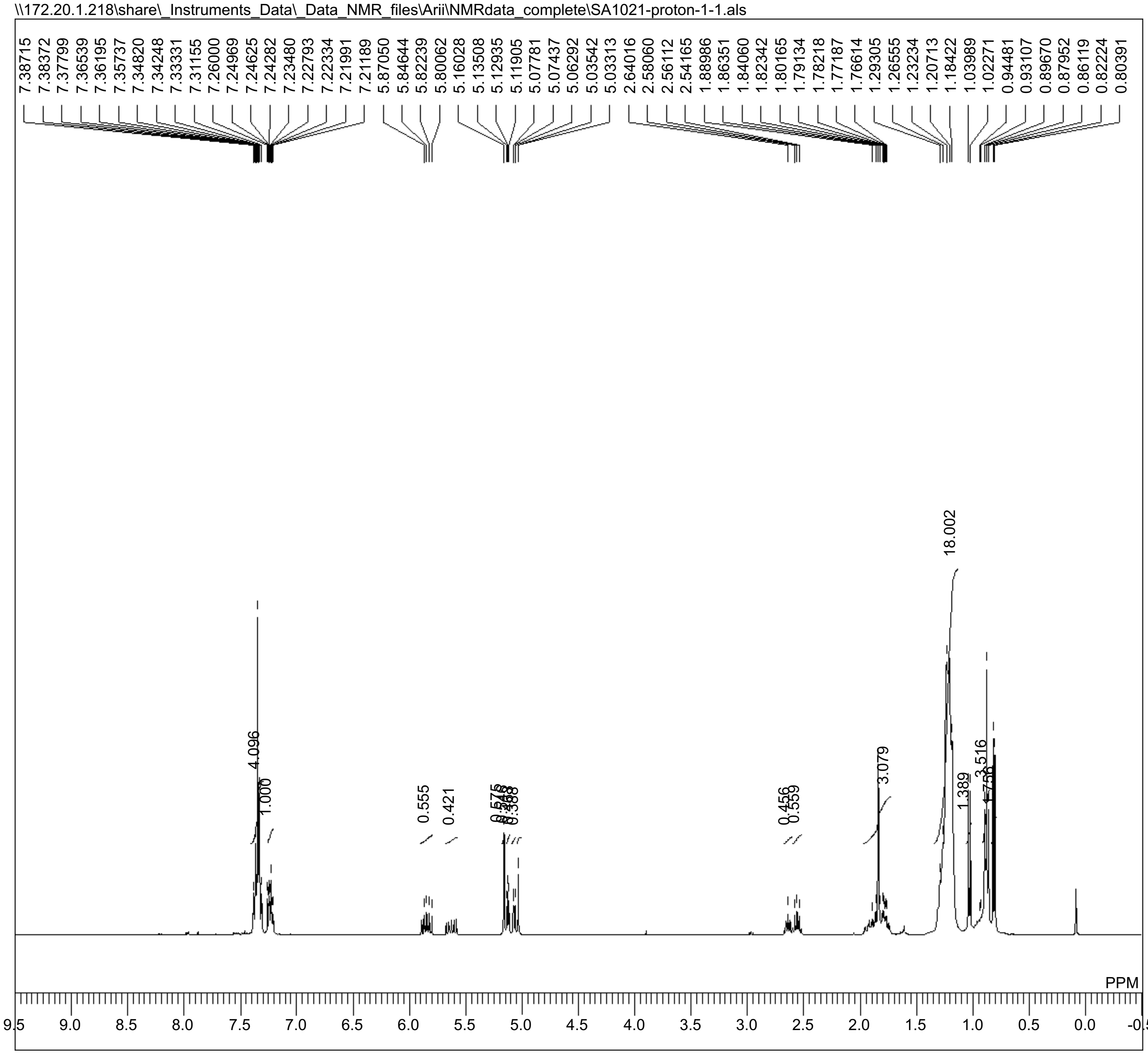

DFILE	SA1021-proton-1-1.als
COMNT	
DATIM	2025-01-11 03:18:07
OBNUC	1H
EXMOD	proton.jxp
OBFRQ	391.78 MHz
OBSET	8.51 KHz
OBFIN	3.34 Hz
POINT	13107
FREQU	5882.35 Hz
SCANS	8
ACQTM	2.2282 sec
PD	4.0000 sec
PW1	6.30 usec
IRNUC	1H
CTEMP	20.1 c
SLVNT	CDCL3
EXREF	7.26 ppm
BF	2.92 Hz
RGAIN	28

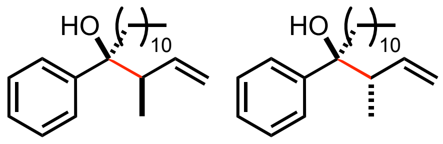

major **8x** *minor*

\\172.20.1.218\share_Instruments_Data_Data_NMR_files\Arii\NMRdata_complete\SA1021-carbon-1-1.als

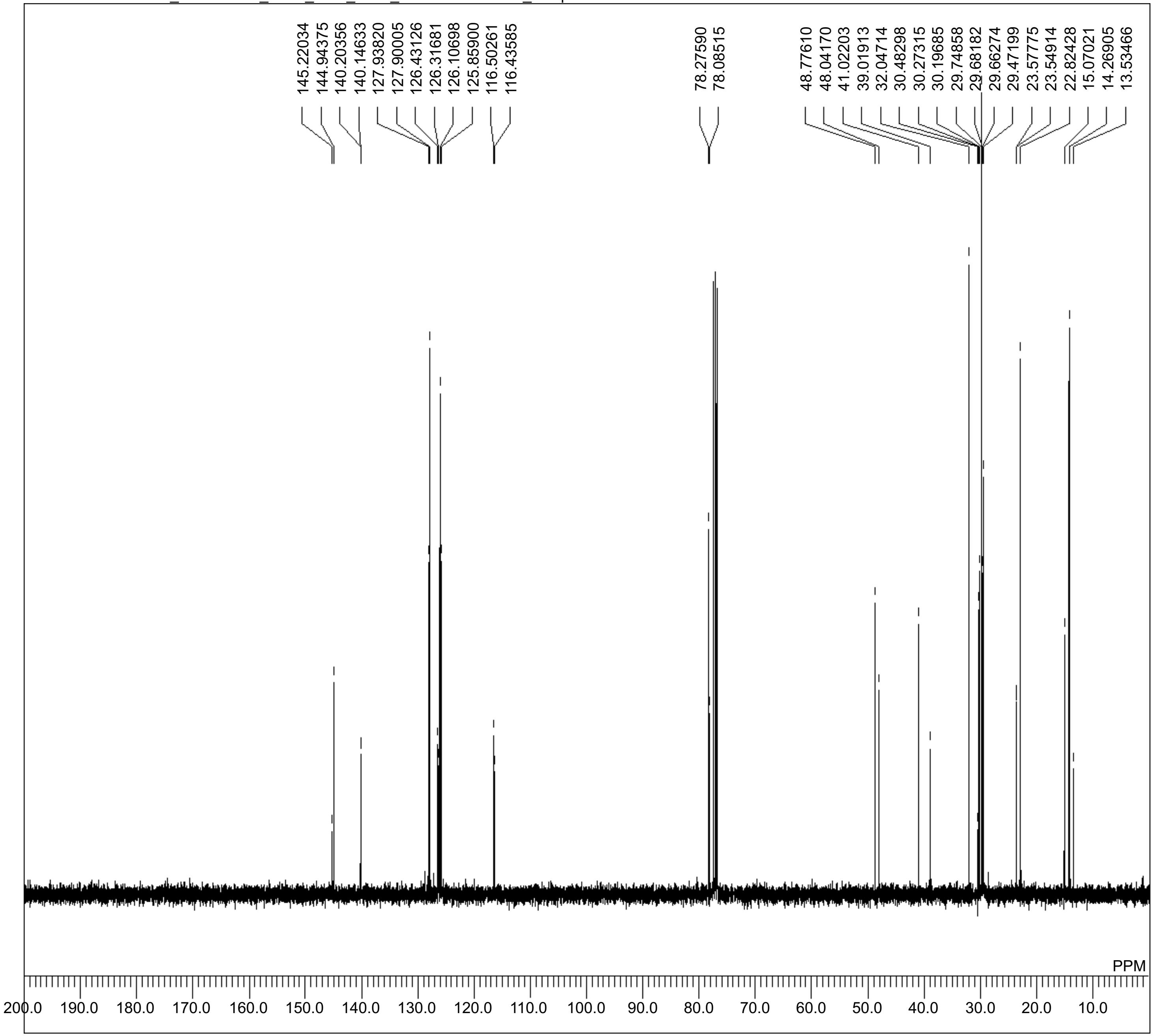

DFILE	SA1021-carbon-1-1.als
COMNT	
DATIM	2025-01-11 03:20:58
OBNUC	13C
EXMOD	carbon.jxp
OBFRQ	98.52 MHz
OBSET	4.64 KHz
OBFIN	8.74 Hz
POINT	26214
FREQU	24630.54 Hz
SCANS	308
ACQTM	1.0643 sec
PD	2.0000 sec
PW1	2.93 usec
IRNUC	1H
CTEMP	20.3 c
SLVNT	CDCL3
EXREF	77.16 ppm
BF	2.92 Hz
RGAIN	60

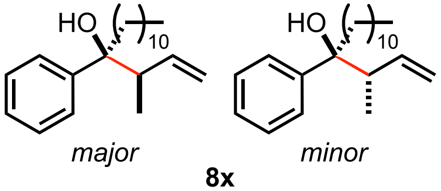

8x

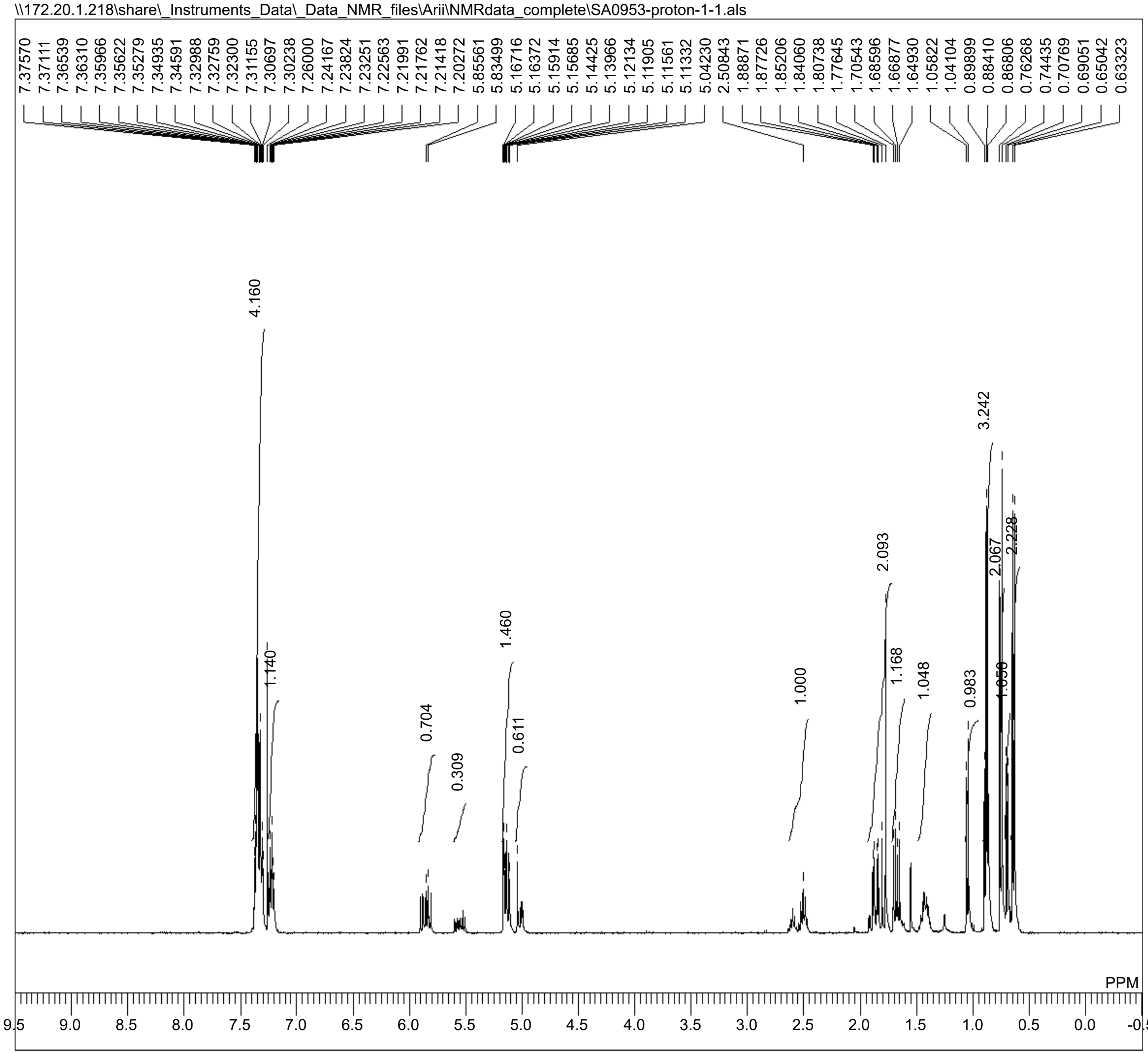

DFILE	SA0953-proton-1-1.als
COMNT	
DATIM	2025-01-10 16:39:04
OBNUC	1H
EXMOD	proton.jxp
OBFRQ	391.78 MHz
OBSET	8.51 KHz
OBFIN	3.34 Hz
POINT	13107
FREQU	5882.35 Hz
SCANS	8
ACQTM	2.2282 sec
PD	4.0000 sec
PW1	6.30 usec
IRNUC	1H
CTEMP	20.7 c
SLVNT	CDCL3
EXREF	7.26 ppm
BF	0.12 Hz
RGAIN	44

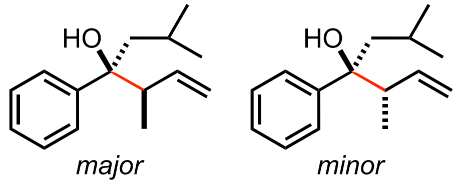

8y

\\172.20.1.218\share_Instruments_Data_Data_NMR_files\Arii\NMRdata_complete\SA0953-carbon-1-1.als

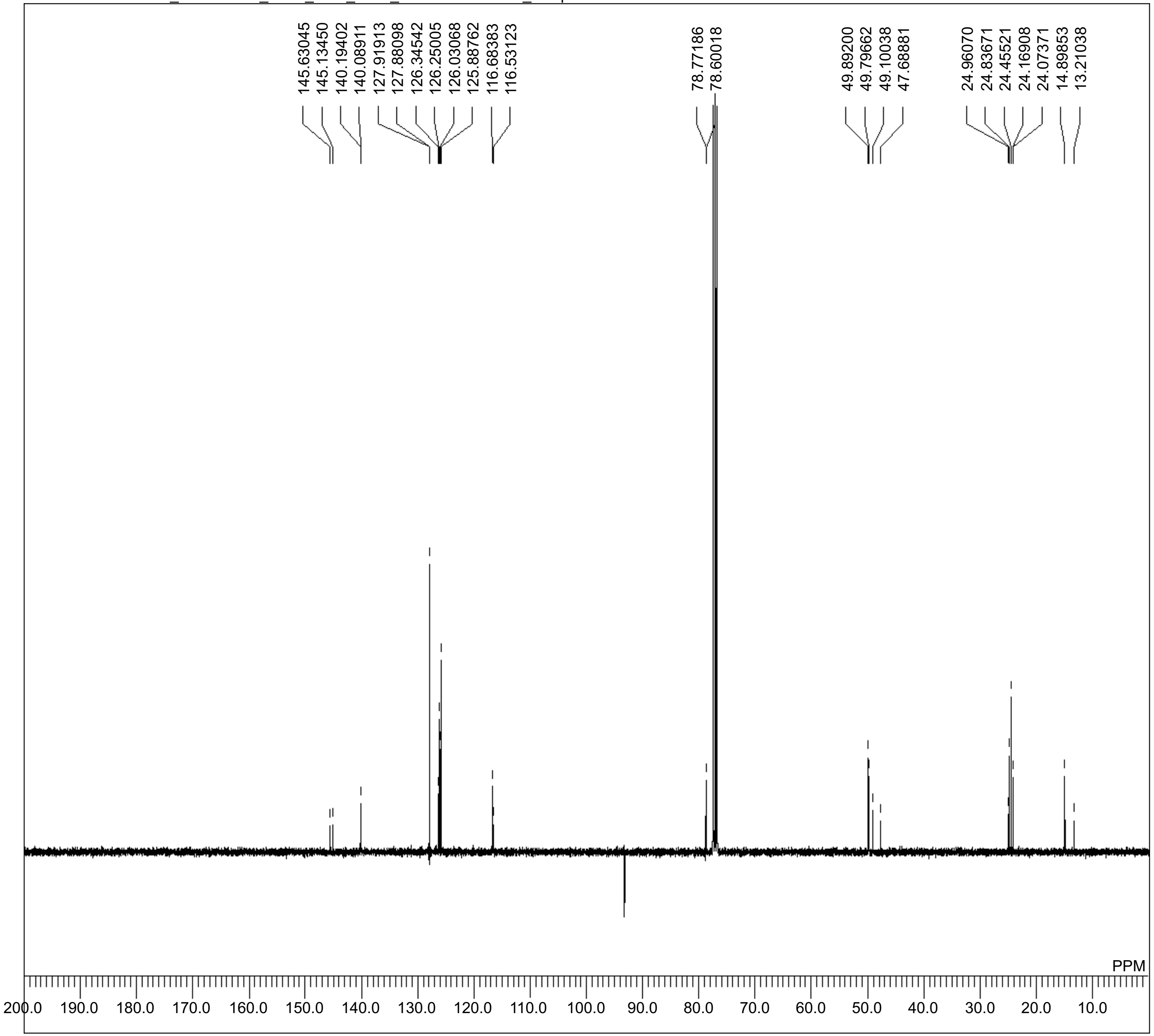

DFILE	SA0953-carbon-1-1.als
COMNT	
DATIM	2025-01-10 16:44:52
OBNUC	13C
EXMOD	carbon.jxp
OBFRQ	98.52 MHz
OBSET	4.64 KHz
OBFIN	8.74 Hz
POINT	26214
FREQU	24630.54 Hz
SCANS	4044
ACQTM	1.0643 sec
PD	2.0000 sec
PW1	2.93 usec
IRNUC	1H
CTEMP	20.7 c
SLVNT	CDCL3
EXREF	77.16 ppm
BF	0.12 Hz
RGAIN	60

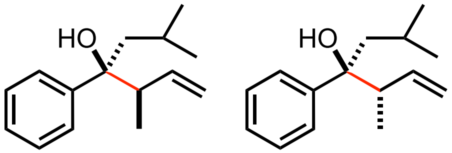

major *minor*

8y

\\172.20.1.218\share_Instruments_Data_Data_NMR_files\Arii\NMRdata_complete\SA1042-proton-1-1.als

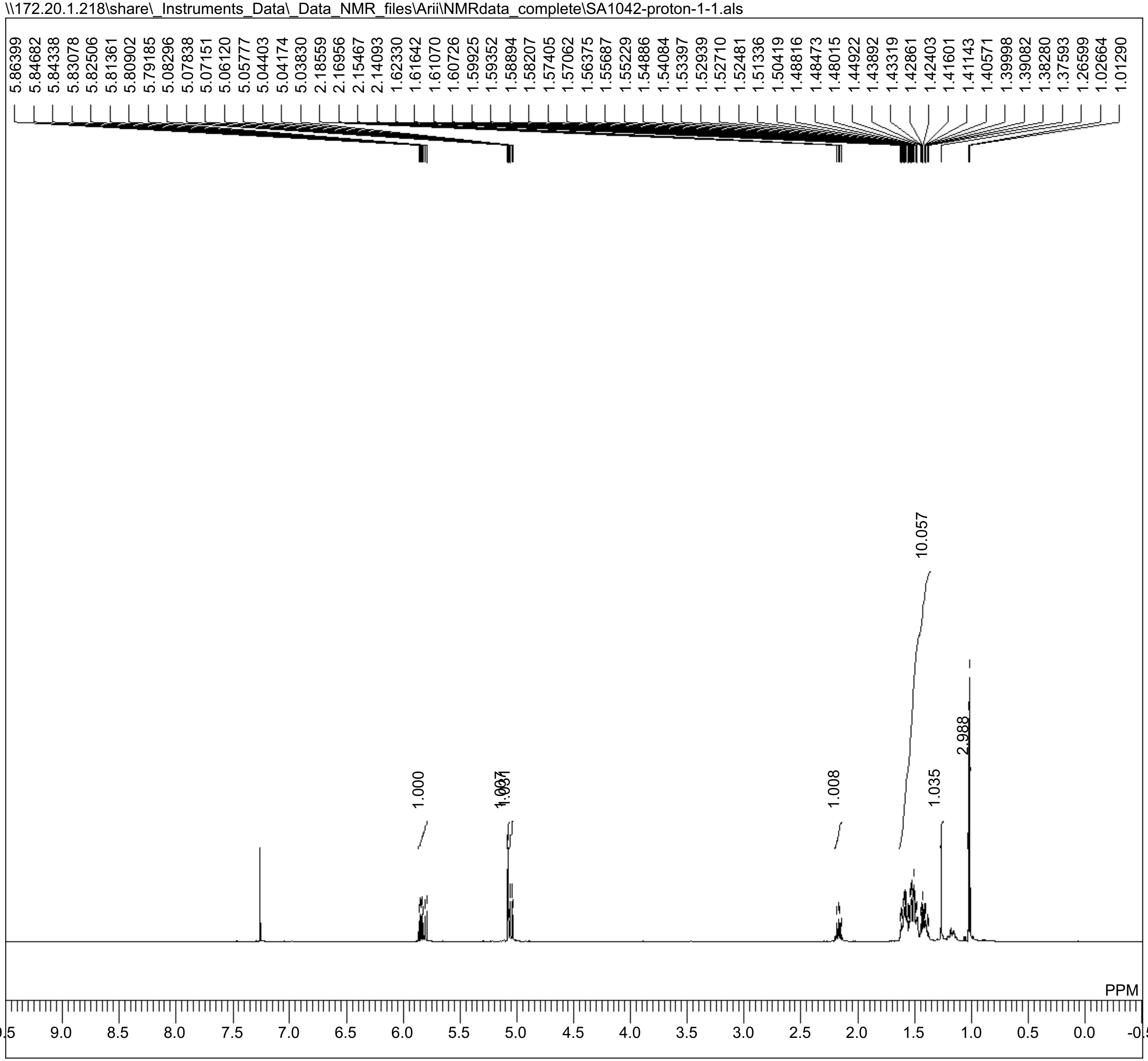

DFILE	SA1042-proton-1-1.als
COMNT	
DATIM	2025-01-17 22:12:44
OBNUC	1H
EXMOD	proton.jxp
OBFRQ	500.16 MHz
OBSET	2.41 KHz
OBFIN	6.01 Hz
POINT	13107
FREQU	7507.51 Hz
SCANS	8
ACQTM	1.7459 sec
PD	5.0000 sec
PW1	5.55 usec
IRNUC	1H
CTEMP	21.2 c
SLVNT	CDCL3
EXREF	7.26 ppm
BF	1.20 Hz
RGAIN	30

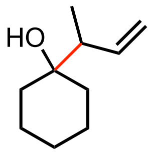

8z

\\172.20.1.218\share_Instruments_Data_Data_NMR_files\Arii\NMRdata_complete\SA1042-carbon-1-1.als

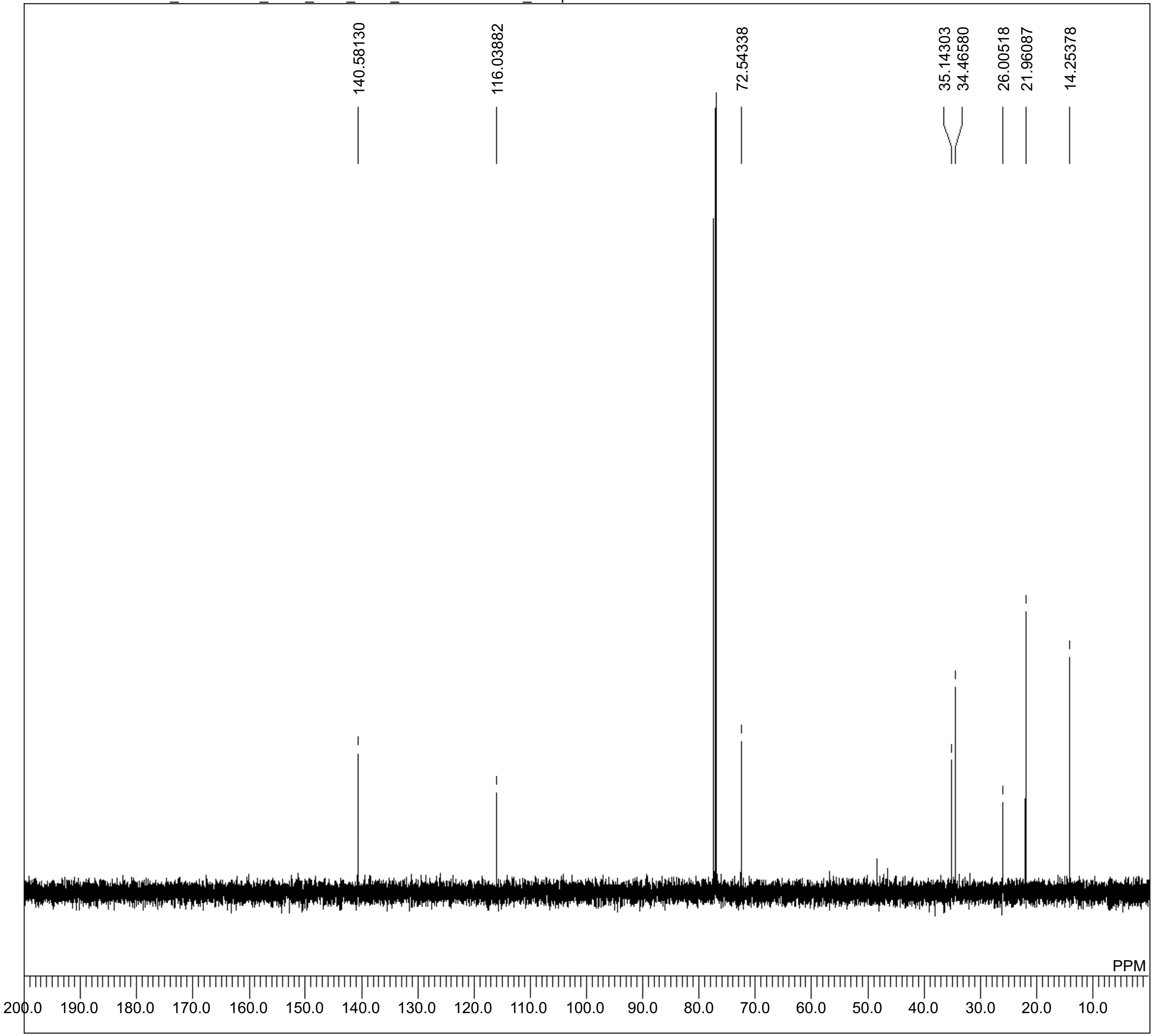

DFILE	SA1042-carbon-1-1.als
COMNT	
DATIM	2025-01-17 22:15:53
OBNUC	13C
EXMOD	carbon.jxp
OBFRQ	125.77 MHz
OBSET	7.87 KHz
OBFIN	4.21 Hz
POINT	26214
FREQU	31446.54 Hz
SCANS	241
ACQTM	0.8336 sec
PD	1.0000 sec
PW1	3.40 usec
IRNUC	1H
CTEMP	22.0 c
SLVNT	CDCL3
EXREF	77.16 ppm
BF	1.20 Hz
RGAIN	60

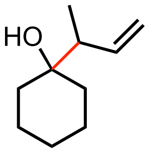

8z

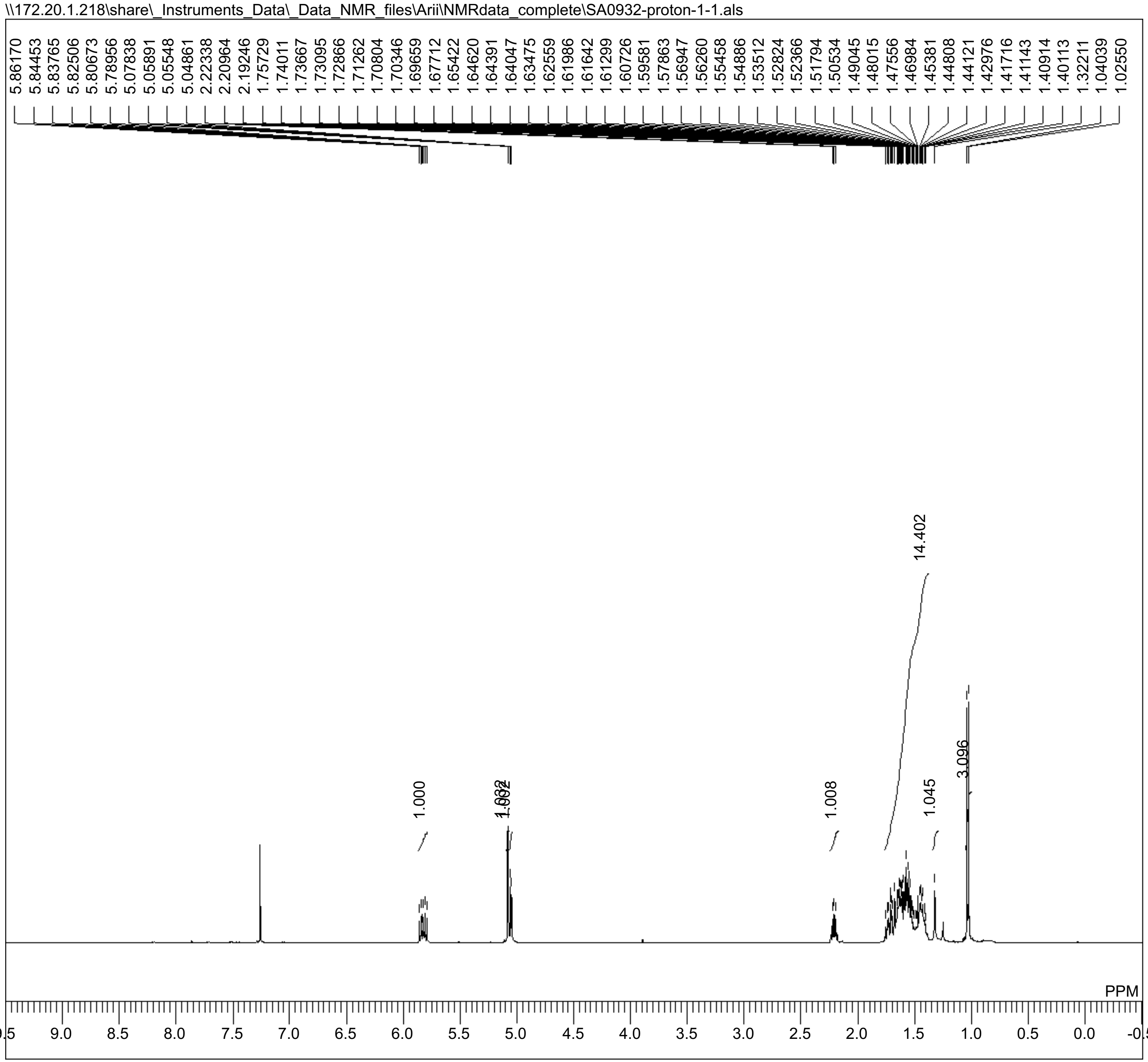

DFILE	SA0932-proton-1-1.als
COMNT	
DATIM	2024-12-06 13:25:29
OBNUC	1H
EXMOD	proton.jxp
OBFRQ	500.16 MHz
OBSET	2.41 KHz
OBFIN	6.01 Hz
POINT	13107
FREQU	7507.51 Hz
SCANS	8
ACQTM	1.7459 sec
PD	5.0000 sec
PW1	5.55 usec
IRNUC	1H
CTEMP	21.6 c
SLVNT	CDCL3
EXREF	7.26 ppm
BF	0.12 Hz
RGAIN	30

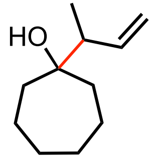

8aa

\\172.20.1.218\share_Instruments_Data_Data_NMR_files\Arii\NMRdata_complete\SA0932-carbon-1-1.als

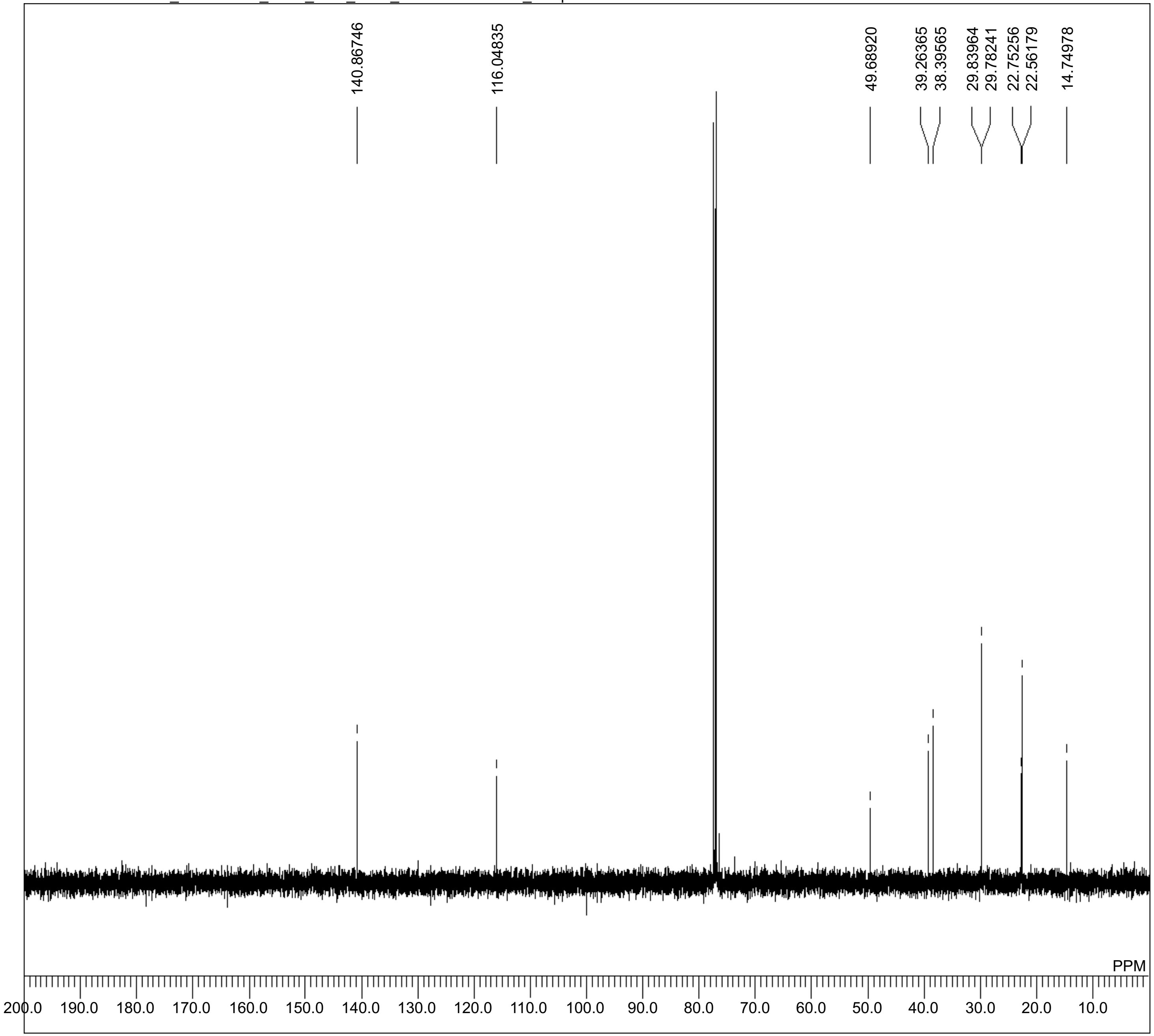

DFILE	SA0932-carbon-1-1.als
COMNT	
DATIM	2024-12-06 13:12:30
OBNUC	13C
EXMOD	carbon.jxp
OBFRQ	125.77 MHz
OBSET	7.87 KHz
OBFIN	4.21 Hz
POINT	26214
FREQU	31446.54 Hz
SCANS	238
ACQTM	0.8336 sec
PD	1.0000 sec
PW1	3.40 usec
IRNUC	1H
CTEMP	22.2 c
SLVNT	CDCL3
EXREF	77.16 ppm
BF	0.12 Hz
RGAIN	60

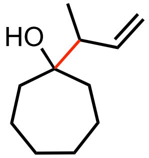

8aa

\\172.20.1.218\share_Instruments_Data_Data_NMR_files\Arii\NMRdata_complete\SA1023-proton-1-1.als

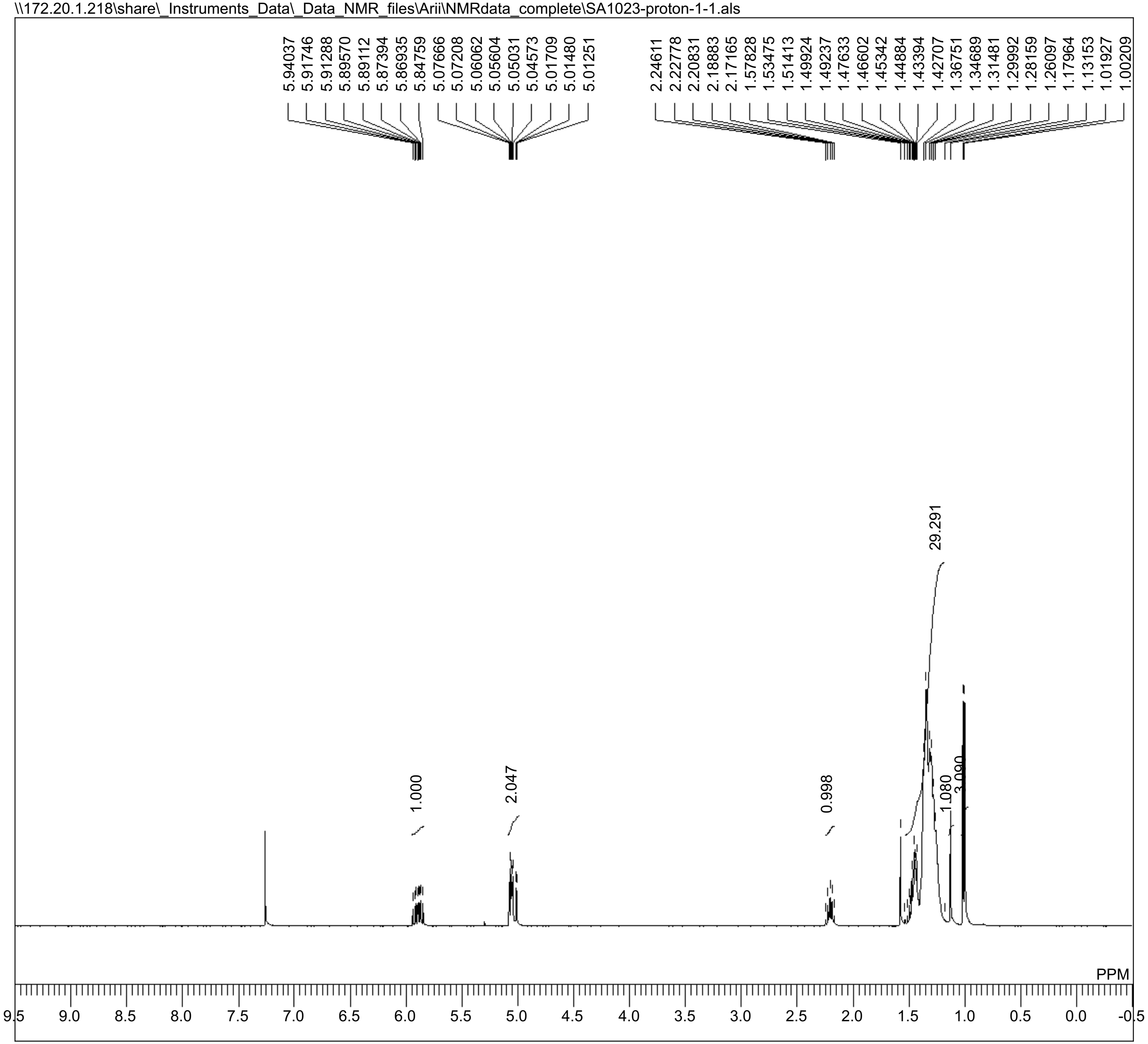

DFILE	SA1023-proton-1-1.als
COMNT	
DATIM	2025-01-25 04:40:03
OBNUC	1H
EXMOD	proton.jxp
OBFRQ	391.78 MHz
OBSET	8.51 KHz
OBFIN	3.34 Hz
POINT	13107
FREQU	5882.35 Hz
SCANS	8
ACQTM	2.2282 sec
PD	4.0000 sec
PW1	6.30 usec
IRNUC	1H
CTEMP	20.4 c
SLVNT	CDCL3
EXREF	7.26 ppm
BF	0.12 Hz
RGAIN	40

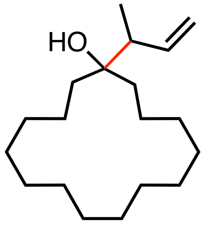

8ab

\\172.20.1.218\share_Instruments_Data_Data_NMR_files\Arii\NMRdata_complete\SA1023-carbon-1-1.als

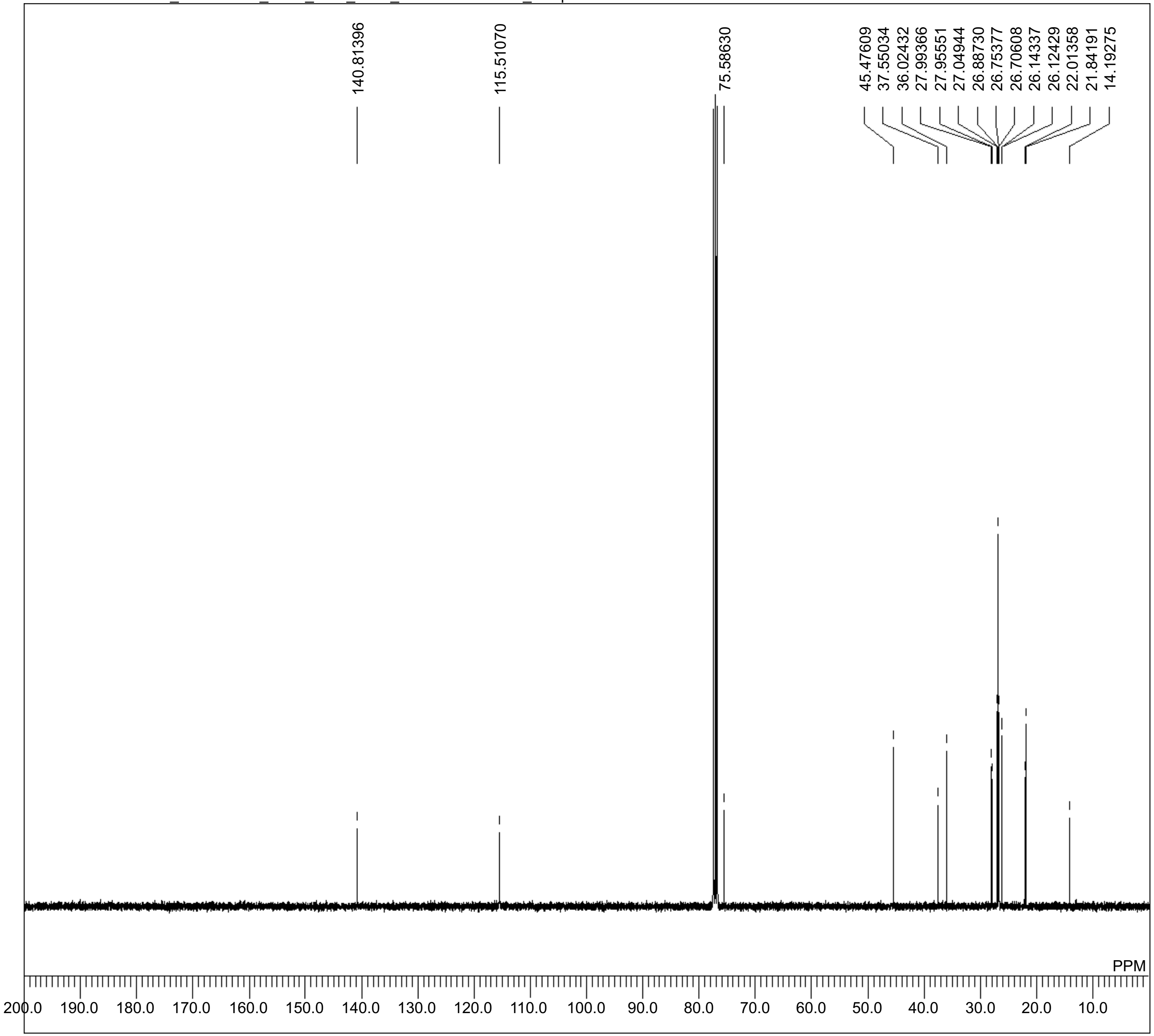

DFILE	SA1023-carbon-1-1.als
COMNT	
DATIM	2025-01-25 04:41:36
OBNUC	13C
EXMOD	carbon.jxp
OBFRQ	98.52 MHz
OBSET	4.64 KHz
OBFIN	8.74 Hz
POINT	26214
FREQU	24630.54 Hz
SCANS	2847
ACQTM	1.0643 sec
PD	2.0000 sec
PW1	2.93 usec
IRNUC	1H
CTEMP	20.4 c
SLVNT	CDCL3
EXREF	77.16 ppm
BF	0.12 Hz
RGAIN	58

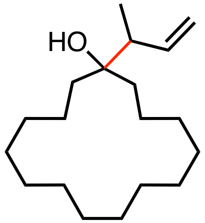

8ab

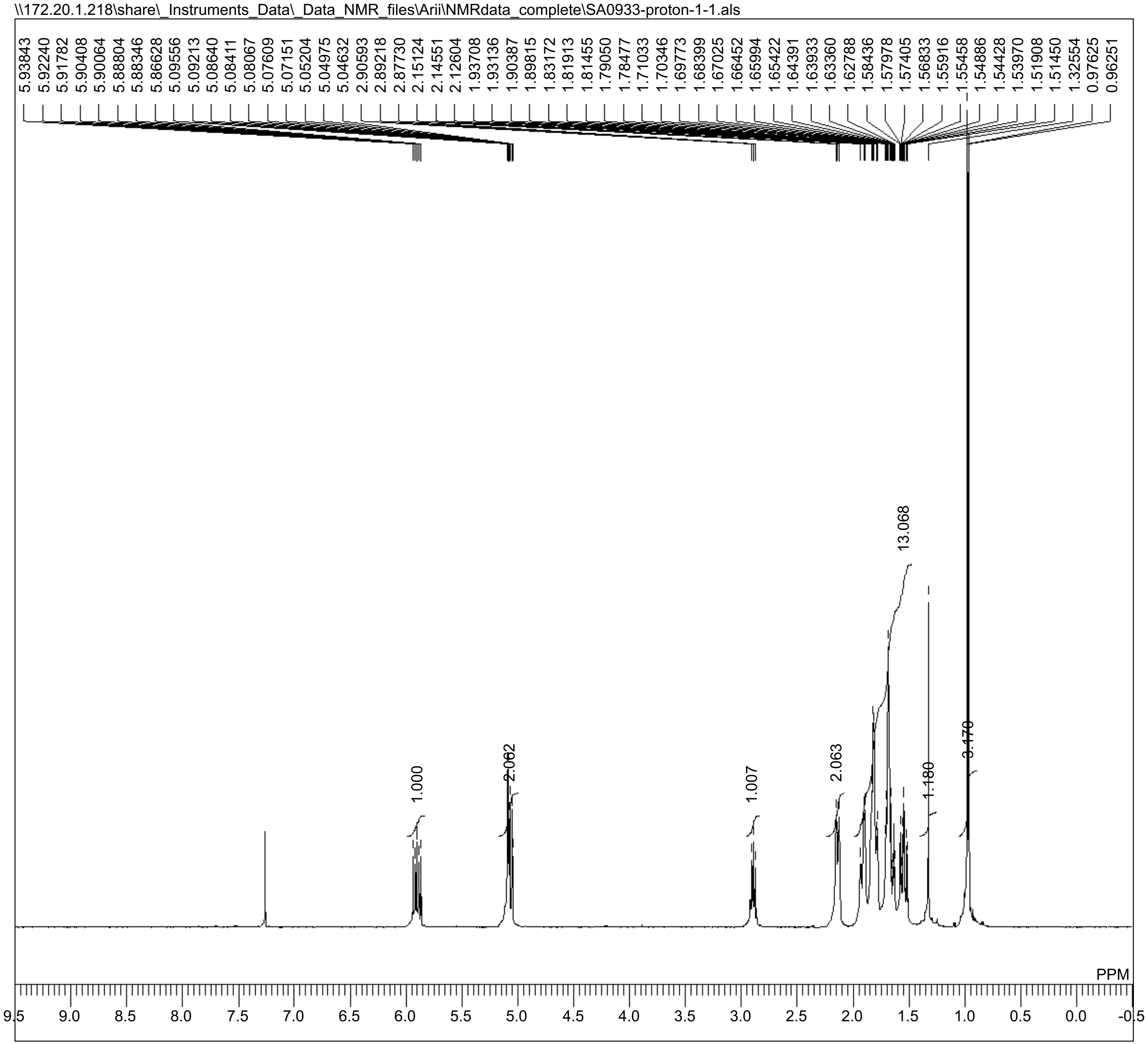

DFILE	SA0933-proton-1-1.als
COMNT	
DATIM	2024-12-06 13:29:38
OBNUC	1H
EXMOD	proton.jxp
OBFRQ	500.16 MHz
OBSET	2.41 KHz
OBFIN	6.01 Hz
POINT	13107
FREQU	7507.51 Hz
SCANS	8
ACQTM	1.7459 sec
PD	5.0000 sec
PW1	5.55 usec
IRNUC	1H
CTEMP	21.8 c
SLVNT	CDCL3
EXREF	7.26 ppm
BF	0.12 Hz
RGAIN	30

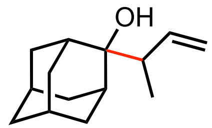

8ac

\\172.20.1.218\share_Instruments_Data_Data_NMR_files\Arii\NMRdata_complete\SA0933-carbon-1-1.als

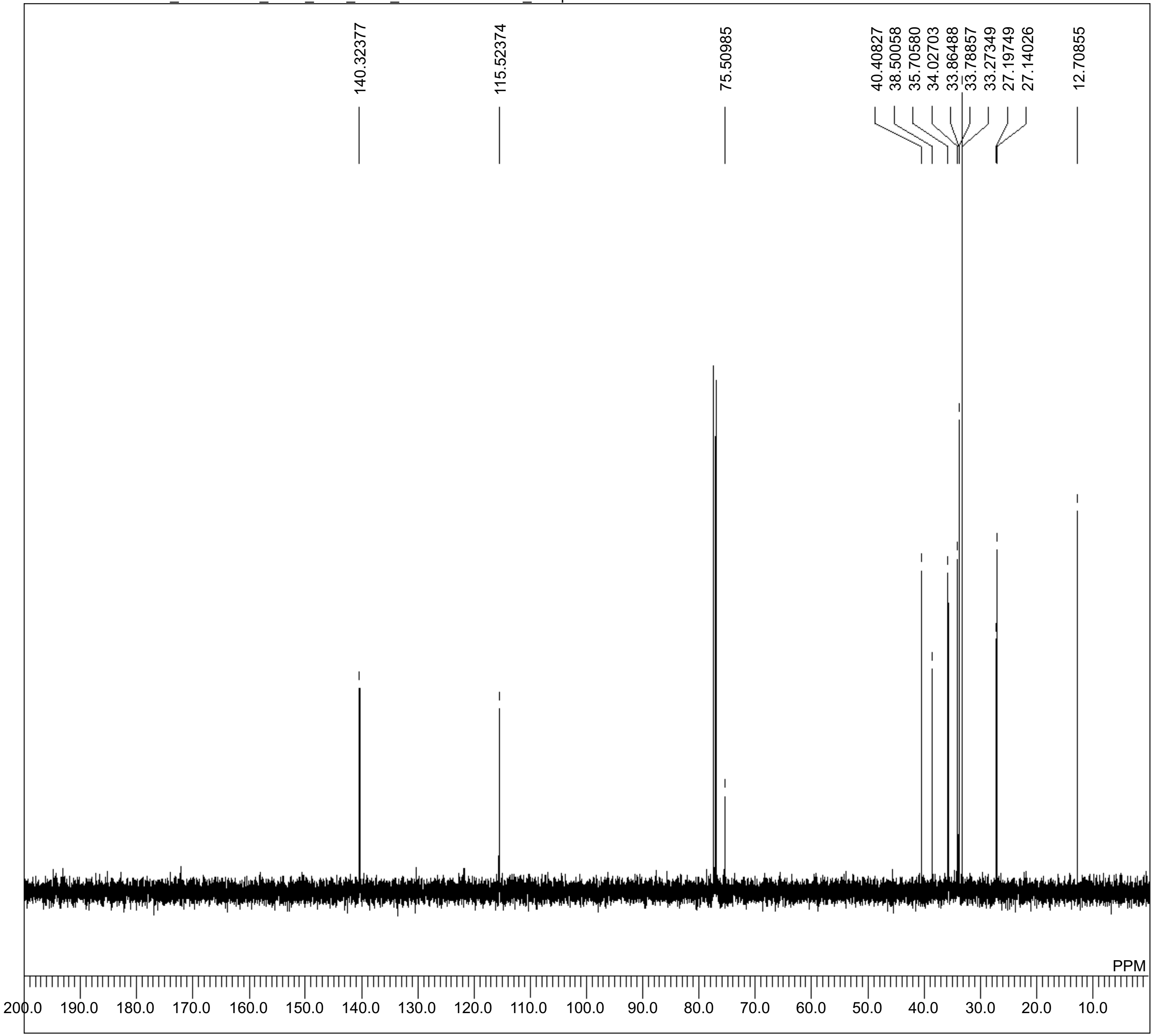

DFILE	SA0933-carbon-1-1.als
COMNT	
DATIM	2024-12-06 13:31:24
OBNUC	13C
EXMOD	carbon.jxp
OBFRQ	125.77 MHz
OBSET	7.87 KHz
OBFIN	4.21 Hz
POINT	26214
FREQU	31446.54 Hz
SCANS	155
ACQTM	0.8336 sec
PD	1.0000 sec
PW1	3.40 usec
IRNUC	1H
CTEMP	22.1 c
SLVNT	CDCL3
EXREF	77.16 ppm
BF	0.12 Hz
RGAIN	60

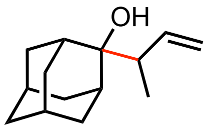

8ac

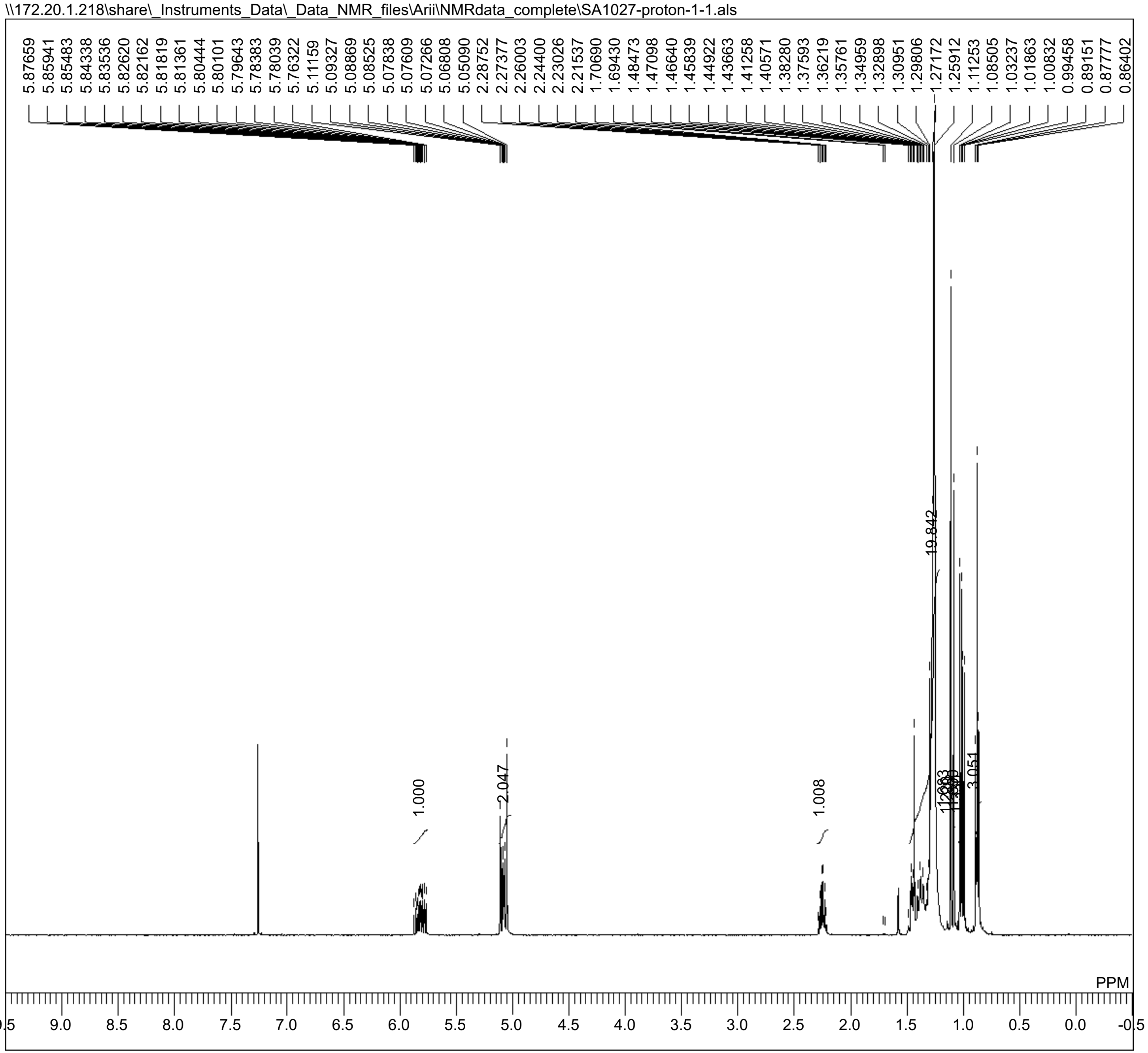

DFILE	SA1027-proton-1-1.als
COMNT	
DATIM	2025-01-14 16:09:55
OBNUC	1H
EXMOD	proton.jxp
OBFRQ	500.16 MHz
OBSET	2.41 KHz
OBFIN	6.01 Hz
POINT	13107
FREQU	7507.51 Hz
SCANS	8
ACQTM	1.7459 sec
PD	5.0000 sec
PW1	5.55 usec
IRNUC	1H
CTEMP	21.6 c
SLVNT	CDCL3
EXREF	7.26 ppm
BF	0.12 Hz
RGAIN	30

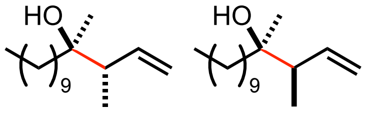

major *minor*

8ad

\\172.20.1.218\share_Instruments_Data_Data_NMR_files\Arii\NMRdata_complete\SA1027-carbon-1-1.als

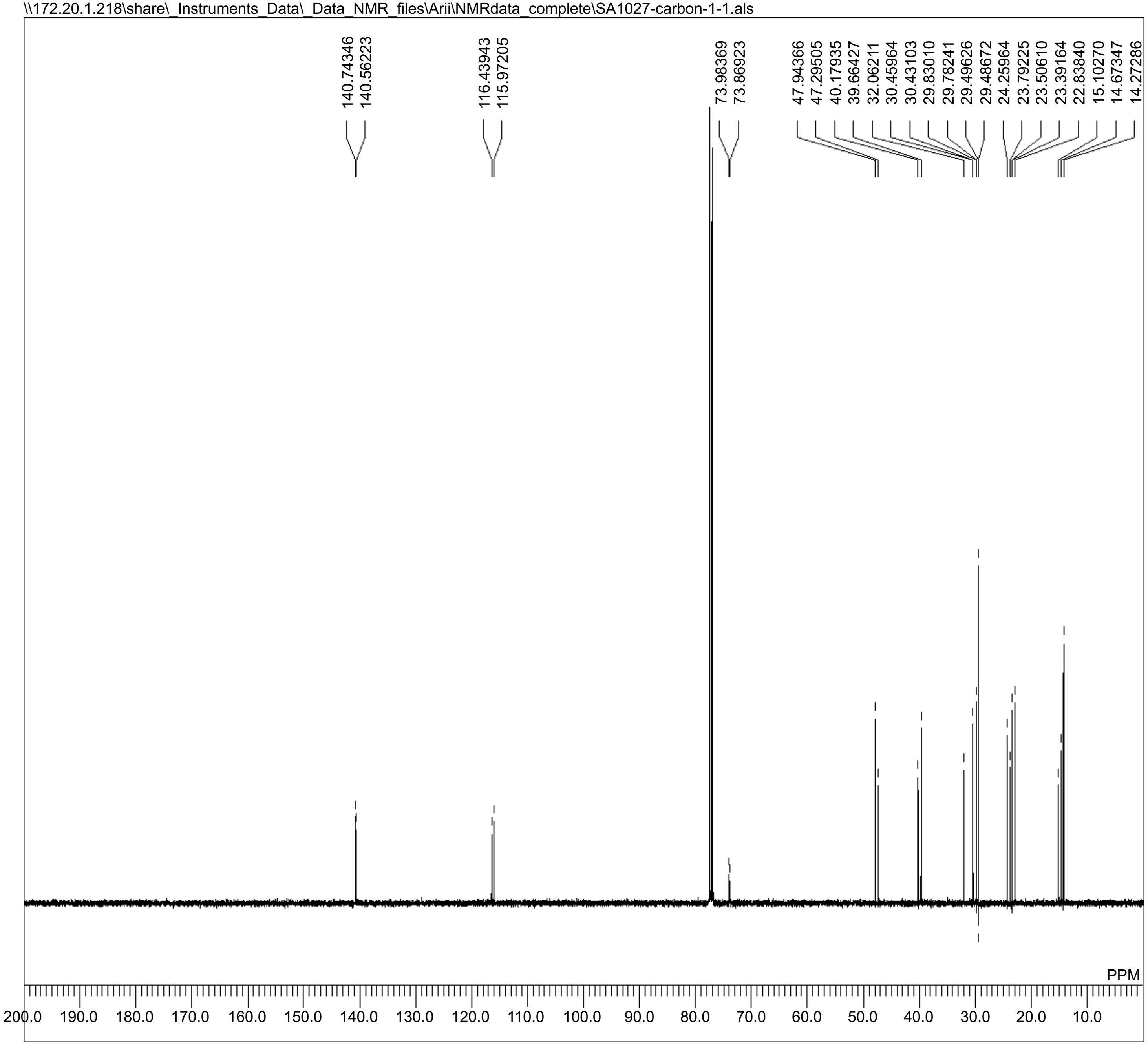

DFILE	SA1027-carbon-1-1.als
COMNT	
DATIM	2025-01-14 16:11:27
OBNUC	13C
EXMOD	carbon.jxp
OBFRQ	125.77 MHz
OBSET	7.87 KHz
OBFIN	4.21 Hz
POINT	26214
FREQU	31446.54 Hz
SCANS	4580
ACQTM	0.8336 sec
PD	1.0000 sec
PW1	3.40 usec
IRNUC	1H
CTEMP	21.8 c
SLVNT	CDCL3
EXREF	77.16 ppm
BF	0.12 Hz
RGAIN	60

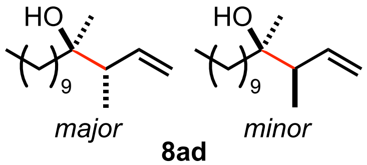

8ad

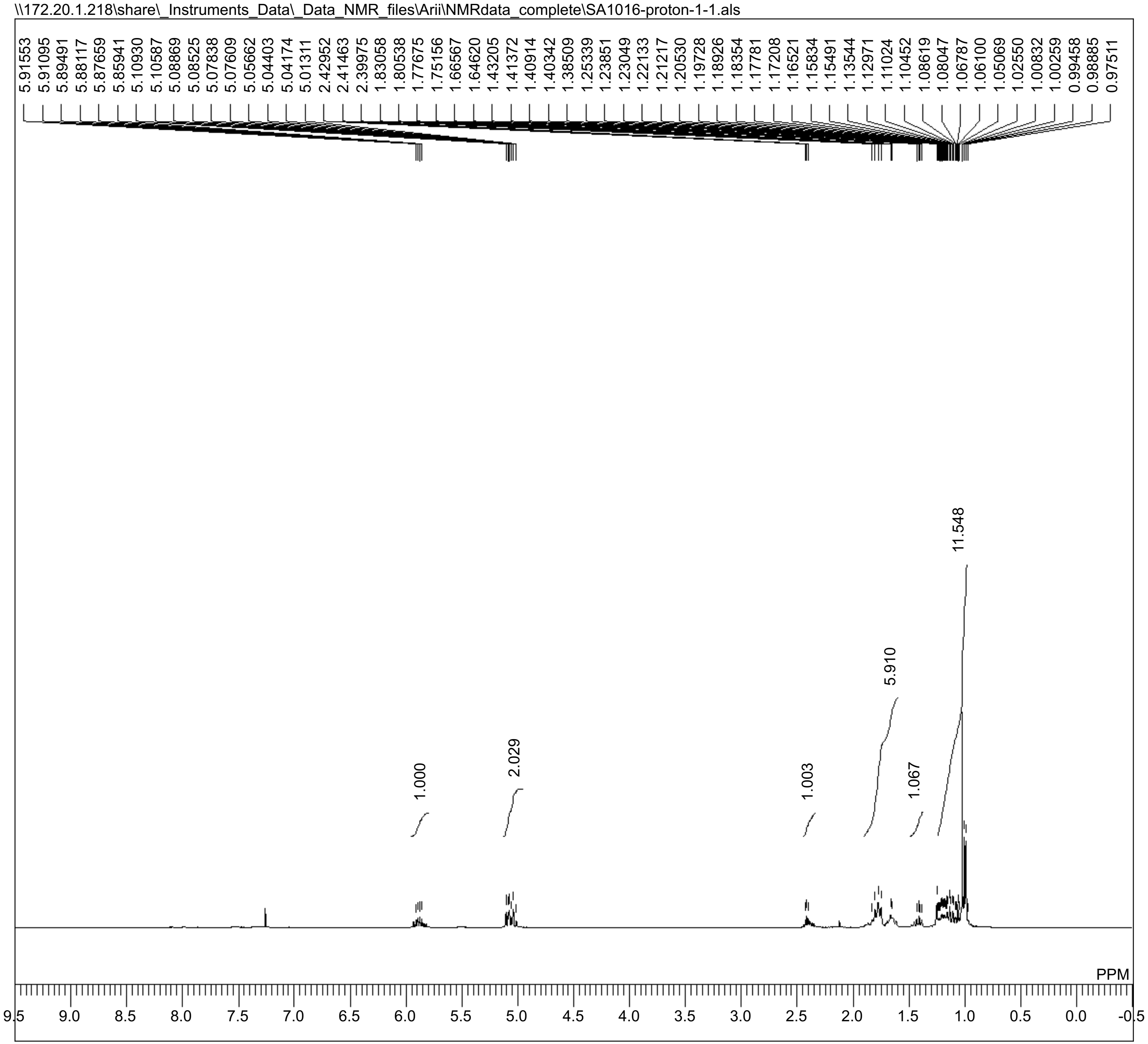

DFILE	SA1016-proton-1-1.als
COMNT	
DATIM	2024-12-19 13:38:47
OBNUC	1H
EXMOD	proton.jxp
OBFRQ	500.16 MHz
OBSET	2.41 KHz
OBFIN	6.01 Hz
POINT	13107
FREQU	7507.51 Hz
SCANS	8
ACQTM	1.7459 sec
PD	5.0000 sec
PW1	5.55 usec
IRNUC	1H
CTEMP	21.8 c
SLVNT	CDCL3
EXREF	7.26 ppm
BF	0.42 Hz
RGAIN	30

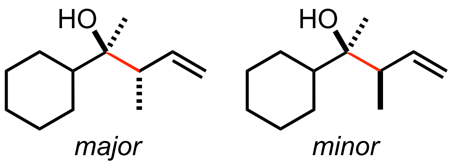

8ae

\\172.20.1.218\share_Instruments_Data_Data_NMR_files\Arii\NMRdata_complete\SA1016-carbon-1-1.als

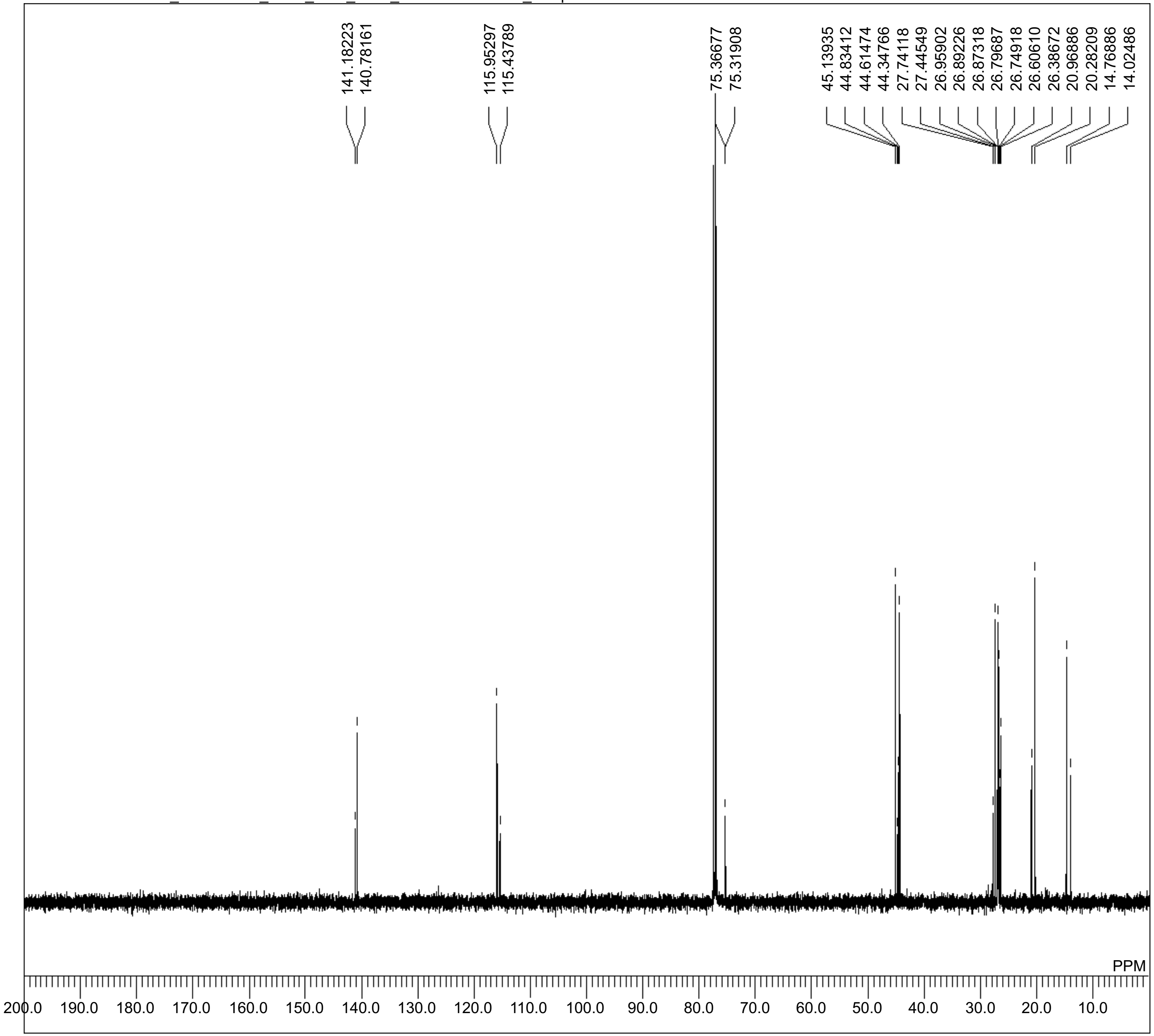

DFILE	SA1016-carbon-1-1.als
COMNT	
DATIM	2024-12-19 13:40:19
OBNUC	13C
EXMOD	carbon.jxp
OBFRQ	125.77 MHz
OBSET	7.87 KHz
OBFIN	4.21 Hz
POINT	26214
FREQU	31446.54 Hz
SCANS	424
ACQTM	0.8336 sec
PD	1.0000 sec
PW1	3.40 usec
IRNUC	1H
CTEMP	22.0 c
SLVNT	CDCL3
EXREF	77.16 ppm
BF	0.42 Hz
RGAIN	60

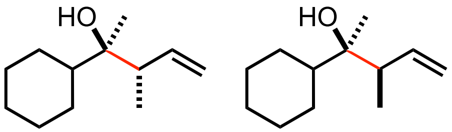

major *minor*

8ae

\\172.20.1.218\share_Instruments_Data_Data_NMR_files\Arii\NMRdata_complete\SA1053-proton-1-1.als

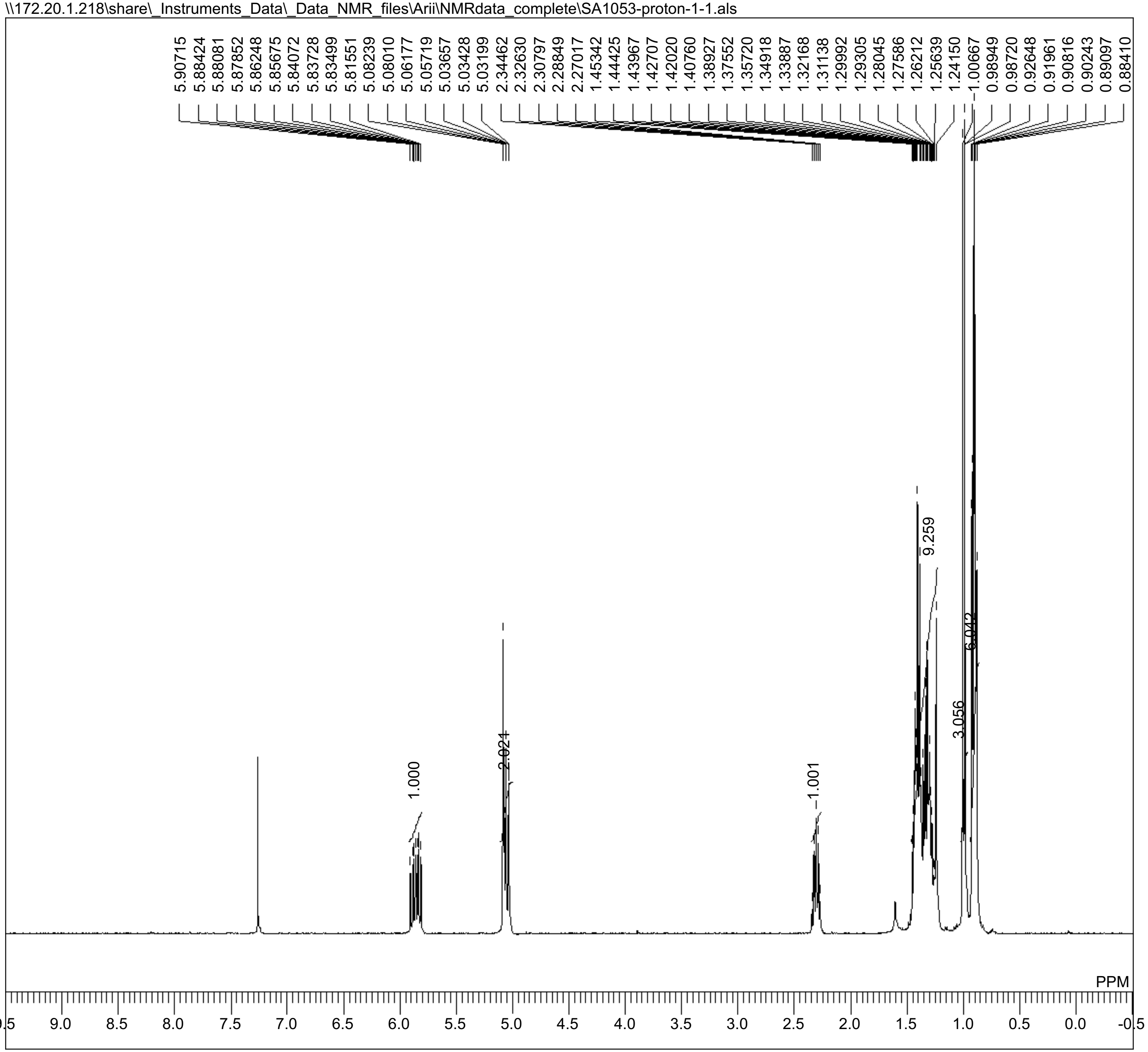

DFILE	SA1053-proton-1-1.als
COMNT	
DATIM	2025-01-23 03:25:35
OBNUC	1H
EXMOD	proton.jxp
OBFRQ	391.78 MHz
OBSET	8.51 KHz
OBFIN	3.34 Hz
POINT	13107
FREQU	5882.35 Hz
SCANS	8
ACQTM	2.2282 sec
PD	4.0000 sec
PW1	6.30 usec
IRNUC	1H
CTEMP	20.6 c
SLVNT	CDCL3
EXREF	7.26 ppm
BF	0.12 Hz
RGAIN	38

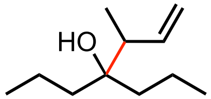

8af

\\172.20.1.218\share_Instruments_Data_Data_NMR_files\Arii\NMRdata_complete\SA1053-carbon-1-1.als

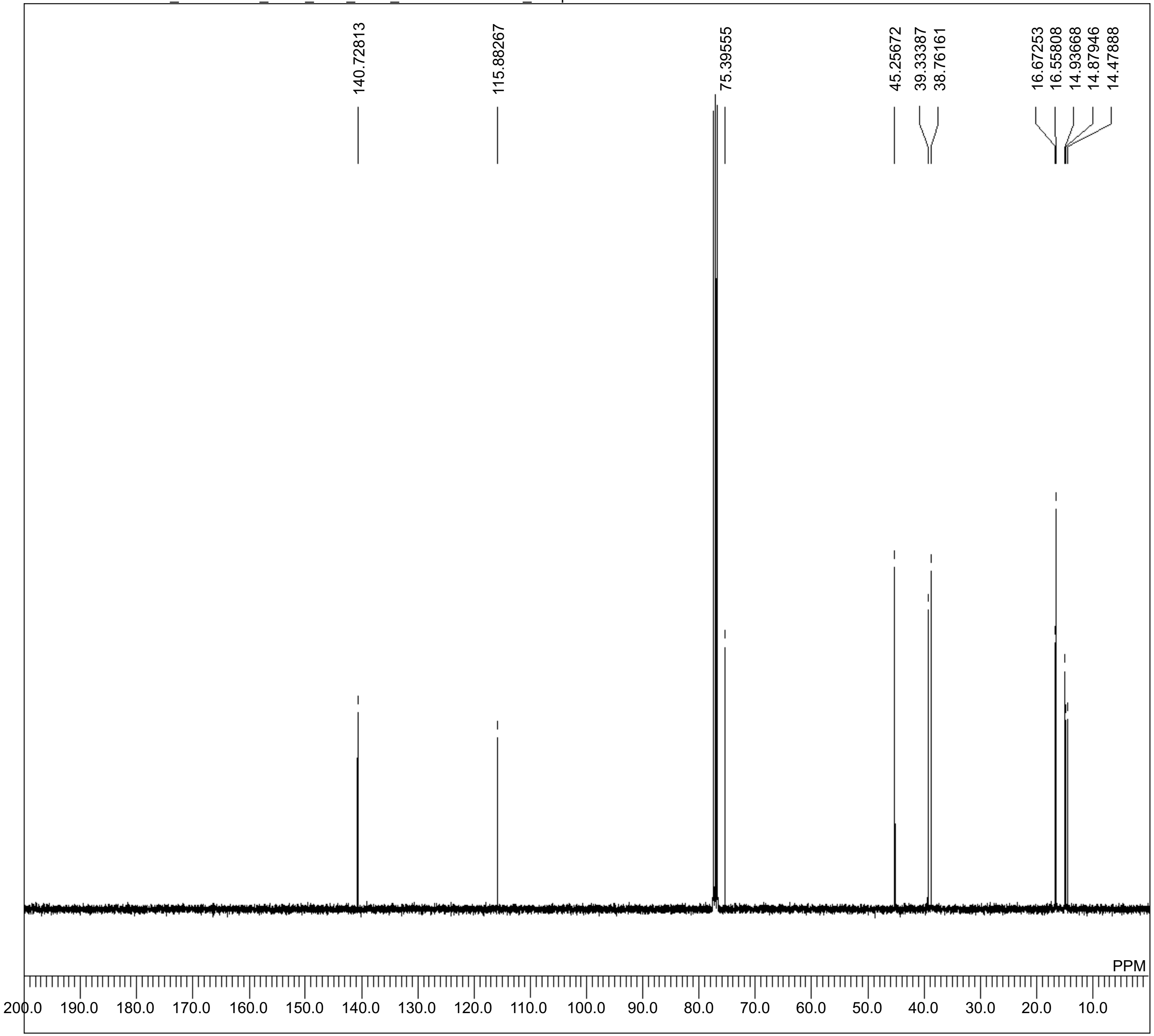

DFILE	SA1053-carbon-1-1.als
COMNT	
DATIM	2025-01-23 03:27:02
OBNUC	13C
EXMOD	carbon.jxp
OBFRQ	98.52 MHz
OBSET	4.64 KHz
OBFIN	8.74 Hz
POINT	26214
FREQU	24630.54 Hz
SCANS	2155
ACQTM	1.0643 sec
PD	2.0000 sec
PW1	2.93 usec
IRNUC	1H
CTEMP	20.3 c
SLVNT	CDCL3
EXREF	77.16 ppm
BF	0.12 Hz
RGAIN	60

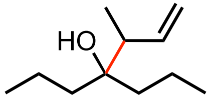

8af

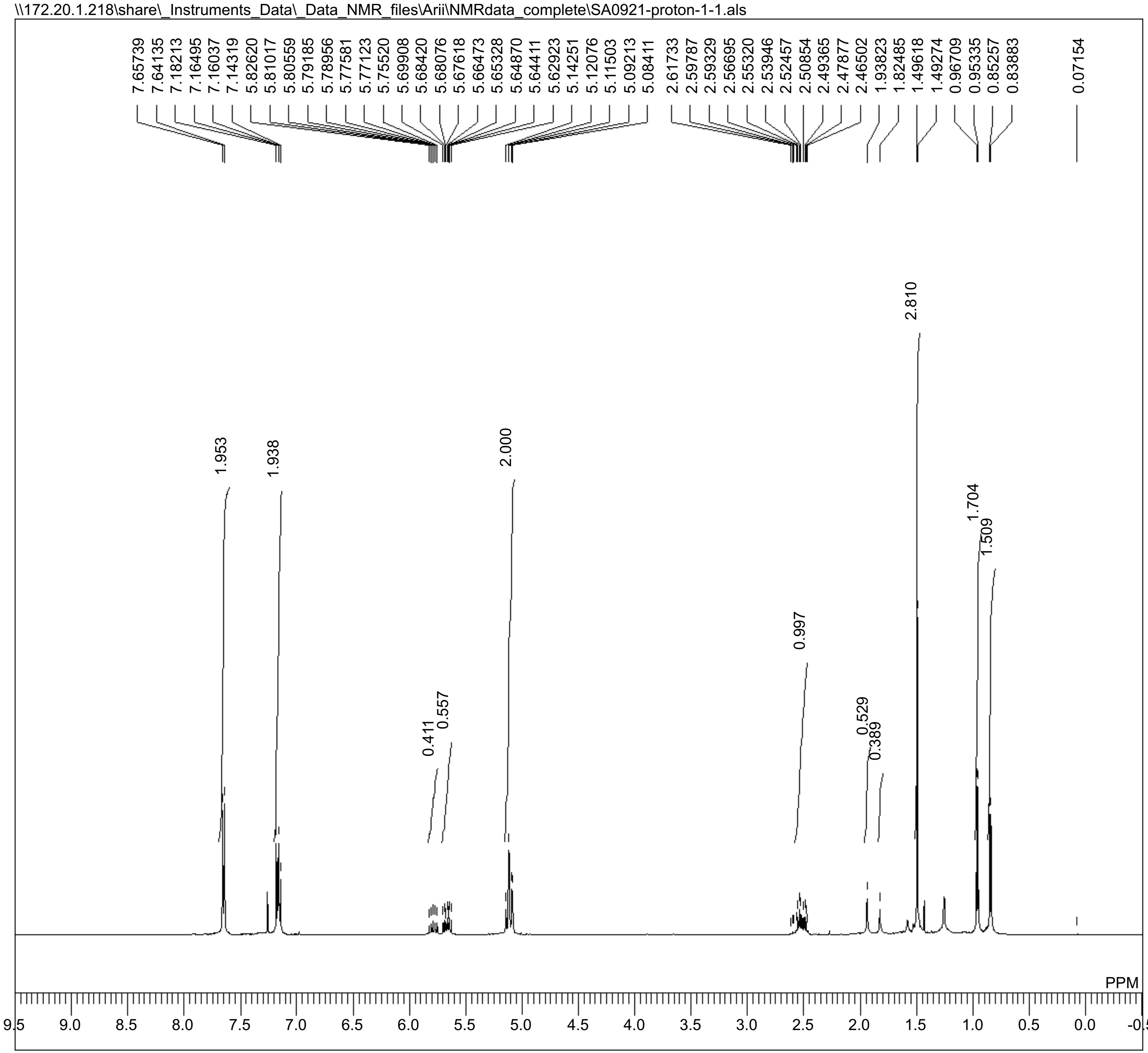

DFILE	SA0921-proton-1-1.als
COMNT	
DATIM	2024-12-16 18:14:59
OBNUC	1H
EXMOD	proton.jxp
OBFRQ	500.16 MHz
OBSET	2.41 KHz
OBFIN	6.01 Hz
POINT	13107
FREQU	7507.51 Hz
SCANS	8
ACQTM	1.7459 sec
PD	5.0000 sec
PW1	5.55 usec
IRNUC	1H
CTEMP	21.8 c
SLVNT	CDCL3
EXREF	7.26 ppm
BF	0.42 Hz
RGAIN	30

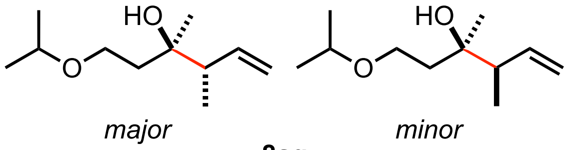

8ag

\\172.20.1.218\share_Instruments_Data_Data_NMR_files\Arii\NMRdata_complete\SA0921-carbon-1-1.als

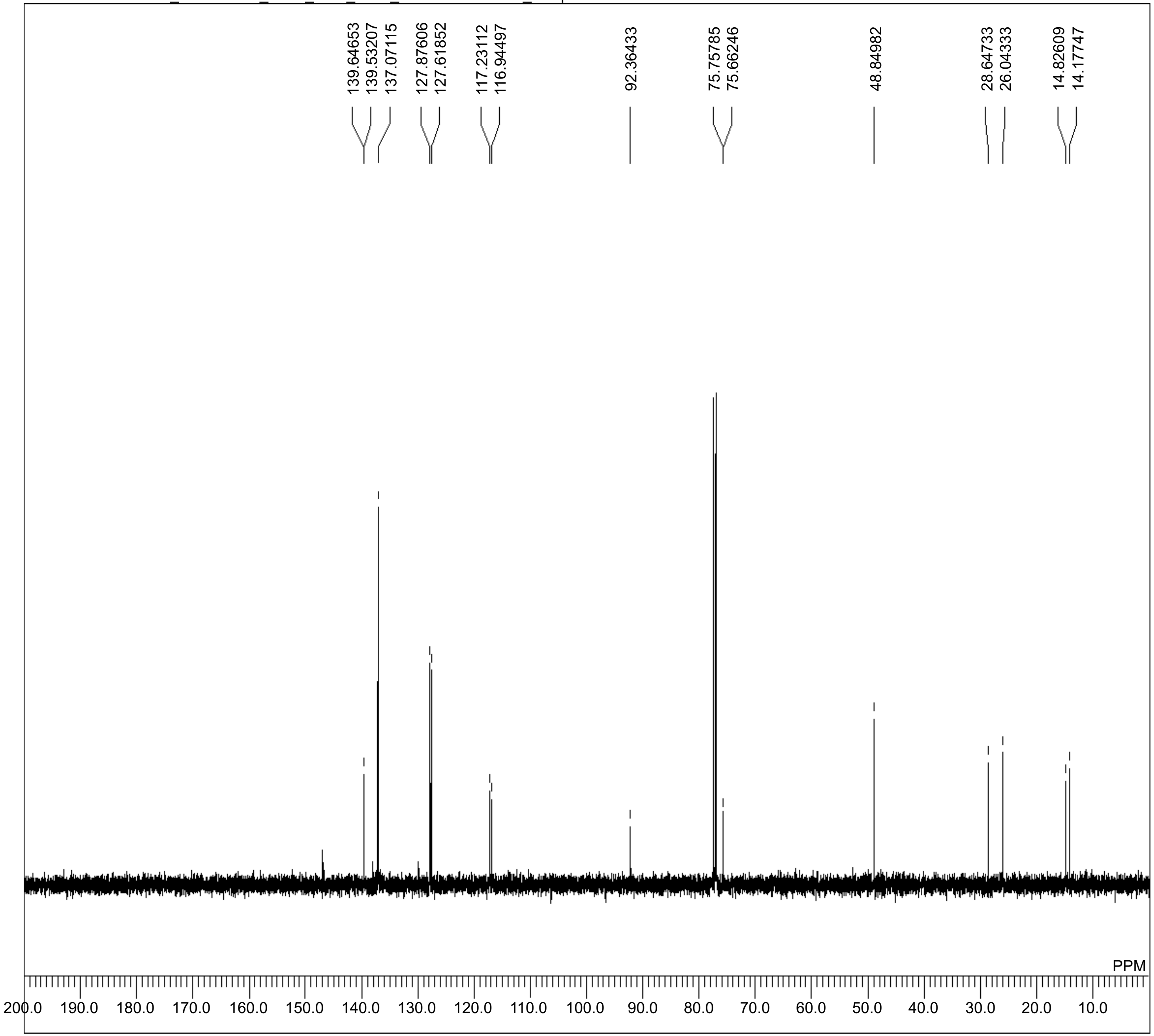

DFILE	SA0921-carbon-1-1.als
COMNT	
DATIM	2024-12-11 19:17:02
OBNUC	13C
EXMOD	carbon.jxp
OBFRQ	125.77 MHz
OBSET	7.87 KHz
OBFIN	4.21 Hz
POINT	26214
FREQU	31446.54 Hz
SCANS	115
ACQTM	0.8336 sec
PD	1.0000 sec
PW1	3.40 usec
IRNUC	1H
CTEMP	21.9 c
SLVNT	CDCL3
EXREF	77.16 ppm
BF	0.42 Hz
RGAIN	60

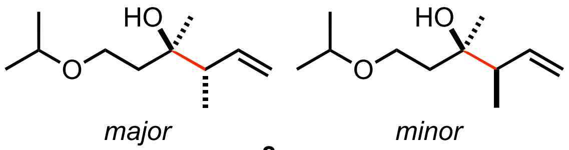

8ag

\\172.20.1.218\share_Instruments_Data_Data_NMR_files\Arii\NMRdata_complete\SA1025-proton-1-1.als

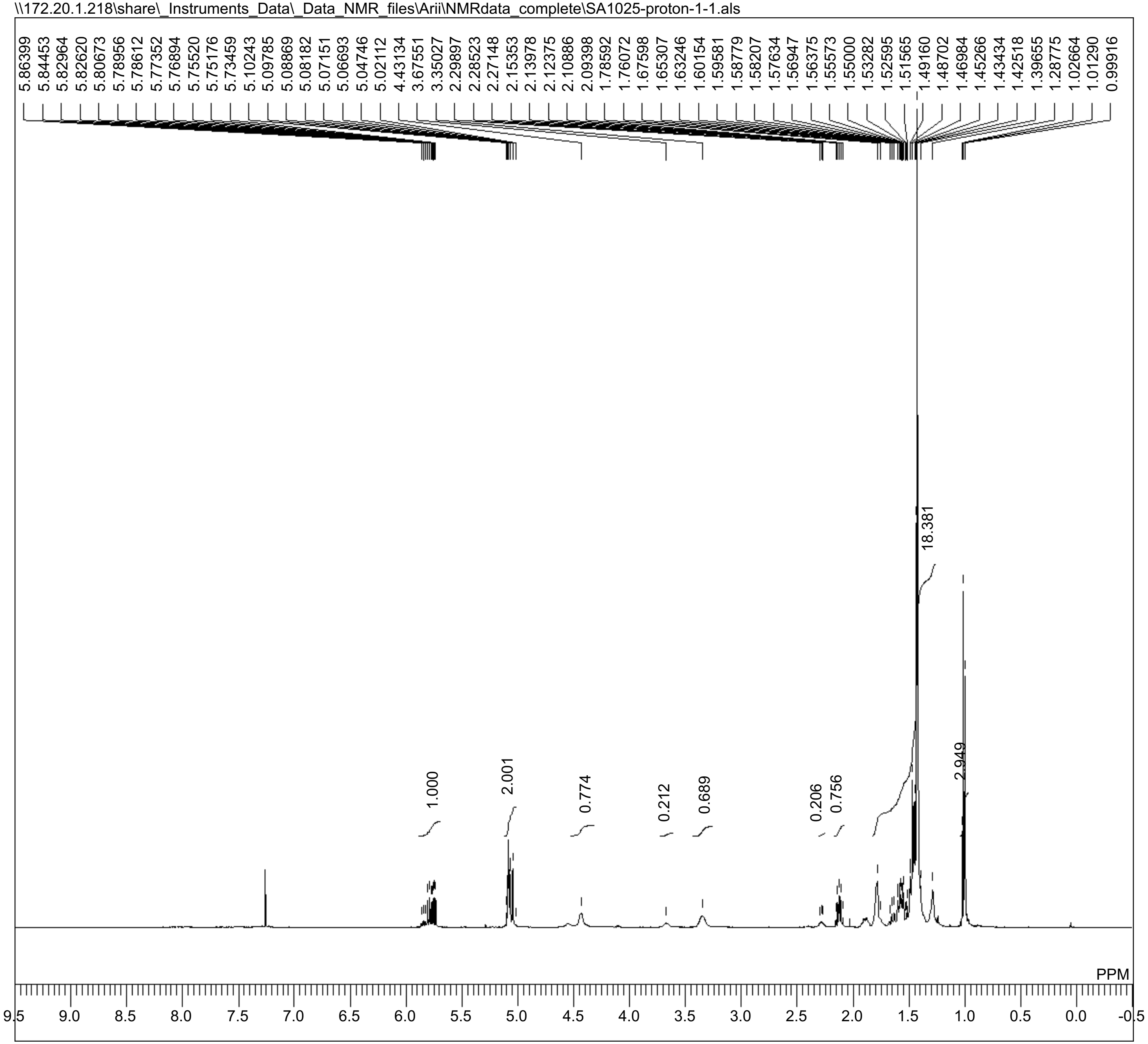

DFILE	SA1025-proton-1-1.als
COMNT	
DATIM	2025-01-14 18:34:39
OBNUC	1H
EXMOD	proton.jxp
OBFRQ	500.16 MHz
OBSET	2.41 KHz
OBFIN	6.01 Hz
POINT	13107
FREQU	7507.51 Hz
SCANS	8
ACQTM	1.7459 sec
PD	5.0000 sec
PW1	5.55 usec
IRNUC	1H
CTEMP	21.8 c
SLVNT	CDCL3
EXREF	7.26 ppm
BF	0.12 Hz
RGAIN	28

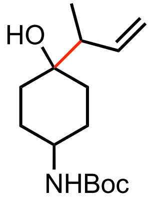

8ah

\\172.20.1.218\share_Instruments_Data_Data_NMR_files\Arii\NMRdata_complete\SA1025-carbon-1-1.als

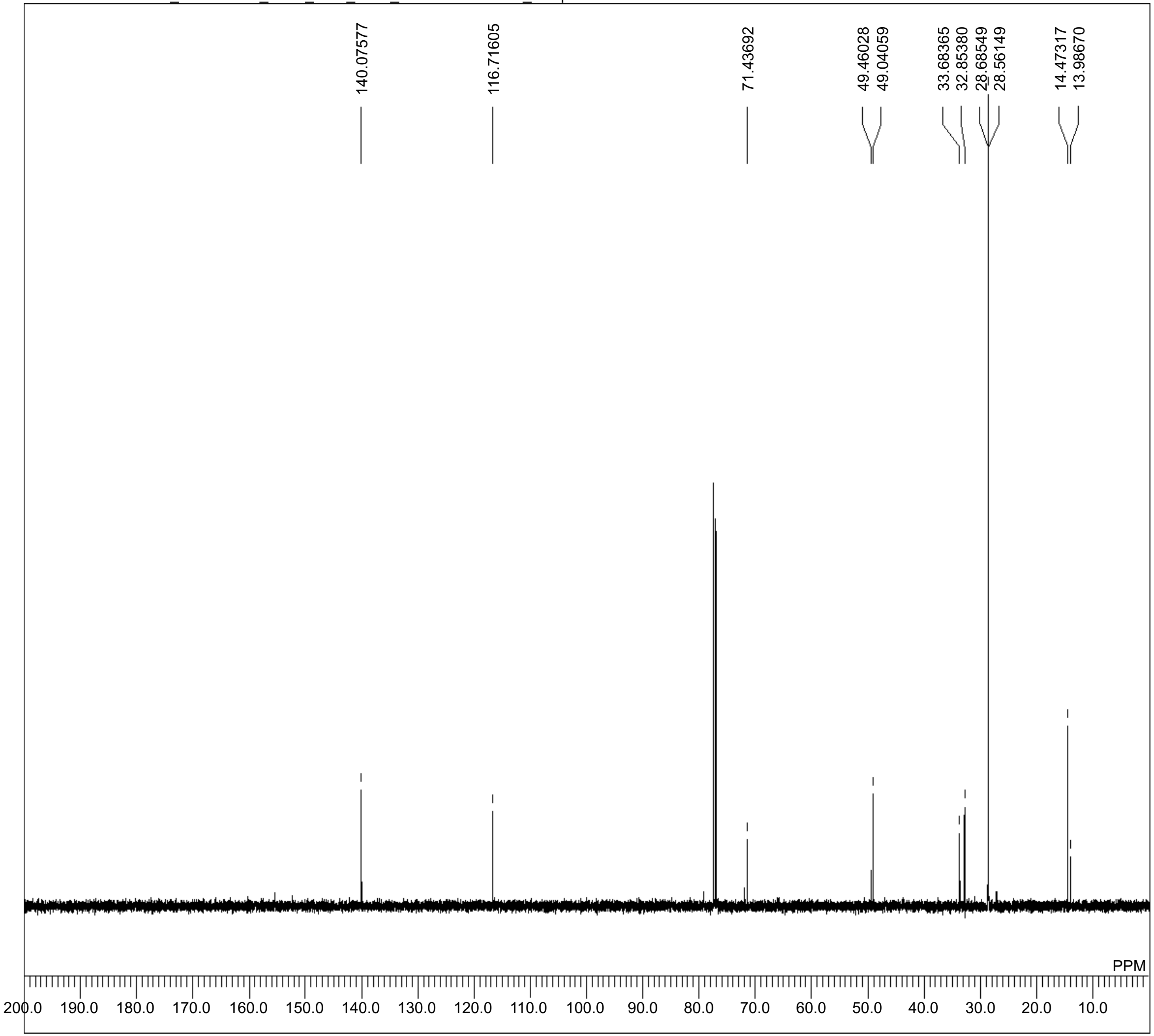

DFILE	SA1025-carbon-1-1.als
COMNT	
DATIM	2025-01-14 18:36:12
OBNUC	13C
EXMOD	carbon.jxp
OBFRQ	125.77 MHz
OBSET	7.87 KHz
OBFIN	4.21 Hz
POINT	26214
FREQU	31446.54 Hz
SCANS	340
ACQTM	0.8336 sec
PD	1.0000 sec
PW1	3.40 usec
IRNUC	1H
CTEMP	21.8 c
SLVNT	CDCL3
EXREF	77.16 ppm
BF	0.12 Hz
RGAIN	60

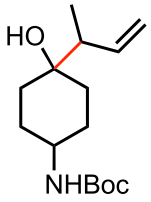

8ah

\\172.20.1.218\share_Instruments_Data_Data_NMR_files\Arii\NMRdata_complete\SA1015-proton-1-1.als

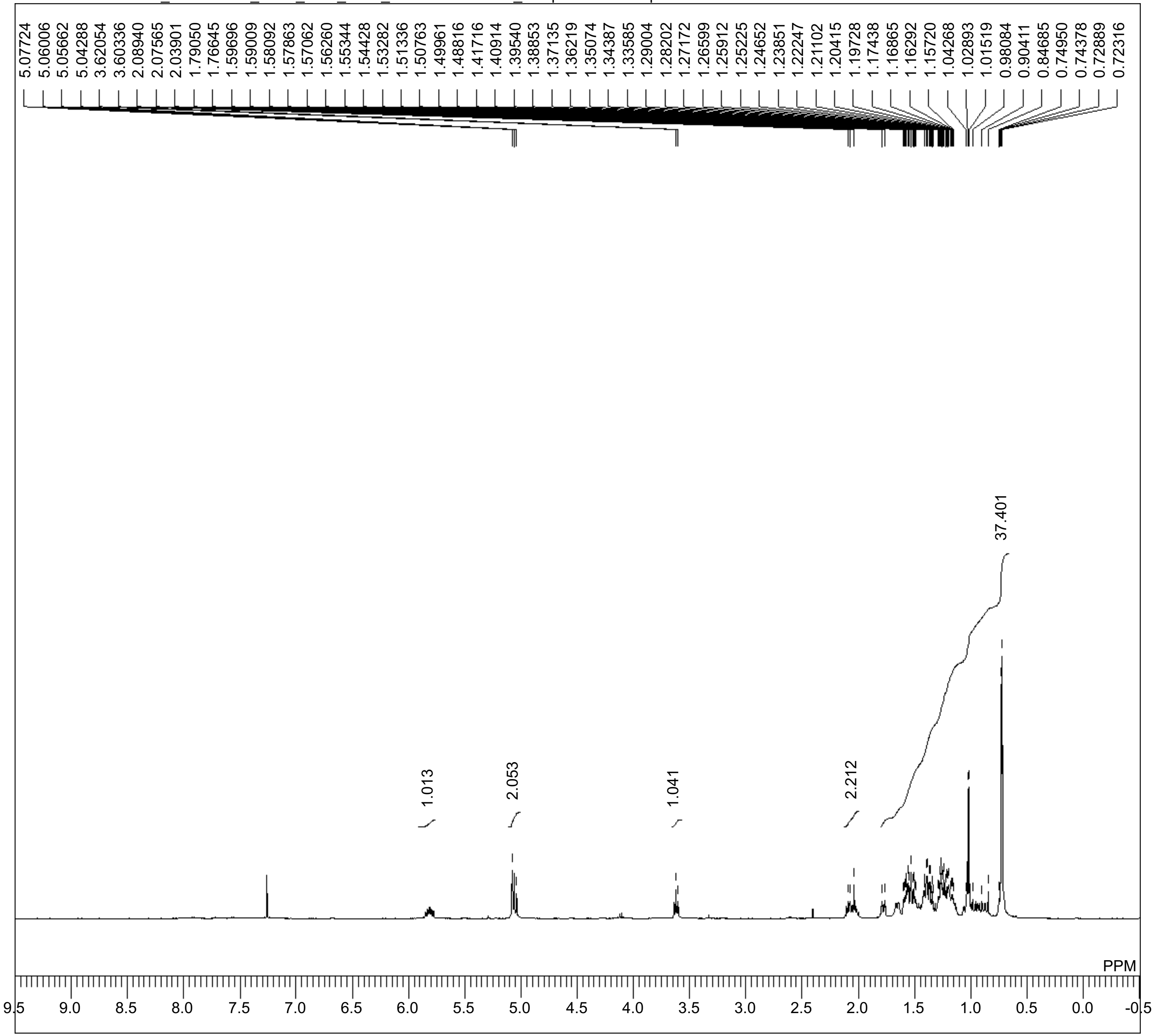

DFILE	SA1015-proton-1-1.als
COMNT	
DATIM	2024-12-19 13:17:50
OBNUC	1H
EXMOD	proton.jxp
OBFRQ	500.16 MHz
OBSET	2.41 KHz
OBFIN	6.01 Hz
POINT	13107
FREQU	7507.51 Hz
SCANS	8
ACQTM	1.7459 sec
PD	5.0000 sec
PW1	5.55 usec
IRNUC	1H
CTEMP	21.8 c
SLVNT	CDCL3
EXREF	7.26 ppm
BF	0.42 Hz
RGAIN	28

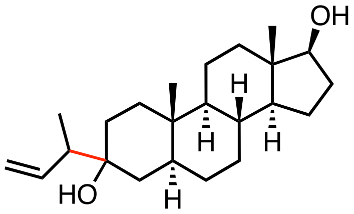

8ai

\\172.20.1.218\share_Instruments_Data_Data_NMR_files\Arii\NMRdata_complete\SA1015-carbon-1-1.als

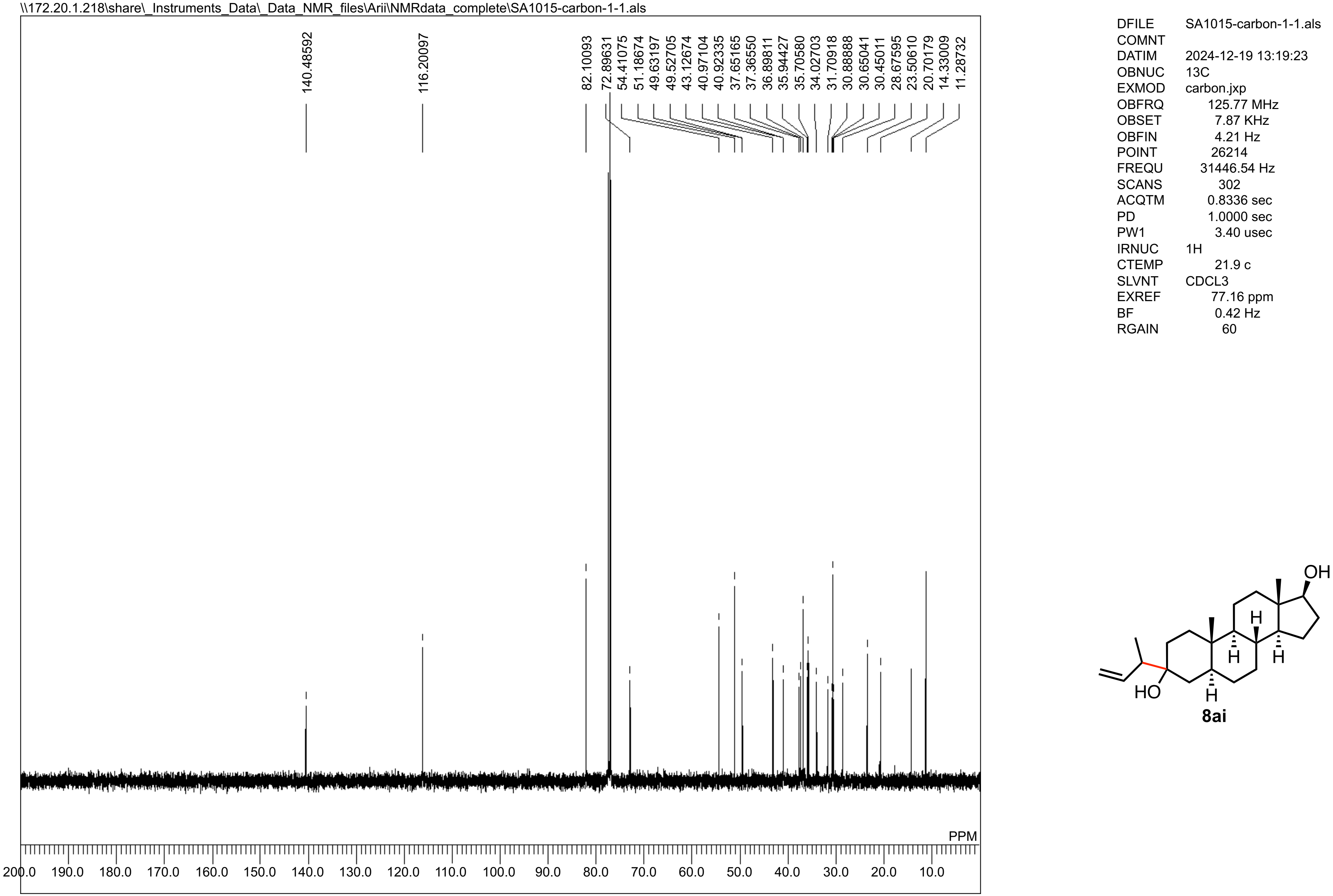

8ai

DFILE	SA1015-carbon-1-1.als
COMNT	
DATIM	2024-12-19 13:19:23
OBNUC	13C
EXMOD	carbon.jxp
OBFRQ	125.77 MHz
OBSET	7.87 KHz
OBFIN	4.21 Hz
POINT	26214
FREQU	31446.54 Hz
SCANS	302
ACQTM	0.8336 sec
PD	1.0000 sec
PW1	3.40 usec
IRNUC	1H
CTEMP	21.9 c
SLVNT	CDCL3
EXREF	77.16 ppm
BF	0.42 Hz
RGAIN	60

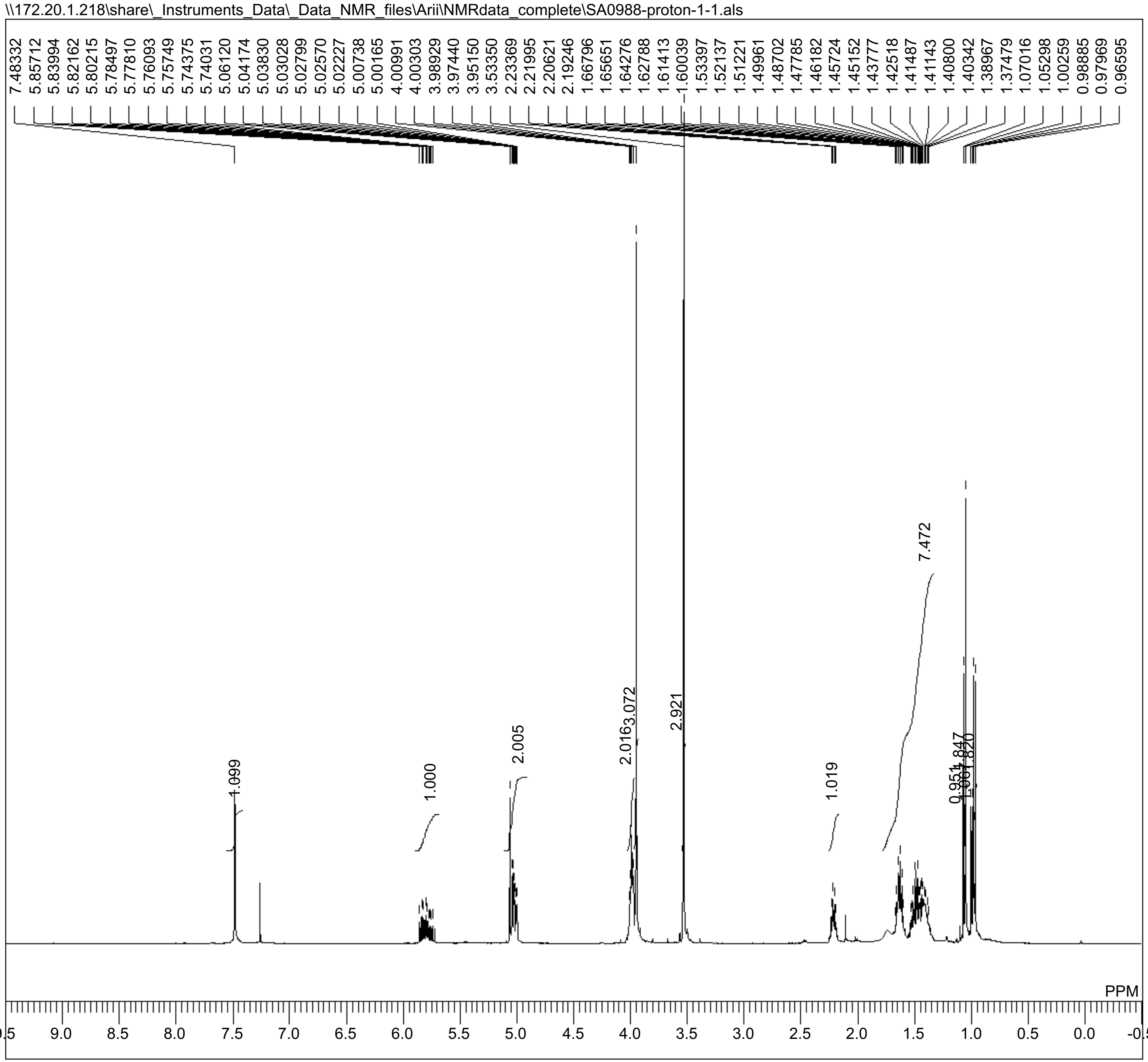

DFILE	SA0988-proton-1-1.als
COMNT	
DATIM	2024-12-06 14:23:36
OBNUC	1H
EXMOD	proton.jxp
OBFRQ	500.16 MHz
OBSET	2.41 KHz
OBFIN	6.01 Hz
POINT	13107
FREQU	7507.51 Hz
SCANS	8
ACQTM	1.7459 sec
PD	5.0000 sec
PW1	5.55 usec
IRNUC	1H
CTEMP	21.9 c
SLVNT	CDCL3
EXREF	7.26 ppm
BF	0.12 Hz
RGAIN	26

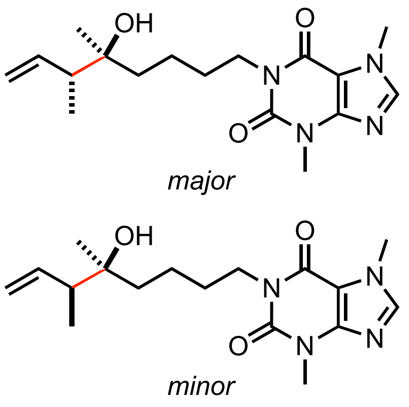

major

minor

8aj

\\172.20.1.218\share_Instruments_Data_Data_NMR_files\Arii\NMRdata_complete\SA0988-carbon-1-1.als

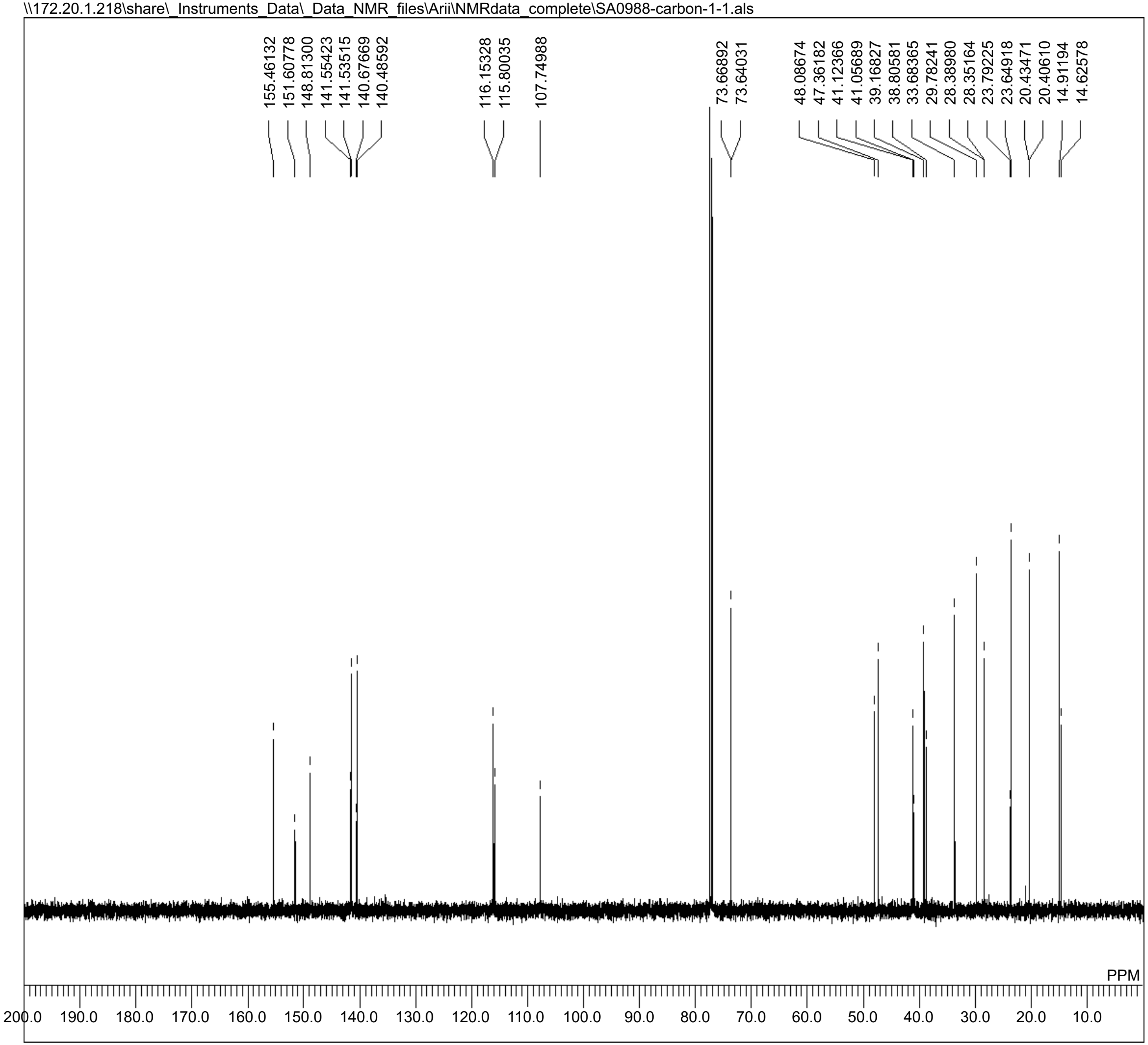

DFILE	SA0988-carbon-1-1.als
COMNT	
DATIM	2024-12-06 14:26:16
OBNUC	13C
EXMOD	carbon.jxp
OBFRQ	125.77 MHz
OBSET	7.87 KHz
OBFIN	4.21 Hz
POINT	26214
FREQU	31446.54 Hz
SCANS	300
ACQTM	0.8336 sec
PD	1.0000 sec
PW1	3.40 usec
IRNUC	1H
CTEMP	22.1 c
SLVNT	CDCL3
EXREF	77.16 ppm
BF	0.12 Hz
RGAIN	60

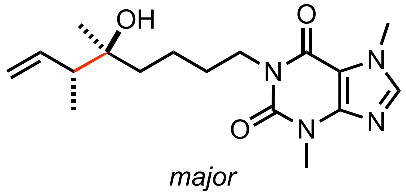

major

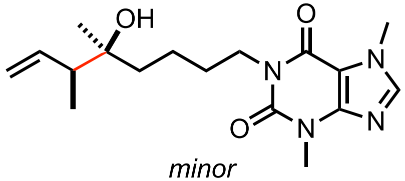

minor

8aj

\\172.20.1.218\share_Instruments_Data_Data_NMR_files\Arii\NMRdata_complete\SA1054-proton-1-1.als

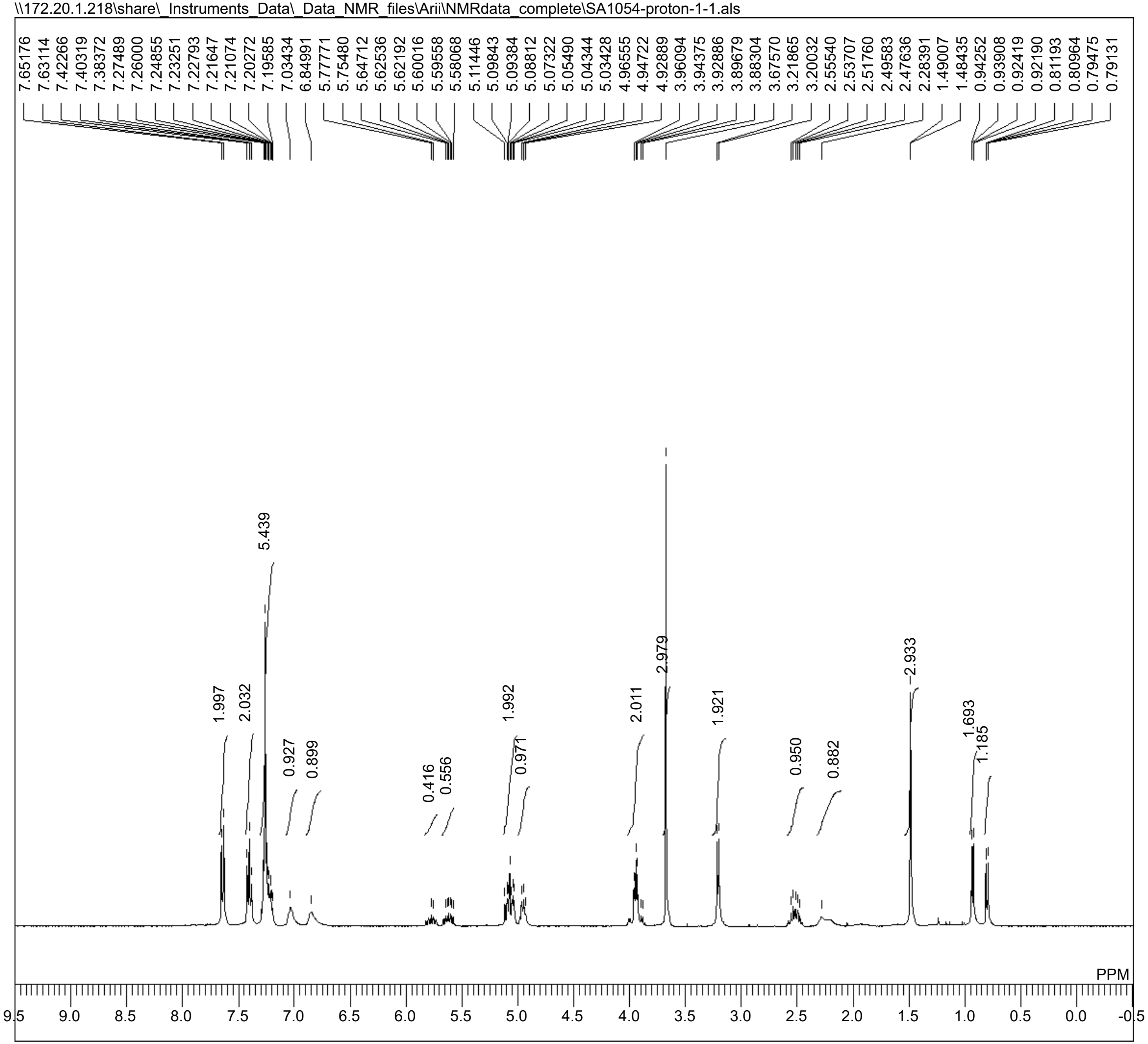

DFILE	SA1054-proton-1-1.als
COMNT	
DATIM	2025-01-23 02:10:51
OBNUC	1H
EXMOD	proton.jxp
OBFRQ	391.78 MHz
OBSET	8.51 KHz
OBFIN	3.34 Hz
POINT	13107
FREQU	5882.35 Hz
SCANS	8
ACQTM	2.2282 sec
PD	4.0000 sec
PW1	6.30 usec
IRNUC	1H
CTEMP	20.5 c
SLVNT	CDCL3
EXREF	7.26 ppm
BF	0.12 Hz
RGAIN	36

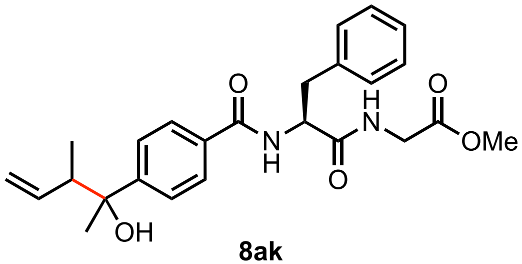

8ak

\\172.20.1.218\share_Instruments_Data_Data_NMR_files\Arii\NMRdata_complete\SA1054-carbon-1-1.als

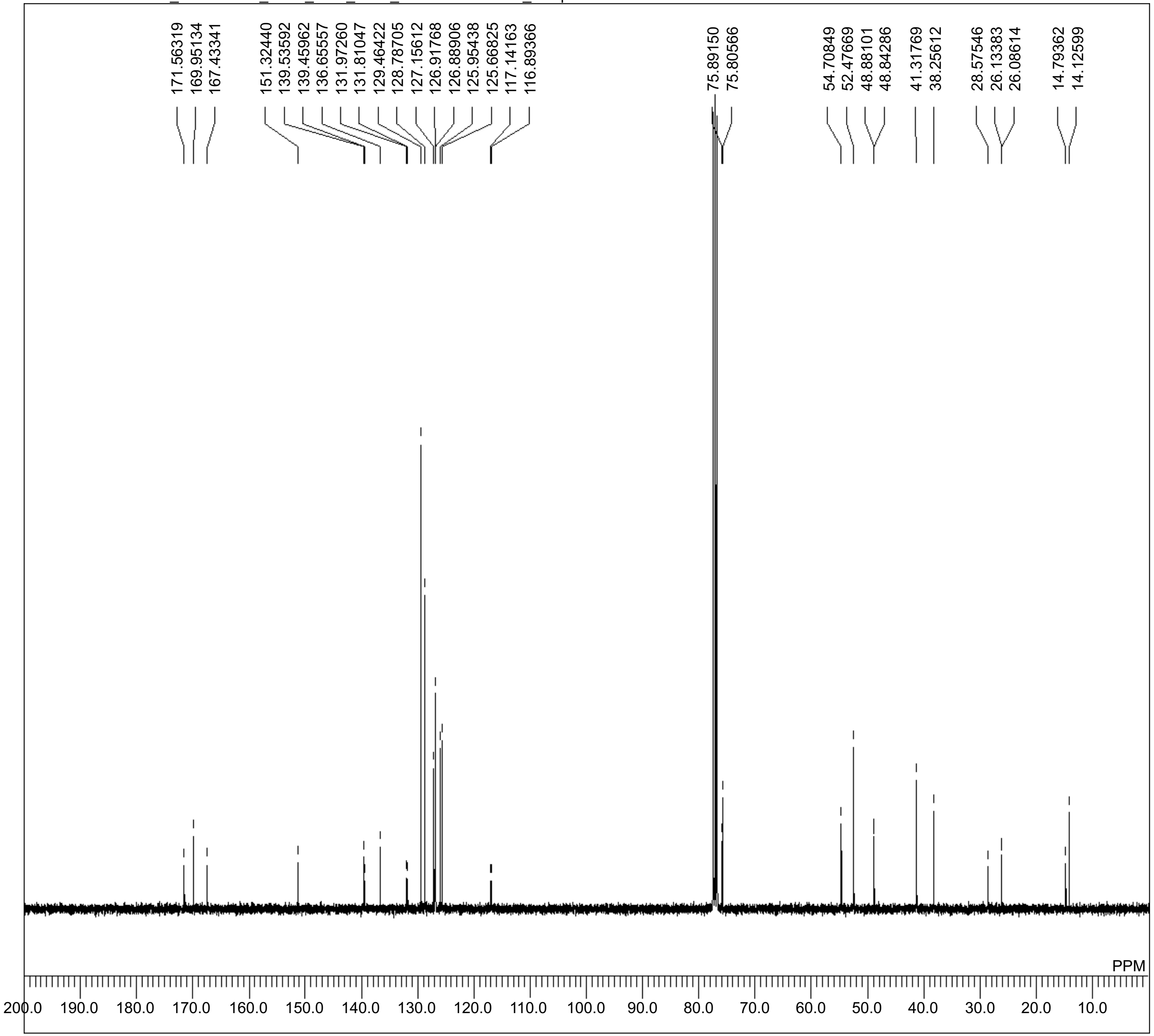

DFILE	SA1054-carbon-1-1.als
COMNT	
DATIM	2025-01-23 02:12:18
OBNUC	13C
EXMOD	carbon.jxp
OBFRQ	98.52 MHz
OBSET	4.64 KHz
OBFIN	8.74 Hz
POINT	26214
FREQU	24630.54 Hz
SCANS	1363
ACQTM	1.0643 sec
PD	2.0000 sec
PW1	2.93 usec
IRNUC	1H
CTEMP	20.7 c
SLVNT	CDCL3
EXREF	77.16 ppm
BF	0.12 Hz
RGAIN	60

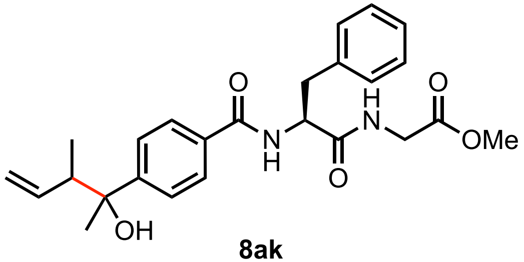

8ak

\\172.20.1.218\share_Instruments_Data_Data_NMR_files\Arii\NMRdata_complete\SA1066-proton-1-1.als

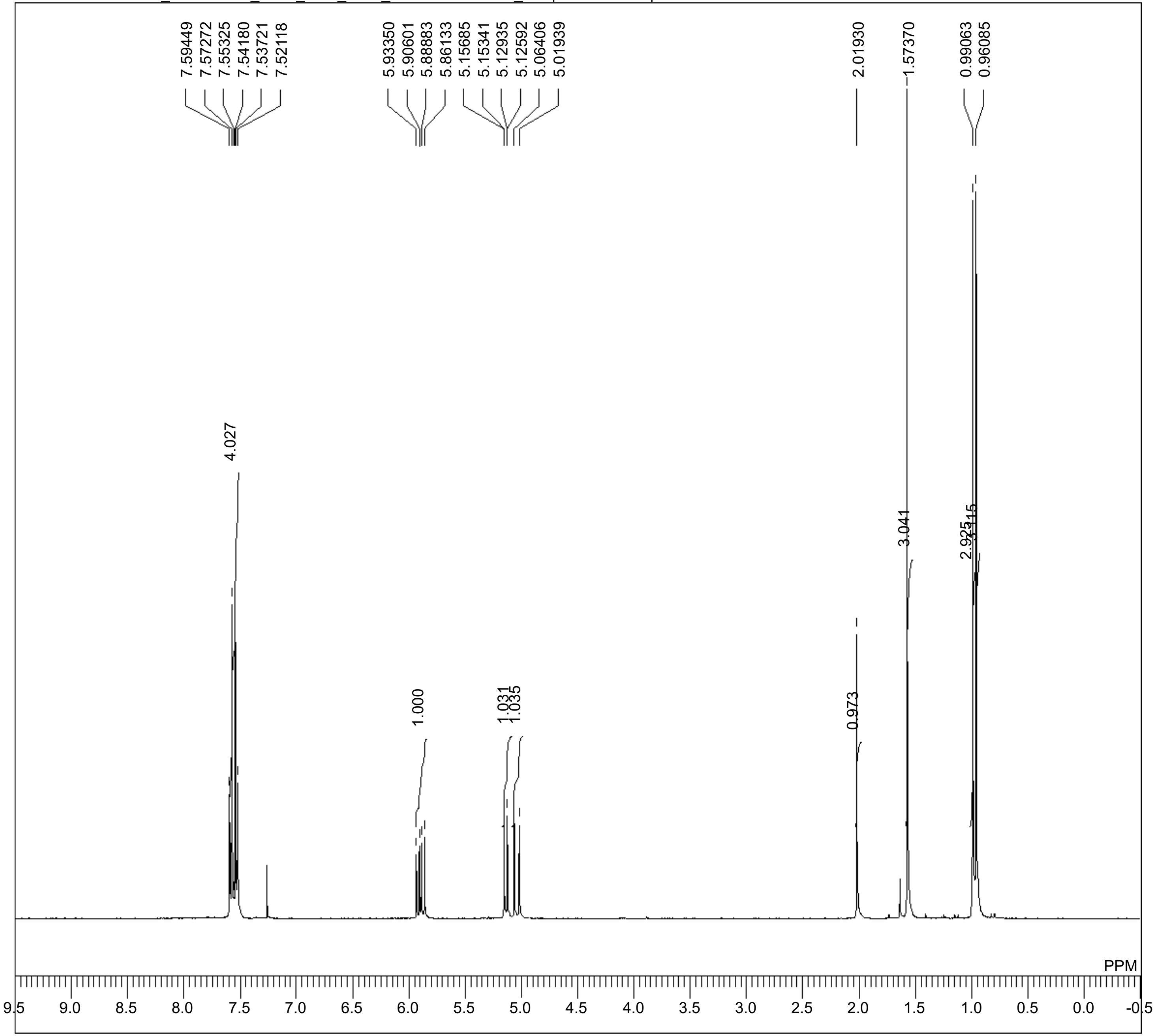

DFILE	SA1066-proton-1-1.als
COMNT	
DATIM	2025-01-24 16:14:30
OBNUC	1H
EXMOD	proton.jxp
OBFRQ	391.78 MHz
OBSET	8.51 KHz
OBFIN	3.34 Hz
POINT	13107
FREQU	5882.35 Hz
SCANS	8
ACQTM	2.2282 sec
PD	4.0000 sec
PW1	6.30 usec
IRNUC	1H
CTEMP	21.1 c
SLVNT	CDCL3
EXREF	7.26 ppm
BF	0.12 Hz
RGAIN	36

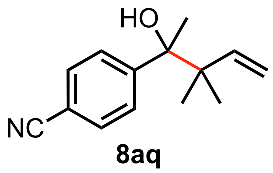

8aq

\\172.20.1.218\share_Instruments_Data_Data_NMR_files\Arii\NMRdata_complete\SA1029-proton-1-1.als

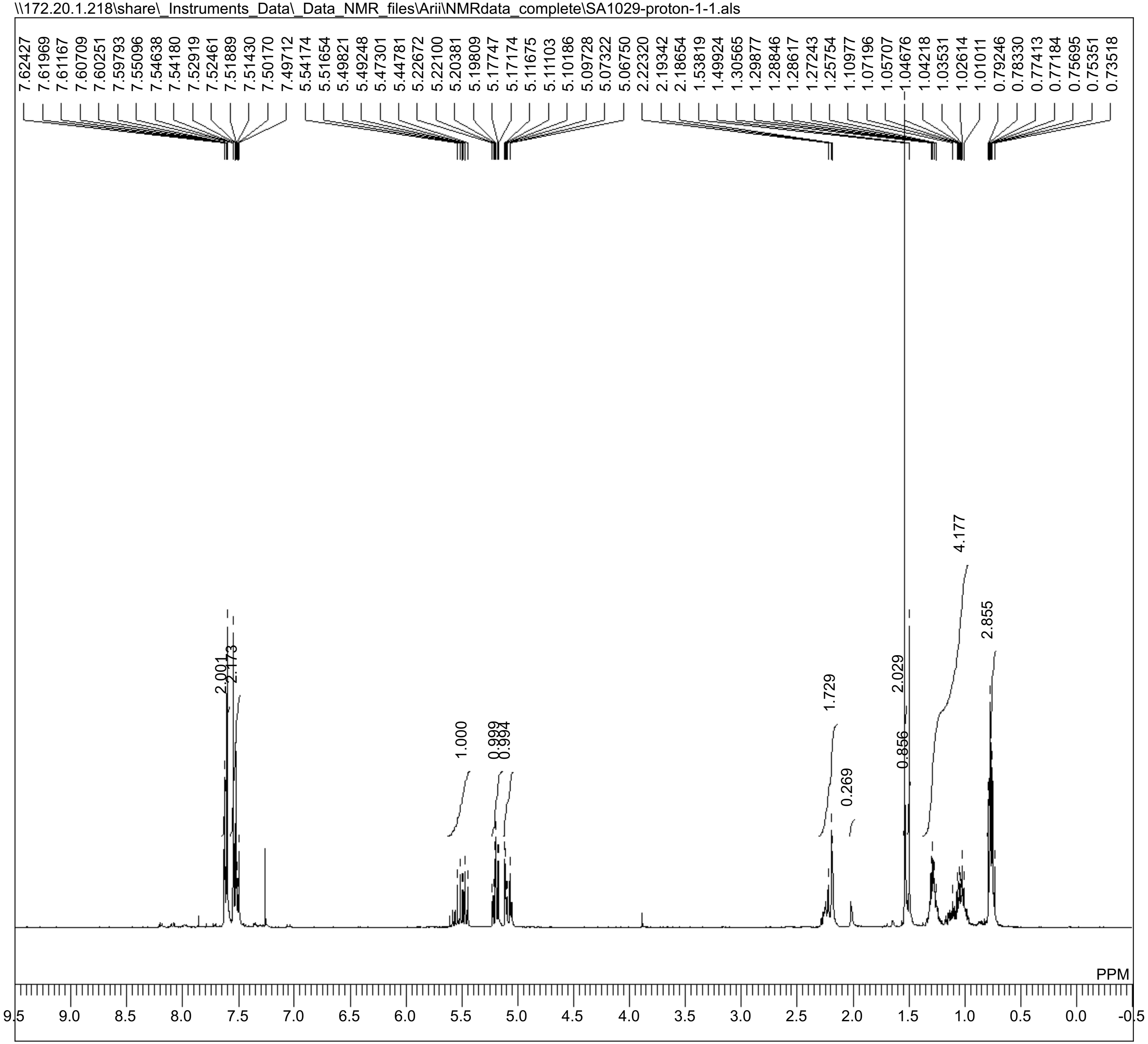

DFILE	SA1029-proton-1-1.als
COMNT	
DATIM	2025-01-19 08:39:54
OBNUC	1H
EXMOD	proton.jxp
OBFRQ	391.78 MHz
OBSET	8.51 KHz
OBFIN	3.34 Hz
POINT	13107
FREQU	5882.35 Hz
SCANS	8
ACQTM	2.2282 sec
PD	4.0000 sec
PW1	6.30 usec
IRNUC	1H
CTEMP	20.3 c
SLVNT	CDCL3
EXREF	7.26 ppm
BF	1.02 Hz
RGAIN	30

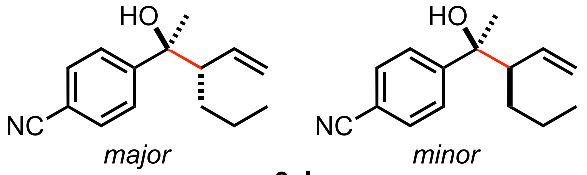

8al

\\172.20.1.218\share_Instruments_Data_Data_NMR_files\Arii\NMRdata_complete\SA1029-carbon-1-1.als

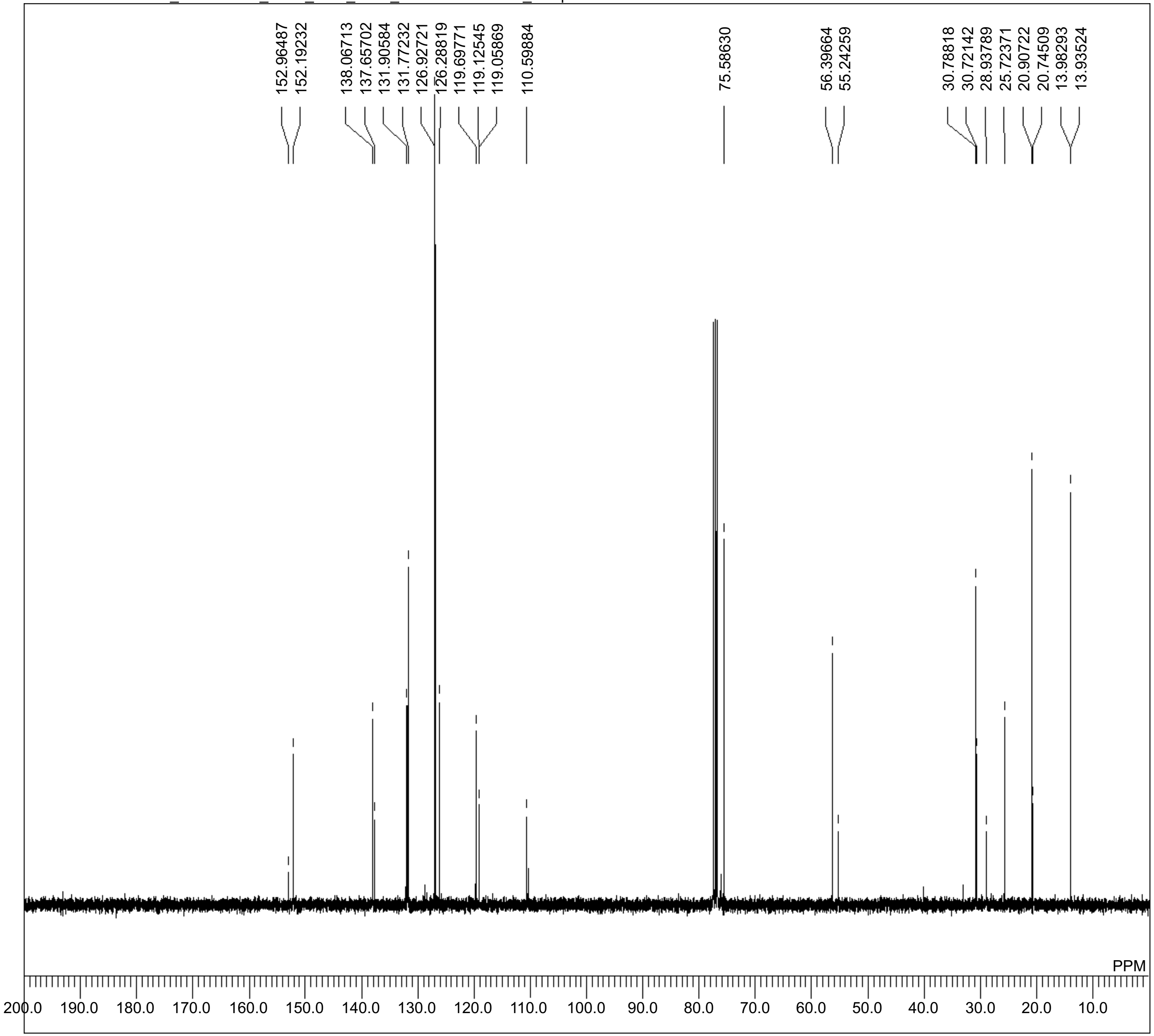

DFILE	SA1029-carbon-1-1.als
COMNT	
DATIM	2025-01-19 08:41:21
OBNUC	13C
EXMOD	carbon.jxp
OBFRQ	98.52 MHz
OBSET	4.64 KHz
OBFIN	8.74 Hz
POINT	26214
FREQU	24630.54 Hz
SCANS	552
ACQTM	1.0643 sec
PD	2.0000 sec
PW1	2.93 usec
IRNUC	1H
CTEMP	20.5 c
SLVNT	CDCL3
EXREF	77.16 ppm
BF	1.02 Hz
RGAIN	60

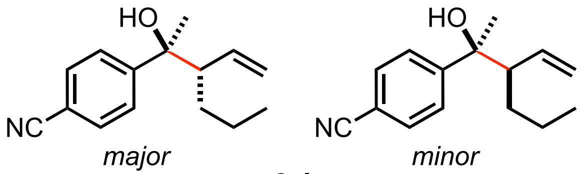

8al

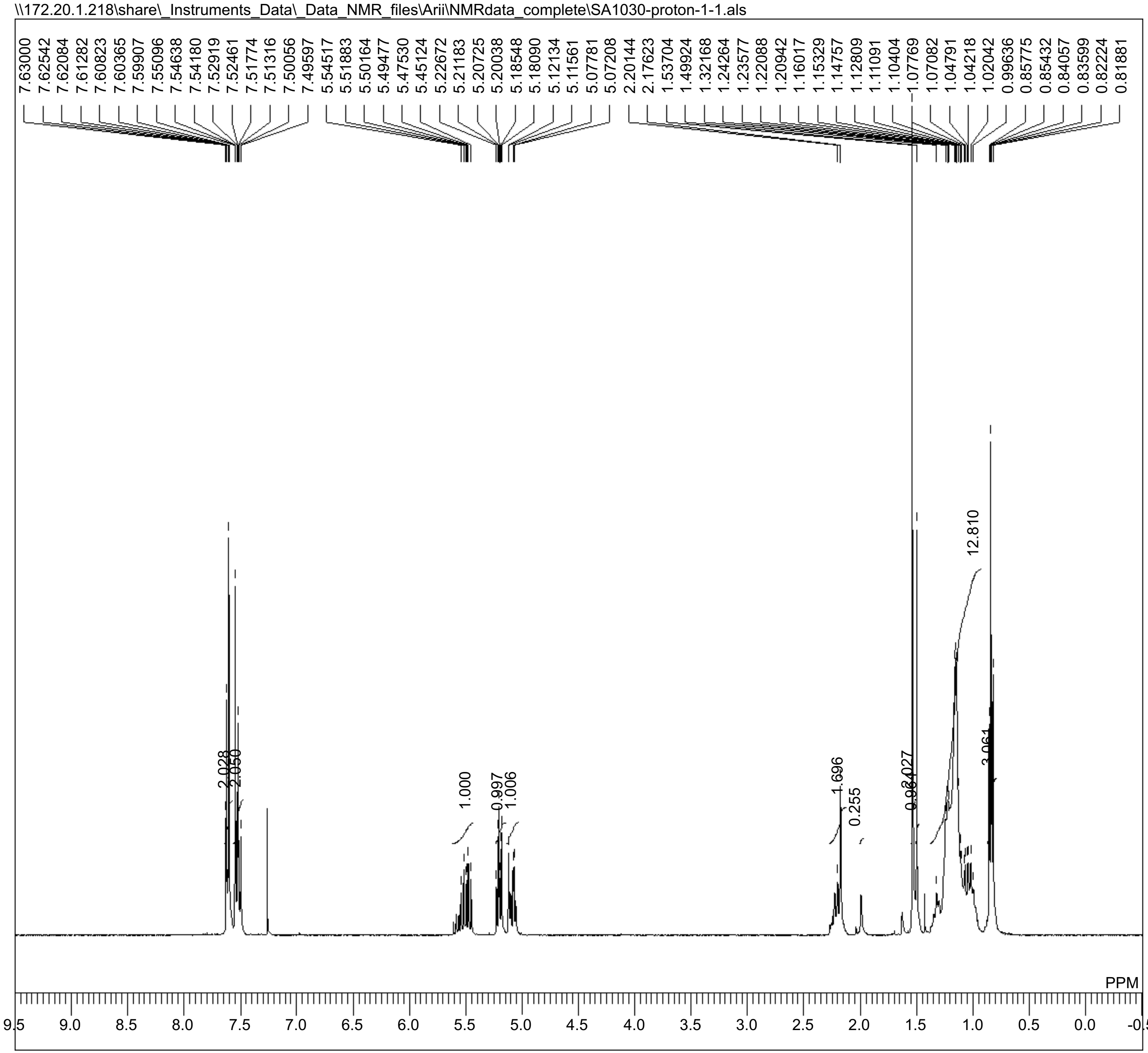

DFILE	SA1030-proton-1-1.als
COMNT	
DATIM	2025-01-23 01:12:39
OBNUC	1H
EXMOD	proton.jxp
OBFRQ	391.78 MHz
OBSET	8.51 KHz
OBFIN	3.34 Hz
POINT	13107
FREQU	5882.35 Hz
SCANS	8
ACQTM	2.2282 sec
PD	4.0000 sec
PW1	6.30 usec
IRNUC	1H
CTEMP	20.6 c
SLVNT	CDCL3
EXREF	7.26 ppm
BF	0.12 Hz
RGAIN	32

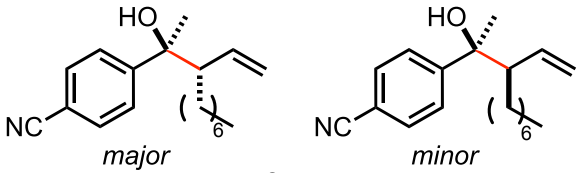

8am

\\172.20.1.218\share_Instruments_Data_Data_NMR_files\Arii\NMRdata_complete\SA1030-carbon-1-1.als

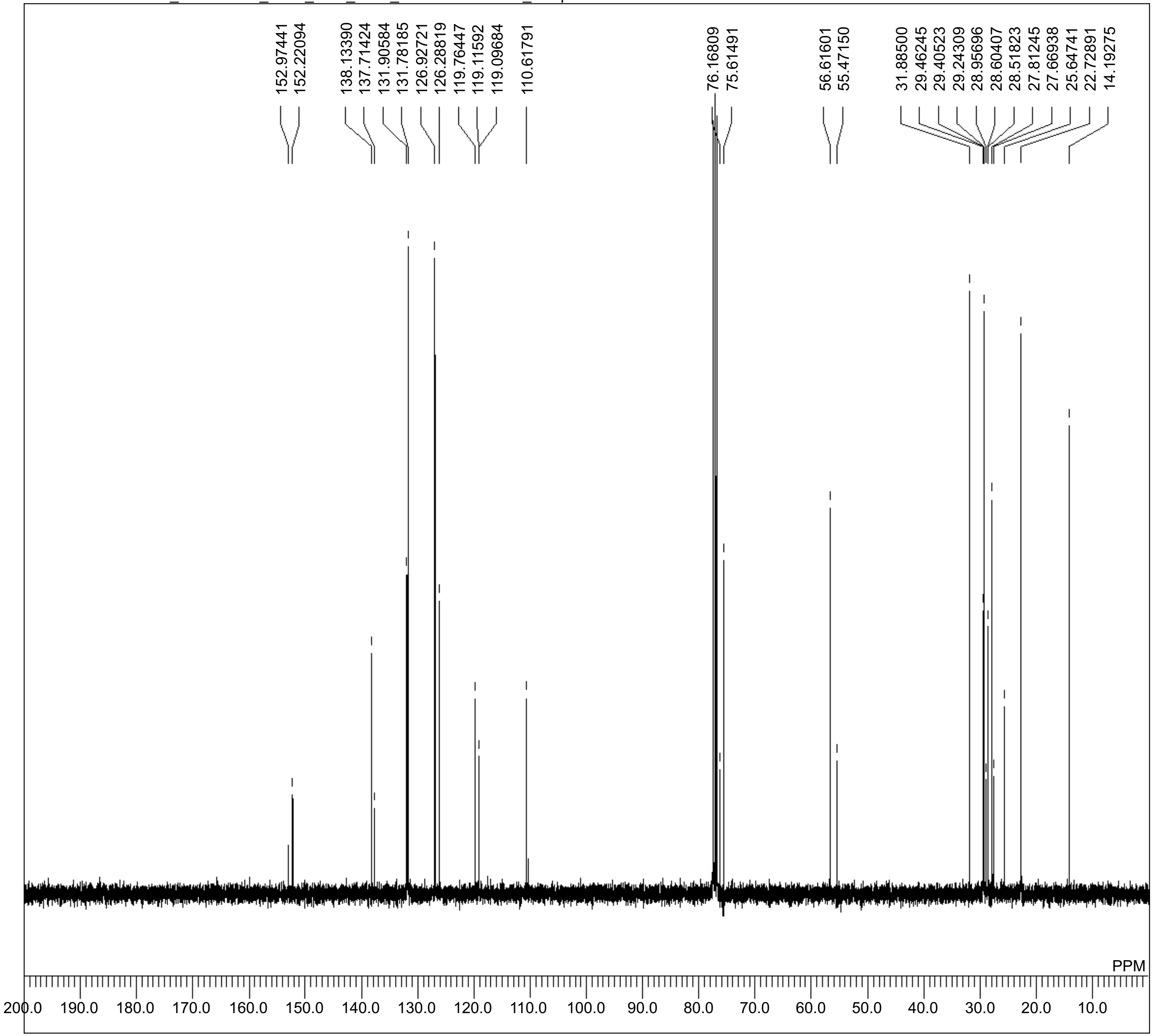

DFILE	SA1030-carbon-1-1.als
COMNT	
DATIM	2025-01-23 01:14:12
OBNUC	13C
EXMOD	carbon.jxp
OBFRQ	98.52 MHz
OBSET	4.64 KHz
OBFIN	8.74 Hz
POINT	26214
FREQU	24630.54 Hz
SCANS	374
ACQTM	1.0643 sec
PD	2.0000 sec
PW1	2.93 usec
IRNUC	1H
CTEMP	20.8 c
SLVNT	CDCL3
EXREF	77.16 ppm
BF	0.12 Hz
RGAIN	60

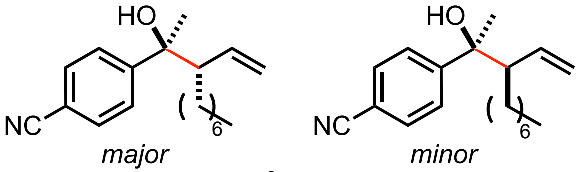

8am

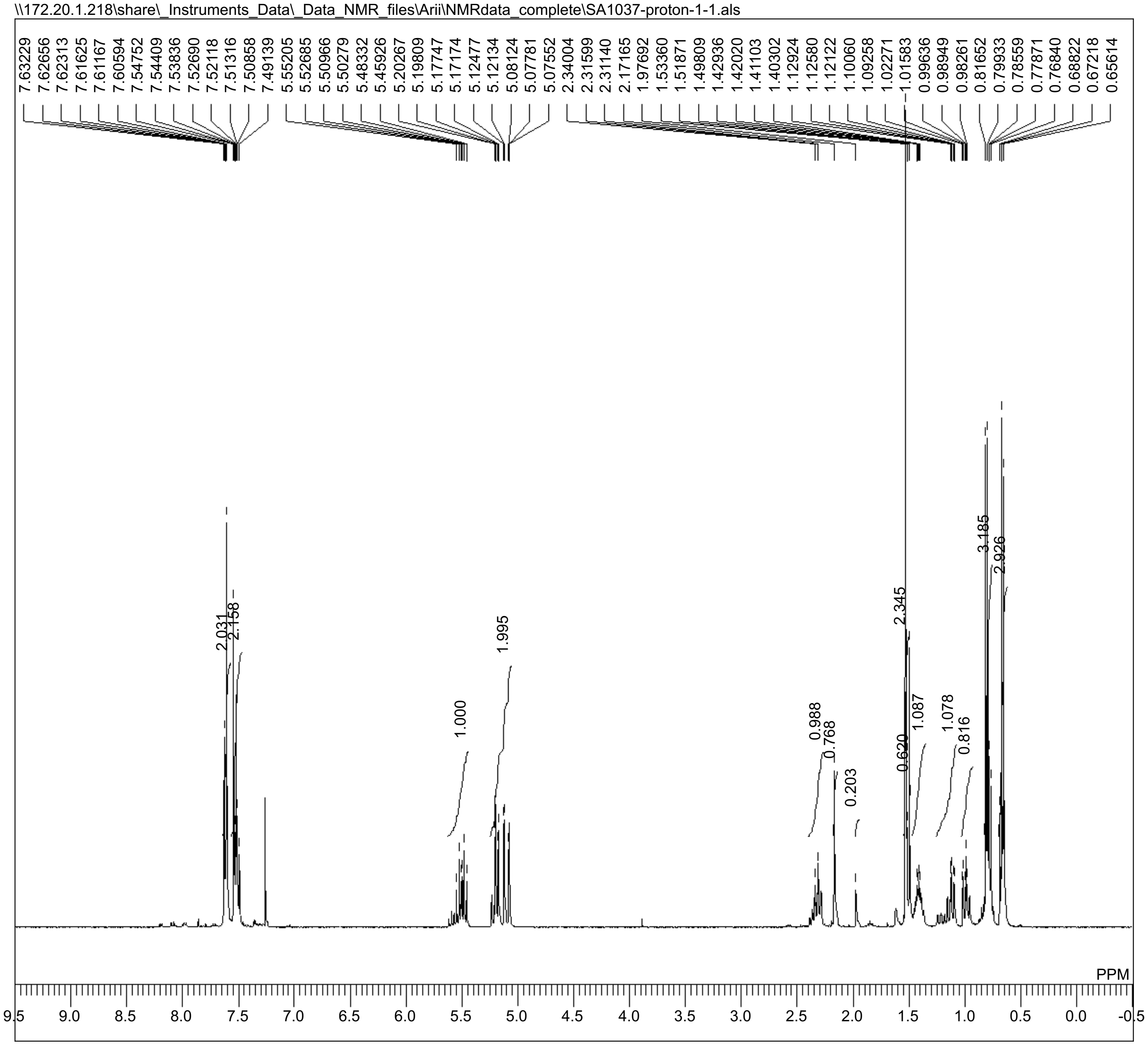

DFILE	SA1037-proton-1-1.als
COMNT	
DATIM	2025-01-20 14:29:52
OBNUC	1H
EXMOD	proton.jxp
OBFRQ	391.78 MHz
OBSET	8.51 KHz
OBFIN	3.34 Hz
POINT	13107
FREQU	5882.35 Hz
SCANS	8
ACQTM	2.2282 sec
PD	4.0000 sec
PW1	6.30 usec
IRNUC	1H
CTEMP	20.5 c
SLVNT	CDCL3
EXREF	7.26 ppm
BF	1.02 Hz
RGAIN	32

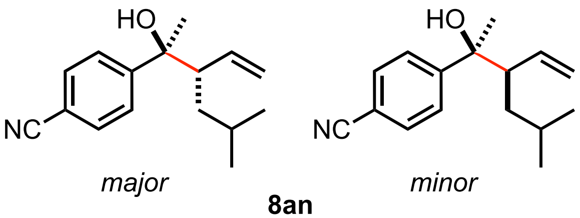

8an

\\172.20.1.218\share_Instruments_Data_Data_NMR_files\Arii\NMRdata_complete\SA1037-carbon-1-1.als

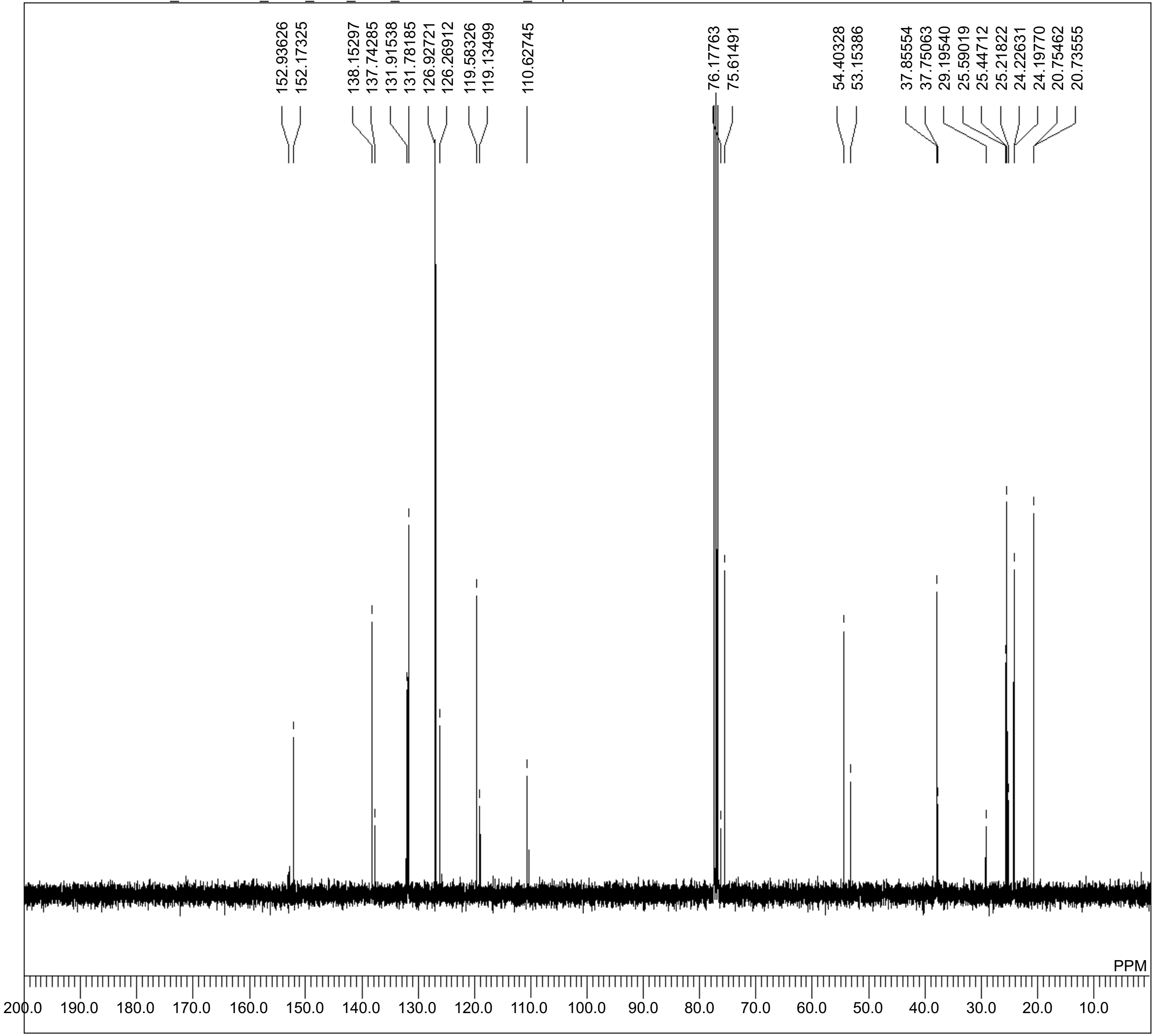

DFILE	SA1037-carbon-1-1.als
COMNT	
DATIM	2025-01-20 14:31:24
OBNUC	13C
EXMOD	carbon.jxp
OBFRQ	98.52 MHz
OBSET	4.64 KHz
OBFIN	8.74 Hz
POINT	26214
FREQU	24630.54 Hz
SCANS	420
ACQTM	1.0643 sec
PD	2.0000 sec
PW1	2.93 usec
IRNUC	1H
CTEMP	20.7 c
SLVNT	CDCL3
EXREF	77.16 ppm
BF	1.02 Hz
RGAIN	60

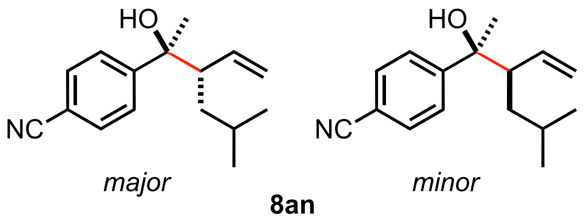

8an

\\172.20.1.218\share_Instruments_Data_Data_NMR_files\Arii\NMRdata_complete\SA1038-proton-1-1.als

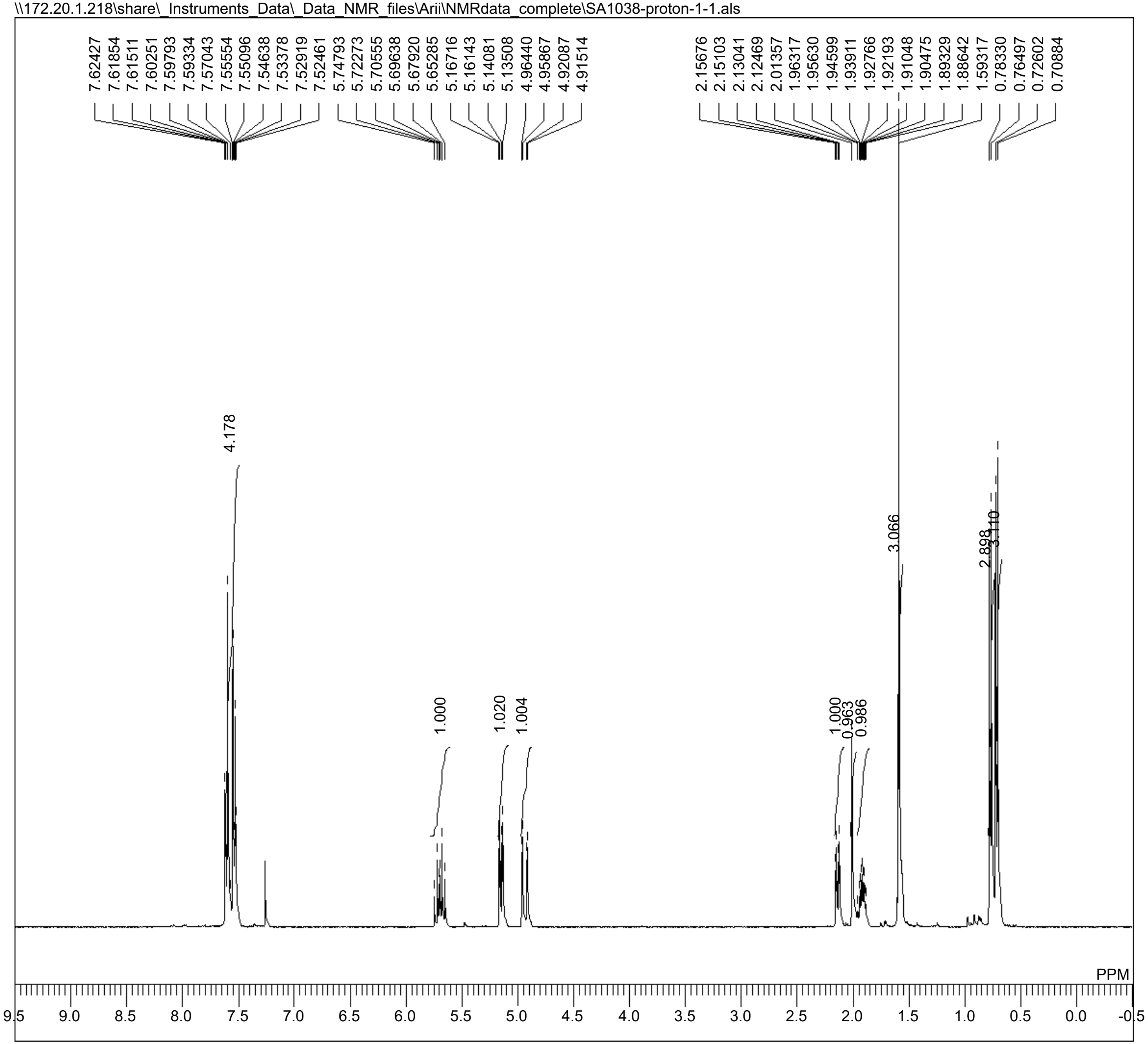

DFILE	SA1038-proton-1-1.als
COMNT	
DATIM	2025-01-20 14:22:34
OBNUC	1H
EXMOD	proton.jxp
OBFRQ	391.78 MHz
OBSET	8.51 KHz
OBFIN	3.34 Hz
POINT	13107
FREQU	5882.35 Hz
SCANS	8
ACQTM	2.2282 sec
PD	4.0000 sec
PW1	6.30 usec
IRNUC	1H
CTEMP	20.5 c
SLVNT	CDCL3
EXREF	7.26 ppm
BF	1.02 Hz
RGAIN	36

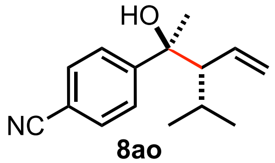

8ao

\\172.20.1.218\share_Instruments_Data_Data_NMR_files\Arii\NMRdata_complete\SA1038-carbon-1-1.als

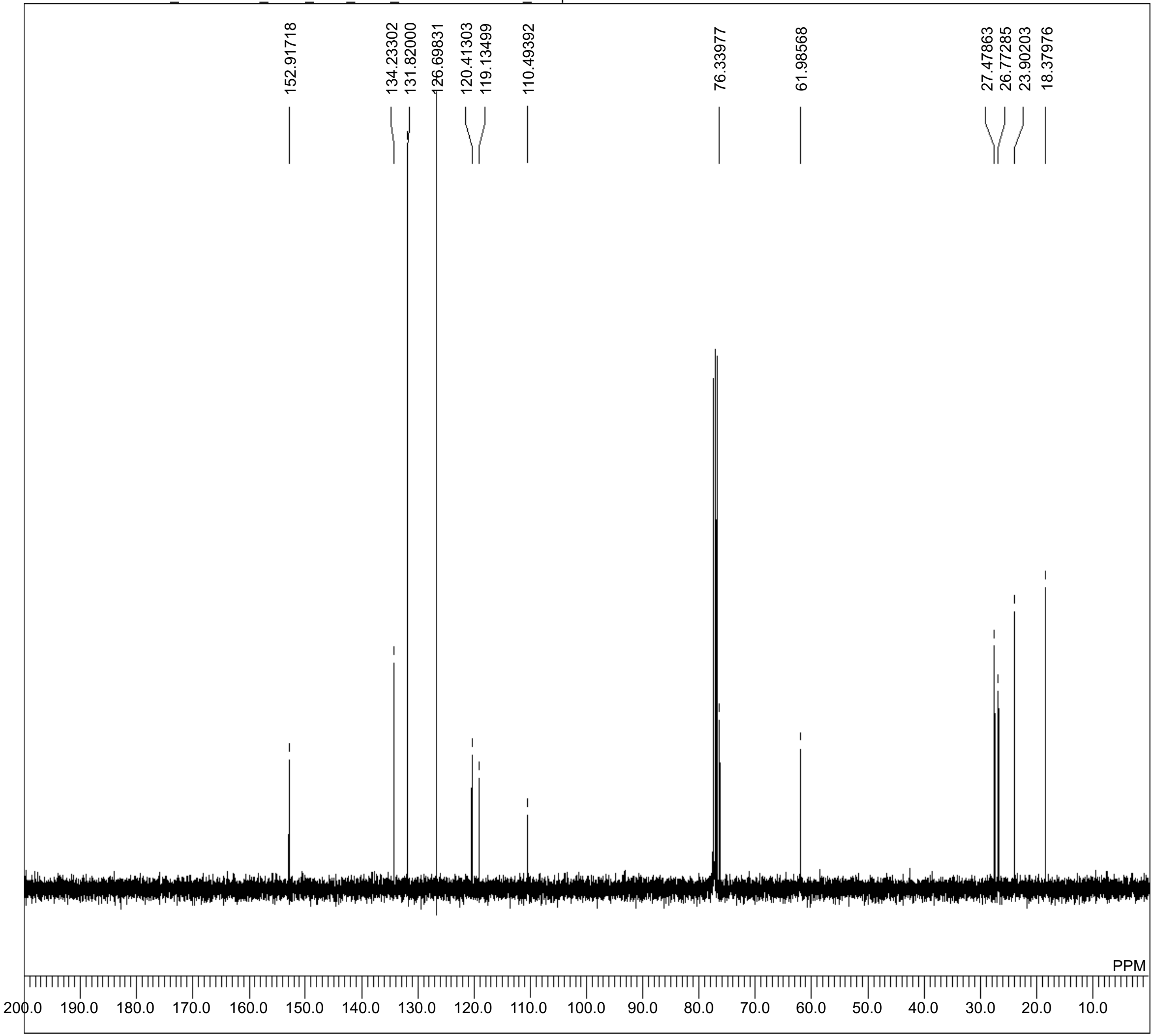

DFILE	SA1038-carbon-1-1.als
COMNT	
DATIM	2025-01-19 09:15:11
OBNUC	13C
EXMOD	carbon.jxp
OBFRQ	98.52 MHz
OBSET	4.64 KHz
OBFIN	8.74 Hz
POINT	26214
FREQU	24630.54 Hz
SCANS	199
ACQTM	1.0643 sec
PD	2.0000 sec
PW1	2.93 usec
IRNUC	1H
CTEMP	20.2 c
SLVNT	CDCL3
EXREF	77.16 ppm
BF	1.02 Hz
RGAIN	60

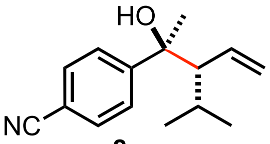

8ao

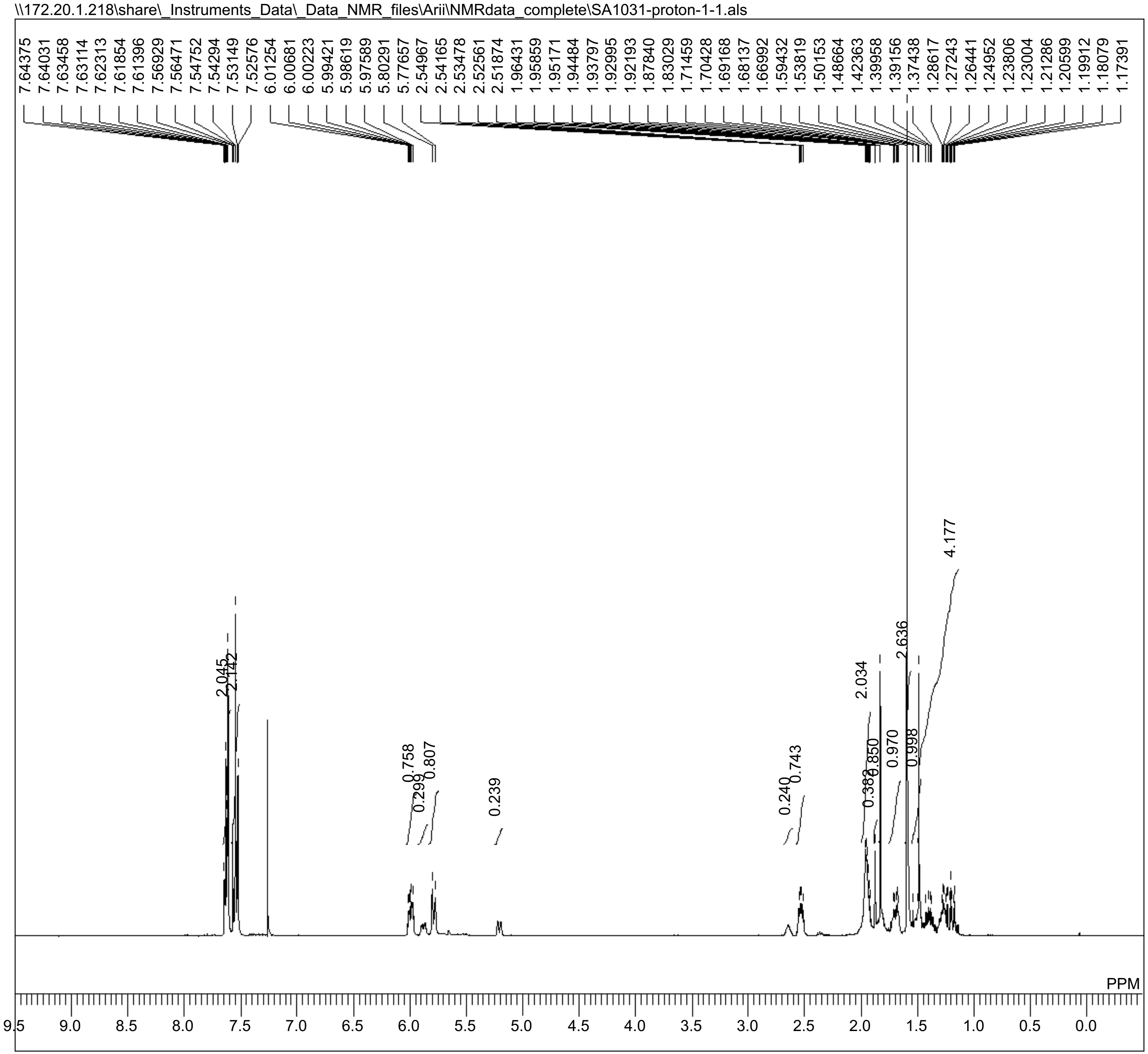

DFILE	SA1031-proton-1-1.als
COMNT	
DATIM	2025-01-20 16:23:11
OBNUC	1H
EXMOD	proton.jxp
OBFRQ	391.78 MHz
OBSET	8.51 KHz
OBFIN	3.34 Hz
POINT	13107
FREQU	5882.35 Hz
SCANS	8
ACQTM	2.2282 sec
PD	4.0000 sec
PW1	6.30 usec
IRNUC	1H
CTEMP	20.7 c
SLVNT	CDCL3
EXREF	7.26 ppm
BF	1.02 Hz
RGAIN	40

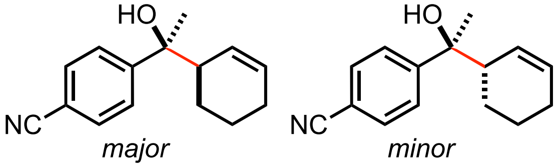

8ap

\\172.20.1.218\share_Instruments_Data_Data_NMR_files\Arii\NMRdata_complete\SA1031-carbon-1-1.als

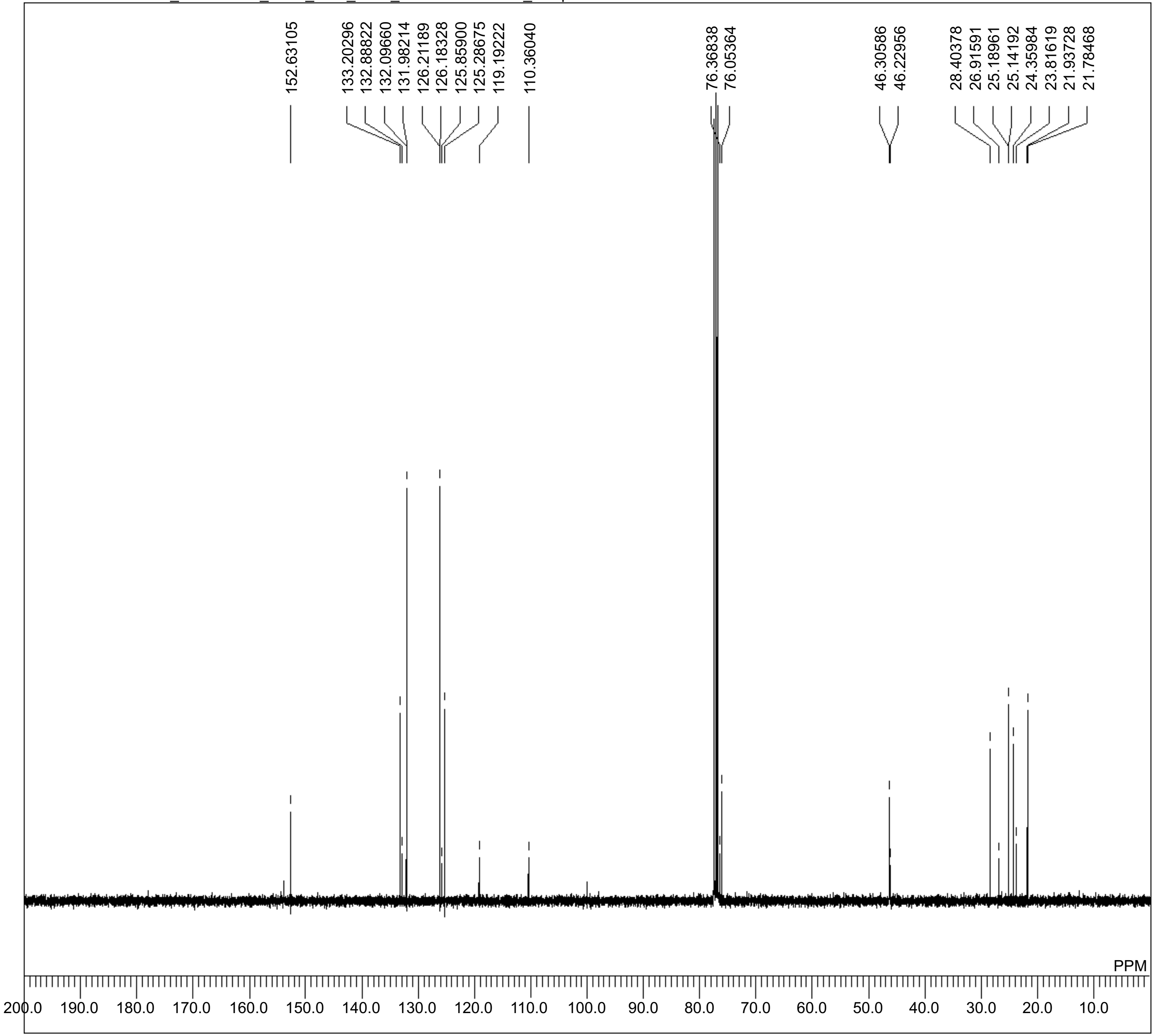

DFILE	SA1031-carbon-1-1.als
COMNT	
DATIM	2025-01-20 16:24:38
OBNUC	13C
EXMOD	carbon.jxp
OBFRQ	98.52 MHz
OBSET	4.64 KHz
OBFIN	8.74 Hz
POINT	26214
FREQU	24630.54 Hz
SCANS	1323
ACQTM	1.0643 sec
PD	2.0000 sec
PW1	2.93 usec
IRNUC	1H
CTEMP	21.1 c
SLVNT	CDCL3
EXREF	77.16 ppm
BF	1.02 Hz
RGAIN	60

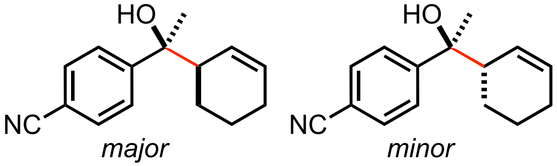

8ap

\\172.20.1.218\share_Instruments_Data_Data_NMR_files\Arii\NMRdata_complete\SA1066-proton-1-1.als

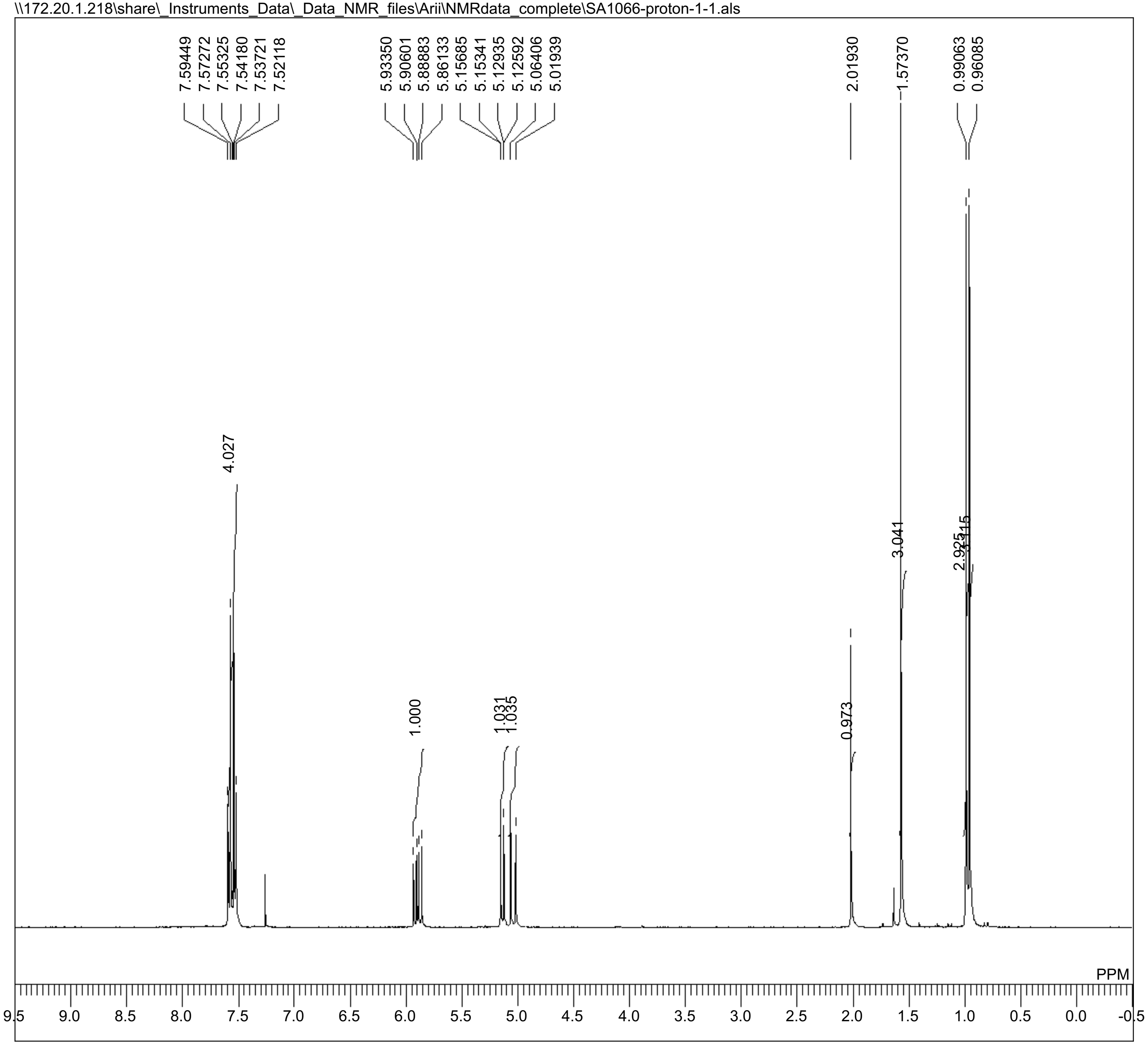

DFILE	SA1066-proton-1-1.als
COMNT	
DATIM	2025-01-24 16:14:30
OBNUC	1H
EXMOD	proton.jxp
OBFRQ	391.78 MHz
OBSET	8.51 KHz
OBFIN	3.34 Hz
POINT	13107
FREQU	5882.35 Hz
SCANS	8
ACQTM	2.2282 sec
PD	4.0000 sec
PW1	6.30 usec
IRNUC	1H
CTEMP	21.1 c
SLVNT	CDCL3
EXREF	7.26 ppm
BF	0.12 Hz
RGAIN	36

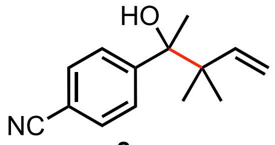

8aq

\\172.20.1.218\share_Instruments_Data_Data_NMR_files\Arii\NMRdata_complete\SA1066-carbon-1-1.als

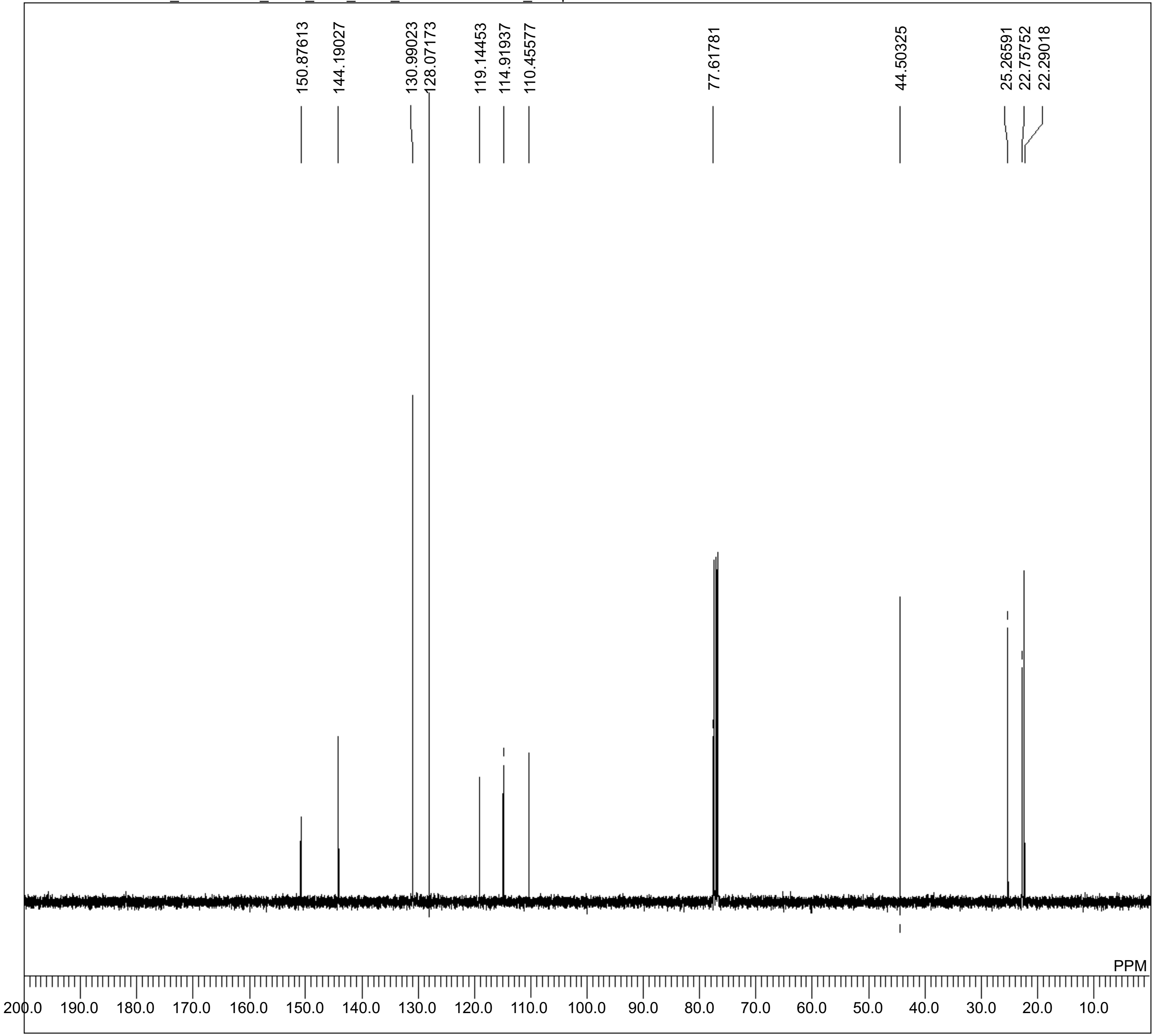

DFILE	SA1066-carbon-1-1.als
COMNT	
DATIM	2025-01-24 16:16:56
OBNUC	13C
EXMOD	carbon.jxp
OBFRQ	98.52 MHz
OBSET	4.64 KHz
OBFIN	8.74 Hz
POINT	26214
FREQU	24630.54 Hz
SCANS	267
ACQTM	1.0643 sec
PD	2.0000 sec
PW1	2.93 usec
IRNUC	1H
CTEMP	21.3 c
SLVNT	CDCL3
EXREF	77.16 ppm
BF	0.12 Hz
RGAIN	60

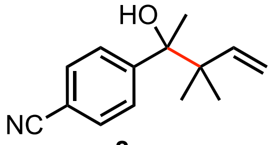

8aq

\\172.20.1.218\share_Instruments_Data_Data_NMR_files\Arii\NMRdata_complete\SA1032-proton-1-1.als

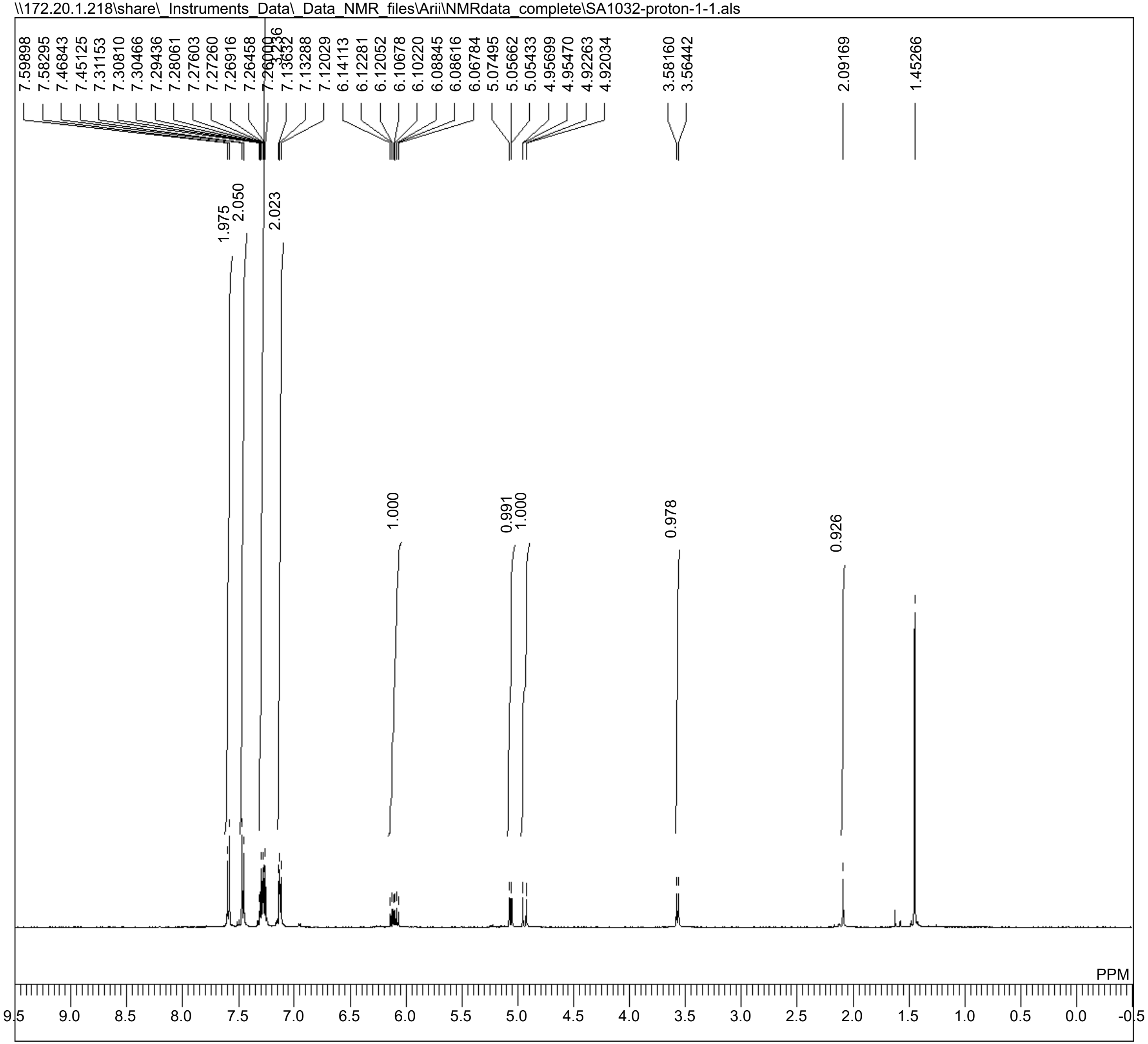

DFILE	SA1032-proton-1-1.als
COMNT	
DATIM	2025-01-19 07:33:06
OBNUC	1H
EXMOD	proton.jxp
OBFRQ	500.16 MHz
OBSET	2.41 KHz
OBFIN	6.01 Hz
POINT	13107
FREQU	7507.51 Hz
SCANS	8
ACQTM	1.7459 sec
PD	5.0000 sec
PW1	5.55 usec
IRNUC	1H
CTEMP	21.2 c
SLVNT	CDCL3
EXREF	7.26 ppm
BF	0.42 Hz
RGAIN	30

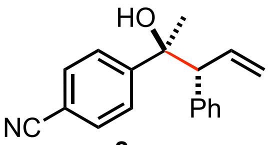

8ar

\\172.20.1.218\share_Instruments_Data_Data_NMR_files\Arii\NMRdata_complete\SA1032-carbon-1-1.als

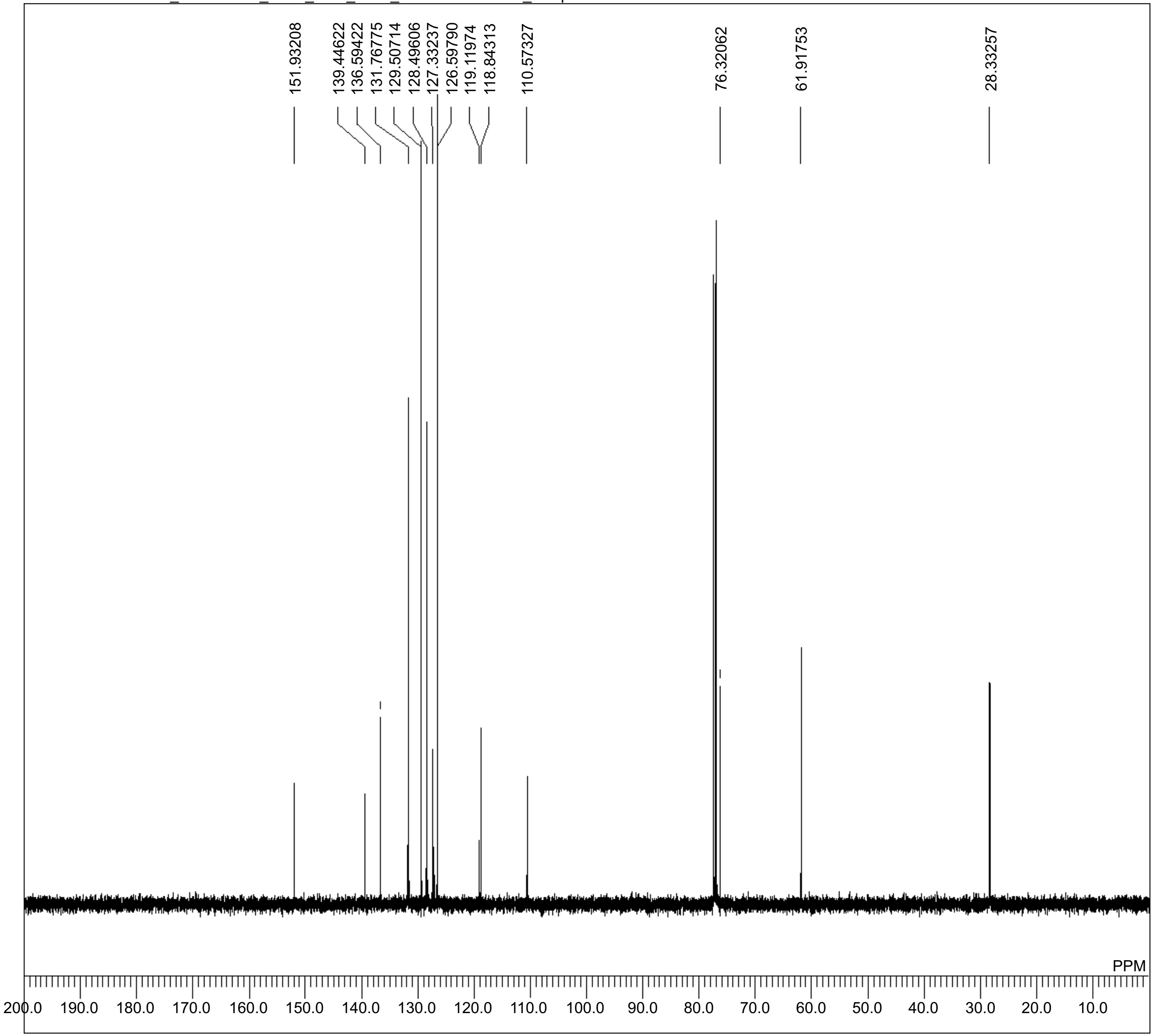

DFILE	SA1032-carbon-1-1.als
COMNT	
DATIM	2025-01-19 07:34:54
OBNUC	13C
EXMOD	carbon.jxp
OBFRQ	125.77 MHz
OBSET	7.87 KHz
OBFIN	4.21 Hz
POINT	26214
FREQU	31446.54 Hz
SCANS	630
ACQTM	0.8336 sec
PD	1.0000 sec
PW1	3.40 usec
IRNUC	1H
CTEMP	21.6 c
SLVNT	CDCL3
EXREF	77.16 ppm
BF	0.42 Hz
RGAIN	60

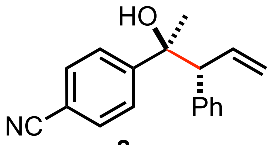

8ar

\\172.20.1.218\share_Instruments_Data_Data_NMR_files\Arii\NMRdata_complete\SA1070-proton-1-1.als

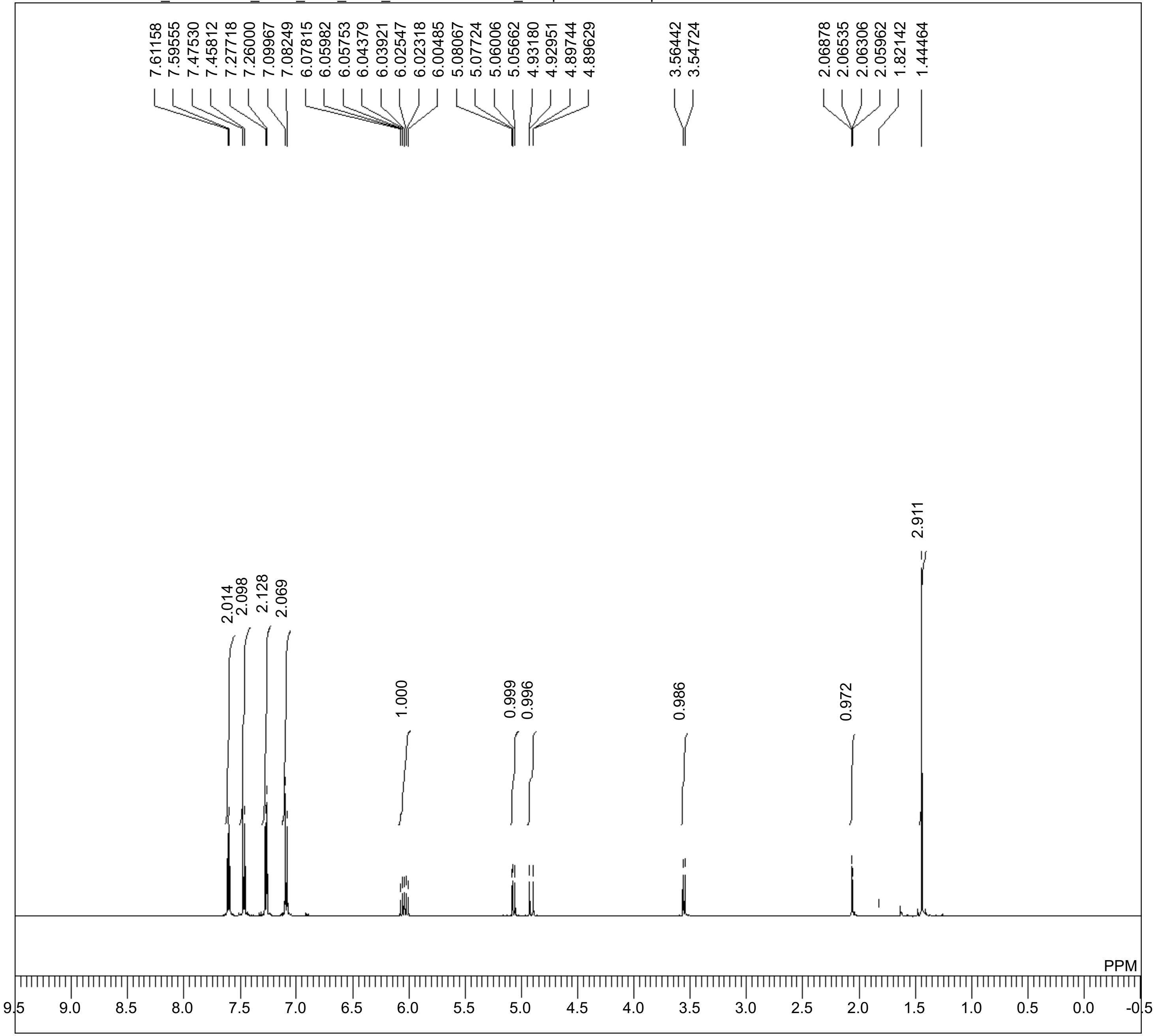

DFILE	SA1070-proton-1-1.als
COMNT	
DATIM	2025-01-25 17:58:10
OBNUC	1H
EXMOD	proton.jxp
OBFRQ	500.16 MHz
OBSET	2.41 KHz
OBFIN	6.01 Hz
POINT	13107
FREQU	7507.51 Hz
SCANS	8
ACQTM	1.7459 sec
PD	5.0000 sec
PW1	5.55 usec
IRNUC	1H
CTEMP	21.5 c
SLVNT	CDCL3
EXREF	7.26 ppm
BF	0.72 Hz
RGAIN	30

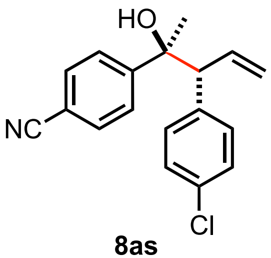

8as

\\172.20.1.218\share_Instruments_Data_Data_NMR_files\Arii\NMRdata_complete\SA1070-carbon-1-1.als

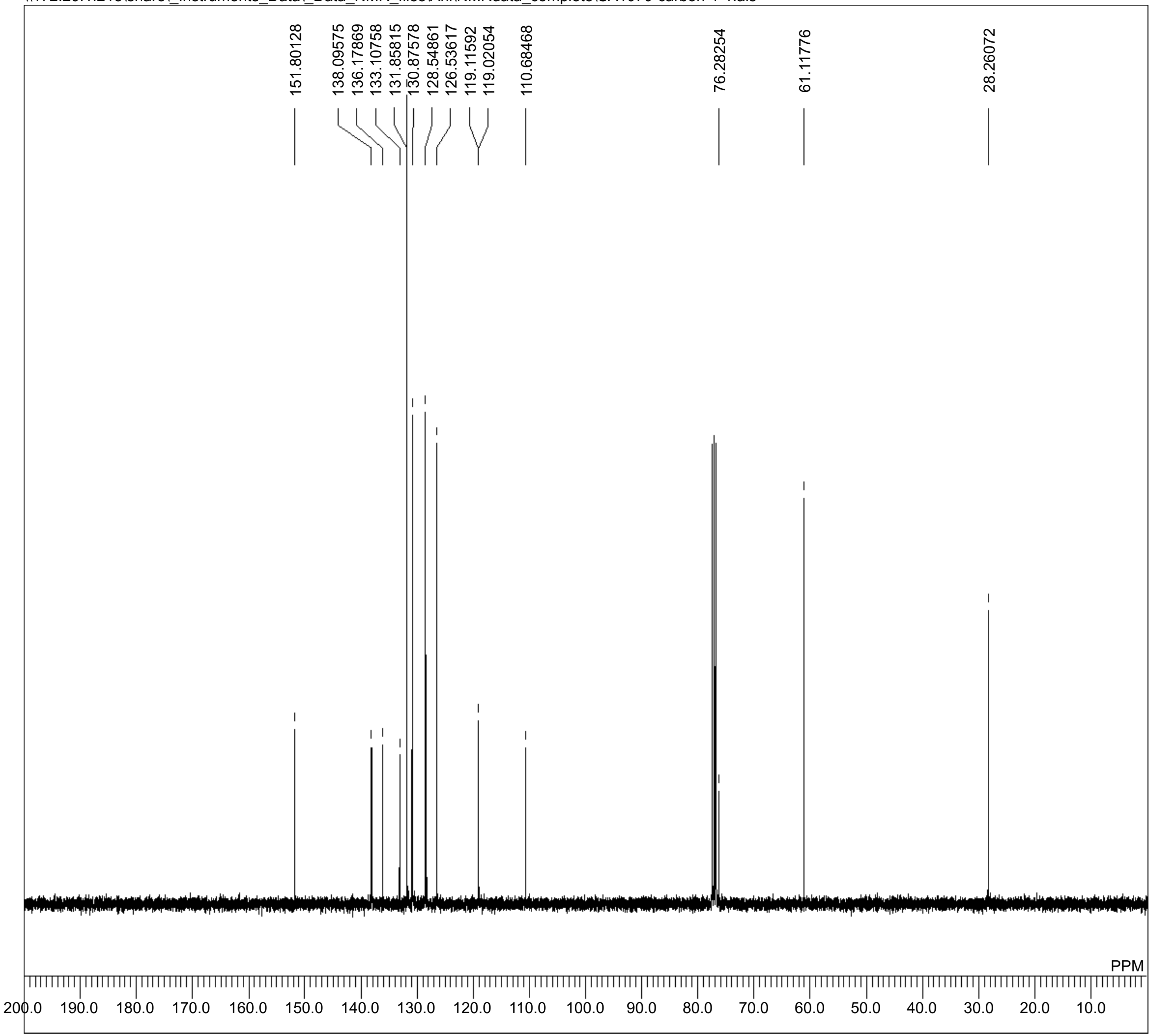

DFILE	SA1070-carbon-1-1.als
COMNT	
DATIM	2025-01-25 20:50:08
OBNUC	13C
EXMOD	carbon.jxp
OBFRQ	98.52 MHz
OBSET	4.64 KHz
OBFIN	8.74 Hz
POINT	26214
FREQU	24630.54 Hz
SCANS	330
ACQTM	1.0643 sec
PD	2.0000 sec
PW1	2.93 usec
IRNUC	1H
CTEMP	20.7 c
SLVNT	CDCL3
EXREF	77.16 ppm
BF	0.12 Hz
RGAIN	60

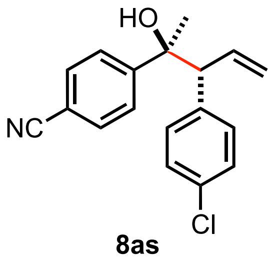

8as

\\172.20.1.218\share_Instruments_Data_Data_NMR_files\Arii\NMRdata_complete\SA1036-proton-1-1.als

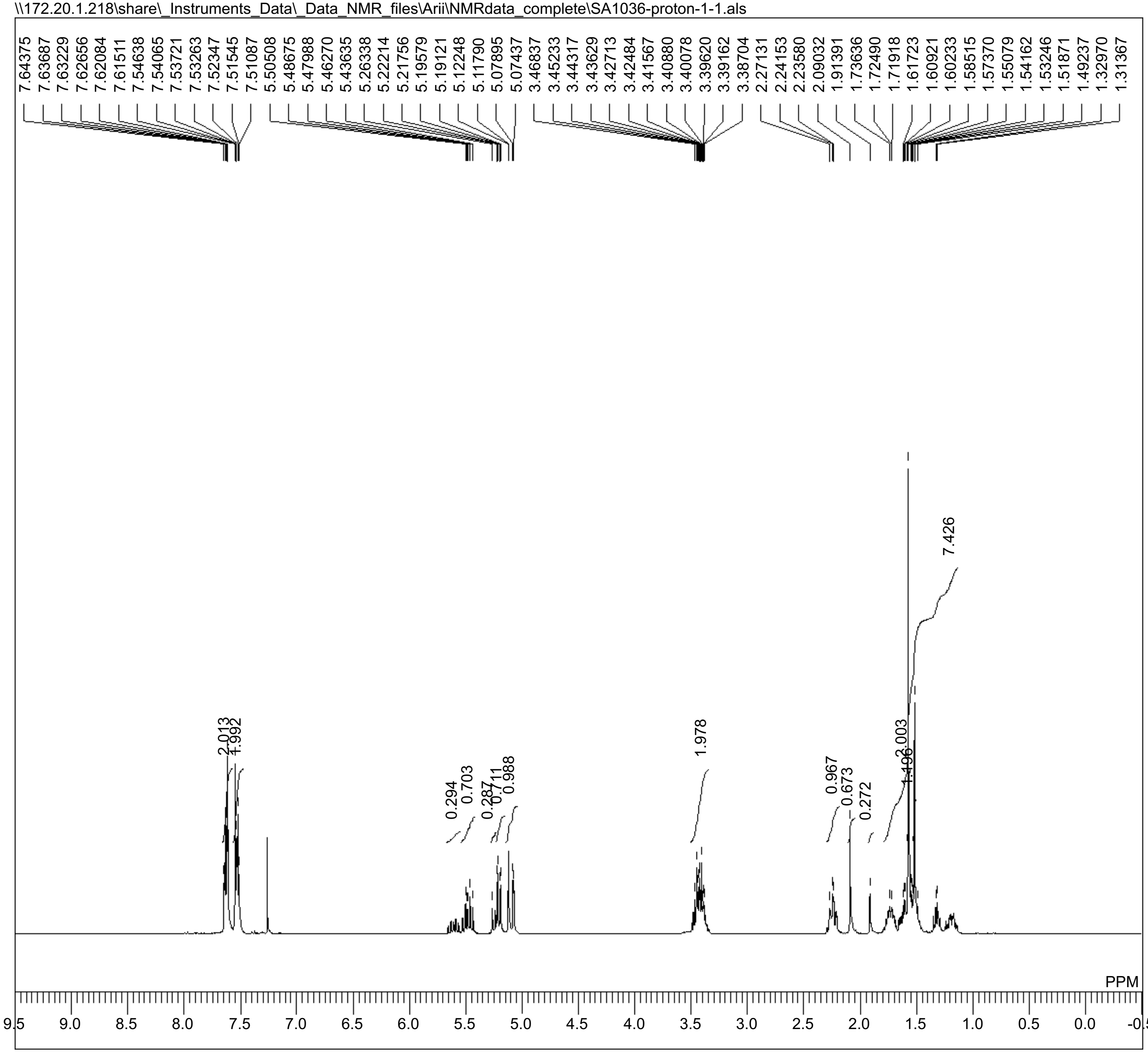

DFILE	SA1036-proton-1-1.als
COMNT	
DATIM	2025-01-23 01:39:53
OBNUC	1H
EXMOD	proton.jxp
OBFRQ	391.78 MHz
OBSET	8.51 KHz
OBFIN	3.34 Hz
POINT	13107
FREQU	5882.35 Hz
SCANS	8
ACQTM	2.2282 sec
PD	4.0000 sec
PW1	6.30 usec
IRNUC	1H
CTEMP	20.6 c
SLVNT	CDCL3
EXREF	7.26 ppm
BF	0.12 Hz
RGAIN	40

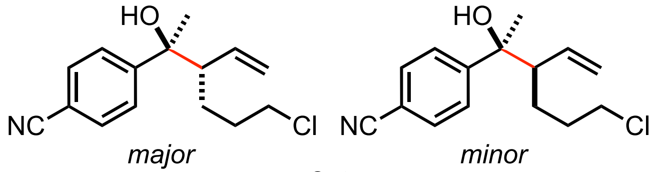

8at

\\172.20.1.218\share_Instruments_Data_Data_NMR_files\Arii\NMRdata_complete\SA1036-carbon-1-1.als

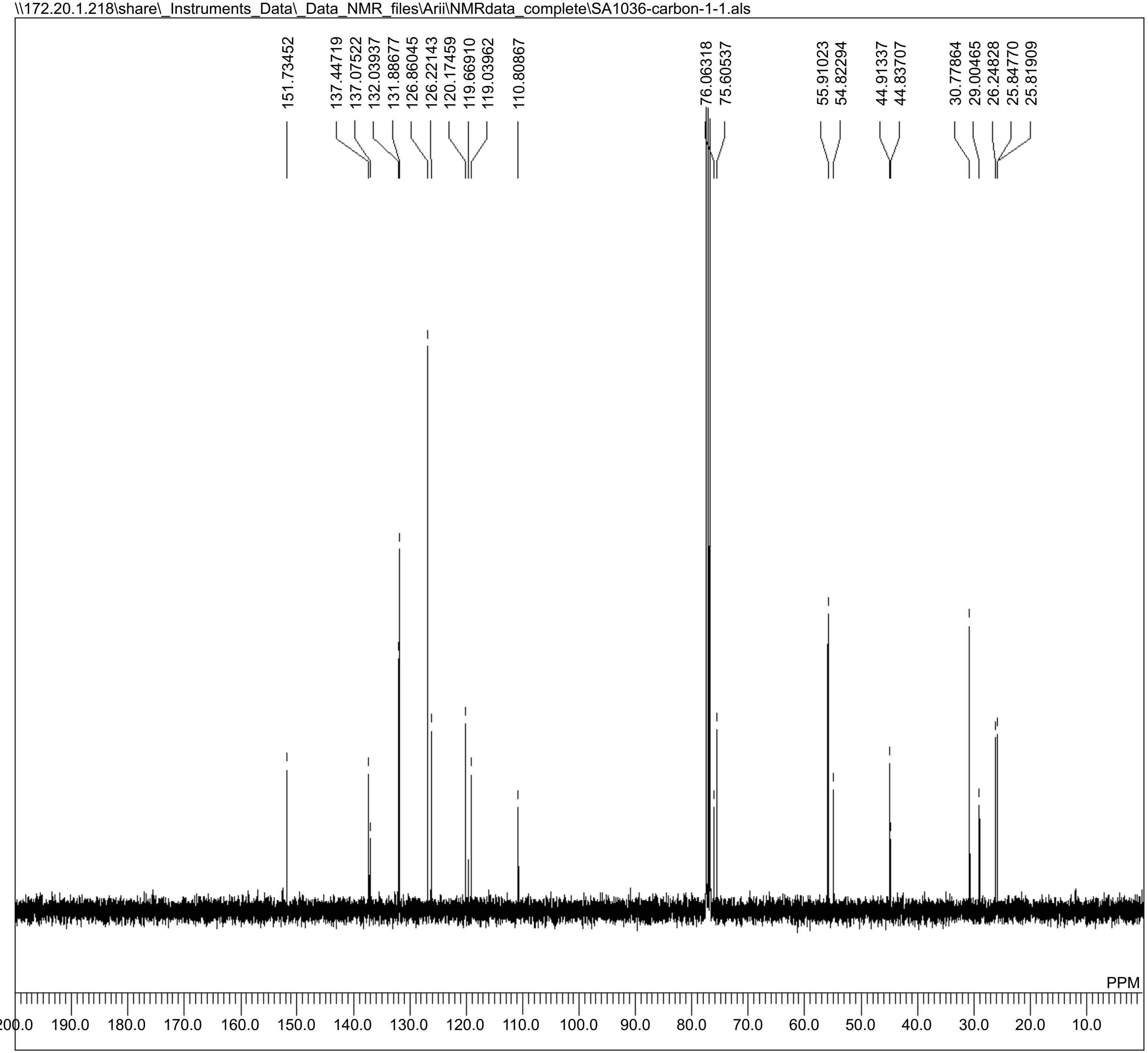

DFILE	SA1036-carbon-1-1.als
COMNT	
DATIM	2025-01-23 01:41:27
OBNUC	13C
EXMOD	carbon.jxp
OBFRQ	98.52 MHz
OBSET	4.64 KHz
OBFIN	8.74 Hz
POINT	26214
FREQU	24630.54 Hz
SCANS	348
ACQTM	1.0643 sec
PD	2.0000 sec
PW1	2.93 usec
IRNUC	1H
CTEMP	20.7 c
SLVNT	CDCL3
EXREF	77.16 ppm
BF	0.12 Hz
RGAIN	60

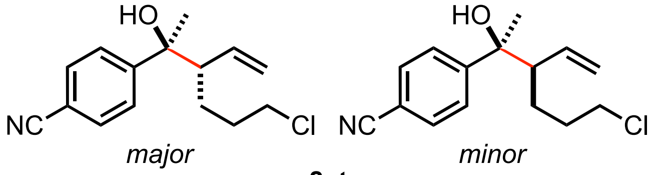

8at

\\172.20.1.218\share_Instruments_Data_Data_NMR_files\Arii\NMRdata_complete\SA1033-proton-1-1.als

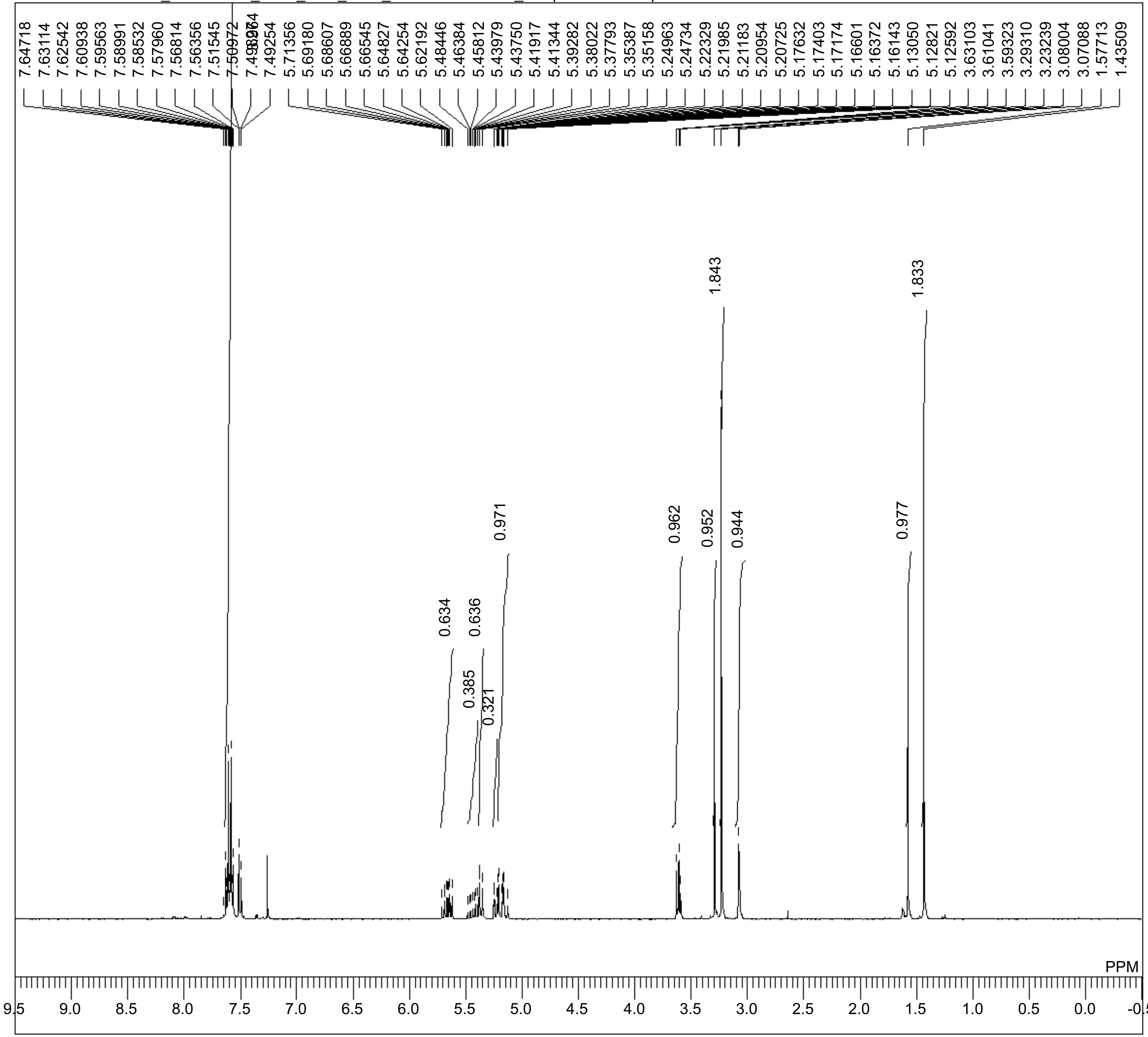

DFILE	SA1033-proton-1-1.als
COMNT	
DATIM	2025-01-20 15:36:02
OBNUC	1H
EXMOD	proton.jxp
OBFRQ	391.78 MHz
OBSET	8.51 KHz
OBFIN	3.34 Hz
POINT	13107
FREQU	5882.35 Hz
SCANS	8
ACQTM	2.2282 sec
PD	4.0000 sec
PW1	6.30 usec
IRNUC	1H
CTEMP	20.5 c
SLVNT	CDCL3
EXREF	7.26 ppm
BF	1.02 Hz
RGAIN	38

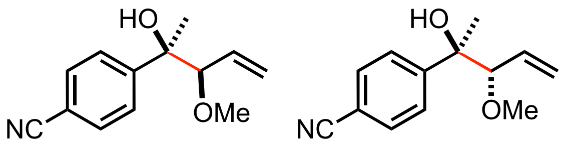

major *minor*

8au

\\172.20.1.218\share_Instruments_Data_Data_NMR_files\Arii\NMRdata_complete\SA1033-carbon-1-1.als

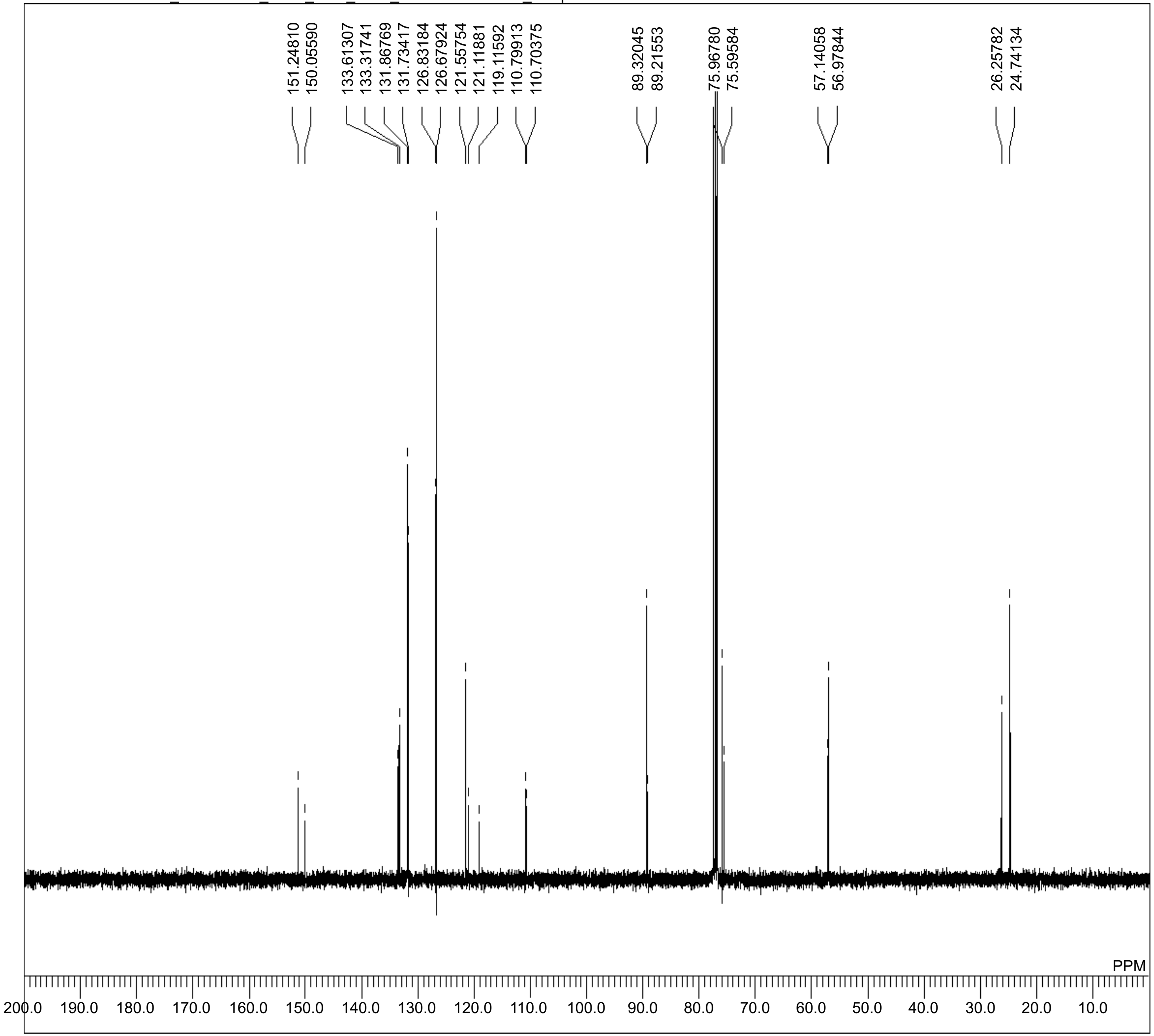

DFILE	SA1033-carbon-1-1.als
COMNT	
DATIM	2025-01-20 15:37:29
OBNUC	13C
EXMOD	carbon.jxp
OBFRQ	98.52 MHz
OBSET	4.64 KHz
OBFIN	8.74 Hz
POINT	26214
FREQU	24630.54 Hz
SCANS	796
ACQTM	1.0643 sec
PD	2.0000 sec
PW1	2.93 usec
IRNUC	1H
CTEMP	20.8 c
SLVNT	CDCL3
EXREF	77.16 ppm
BF	1.02 Hz
RGAIN	60

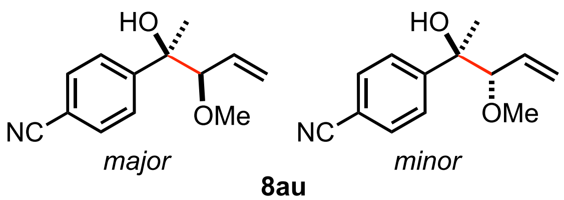

8au

\\172.20.1.218\share_Instruments_Data_Data_NMR_files\Arii\NMRdata_complete\SA1035-proton-1-1.als

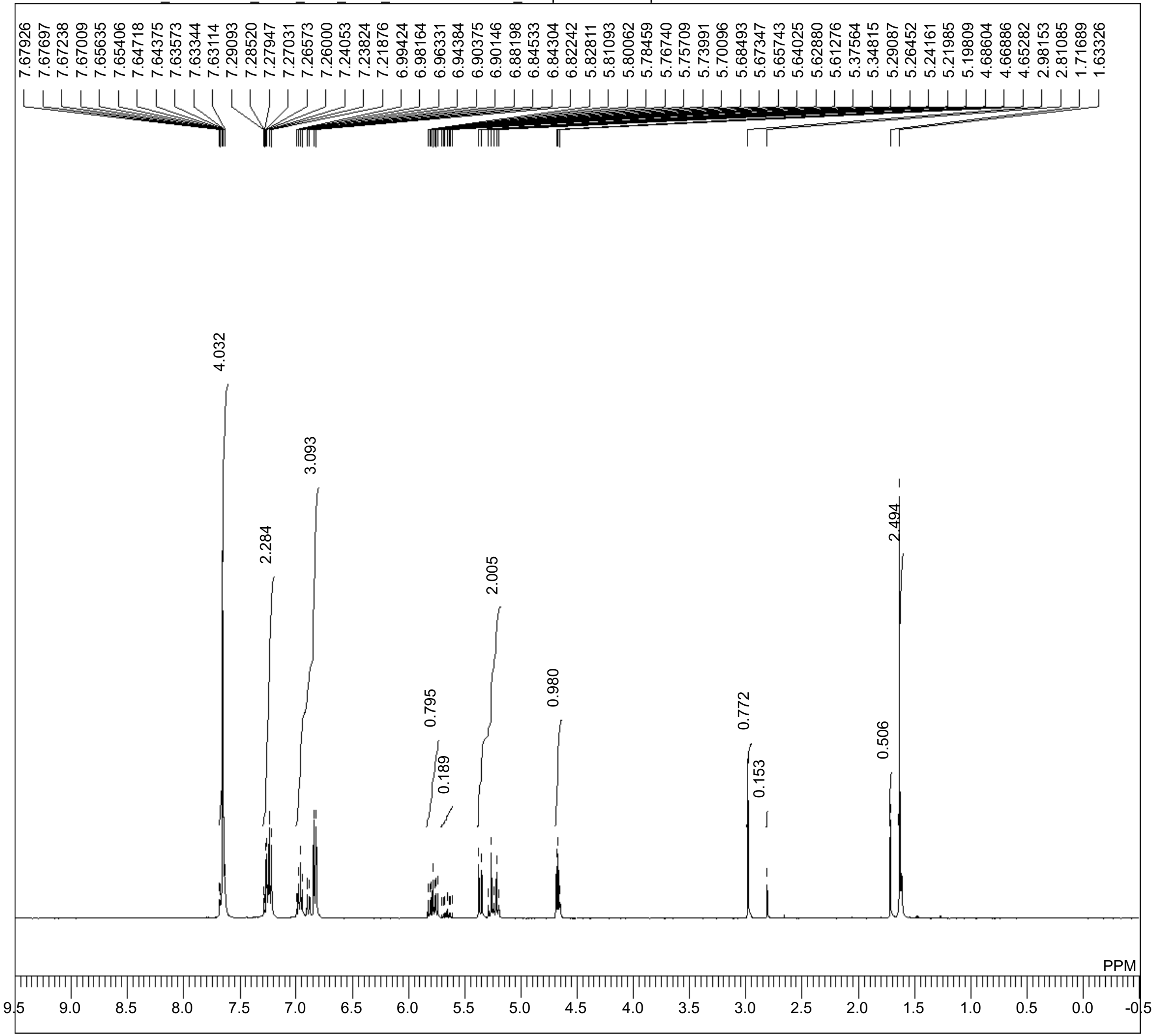

DFILE	SA1035-proton-1-1.als
COMNT	
DATIM	2025-01-20 14:59:08
OBNUC	1H
EXMOD	proton.jxp
OBFRQ	391.78 MHz
OBSET	8.51 KHz
OBFIN	3.34 Hz
POINT	13107
FREQU	5882.35 Hz
SCANS	8
ACQTM	2.2282 sec
PD	4.0000 sec
PW1	6.30 usec
IRNUC	1H
CTEMP	20.5 c
SLVNT	CDCL3
EXREF	7.26 ppm
BF	1.02 Hz
RGAIN	36

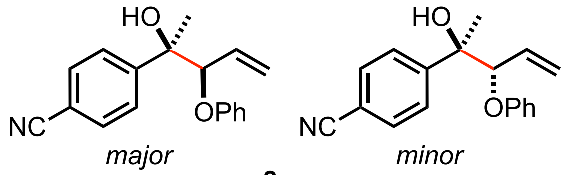

8av

\\172.20.1.218\share_Instruments_Data_Data_NMR_files\Arii\NMRdata_complete\SA1035-carbon-1-1.als

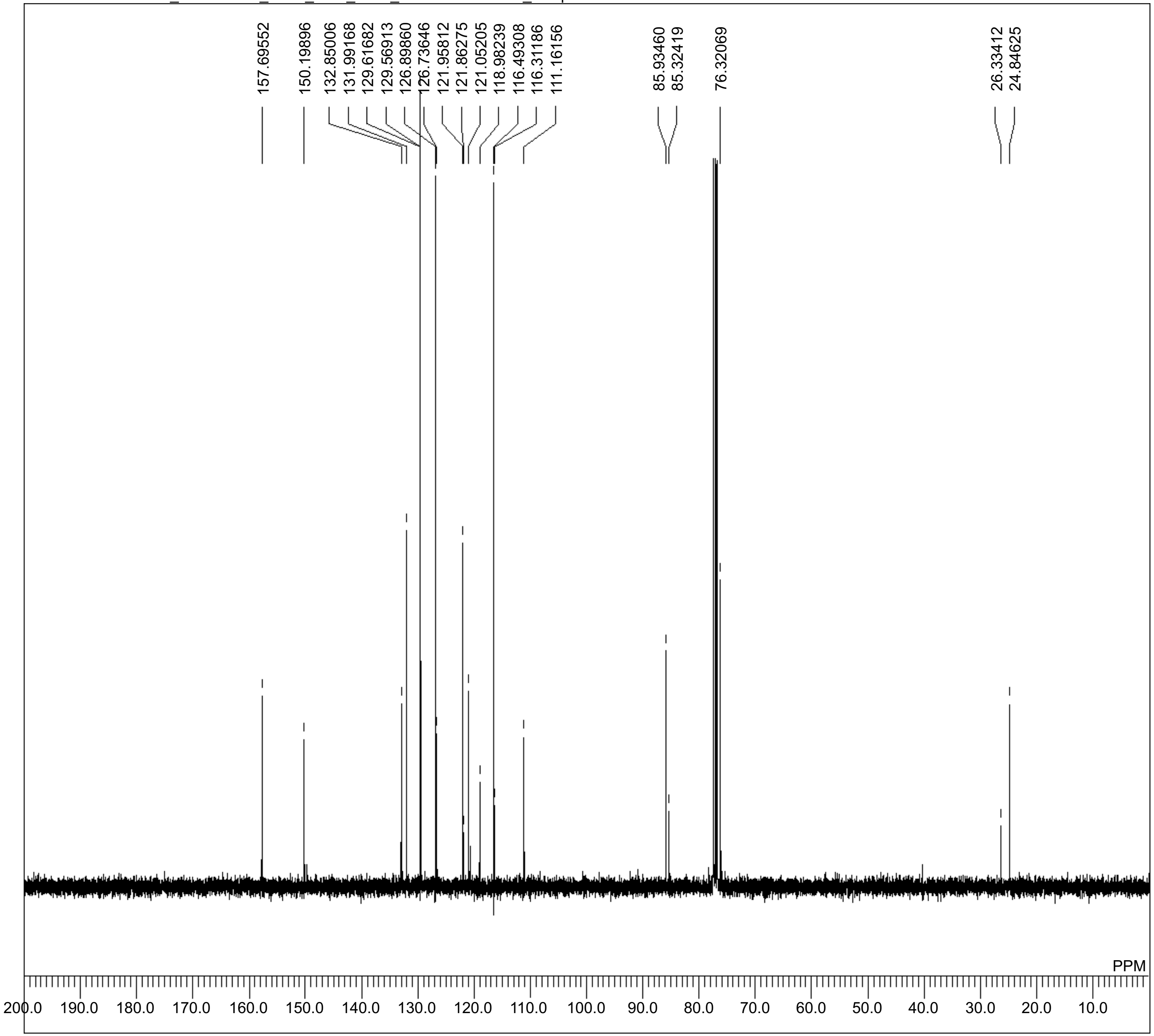

DFILE	SA1035-carbon-1-1.als
COMNT	
DATIM	2025-01-20 15:00:42
OBNUC	13C
EXMOD	carbon.jxp
OBFRQ	98.52 MHz
OBSET	4.64 KHz
OBFIN	8.74 Hz
POINT	26214
FREQU	24630.54 Hz
SCANS	545
ACQTM	1.0643 sec
PD	2.0000 sec
PW1	2.93 usec
IRNUC	1H
CTEMP	20.7 c
SLVNT	CDCL3
EXREF	77.16 ppm
BF	1.02 Hz
RGAIN	60

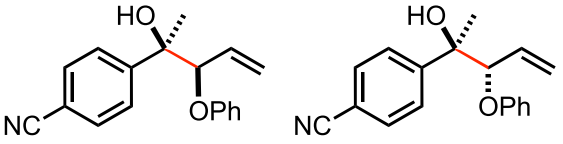

major *minor*

8av

\\172.20.1.218\share_Instruments_Data_Data_NMR_files\Arii\NMRdata_complete\SA1056-proton-1-1.als

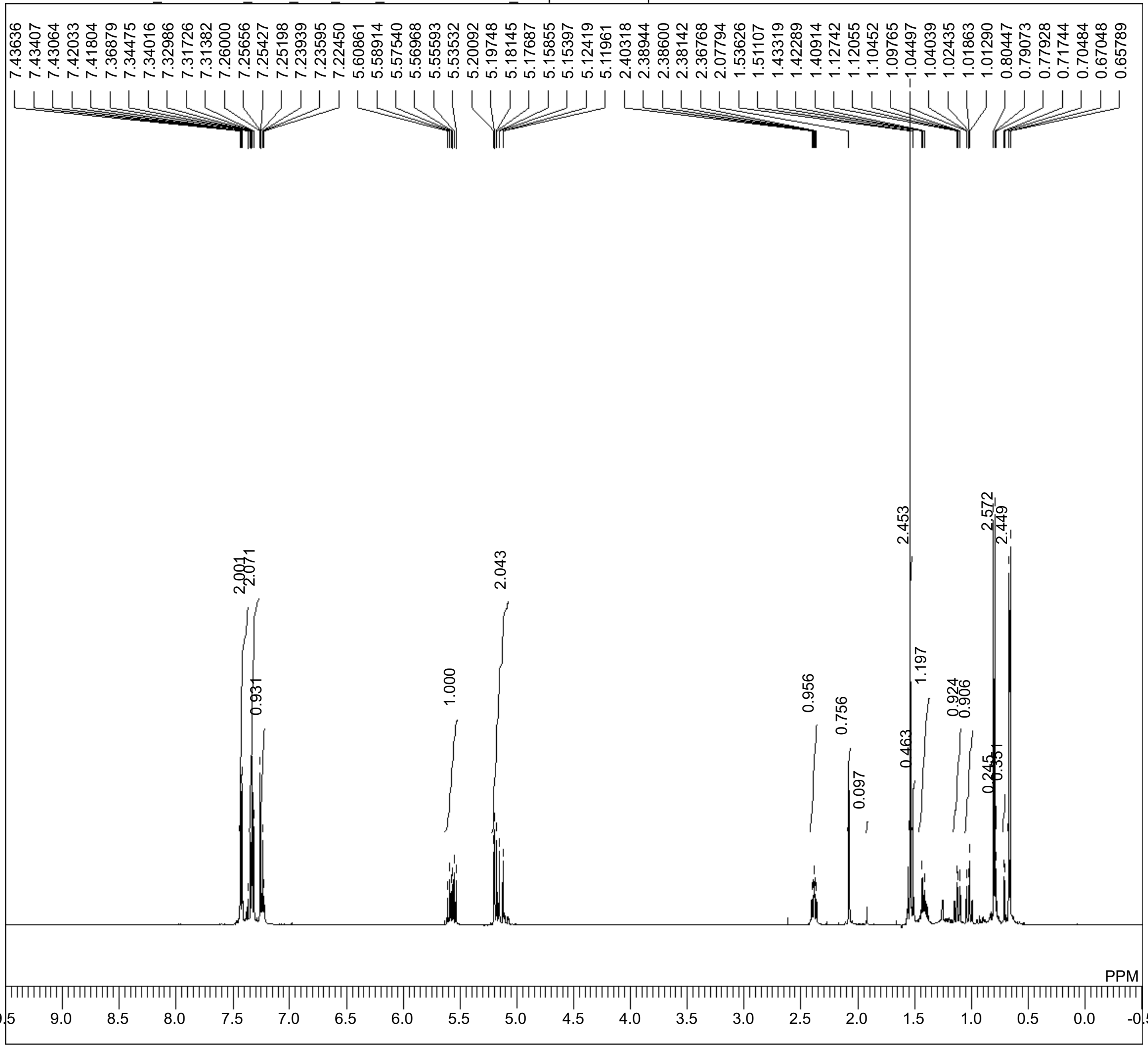

DFILE	SA1056-proton-1-1.als
COMNT	
DATIM	2025-01-25 17:19:18
OBNUC	1H
EXMOD	proton.jxp
OBFRQ	500.16 MHz
OBSET	2.41 KHz
OBFIN	6.01 Hz
POINT	13107
FREQU	7507.51 Hz
SCANS	8
ACQTM	1.7459 sec
PD	5.0000 sec
PW1	5.55 usec
IRNUC	1H
CTEMP	21.4 c
SLVNT	CDCL3
EXREF	7.26 ppm
BF	0.72 Hz
RGAIN	30

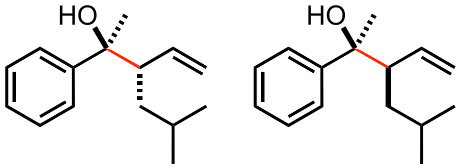

major *minor*

8aw

\\172.20.1.218\share_Instruments_Data_Data_NMR_files\Arii\NMRdata_complete\SA1056-carbon-1-1.als

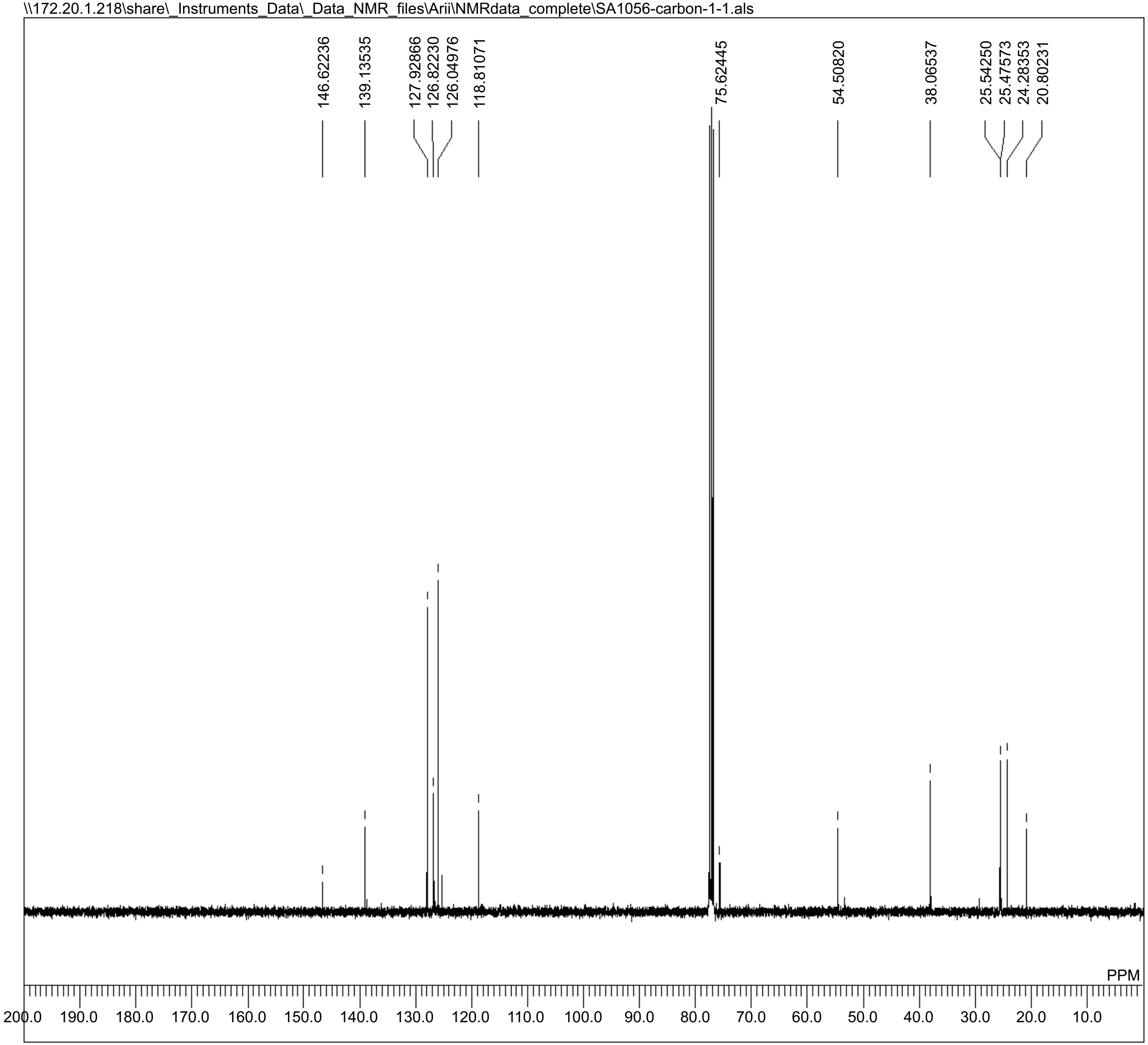

DFILE	SA1056-carbon-1-1.als
COMNT	
DATIM	2025-01-25 13:44:12
OBNUC	13C
EXMOD	carbon.jxp
OBFRQ	98.52 MHz
OBSET	4.64 KHz
OBFIN	8.74 Hz
POINT	26214
FREQU	24630.54 Hz
SCANS	1975
ACQTM	1.0643 sec
PD	2.0000 sec
PW1	2.93 usec
IRNUC	1H
CTEMP	20.6 c
SLVNT	CDCL3
EXREF	77.16 ppm
BF	0.12 Hz
RGAIN	60

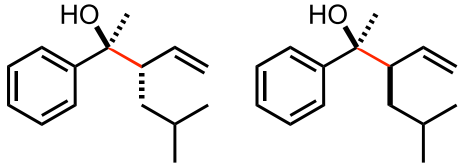

major **8aw** *minor*

\\172.20.1.218\share_Instruments_Data_Data_NMR_files\Arii\NMRdata_complete\SA1063-proton-1-1.als

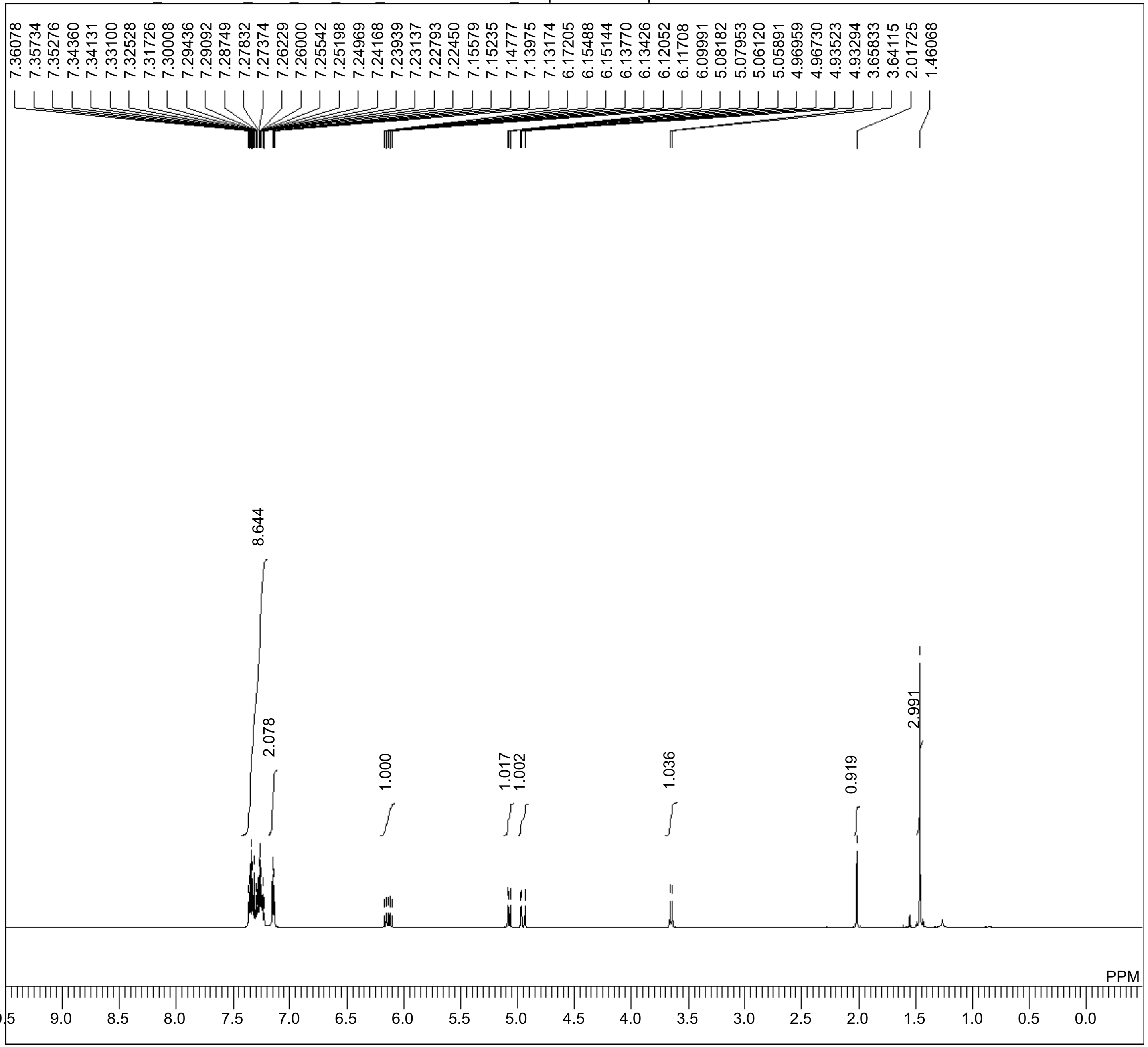

DFILE	SA1063-proton-1-1.als
COMNT	
DATIM	2025-01-25 14:19:46
OBNUC	1H
EXMOD	proton.jxp
OBFRQ	500.16 MHz
OBSET	2.41 KHz
OBFIN	6.01 Hz
POINT	13107
FREQU	7507.51 Hz
SCANS	8
ACQTM	1.7459 sec
PD	5.0000 sec
PW1	5.55 usec
IRNUC	1H
CTEMP	21.2 c
SLVNT	CDCL3
EXREF	7.26 ppm
BF	0.72 Hz
RGAIN	30

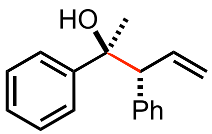

8ax

s

\\172.20.1.218\share_Instruments_Data_Data_NMR_files\Arii\NMRdata_complete\SA1063-carbon-1-1.als

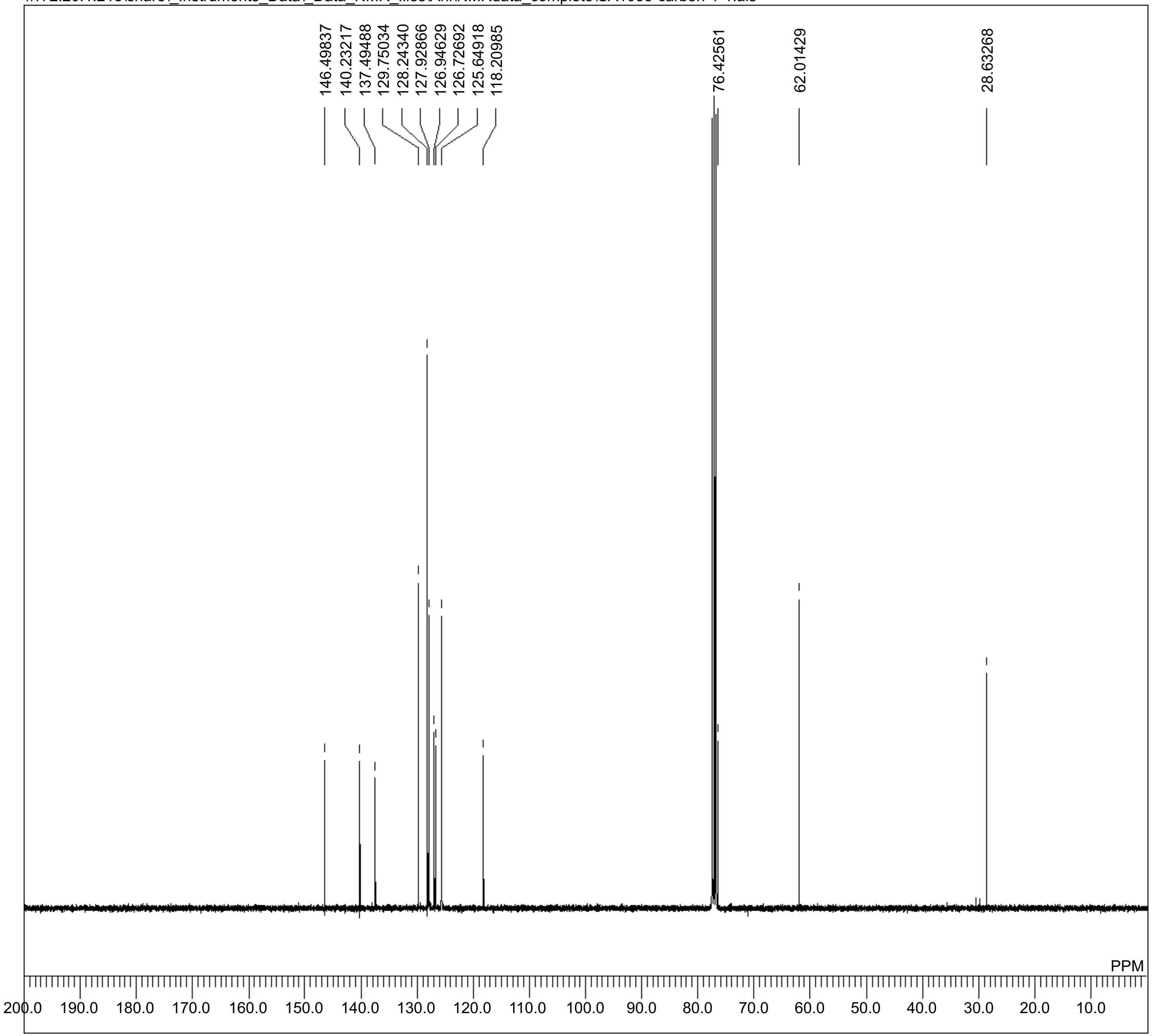

DFILE	SA1063-carbon-1-1.als
COMNT	
DATIM	2025-01-25 07:25:04
OBNUC	13C
EXMOD	carbon.jxp
OBFRQ	98.52 MHz
OBSET	4.64 KHz
OBFIN	8.74 Hz
POINT	26214
FREQU	24630.54 Hz
SCANS	4155
ACQTM	1.0643 sec
PD	2.0000 sec
PW1	2.93 usec
IRNUC	1H
CTEMP	20.5 c
SLVNT	CDCL3
EXREF	77.16 ppm
BF	0.12 Hz
RGAIN	60

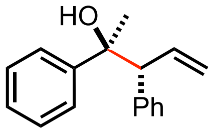

8ax

\\172.20.1.218\share_Instruments_Data_Data_NMR_files\Arii\NMRdata_complete\SA1065-proton-1-1.als

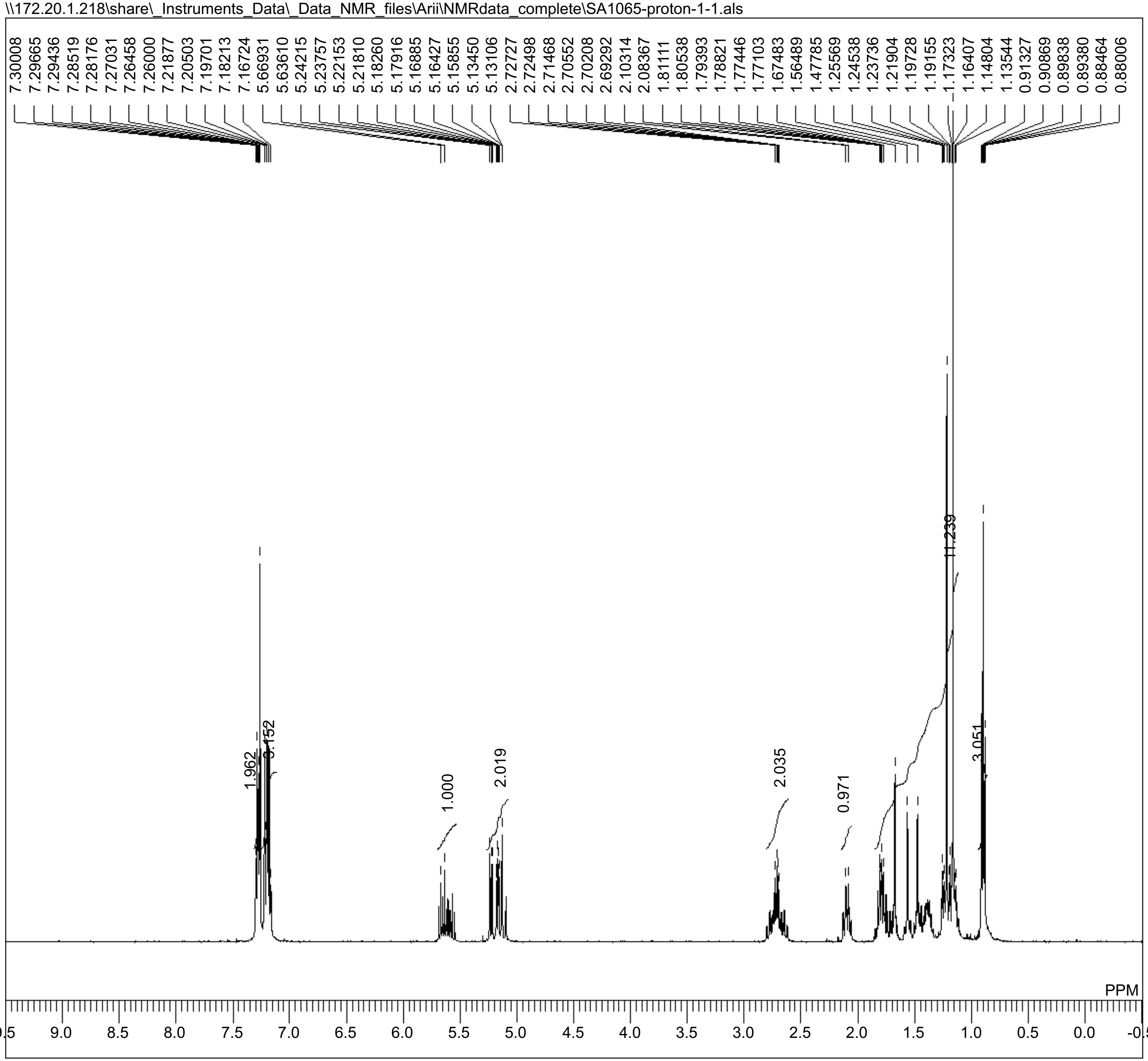

DFILE	SA1065-proton-1-1.als
COMNT	
DATIM	2025-01-25 17:25:50
OBNUC	1H
EXMOD	proton.jxp
OBFRQ	500.16 MHz
OBSET	2.41 KHz
OBFIN	6.01 Hz
POINT	13107
FREQU	7507.51 Hz
SCANS	8
ACQTM	1.7459 sec
PD	5.0000 sec
PW1	5.55 usec
IRNUC	1H
CTEMP	21.5 c
SLVNT	CDCL3
EXREF	7.26 ppm
BF	0.72 Hz
RGAIN	30

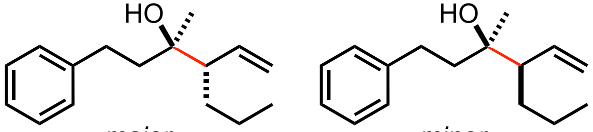

major *minor*

8ay

\\172.20.1.218\share_Instruments_Data_Data_NMR_files\Arii\NMRdata_complete\SA1065-carbon-1-1.als

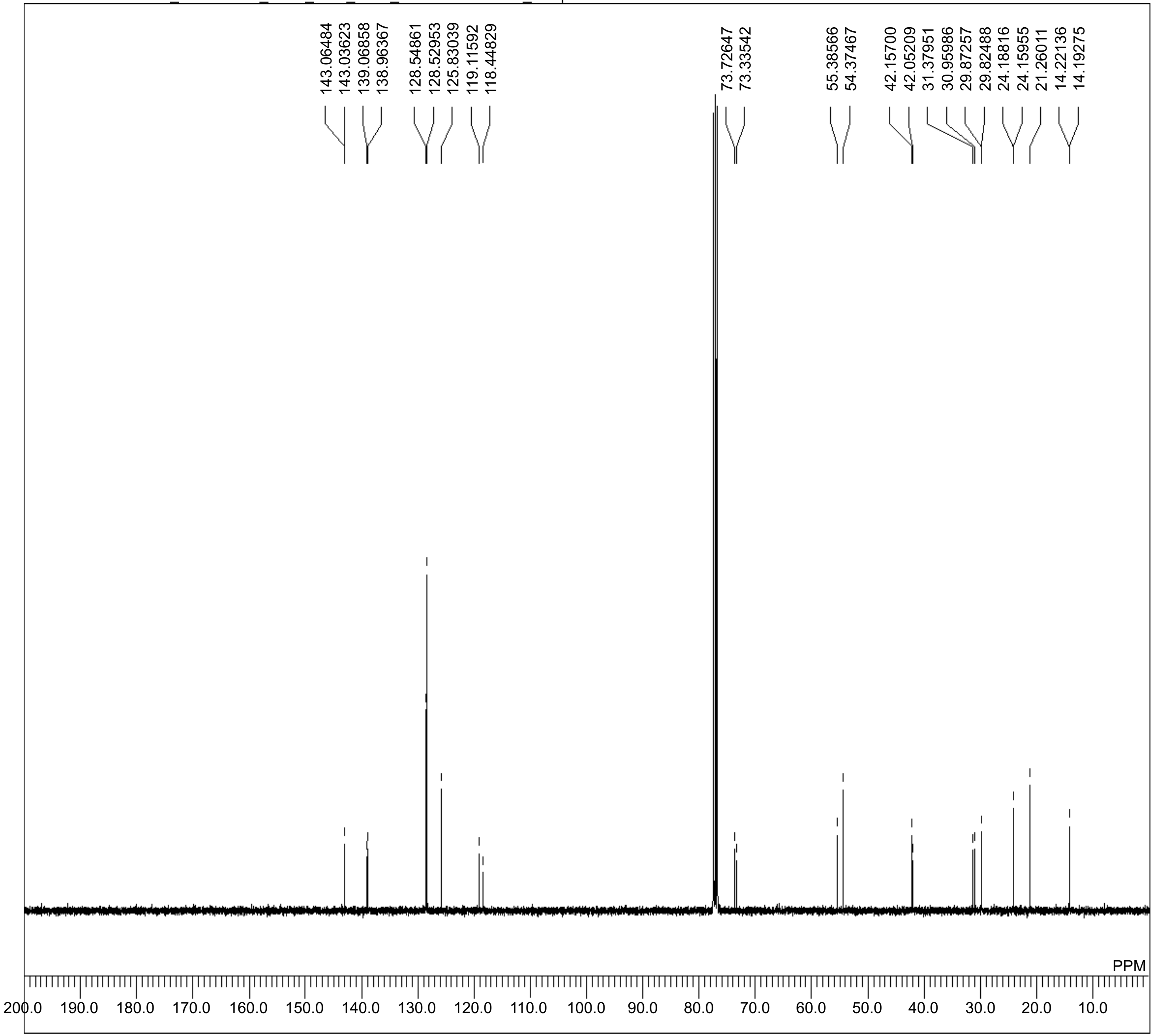

DFILE	SA1065-carbon-1-1.als
COMNT	
DATIM	2025-01-25 02:26:35
OBNUC	13C
EXMOD	carbon.jxp
OBFRQ	98.52 MHz
OBSET	4.64 KHz
OBFIN	8.74 Hz
POINT	26214
FREQU	24630.54 Hz
SCANS	2512
ACQTM	1.0643 sec
PD	2.0000 sec
PW1	2.93 usec
IRNUC	1H
CTEMP	20.5 c
SLVNT	CDCL3
EXREF	77.16 ppm
BF	0.12 Hz
RGAIN	60

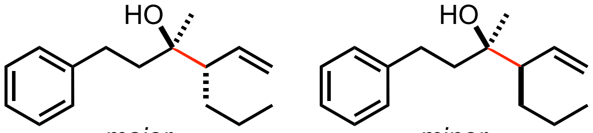

major *minor*

8ay
